# Numerical simulation of landscape ecological river flow structure based on vegetation patch distribution and fragmentation

**DOI:** 10.3389/fpls.2024.1424566

**Published:** 2024-10-10

**Authors:** Jingzhou Zhang, Shengtang Zhang, Shufang Li, Zicheng Yu, Wenjun Wang, Wenhao Zhao, Guohao Li, Zheng Zhou

**Affiliations:** ^1^ School of Water Conservancy and Hydroelectric Power, Hebei University of Engineering, Handan, China; ^2^ Hebei Key Laboratory of Smart Water Conservancy, Hebei University of Engineering, Handan, China; ^3^ College of Earth Science and Engineering, Shandong University of Science and Technology, Qingdao, China; ^4^ College of Water Conservancy and Architectural Engineering, Shihezi University, Shihezi, China

**Keywords:** river wetland, landscape vegetation, patch coverage, patch fragmentation, numerical simulation

## Abstract

The self-organizing biological characteristics of vegetation and human activities lead to the disruption of the continuous spatial attributes of natural watersheds, which are significant factors affecting river wetland ecosystems. To clarify the landscape ecological flow characteristics of vegetation patch distribution and fragmentation, this study used the three-dimensional Reynolds stress turbulence model in ANSYS Fluent software. The model considered three vegetation patch coverages under two different submersion states and four fragmentation types of vegetation patches under the same coverage conditions within specific vegetation areas. The flow characteristics of longitudinally discontinuous rigid vegetation patches, occupying half of the width of the river channel, were numerically simulated. The model’s applicability was verified by indoor open-channel flume experiments. The results indicated that: (1) The streamwise velocity in vegetated areas is significantly lower than in non-vegetated areas, and the difference in flow capacity between vegetated and non-vegetated areas increases with patch coverage and fragmentation degree. (2) In the non-submerged state, the maximum Reynolds stress in the vegetated area is located at the bottom of the vegetation and is negatively correlated with patch coverage but positively correlated with fragmentation degree. In the submerged state, the maximum Reynolds stress is located near the top of the canopy and is positively correlated with both patch coverage and fragmentation degree. (3) The longitudinal turbulent kinetic energy in the vegetated area is significantly higher than in the non-vegetated area. In the non-submerged state, the turbulent kinetic energy in the vegetated area is negatively correlated with patch coverage but positively correlated with fragmentation degree. In the submerged state, the turbulent kinetic energy of the longitudinal distribution in the free layer of the vegetated area is positively correlated with patch coverage, negatively correlated with fragmentation degree, and is only reflected in the upstream vegetation area.

## 1 Introduction

As an important part of the wetland ecosystem, rivers are essential conduits for the circulation of materials, and the transmission of energy and information in nature (Dutykh et al., 2011a, b), making them a focal area of global wetland ecological restoration efforts. As a fundamental component of river wetland ecosystems, aquatic vegetation serves as a key indicator of the health of ecological rivers (Curran and Hession, 2013; Zdankus et al., 2016; Tinoco et al., 2020; Pan et al., 2023). River vegetation plays a role in regulating runoff (Temmerman et al., 2013; Kazemi et al., 2018), climate regulation (Aberle and Järvelä, 2013; Boothroyd et al., 2017), carbon fixation (Förster et al., 2021; Chen et al., 2023), pollution purification (Chen et al., 2012; Gurnell, 2014), biodiversity conservation (Bennett et al., 2008; Liu et al., 2023), and ecological integration in river wetland landscape planning (Grabowski et al., 2014; Huai et al., 2019), among other ecological services. Currently, with the global emphasis on river wetland ecological protection and sustainable development, the strategic allocation of vegetation is being implemented in various ecological restoration projects. While the coverage of vegetation in floodplains, and even in main channels, has significantly increased, it has also directly altered the flow structure of ecological river channels, influencing flood runoff processes, and the relationship between channel storage and discharge during floods and droughts (Zhang and Su, 2008; Rominger and Nepf, 2011; Veĺısková et al., 2017). Consequently, this has become a prominent topic in river wetland planning, management, and ecological restoration (Mulahasan and Stoesser, 2017; Farzadkhoo et al., 2018).

In recent years, with the global focus on the governance of water resources, water ecology, and the water environment, the restoration of wetland river and lake ecosystems has accelerated, leading to a more diverse and complex aquatic vegetation community (Liu et al., 2020a; Tang et al., 2023). While enhancing the ecological functions of rivers, this has also altered the internal flow structure and flood discharge characteristics. Consequently, many scholars have conducted extensive research on the flow characteristics of vegetation under various river conditions. Regarding studies on vegetation belts covering entire river channels, Murphy et al. (2007) proposed a method for predicting the longitudinal dispersion coefficient under submerged rigid vegetation using the slow-flow zone model. This model divides the water flow vertically into an upper fast-flow zone and a lower slow-flow zone. Xu et al. (2022) investigated the dynamic flow response of flexible vegetation with varying plant flexibility and spacing through large eddy simulations. Their results showed that flexible vegetation is subject to complex flow-induced vibrations. Wu et al. (2023) experimentally studied the effects of different types of flexible vegetation on flow field structure and turbulence characteristics under unidirectional flow conditions and developed a turbulent kinetic energy model. The *TKE* model accurately predicts the turbulent kinetic energy of vegetation with varying flexibility under unidirectional flow conditions, as well as the sediment and material transport characteristics influenced by underwater vegetation.

The continuous distribution of vegetation along the sides of rivers is a prevalent landscape characteristic in wetlands, intertidal zones, natural rivers, and artificial ecological rivers (Shan et al., 2018; Truong and Uijttewaal, 2019). In studies focused on the continuous distribution of vegetation zones along river channels, Liu and Shan (2019), (Liu et al., 2020a) investigated the longitudinal profile flow velocity of rigid, non-submerged, continuous vegetation distributed in the middle of the river through experiments. They proposed an analytical model for predicting the flow velocity within the channel and the longitudinal profile inside the vegetation for non-submerged, rigid, rectangular vegetation groups, providing a theoretical basis for estimating the mechanisms of sediment or organic matter accumulation and reduction in vegetated areas. Yan et al. (2022) explored the three-dimensional hydrodynamic structure of submerged vegetation on one side of the flume through experiments. They visualized the results of multi-dimensional (vertical and horizontal) large-scale coherent vortices, mean flow fields, and turbulent flow fields from the top and side of the vegetation by varying vegetation density, flow rate, and water depth. Caroppi et al. (2021) used reconfigurable vegetation, similar to natural shrubs and grasses, as well as rigid columns, to conduct a unique comparative experiment on the flow structure of riparian vegetation. Their study demonstrated that although the lateral distribution of flow characteristics is highly similar in terms of the shear layer differential ratio, average vegetation resistance, and large-scale vortices, the flexibility mechanism akin to natural vegetation has a significant effect on flow characteristics at the interface.

However, due to the influence of seasonal, temporal, and spatial changes, as well as the self-organizing biological characteristics of vegetation and human activities, the spatial variability of river wetland vegetation communities is exacerbated, and vegetation typically exhibits a spatial configuration of longitudinal discontinuous patches along the riverbank. Regarding the study of river channels with discontinuous distributions of vegetation patch groups, Anjum and Tanaka (2020) conducted a numerical simulation of the flow characteristics of longitudinal discontinuous double-layer rigid vegetation patches occupying half the width of the river channel. The study revealed that the flow distribution of double-layer submerged vegetation was more complex than that of non-submerged vegetation. Li et al. (2020) investigated the flow characteristics of single-row discontinuous non-submerged vegetation patches in open channels through experiments, concluding that the flow field is divided into three main areas: the boundary influence area, the mixed layer area, and the uniform non-vegetated area. Zhang et al. (2022a) performed an open-channel experiment on the distribution of partially discontinuous rigid vegetation patches and explained the influence of stem thickness combinations within vegetation patches on flow characteristics. Additionally, previous studies have primarily focused on the flow field effects of single vegetation patches and multiple patches (Meire et al., 2014; Chang et al., 2017; Tny et al., 2019).

The transformation of vegetation communities from continuous to interspersed vegetation patch networks is a concrete manifestation of river habitat fragmentation (Tanner, 2003; Aina et al., 2021). Currently, modern watersheds are impacted by highly complex human activities, and the self-organizing behavior of aquatic vegetation under the constraint of nutrient resources (Ruiz-Reynés et al., 2017) has led to the formation of fragmented patches on the surface of watersheds. This has disrupted the spatial attributes of continuous changes in natural watersheds, accelerated the evolution from natural watersheds to modern landscape watersheds, and caused abnormal responses in river runoff patterns (Folkard, 2005).

**Figure 1:**
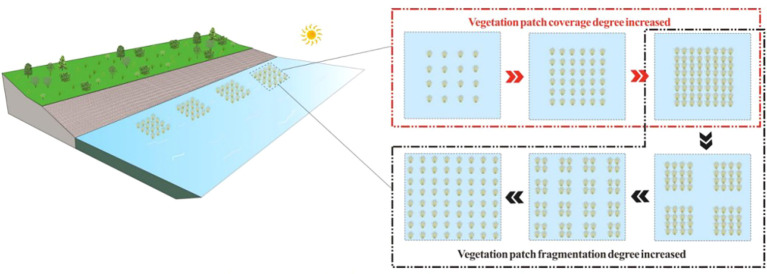
Schematic diagram of landscape ecological river vegetation distribution.

However, there is a relative lack of research on the fragmentation of vegetation patches considering the heterogeneity of vegetation landscapes in river channels, and how the landscape pattern of vegetation patches affects changes in stream-scale flow structure remains unclear (Liu et al., 2020b). In particular, the influence of the degree of patch fragmentation with the same vegetation quantity configuration on flow characteristics, under the challenge of sustainable water resources and soil and water flow carrying capacity, is still underexplored. This creates significant uncertainty in the ecological restoration and planning of river wetlands, vegetation landscape integration, and the sustainable utilization and management of watershed water resources. The distribution of partially discontinuous vegetation patches in ecological river channels is a typical example of modern watershed landscape planning (**Figure 1**). Due to changes in patch vegetation coverage and fragmentation, the grid of watershed surface units is regenerated, leading to the spatial variation of watershed parameters. Therefore, it is necessary to comprehensively consider the influence of partially discontinuous vegetation patch distribution on river flow characteristics to accurately replicate the modern riparian planning environment and provide an effective scientific basis for the management and restoration engineering design of landscape ecological river wetlands.

In this study, the three-dimensional flow structure of vegetation communities in partially discontinuous patches of rectangular open channels was simulated numerically. By altering the coverage and fragmentation degree of unit vegetation patches, the following aspects were examined: (1) The impact of the coverage and fragmentation of partially discontinuous vegetation patches on the longitudinal, transverse, and vertical streamwise velocity structures at specific locations under different submerged conditions. Additionally, the velocity distribution characteristics and energy exchange of the overall river channel were analyzed, combined with the spatial distribution of horizontal, vertical, and streamwise velocity isolines. (2) The effects of vegetation patch coverage and fragmentation characteristics on the vertical distribution of Reynolds stress at specific locations under different submerged conditions. This revealed changes in turbulent kinetic energy in vegetated and non-vegetated areas and clarified the effects of vegetation patch coverage and fragmentation characteristics on the turbulent characteristics of river flow.

## 2 Materials and methods

### 2.1 Modelling setup and boundary conditions

**Figure 2:**
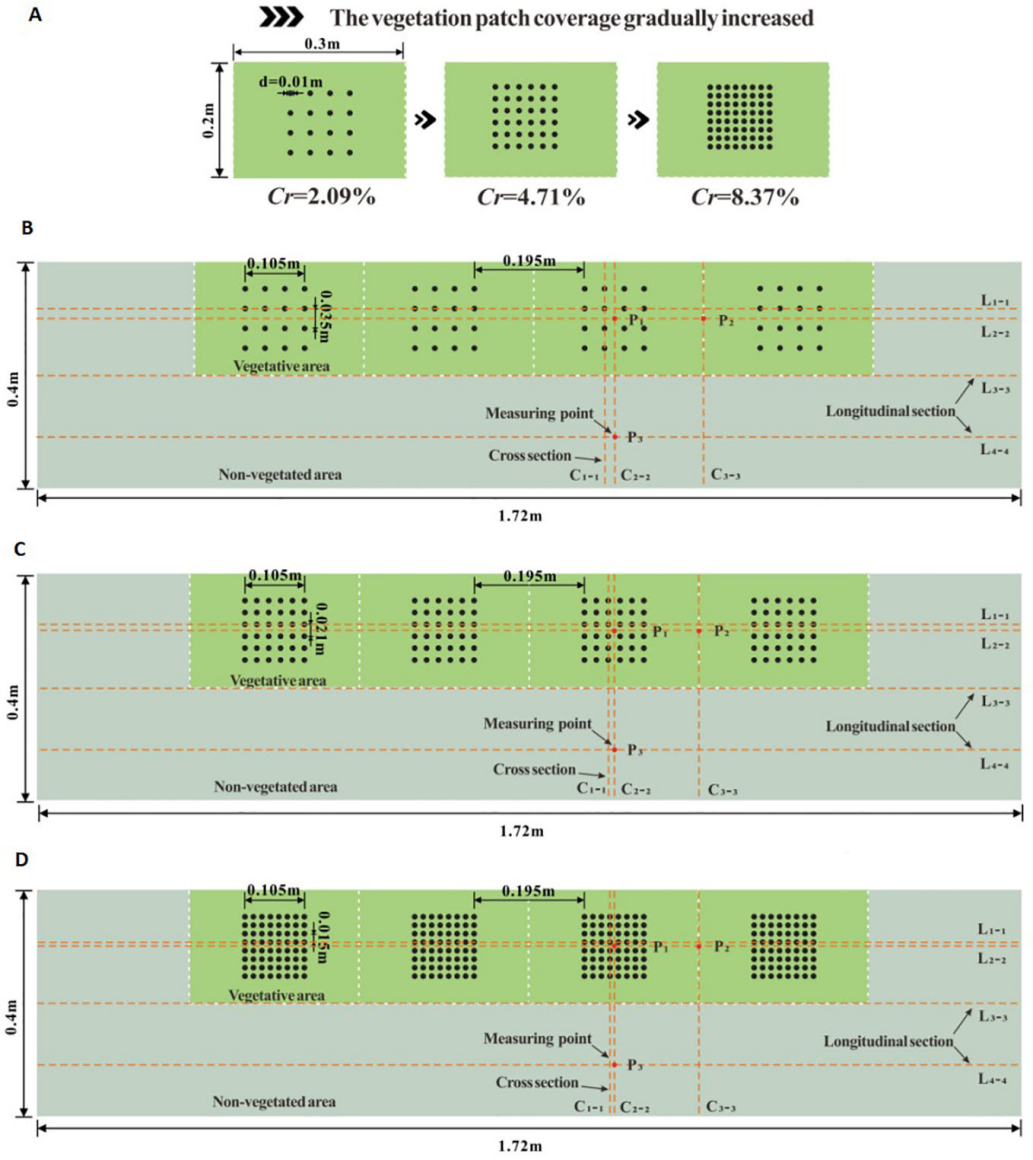
The schematic diagram of different coverage of unit vegetation patches **(A)** and schematic diagram of floor layout of numerical calculation areas with different vegetation patches **(B–D)**.

**Figure 3:**
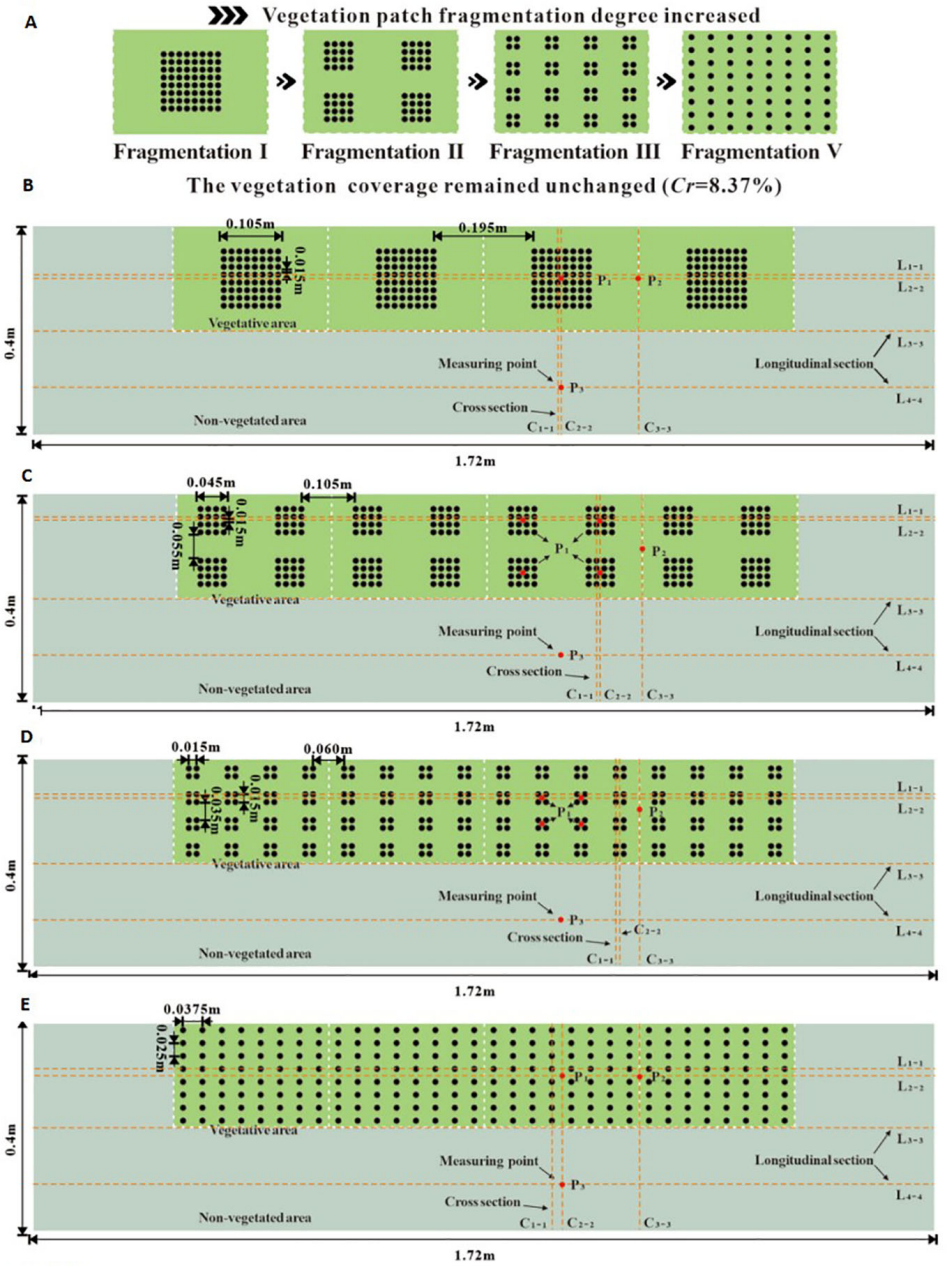
The schematic diagram of different fragmentation types of unit vegetation patches **(A)** and schematic diagram of floor layout of numerical calculation areas with different vegetation patch fragmentation **(B–E)**.

With the rapid development of computer technology, numerical simulation offers many advantages, such as cost-effectiveness, predictive capability, data processing, and interdisciplinary applications. As a result, numerical simulation theory has been extended to the study of river channels (Dutykh et al., 2011c). In this study, ANSYS Fluent software was used for numerical simulation, with the Reynolds stress model selected, and the Navier-Stokes equation discretely solved based on the finite volume method. The numerical modeling focused on longitudinal, partially discontinuous, rigid vegetation patches along the side of an open channel, where the width of the vegetated area accounts for half the river width. The calculation domain was set to a length of 1.72 m and a width of 0.4 m. **Figures 2**, **3** show schematic diagrams of the floor layout in the calculation area. For the simulated vegetation, plants are typically represented as water-blocking cylinders in numerical simulations and experiments (Zhang et al., 2022b). In this study, cylindrical simulated vegetation with a height (*h_v_*) of 0.08 m and a stem thickness (*d*) of 0.01 m was selected. The distribution of vegetation patch coverage and fragmentation degree under two submerged states (non-submerged and submerged) was considered. The *x*, *y*, and *z* axes represent the flow direction, and horizontal, and vertical coordinate systems, respectively. The vegetated patch side of the open channel is referred to as the vegetated area, while the non-vegetated side is referred to as the non-vegetated area. The region below the vegetation top is defined as the vegetation layer in the vertical direction, and the area above the vegetation top is defined as the free layer.

In this model, the pressure-based segregated solver was selected, and the SIMPLEC algorithm was used for pressure-velocity coupling. The relaxation factors for turbulent kinetic energy and pressure were set to 0.7 and 0.3, respectively. The velocity relaxation factor was set to 0.7, the Reynolds stress factor to 0.5, and both the turbulent kinetic energy and turbulent dissipation rate were set to 0.8. Gradient discretization was performed using the element-based least squares method, while pressure interpolation employed the standard method, and the flow direction parameter interpolation used the second-order upwind scheme. The minimum residual value for each equation was set to 1×10^-5^, meaning the iteration process ended when the calculated residual value was below this threshold. The inlet boundary condition of the model was set as a velocity inlet with a velocity of 0.3 m/s, and the outlet boundary was defined as a pressure outlet. The cylindrical surface and walls were set as no-slip solid wall boundaries. For the free water surface, the stable and easily convergent rigid cap assumption method was applied. The model was solved using these settings.

To further investigate the effect of vegetation patch coverage on flow characteristics, the distance between adjacent plants was reduced, increasing the number of plants in the patch while maintaining the same patch shape and size. For the specific vegetation area in this study (the average area of unit patches being 0.3 m × 0.2 m), the vegetation patch coverage was set to 2.09%, 4.71%, and 8.37%, respectively. **Figure 2** shows the layout diagram of the different vegetation patches.

Additionally, due to the combined influence of the self-organizing biological characteristics of vegetation and human planning, the scientific planning and transformation of ecological river vegetation landscapes have altered the spatial attributes of ecological rivers. Under unique regional water constraints, different vegetation distribution patterns have varying effects on river flow characteristics. In this study, the landscape heterogeneity of vegetation patches was considered, and unit vegetation patches under the same coverage conditions were divided into four distinct fragmentation types. Four levels of vegetation patch fragmentation were established. The side lengths of the first three unit square patches were 0.115 m, 0.055 m, and 0.025 m, respectively. The fourth type involved the most commonly studied uniform plant distribution (plant spacing of 0.0375 m × 0.025 m) to investigate the effect of patch fragmentation characteristics on river flow characteristics under consistent patch coverage conditions. **Figure 3** presents a schematic diagram of the layout for the different degrees of vegetation patch fragmentation.

### 2.2 Validation of numerical simulation methods

The validity of the numerical model used in this study was verified through an indoor open-channel flume experiment. The experimental flume was 5 m long, 0.4 m wide, and 0.3 m deep. A plastic cylindrical rod with a diameter of 0.01 m and a height of 0.08 m was used to simulate rigid vegetation. The vegetation distribution in the flume was consistent with the model. To ensure the experimental conditions aligned with the model settings, the flow discharge under submerged vegetation conditions was set to 0.012 m^3^/s. By adjusting the tailgate at the downstream end of the flume, the water depth was regulated to match the water depth at the 1.64 m position of the flume with the inlet position in the model, ensuring an inlet velocity of 0.3 m/s. To achieve flow homogenization, measurements were taken every 10 minutes during the experiment. Additionally, a 3D ADV was used to measure the velocity at position P_2_ under submerged conditions, as shown in **Figures 2** and **3**, to verify the model’s applicability.

**Figure 4:**
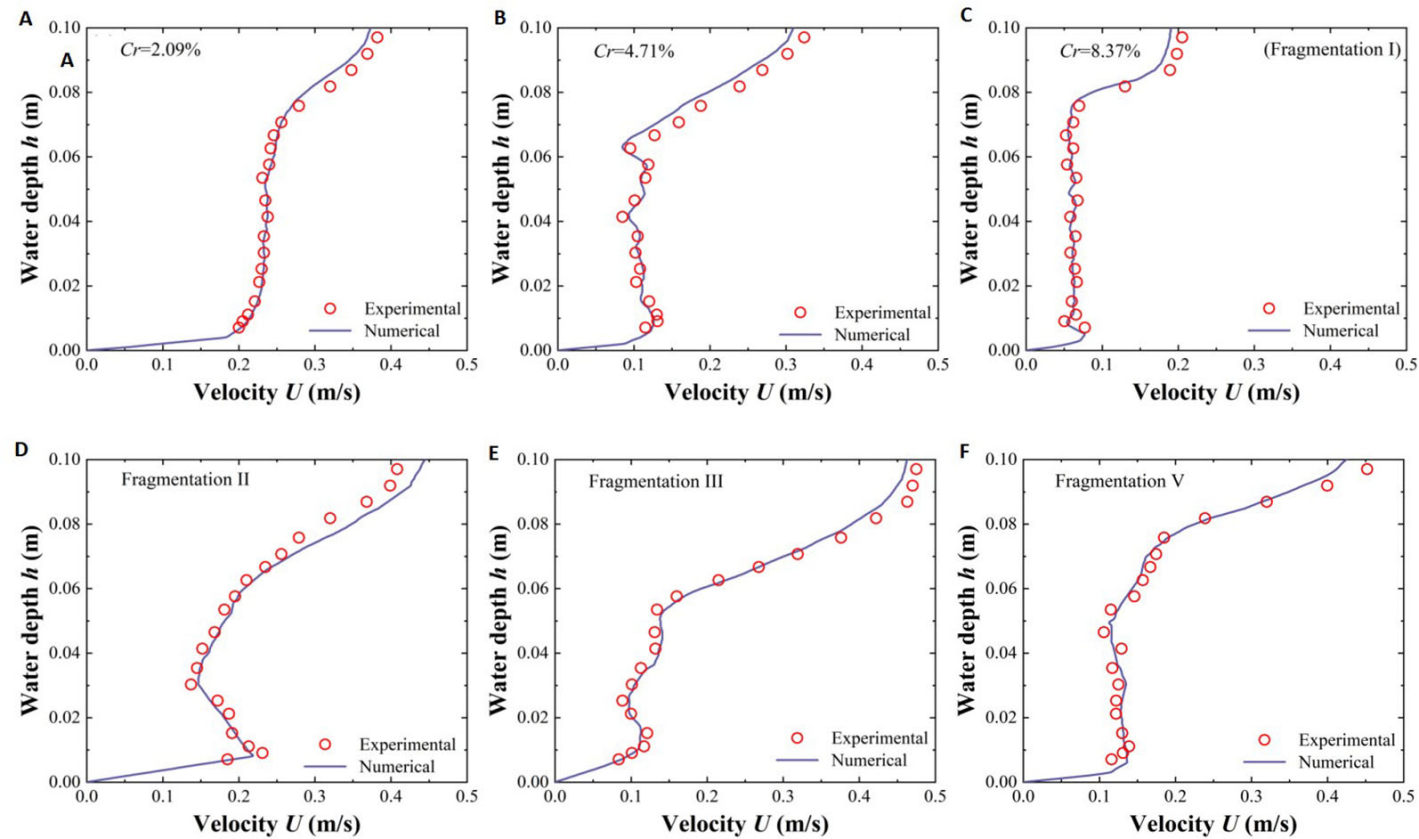
Comparison diagram of the relationship between P_2_ position velocity *U* and water depth *h* verified by the experiment and numerical simulation, different coverage of vegetation patches **(A–C)**, and different fragmentation degrees of vegatation patches **(C–F)**.

The experimental results closely aligned with the numerical calculation results (**Figure 4**), confirming the effectiveness of the numerical model. However, there was a minor difference between the experimental and numerical results (the average error was 8.09%, **Table 1**), likely due to modeling simplifications or inevitable experimental measurement errors.

**Table 1:** Error ratio between simulated value and experimental value under different water depth conditions at P2 position under submerged state.

Patch coverage and fragmentation degree	h (m)	0.025	0.030	0.035	0.041	0.047	0.054	0.058	0.063	0.067	0.071	0.076	0.082	0.087	0.092	0.097
*Cr*=2.09%	*δ*	4.07%	3.47%	3.51%	7.74%	5.39%	6.40%	12.07%	10.38%	7.04%	6.48%	7.00%	7.45%	6.77%	6.93%	4.03%
*Cr*=4.71%	*δ*	2.39%	2.04%	3.85%	4.62%	1.94%	6.17%	10.48%	20.12%	16.16%	12.86%	14.81%	13.41%	13.30%	10.61%	6.84%
*Cr*=8.37% (Fragmentation I)	*δ*	9.97%	13.98%	24.58%	10.89%	20.82%	17.95%	35.29%	4.98%	1.81%	11.59%	3.60%	16.04%	8.76%	11.44%	12.07%
*Cr*=8.37% (Fragmentation II)	*δ*	8.40%	2.34%	9.02%	2.07%	0.00%	5.73%	3.86%	11.39%	10.31%	10.80%	10.80%	9.86%	2.13%	1.01%	0.99%
*Cr*=8.37% (Fragmentation III)	*δ*	3.49%	5.20%	2.34%	3.57%	3.59%	4.65%	7.47%	3.28%	5.53%	5.09%	7.41%	8.51%	6.77%	6.02%	1.44%
*Cr*=8.37% (Fragmentation V)	*δ*	3.39%	14.68%	11.43%	5.74%	11.45%	4.58%	5.43%	9.60%	0.76%	5.13%	14.75%	18.15%	6.43%	5.13%	6.67%

δ is the error ratio of the simulated value to the experimental value.

## 3 Results

### 3.1 Velocity distribution

#### 3.1.1 Streamwise velocity distribution of specific longitudinal section

**Figure 5:**
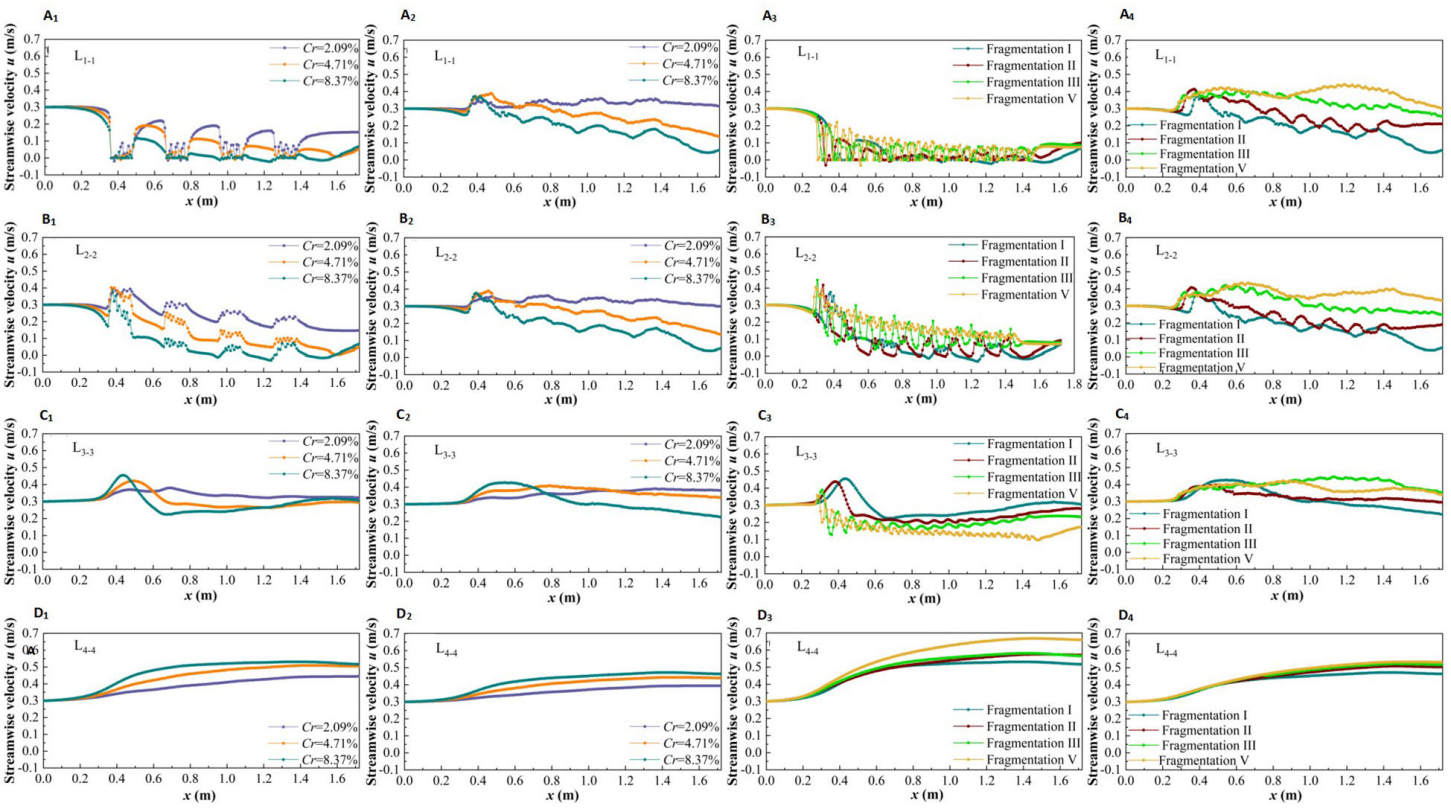
The longitudinal streamwise velocity distribution of vegetation patches with different coverage and fragmentation degree at specificic positions under two submerged states, non submerged state **(A_1_–D_1_, A_3_–D_3_)**, submerged state **(A_2_–D_2_, A_4_–D_4_)**.

To better describe the change in streamwise velocity (*u*) within partially discontinuous vegetation patches, the following areas were selected for study: the vegetation area through the vegetation column (L_1-1_), the inter-plant water area in the unit patch (L_2-2_), the river width center line (L_3-3_), and the non-vegetation area width center line (L_4-4_). The variation in *u* with specific water depths under different submerged conditions (non-submerged state *h=*0.05 m, submerged state *h=*0.09 m) is shown in **Figure 5**.

Under non-inundation conditions and varying patch coverage, the changes in *u* at specific locations are illustrated in **Figures 5A_1_-D_1_**. It can be observed that the *u* value in the longitudinal section (L_1-1_) through the vegetation cylinder is smaller than that in the gap area behind it (**Figure 5A_1_**), while the *u* value in the longitudinal section (L_2-2_) in the flow area between the two plants is larger than that in the gap area behind it (**Figure 5B_1_**). Additionally, the *u* value in the vegetation patch area is serrated due to the influence of the wake structure of individual plants. Furthermore, for the vegetation area (**Figures 5A_1_-B_1_**), the *u* value decreases with increasing patch coverage along the path. In the transition area between vegetation and non-vegetation (**Figures 5C_1_, L_3-3_**), *u* shows an increasing-decreasing-slowly increasing trend, eventually tending towards the initial flow velocity along the path. In the non-vegetated area (**Figures 5D_1_, L_4-4_**), *u* exhibits a trend of gradual increase along the path, with the relationship between *u* and patch coverage being opposite to that in the vegetated area, i.e., *u* increases with increasing patch coverage.

The variation in *u* for different types of vegetation fragmentation at specific positions along the path under non-submerged conditions is shown in **Figures 5A_3_–D_3_**. For the longitudinal section (L_1-1_) (**Figure 5A_3_**), the flow entering the vegetation area exhibits *u* value lower than the initial flow velocity, and *u* decreases with increasing path length. As the fragmentation degree of the vegetation patch increases, the amplitude of *u* continuously increases, resulting in an overall rise in streamwise velocity, though the increase is not significant. In the longitudinal section (L_2-2_), the *u* value also decreases with increasing path length. However, with greater fragmentation of the vegetation patch, *u* increases, and both its frequency and amplitude rise. When the vegetation is evenly distributed across the area, the amplitude of the *u* value suddenly decreases (**Figure 5B_3_**). In the transition area between vegetation and non-vegetation (**Figures 5C_3_, L_3-3_**), *u* decreases with increasing fragmentation degree. In the non-vegetated area (**Figures 5D_3_, L_4-4_**), the flow entering the non-vegetated area has a higher *u* value than the initial flow velocity, and *u* increases with increasing path length and patch fragmentation degree.

To explore the influence of the submerged state on *u*, the variation of free-layer *u* along the path under submerged conditions is shown in **Figures 5A_2_–D_2_, A_4_–D_4_**. Compared with the non-submerged state, the longitudinal continuity of free-layer flow velocity is less disturbed by vegetation, and the longitudinal flow field is relatively uniform. However, due to the small submergence height of vegetation, the influence of vegetation distribution patterns on *u* under submerged conditions still shows some reservations compared to non-submerged conditions. Specifically, the *u* value in vegetation areas is negatively correlated with vegetation patch coverage and path length, and positively correlated with patch fragmentation (**Figures 5A_2_–B_2_, A_4_–B_4_**). In the boundary area between vegetation and non-vegetation, the distribution pattern of patch vegetation has a weaker influence on *u* (**Figures 5C_2_, C_4_**). For non-vegetated areas, *u* is positively correlated with vegetation patch coverage, fragmentation degree, and path length (**Figures 5D_2_, D_4_**).

#### 3.1.2 Streamwise velocity distribution in a specific cross-section direction

**Figure 6:**
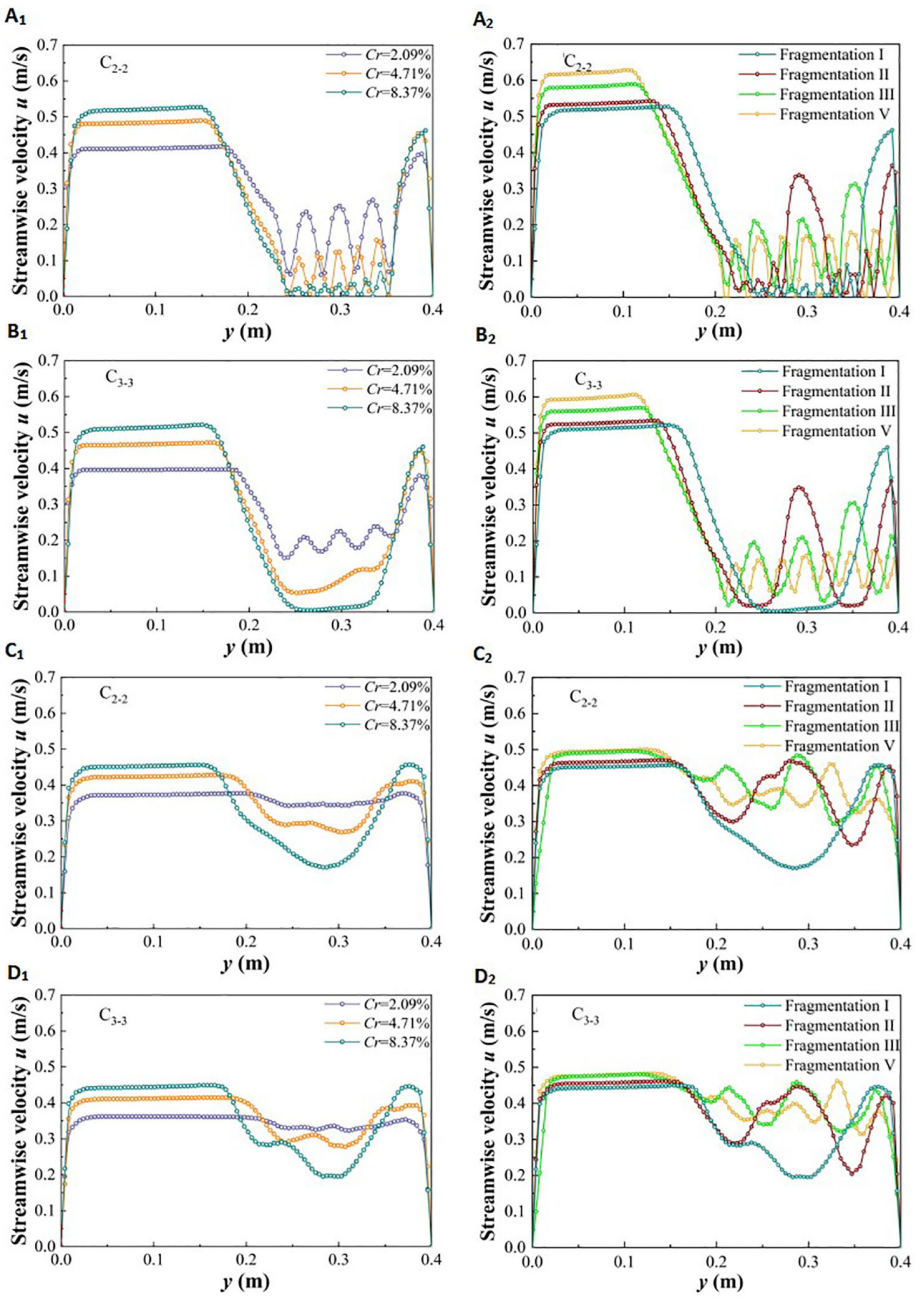
Variation of streamwise velocity in specific y section under different submerged states: non-submerged **(A_1_, B_1_, A_2_, B_2_)**, submerged state **(C_1_, D_1_, C_2_, D_2_)**.

For discontinuous vegetation patches on one side of a distributed river, changes in vegetation coverage and fragmentation will have varying effects on the streamwise velocity in both the lateral vegetation water-blocking area and the non-vegetated flow-through area. The feature cross-section C_1-1_ in the middle of the patch and cross-section C_2-2_ in the gap area behind the patch were selected to study changes in the cross-sectional velocity structure. **Figure 6** shows the streamwise velocity at these cross-sections corresponding to changes in vegetation patch coverage and fragmentation degree under different submerged states.

In the non-submerged state, due to the blocking effect of vegetation, *u* value in the non-vegetated area is significantly higher than in the vegetated area (**Figures 6A_1_-B_1_**, **A_2_-B_2_**), indicating a substantial difference in flow velocity at the junction of the two areas. Within the vegetation area, the *u* value behind the plants is relatively low. The *u* value of water flowing through the vegetation area increases abruptly between adjacent plants and in narrow channels between patches. This increase is due to the lateral divergence caused by water flow colliding with plant obstacles.

Additionally, changes in vegetation patch coverage and fragmentation degree on the side of the river channel under non-submerged conditions are important factors influencing the variation in the *u* value across the entire cross-section. Within the vegetation area, as patch coverage increases, the *u* value at the cross-section inside the patch tends to fluctuate frequently, although its overall value decreases continuously (**Figure 6A_1_**). As the degree of fragmentation of vegetation patches increases, fluctuations in the *u* value at the cross-section inside the patch become more frequent, and the velocity difference between the inner patch and the adjacent patch channel decreases (**Figure 6A_2_**). When the flow enters the rear gap area through the interior of the vegetation patch, the amplitude of *u* in the gap area is significantly reduced compared to that in the patch vegetation, but the original change trend is still partially retained (**Figures 6B_1_, B_2_**). This indicates that the influence of the patch on the water flow persists within a certain distance outside the edge of the patch. Meanwhile, influenced by changes in patch coverage and fragmentation degree in vegetation areas, the *u* value in non-vegetated areas is positively correlated with both patch coverage and fragmentation degree.

Compared with the non-submerged state, the difference in *u* value between the vegetation area and the non-vegetated area under submerged conditions is reduced (**Figures 6C_1_-D_1_, C_2_-D_2_**). Additionally, the patch coverage in the vegetation area has a significant effect on the difference in the *u* value of the free layer; specifically, larger vegetation coverage results in lower flow velocity (**Figures 6C_1_-D_1_**). However, the degree of patch fragmentation does not have a significant effect on the difference in the *u* value (**Figures 6C_2_-D_2_**). In the non-vegetated area, the *u* value continues to increase with increasing patch coverage and fragmentation degree in the vegetation area.

#### 3.1.3 Streamwise velocity distribution in specific vertical section direction

The presence of vegetation alters the mixing and energy dissipation effects of river flow, with the unique vertical morphological characteristics of vegetation being the primary factors that cause significant changes in the vertical flow structure. Variations in the coverage and fragmentation degree of vegetation patches lead to notable differences in flow behavior within and around vegetation patches, as well as in non-vegetated areas. Therefore, it is essential to analyze the distribution of streamwise velocity along the water depth at typical locations, namely P_1_ inside the patch, P_2_ between the patches, and P_3_ at a specific point in the center of the non-vegetated channel.

**Figure 7:**
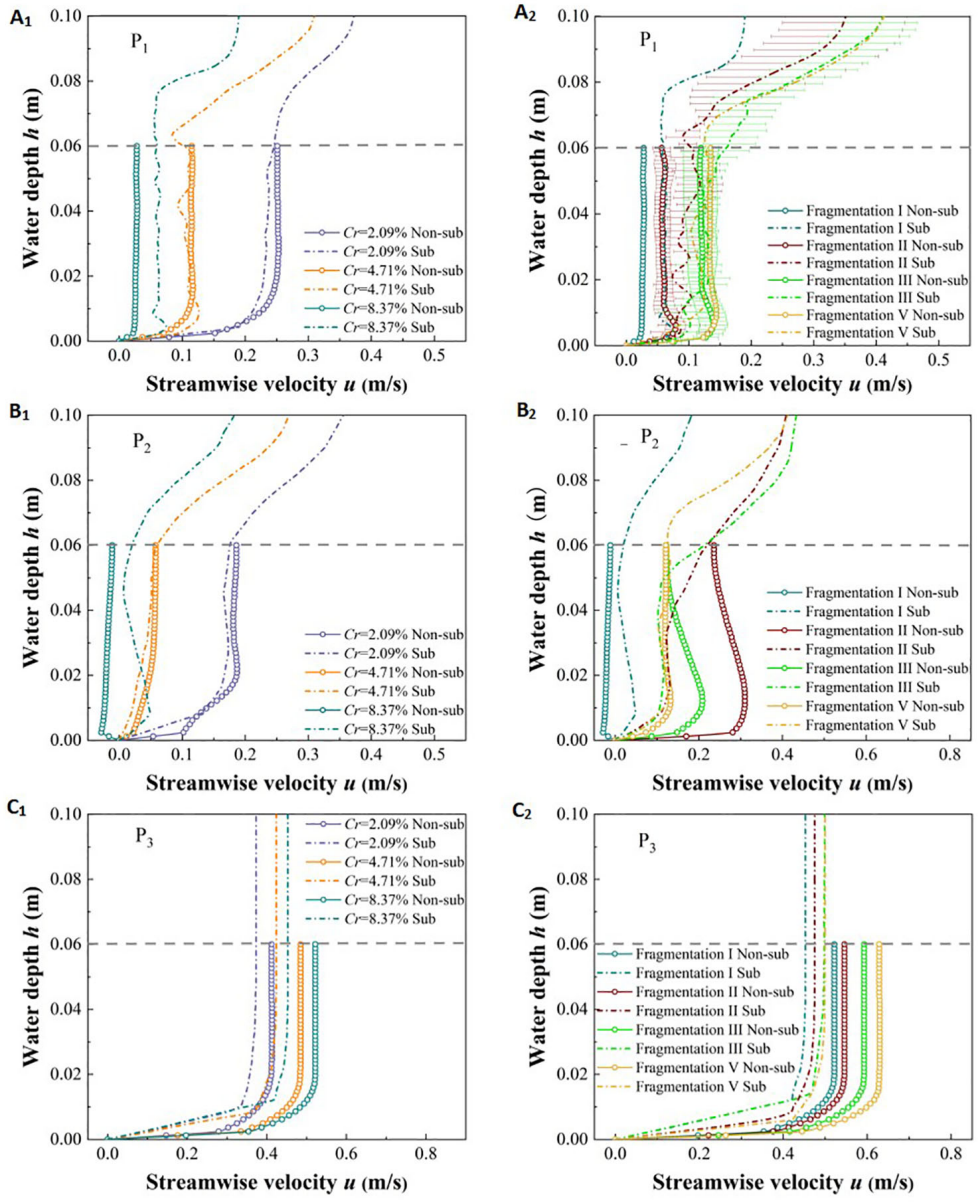
Variation of streamwise velocity along water depth under different submerged states, different coverage of vegetation patches **(A_1_, B_1_, C_1_)**, and different fragmentation degrees of vegetation patches **(A_2_, B_2_, C_2_)**.

Due to the water-blocking effect of vegetation (Liu et al., 2021), in the non-submerged state, the overall *u* value at P_1_ and P_2_ within the vegetation area is less than the initial flow velocity of 0.3 m/s (**Figures 7A_1_-B_2_**). In the non-vegetated area, the overall *u* value at P_3_ is greater than the initial flow velocity of 0.3 m/s due to the absence of direct obstacles (**Figures 7C_1_-C_2_**). In the submerged state, the free layer above the vegetation patch does not obstruct the flow, allowing the flow to develop fully (Wu et al., 2020; Liu and Shan, 2022), which results in a significant increase in the *u* values of P_1_ and P_2_ in the vegetation area near the top of the vegetation patch. Additionally, in the non-submerged state, the *u* values at points P_1_ and P_2_ within the vegetation area exhibit a “*J*-type” distribution along the water depth. In contrast, in the submerged state, the *u* values at points P_1_ and P_2_ show an “*S*-type” distribution along the water depth. Conversely, the overall distribution of *u* values along the water depth at position P_3_ in the non-vegetated area remains “*J*-type” in both submerged states.

Under different coverage conditions of vegetation patches, the *u* values at points P_1_ and P_2_ within the vegetation area decrease as the unit vegetation patch coverage increases. However, when the coverage is below 8.37%, there is minimal difference in the *u* values of the vegetation layer between the two submerged states. When the coverage reaches 8.37%, there is a significant increase in the *u* value of the vegetation layer in the submerged state compared to the non-submerged state (**Figures 7A_1_, B_1_**). For P_3_, the *u* values in both submerged states are positively correlated with vegetation patch coverage, and the overall *u* value of the vegetation layer in the submerged state is smaller than that in the non-submerged state (**Figure 7C_1_**). Therefore, it can be concluded that patch coverage and submerged state are important factors affecting the u value of the vegetation layer.

Under different degrees of patch fragmentation, although the row spacing of adjacent plants within the unit patch is constant, an increase in fragmentation degree leads to a rise in the u value at point P_1_ in the patch under both submerged conditions (**Figure 7A_2_**). The *u* value at point P_2_ between patches decreases overall with the increase in vegetation patch fragmentation under the two inundation states (**Figure 7B_2_**). This occurs because, within a specific vegetation area, increased patch fragmentation shortens the distance between adjacent patches, thus weakening the water flow between them (Colomer et al., 2017). For point, P_3_ in the non-vegetated area (**Figure 7C_2_**), the *u* values in both submerged states are positively correlated with the degree of patch fragmentation, and the *u* values of the vegetation layer under the two submerged states are significantly different. Specifically, the *u* value of the vegetation layer in the submerged state is smaller than that in the non-submerged state. This further confirms that changes in vegetation patch fragmentation under the same coverage conditions are an important factor affecting flow velocity in the flow field.

### 3.2 Spatial distribution of velocity contours in different sections

**Figure 8:**
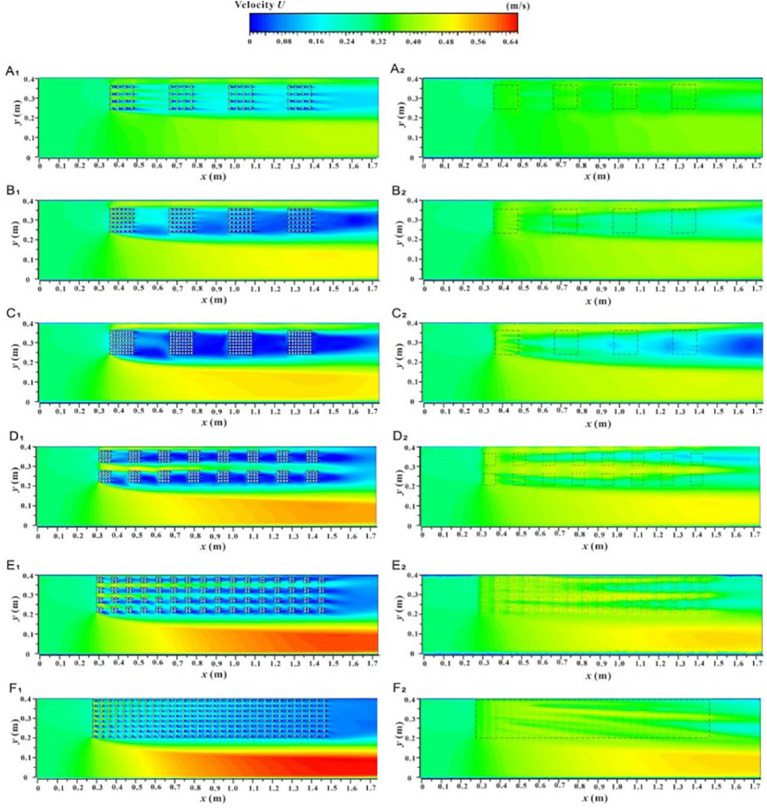
Spatial distribution of velocity contour of *xy* profile unde different submerged states, non-submerged state **(A_1_–F_1_)**, submerged state **(A_2_–F_2_)**.

**Figure 9:**
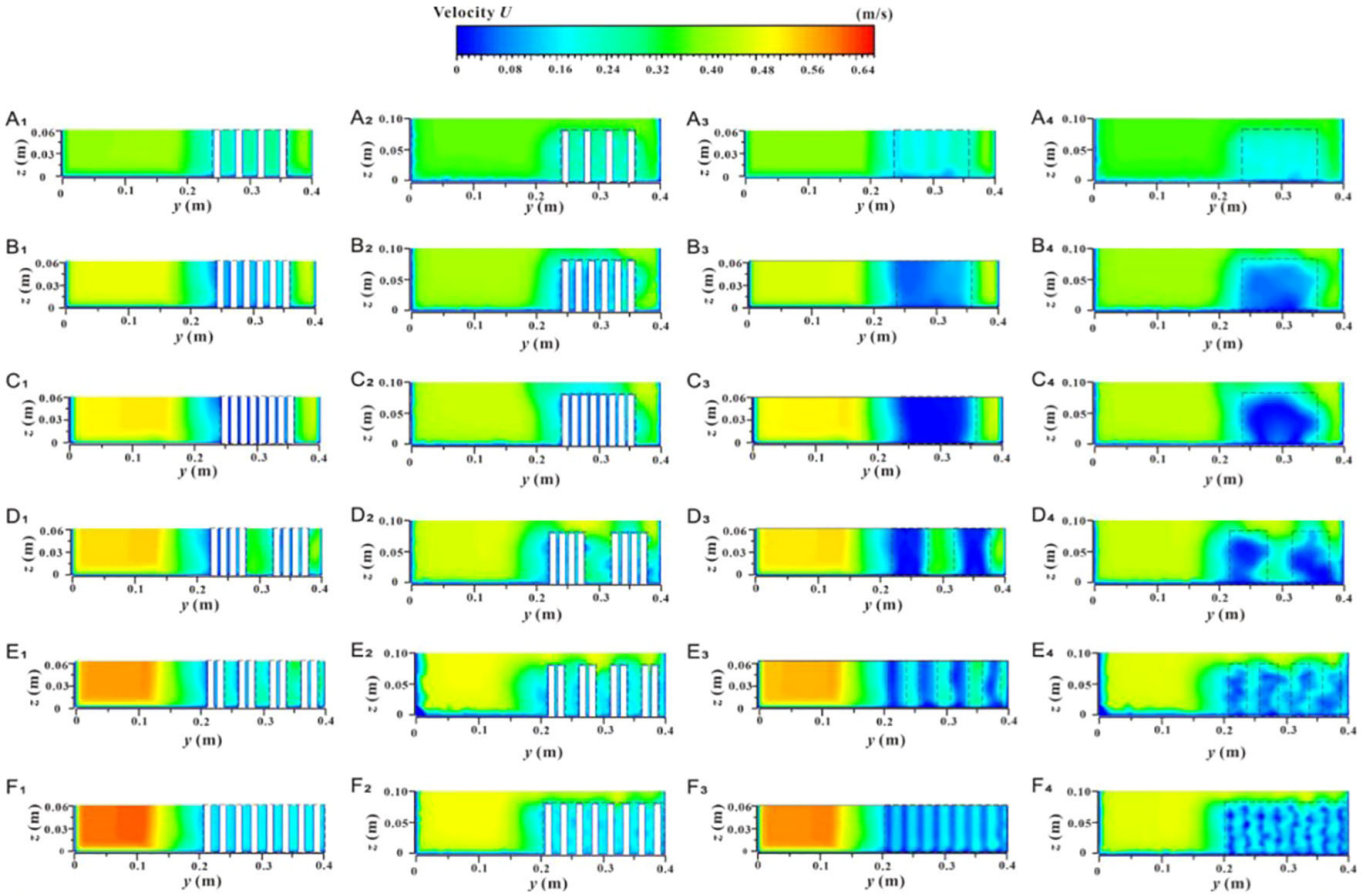
Spatial distribution of velocity contours of *yz* section under different submerged conditions, velocity profile at the C_1-1_ position in non-submerged state **(A_1_–F_1_)**, velocity profile at the C_1-1_ positon in submerged state **(A_2_–F_2_)**, velocity profile at the C_2-2_ position in non-submerged state **(A_3_–F_3_)**, and velocity profile at the C_2-2_ position in submerged state **(A_4_–F_4_)**.

**Figure 8** shows the spatial distribution of velocity contours for the *xy* profile under two submerged conditions, which helps to understand the overall change in river flow velocity due to vegetation patches and their fragmentation characteristics. In the non-submerged state, the *U* value in the non-vegetated area is significantly greater than that in the vegetated area, and this difference gradually increases with the increase in unit patch coverage (**Figures 8A_1_–C_1_**) and fragmentation degree (**Figures 8C_1_–F_1_**). However, in the submerged state (**Figures 8A_2_–F_2_**), the difference in *U* values between the vegetated and non-vegetated areas is relatively reduced, indicating that energy exchange between the two areas has occurred to some extent. Nonetheless, the *U* value in the non-vegetated area still follows the trend of gradually increasing with the increase in vegetation patch coverage (**Figures 8A_2_–C_2_**) and fragmentation degree (**Figures 8C_2_–F_2_**). This is because increased unit vegetation patch coverage under a specific distribution pattern enhances the water-blocking effect of the vegetation area while weakening flowability. Conversely, increased patch fragmentation alters the vegetation distribution pattern, improves the uniform distribution of vegetation, and refines the water grid within the vegetation area, which facilitates a more even allocation of overall water flow. However, it also disrupts runoff connectivity, increases the tortuosity of runoff paths, and makes reaching the target area more challenging. Therefore, whether due to increased vegetation patch coverage or increased fragmentation, the obstruction effect in the vegetation area intensifies, resulting in enhanced flow capacity in the non-vegetated area. **Figure 9** shows the spatial distribution of velocity contours for the *yz* profile (C_1-1_) along the inner patch and the *yz* profile (C_2-2_) along the gap between patches under different coverage and fragmentation conditions of vegetation patches in both submerged states, which also reflects these changes.

**Figure 10:**
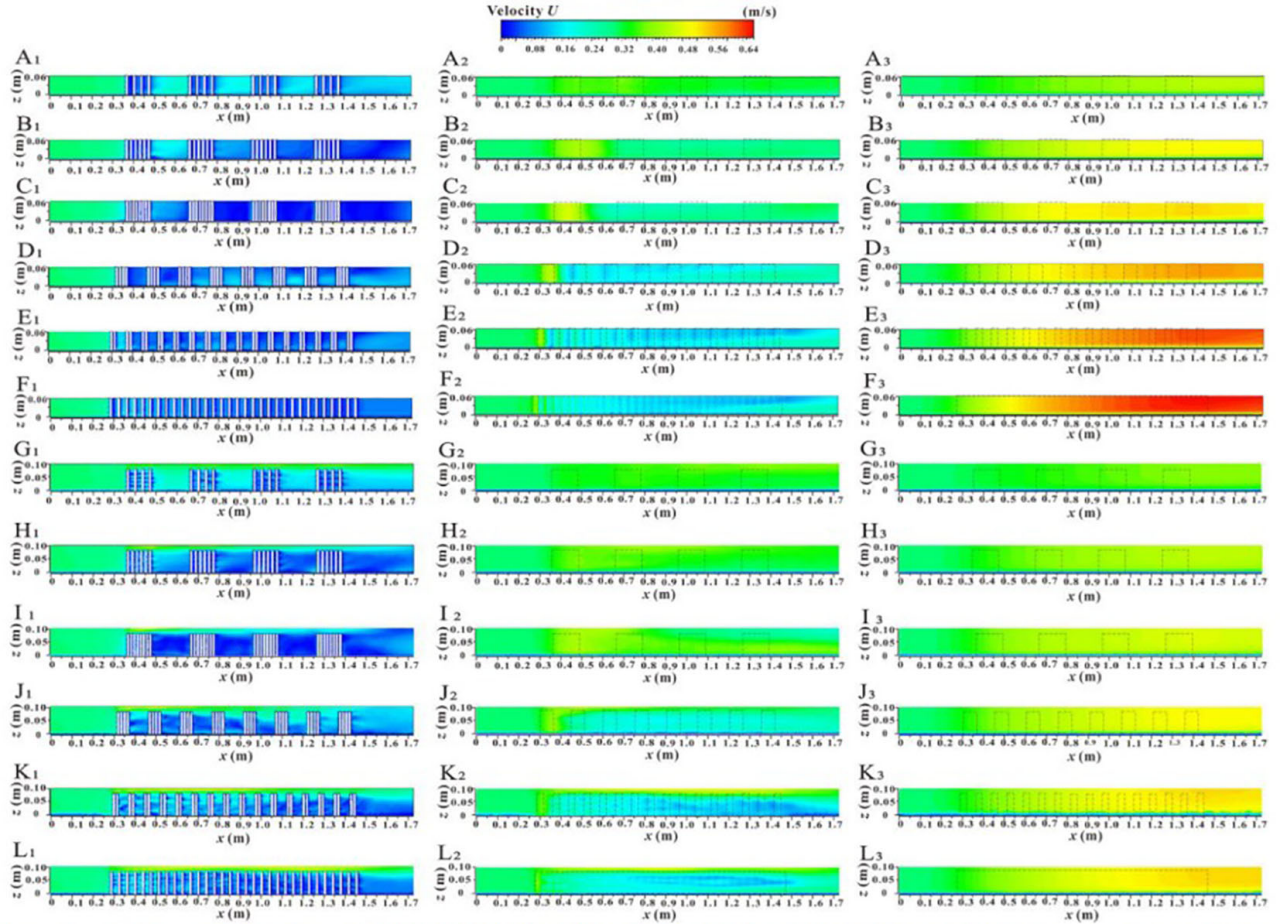
Spatial distribution of velocity contours of *xz* profile under different submerged conditions, velocity profile at the L_1-1_ position in non-submerged state **(A1–F1)**, velocity profile at the L_1-1_ position in submerged state **(G_1_–L_1_)**, velocity profile at the L_2-2_ position in non-submerged state **(A_2_–F_2_)**, velocity profile at the L_2-2_ position in submerged state **(G_2_-L_2_)**, velocity profile at the L_4-4_ position in non-submerged state **(A_3_-F_3_)**, velocity profile at the L_4-4_ position in submerged state **(G_3_-L_3_)**.

**Figure 10** shows the spatial distribution of the velocity contour for the *xz* section under two submerged conditions. This provides an intuitive understanding of the overall velocity distribution along the longitudinal section of the vegetation array, the adjacent plant channels, and the non-vegetated channel, based on patch coverage and fragmentation degree. In the non-submerged state, when the flow passes through the vegetation array (L_1-1_), the *U* value is low, but the *U* value inside the patch is smaller than that in the rear patch gap area due to the wake effect directly behind the vegetation. When the flow passes through the vegetation channel (L_2-2_), the *U* value inside the patch is higher than that in the rear gap area due to the influence of vegetation squeezing. Additionally, the *U* value in the vegetation area decreases with increasing distance along the path, while the non-vegetated area (L_4-4_) shows an increasing trend with distance. In the submerged state, the *U* values of the vegetation layer and the free layer are significantly different, indicating a strong mixing layer near the top of the canopy with substantial energy exchange.

Under both submerged states, the *U* values in and between patches of the vegetation array (L_1-1_) decrease with increasing unit patch coverage and increase with increasing patch fragmentation. When the flow passes through the vegetation channel (L_2-2_), the effects of patch coverage and fragmentation on *U* in and between patches are similar to those observed in the vegetation array (L_1-1_). In contrast, the *U* value of the longitudinal profile in the non-vegetated area (L_4-4_) increases with both patch coverage and fragmentation degree. The reasons for these observations have been explained above and will not be elaborated further.

### 3.3 Turbulence characteristics

#### 3.3.1 Vertical distribution of Reynolds stress at a specific location

Reynolds stress (*Rs*) refers to the shear stress generated by the exchange of turbulent water masses between flow layers, reflecting the inhomogeneity of flow velocity within the flow field. A higher *Rs* value indicates a more uneven spatial distribution of flow velocity and more intense turbulence in the area. It is a key parameter for studying the hydraulic characteristics of vegetation-type rivers (Li et al., 2020; Caroppi et al., 2022).

**Figure 11:**
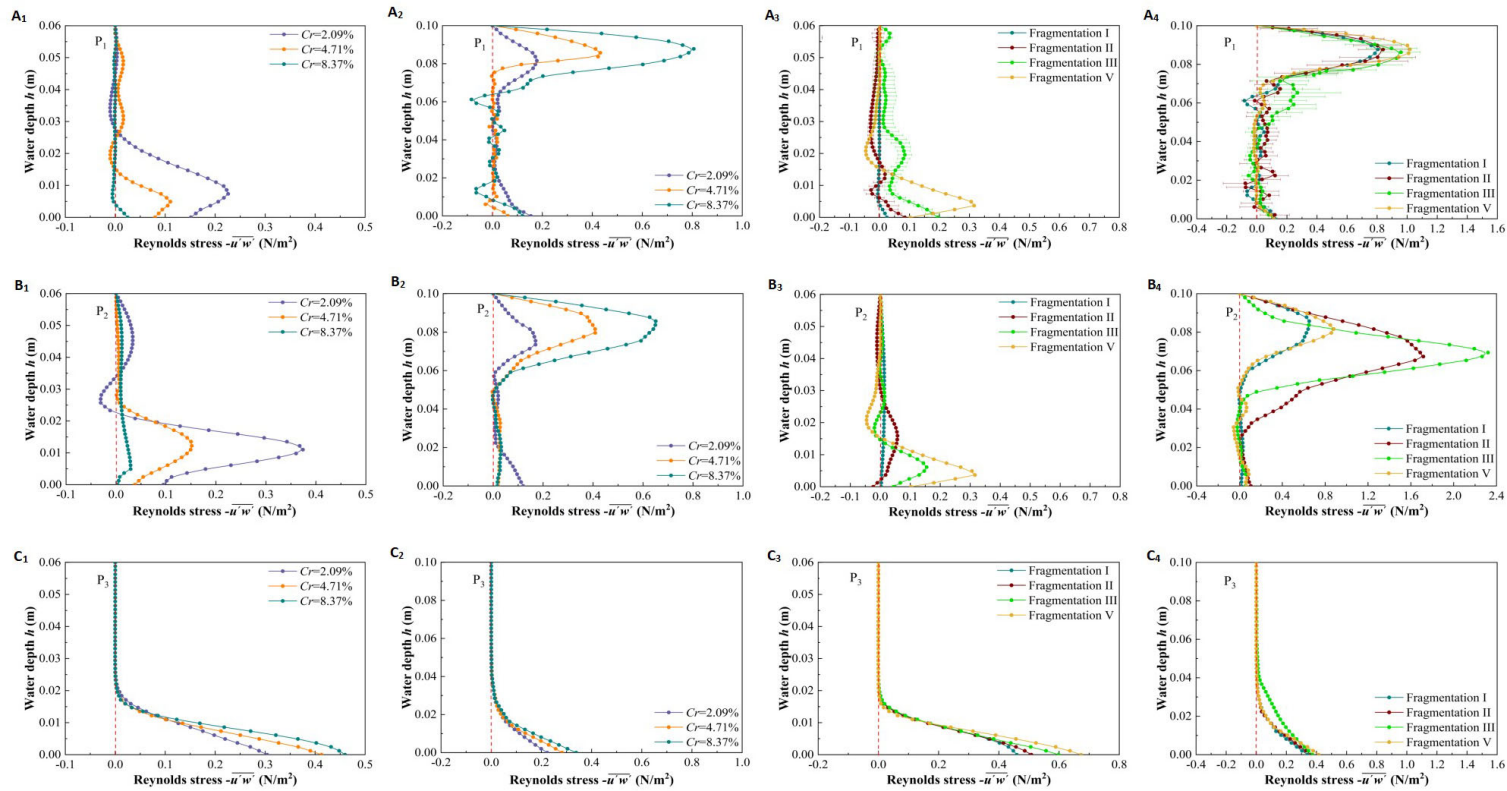
The vertical distribution of Reynolds stress of vegetationpatches with different coverage and fragmentation degree at specific locations (P_1_,P_2_,P_3_) in two submerged states, different coverage in non-submerged state **(A_1_–C_1_)**, different coverage in submerged state **(A_2_–C_2_)**, different fragmentation degree in non-submerged state **(A_3_–C_3_)**, different fragmentation degree in submerged state **(A_4_–C_4_)**.

**Figures 11A_1_–C_1_** and **A_2_–C_2_** show the vertical distribution of *Rs* at different patch coverage levels and specific locations (P_1_, P_2_, P_3_) under two submerged states. In the non-submerged state (**Figures 11A_1_–C_1_**), when *h* > 0.02* m* (*h*/*h_v_* > 1/4), patch coverage, water depth, and location have little effect on *Rs*, which remains close to zero. When *h*/*h_v_* < 1/4, due to the resistance shear force generated at the riverbed, *Rs* varies with patch coverage, water depth, and location. For the vegetated area, the *Rs* variation at P_1_ (inside the patch) and P_2_ (between patches) with water depth is similar (**Figures 11A_1_, B_1_**). Specifically, when *h*/*h_v_* < 1/4, *Rs* increases initially and then decreases with increasing water depth. At *h*/*h_v_* ≈ 1/8, *Rs* reaches an extreme point, and the maximum value decreases with increasing patch coverage. Notably, *Rs* at P_2_ is generally greater than that at P_1_. Additionally, at P_3_ (in the non-vegetated area), *Rs* decreases with increasing water depth, with its maximum value located at the riverbed. When *h*/*h_v_* < 1/4, *Rs* increases as patch coverage increases (**Figure 11C_1_**).

In the submerged state (**Figures 11A_2_–C_2_**), the variation of *Rs* at the P_1_ and P_2_ positions within the vegetation area with water depth is similar (**Figures 11A_2_, B_2_**). Specifically, due to the transition of water flow from the vegetation layer to the free layer, there is significant shear between the top of the vegetation canopy and the water body, resulting in the maximum *Rs* value near the top of the canopy (*h*/*h_v_* ≈ 1), with *Rs* increasing as coverage increases. Additionally, the variation of *Rs* with water depth at the P_3_ position in the non-vegetated area under submerged conditions is similar to that in the non-submerged state, but *Rs* near the bottom of the riverbed is smaller in the submerged state (**Figure 11C_2_**), indicating that the submerged state enhances sedimentation at the bottom of the riverbed compared to the non-submerged state.

**Figures 11A_3_–C_3_, A_4_–C_4_** show the vertical distribution of *Rs* with different fragmentation degrees at specific locations (P_1_, P_2_, P_3_) under two submerged states. Under the same submerged conditions, the overall pattern of vertical *Rs* distribution with water depth is similar to the pattern observed with different vegetation patch coverages. In the non-submerged state, there is a strong stress effect at the bottom of the riverbed in the vegetation area (**Figures 11A_3_, B_3_**), whereas, in the submerged state, the strong stress effect is near the top of the vegetation canopy (**Figures 11A_4_, B_4_**). It is important to note that the effect of patch fragmentation on *Rs* at these two locations is opposite to that of patch coverage: *Rs* is positively correlated with patch fragmentation, indicating that fragmentation is a key factor affecting *Rs*. Furthermore, in both submerged states, the degree of patch fragmentation has little effect on *Rs* in the non-vegetated channel area (**Figures 11C_3_, C_4_**).

#### 3.3.2 Longitudinal distribution of turbulent kinetic energy at specific locations

**Figure 12:**
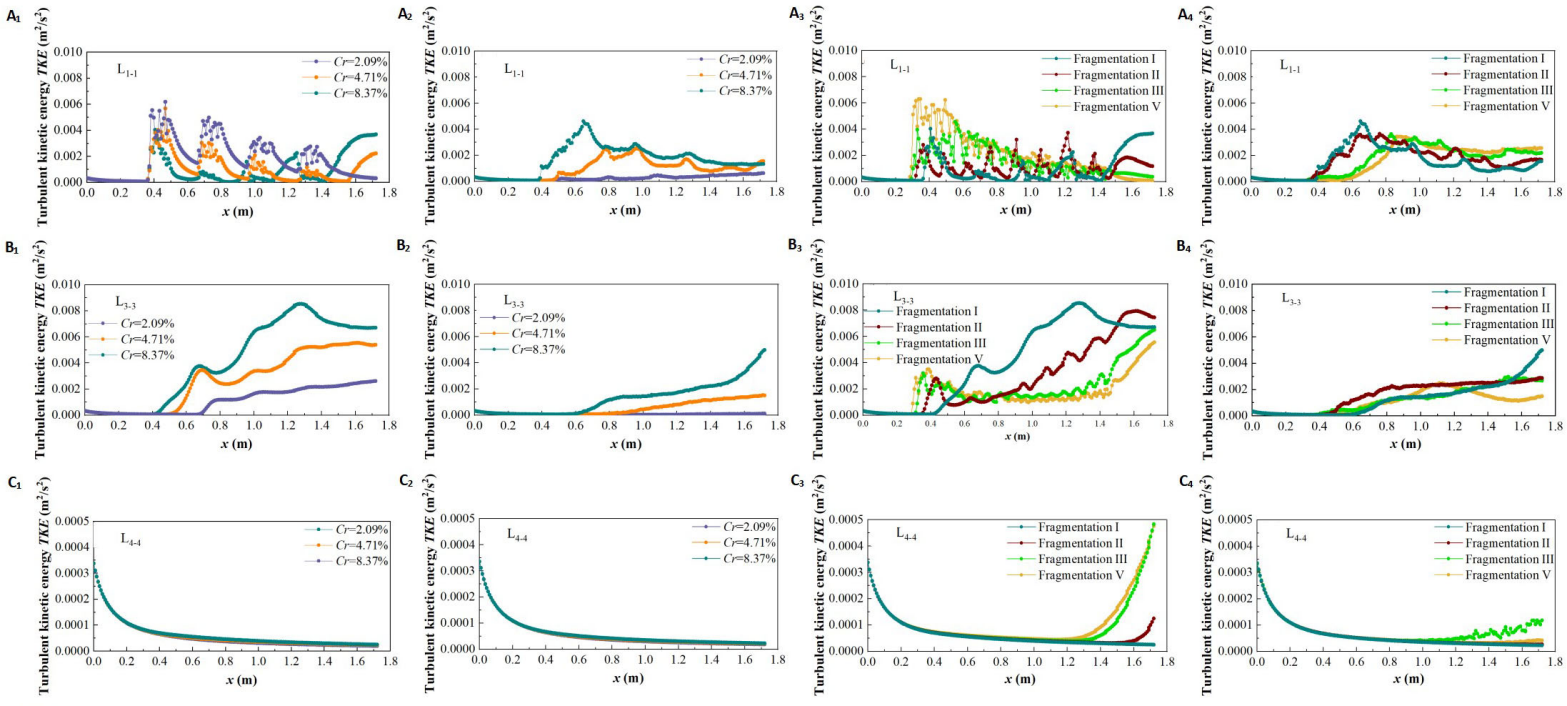
The turbulent kinetic energy distribution of different coverage and fragmentation degree of vegetation patches at specific positions in two submerged states, different coverage in non-submerged state **(A_1_–C_1_)**, different coverage in submerged state **(A_2_–C_2_)**, different fragmentation degree in non-submerged state **(A_3_–C_3_)**, different fragmentation degree in submerged state **(A_4_–C_4_)**.

Turbulent kinetic energy (*TKE*) is a key indicator of turbulence intensity, defined as the ratio of the root mean square of the fluctuating velocity to the corresponding time-averaged velocity. To clarify the influence of vegetation patch coverage and fragmentation characteristics on the turbulent structure of water flow, **Figure 12** illustrates the longitudinal distribution of *TKE* in specific vertical sections (L_1-1_, L_3-3_, L_4-4_) under two submerged conditions (*h* = 0.05 m and *h* = 0.09 m).

**Figure 13:**
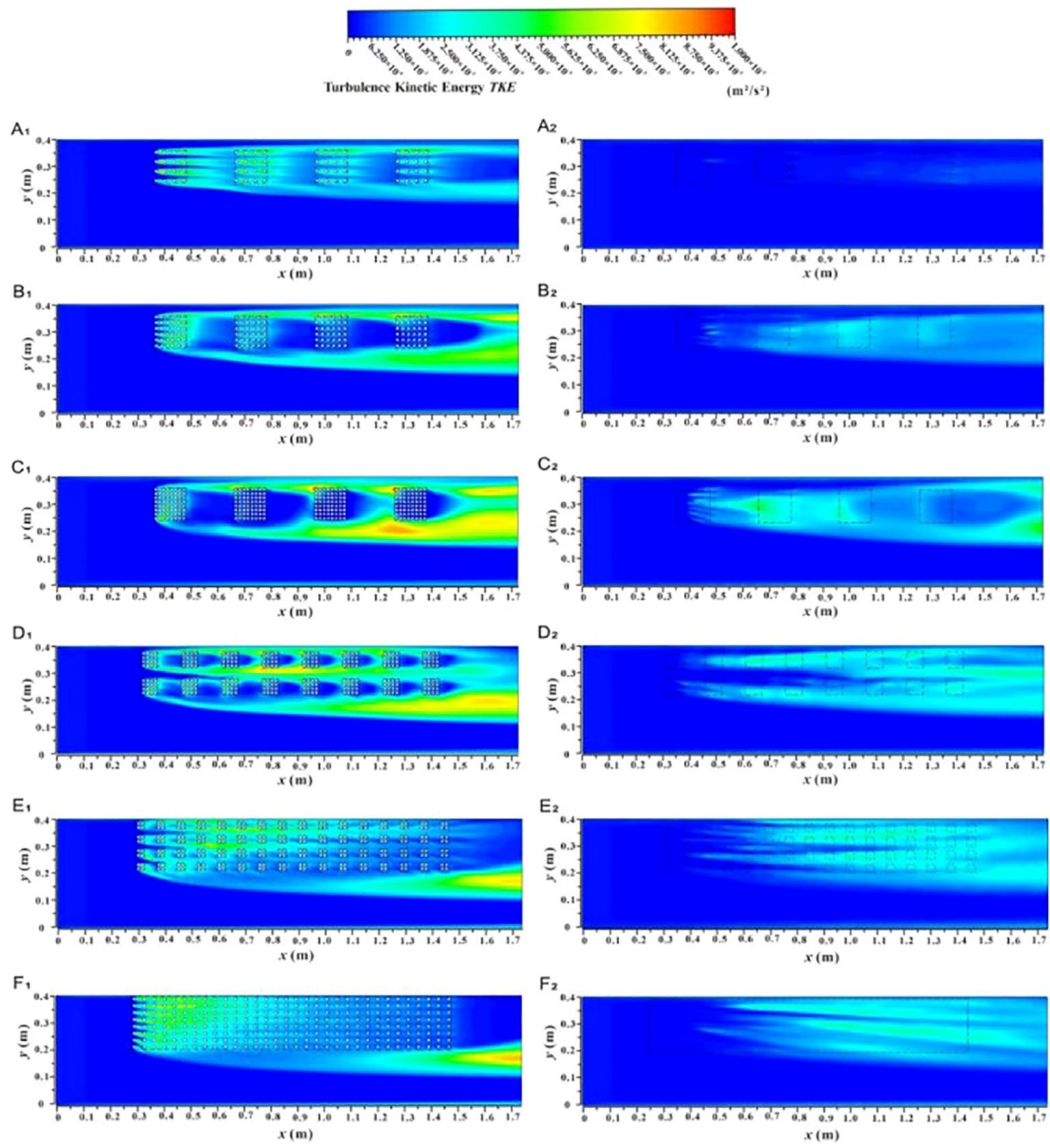
The spatial distributom of the turbulent kinetic energy contour of the *xy* profile under different submerged states, non-submerged state **(A_1_–F_1_)**, submerged **(A_2_–F_2_)**.

**Figures 12A_1_–C_1_, A_2_–C_2_** show the distribution of *TKE* under different vegetation patch coverages in specific profiles for the two inundation states. In the non-submerged state, when the flow is through the vegetation area (L_1-1_) (**Figure 12A_1_**), the *TKE* inside the patch is serrated and significantly larger than in the patch gap area due to the flow resistance of the vegetation patch. This creates a high-low alternating turbulent zone along the longitudinal section, which shows an overall downward trend. Additionally, *TKE* decreases with increasing patch coverage. This is because, as patch coverage increases, flow resistance rises, causing a reduction in flow velocity inside the patch and a corresponding weakening of turbulence intensity within the vegetation area (**Figures 13A_1_–C_1_**).

When the flow is through the boundary area between vegetation and non-vegetation (L_3-3_) (**Figure 12B_1_**), *TKE* shows an overall increasing trend along the profile. With increased patch coverage, the boundary area generates strong lateral radiation flow, leading to higher *TKE*. Conversely, when the flow is through the non-vegetated area (L_4-4_) (**Figure 12C_1_**), the *TKE* value is generally low and almost unaffected by changes in coverage and length along the path. This is because L_4-4_ is distant from the vegetated area, and the flow structure is less influenced by the patches.

Compared with the non-submerged state, when water flows through the vegetation area (L_1-1_) under the submerged state (**Figure 12A_2_**), the trend of *TKE* values in the free layer concerning patch coverage is opposite to that in the vegetation layer. Specifically, *TKE* increases with the increase in patch coverage in the free layer, while it decreases in the vegetation layer. This occurs because an increase in patch coverage within the vegetation layer raises the difficulty of water flow, causing the flow to release significant turbulence into the free layer, thus increasing the turbulence intensity of the free layer flow (**Figures 13A_2_–C_2_**). Additionally, the *TKE* in the free layer of the vegetation and non-vegetation boundary area (L_3-3_) in the submerged state shows an overall increasing trend with the path length and increases with patch coverage (**Figure 12B_2_**). Conversely, the *TKE* value in the free layer of the non-vegetation area (L_4-4_) is almost unaffected by changes in path length and coverage (**Figure 12C_2_**). It is worth noting that the *TKE* value in the free layer under submerged conditions decreases compared to the non-submerged vegetation layer, indicating that the influence of the vegetation distribution pattern on free layer flow turbulence is reduced under submerged conditions.

To explore the influence of patch fragmentation degree on the turbulence characteristics of the flow field under the same vegetation coverage conditions, this study examined the *TKE* distribution in specific vertical sections with different fragmentation degrees of patches under two submerged conditions (**Figures 12A_3_–C_3_**, **12A_4_–C_4_**). The overall trend of *TKE* concerning path length in the same submerged state and longitudinal section under varying patch fragmentation is similar to that observed with different vegetation patch coverage.

The overall variation of *TKE* along the length is similar to that observed with vegetation patch coverage. Additionally, the degree of vegetation fragmentation has a significant effect on *TKE*. Under non-inundated conditions, the *TKE* in the vegetation area (L_1-1_) increases with increasing patch fragmentation (**Figure 12A_3_**). This is because increased patch fragmentation in the vegetation area promotes a more homogeneous distribution of vegetation, which also homogenizes the flow within the vegetation area. As a result, the turbulence intensity becomes more evenly distributed and improves to a certain extent (**Figures 13D1–F1**). In other words, the degree of patch fragmentation is a key factor influencing changes in turbulence intensity within the flow field under specific regional and vegetation coverage conditions.

*TKE* in the boundary area between vegetation and non-vegetation (L_3-3_) shows a decreasing trend with increasing patch fragmentation (**Figure 12B_3_**). This is due to the homogenization of patch distribution in the vegetation area, which reduces turbulence intensity in the boundary area between vegetation and non-vegetation. In contrast, *TKE* in non-vegetated areas (L_4-4_) is almost unaffected by the degree of patch fragmentation in vegetated areas (**Figure 12C_3_**).

Under submerged conditions (**Figures 12A_4_–C_4_**), the degree of vegetation patch fragmentation has little effect on the turbulent characteristics of free-layer flow. The impact of vegetation patch fragmentation on free-layer *TKE* is only observed in the upstream vegetation area (**Figures 11A_4_**, **13D_2_–F_2_**).

## 4 Discussion

According to the study of velocity distribution, the discontinuous distribution of vegetation patches disrupts the longitudinal continuity of flow velocity and compromises the uniformity of the longitudinal flow field, which is consistent with the findings of Zhang et al. (2022a). The specific arrangement of vegetation on one side of the river determines the difference in flow-carrying capacity between vegetated and non-vegetated areas. A larger velocity difference between these areas indicates a stronger energy exchange (Huai et al., 2019; Tang et al., 2021). The increase in flow velocity in non-vegetated areas inhibits sediment deposition and accelerates channel erosion, thus limiting the lateral expansion of vegetation patches (Balke et al., 2012; Li et al., 2020). In contrast, the decrease in flow velocity in the wake region behind plants and patches in vegetated areas promotes sediment deposition, fostering the growth of aquatic vegetation and creating a favorable environment for the survival and habitat of aquatic organisms (Shi et al., 2016). This process also explains the common distribution of vegetation along riverbanks. Folkard and Gascoigne (2009) and Kim et al. (2018) similarly proposed that low-velocity zones in ecological river channels are beneficial for aquatic organisms. Thus, considering the attributes of vegetation patches is critical for guiding decision-making and management of protected species in fragmented watershed environments (Mazerolle and Villard, 1999; Banks et al., 2005; Holland and Bennett, 2009).

Furthermore, altering the coverage and fragmentation of vegetation patches plays an important role in regulating the flow-carrying capacity of rivers. An increase in vegetation patch coverage significantly reduces flow within vegetated areas, but markedly enhances the flow-carrying capacity of non-vegetated areas. Increased patch fragmentation promotes the homogenization of flow velocity within vegetated areas, while also improving the flow-carrying capacity of non-vegetated regions. Studying the overall changes in flow field velocity between vegetated and non-vegetated areas under different vegetation patch distribution patterns provides a scientific basis for understanding the mechanisms of riverbank sediment erosion, deposition, and the growth and evolution of vegetation communities. These insights can be applied to the planning and restoration of actual river wetlands.

According to the study on turbulent characteristics, the vertical Reynolds stress exhibits a strong effect at the bottom of the river channel in the non-submerged state, which aligns with the findings of Zhang et al. (2022a). In contrast, there is a strong stress effect near the top of the vegetation canopy in the submerged state, consistent with the conclusions of Anjum et al. (2018). The stress intensity at the bottom of the vegetated area is significantly lower than that in the non-vegetated area, indicating that aquatic vegetation plays a role in reducing riverbed erosion and deposition, thereby increasing riverbed stability (Zhu and Chen, 2019; Zaha et al., 2019). Additionally, the stress effect at the bottom of the river in the submerged state is smaller than in the non-submerged state, suggesting that the submerged state is also a critical factor influencing riverbed stability.

Furthermore, changes in vegetation patch coverage and fragmentation on one side of the distributed channel have significant effects on overall channel flow turbulence. In the non-submerged state, an increase in patch coverage within the vegetated area leads to a reduction in flow stress within the vegetation layer, which facilitates sediment deposition. In the submerged state, however, an increase in patch coverage enhances the flow stress near the top of the vegetation canopy. As patch coverage increases in the vegetated area, the flow stress at the bottom of the non-vegetated area on the opposite side of the river channel also increases, promoting sediment erosion and deposition in the non-vegetated area.

In the non-submerged state, the degree of patch fragmentation within the vegetated area enhances the flow stress effect in the vegetation layer and also amplifies the flow stress at the bottom of the river in non-vegetated areas. In the submerged state, an increase in patch fragmentation strengthens the stress effect near the top of the vegetation canopy.

From the study of turbulence characteristics, it can be concluded that for longitudinal turbulent kinetic energy at typical positions, *TKE* in the non-submerged state exhibits an overall upward trend in the patch area, consistent with previous research on the behavior of *TKE* in discontinuous vegetation patches in rivers (Zhao and Huai, 2016; Ghani et al., 2019). The increase in vegetation patch coverage in the non-submerged state leads to a decrease in TKE within the vegetated area, which contradicts the findings of Anjum and Tanaka (2019) and Aina et al. (2021).

The reasons for this discrepancy are as follows: In terms of calculating vegetation coverage based on the outline of unit patches, the rigid vegetation patch coverage in Anjum and Tanaka (2019) study was 0.72–2.17%, whereas the vegetation patch coverage range in this study (9.50–37.99%) was significantly larger. In this study, the considerable increase in vegetation volume greatly reduced wave penetration in the patch, resulting in a substantial decrease in *TKE* within the vegetated area. Based on these different conclusions, we hypothesize that there may be a critical threshold for rigid vegetation patch coverage. When the coverage is below this threshold, vegetation coverage is positively correlated with *TKE*, and above the threshold, it is negatively correlated. This hypothesis requires further investigation in future work.

Regarding the findings of Aina et al. (2021), their study used polyethylene filaments with a diameter of 0.075 mm to simulate flexible vegetation (algae). Due to the significant difference in material flexibility, the water-blocking effect of the vegetation patch in their study was much lower than that of the rigid vegetation patches in this study. Consequently, the increase in vegetation coverage had little effect on wave penetration within the patch. Moreover, the swaying motion of flexible vegetation generates stronger vortex shedding, promoting an increase in wake turbulence, which contradicts the results of this study.

Additionally, an increase in vegetation patch coverage in the submerged state leads to an increase in *TKE* in the free layer of the vegetation area. Related research by Pujol et al. (2013) also shows that the attenuation of *TKE* depends on vegetation density, flexibility, and submergence level. Under the condition of constant vegetation patch coverage, the fragmentation of patches promotes the homogenization of *TKE* within the vegetation area and the surrounding flow field. In the non-submerged state, turbulence intensity in the upstream vegetation area gradually increases, whereas, in the submerged state, turbulence intensity in the upstream free layer of the vegetation area gradually weakens. Thus, the fragmentation of vegetation patches is also an important factor influencing *TKE* in the vegetated area, indicating the need for further study of vegetation patch fragmentation in river systems.

In general, the ecological planning and design of integrated wetland vegetation landscapes is of great significance. Through scientific, site-specific planning, the harmonious integration of river wetland ecological protection and plant landscape beautification can be achieved. This study comprehensively considers the influence of partial, discontinuous vegetation patch distribution on river flow structure under different coverage and fragmentation conditions, thereby enriching the theoretical framework of vegetated river channels. The rational and optimal allocation of vegetation patches within rivers has positive effects on aquatic habitats, riverbed stability, vegetation growth mechanisms, and habitat diversity (Huai et al., 2021; Qu et al., 2022; Federico and Lenore, 2023). Additionally, the appropriate allocation of patch coverage and fragmentation within vegetation areas can serve as a feasible approach for ecological restoration management and practice in riverbanks or floodplains.

## 5 Conclusion

A three-dimensional Reynolds stress model numerical simulation method was used to study an ecological river with three levels of vegetation patch coverage and four types of patch fragmentation. The changes in flow structure characteristics were examined. The main conclusions are as follows:

1. In terms of the overall change in streamwise velocity along the path, the distribution of partially discontinuous vegetation patches disrupts the longitudinal continuity of streamwise velocity and diminishes the homogenization of the longitudinal flow field. Under both submerged conditions, the streamwise velocity in the vegetated area gradually decreases along the path, while the opposite occurs in the non-vegetated area.
2. Regarding the influence of vegetation patch coverage and fragmentation on streamwise velocity, in the non-submerged state, patch coverage is negatively correlated with streamwise velocity in the vegetated area and positively correlated with streamwise velocity in the non-vegetated area. Patch fragmentation promotes the homogenization of streamwise velocity in the vegetated area, and streamwise velocity in the non-vegetated area is positively correlated with the degree of patch fragmentation. Under the same vegetation coverage, even when the row and column spacing of adjacent plants in the unit patch is constant, streamwise velocity in the vegetated area is positively correlated with patch fragmentation under both submerged conditions.
3. Regarding the vertical distribution of Raynaud stress, in the non-submerged state, when *h*/*h_v_* < 1/4, the characteristics of patch distribution and the degree of vegetation submergence have significant effects on Reynolds stress. When *h*/*h_v_* = 1/8, there is a strong stress effect in the vegetated area, with Reynolds stress negatively correlated with patch coverage and positively correlated with patch fragmentation. In the non-vegetated area, there is a strong stress effect at the bottom of the riverbed, where Reynolds stress is positively correlated with both patch coverage and fragmentation. In the submerged state, a strong stress effect is observed near the top of the vegetation canopy (*h*/*h_v_* ≈ 1), where Reynolds stress is positively correlated with patch coverage and fragmentation. At the bottom of the riverbed in the non-vegetated area, Reynolds stress is positively correlated with patch coverage, though the degree of fragmentation has little effect on Reynolds stress.
4. Concerning the longitudinal distribution of turbulent kinetic energy, in the non-submerged state, *TKE* in the patch region shows a zigzag distribution, with intensity significantly greater than in the gap region. However, this pattern weakens as the degree of patch fragmentation increases. Additionally, the longitudinal distribution of *TKE* in the vegetated area is negatively correlated with patch coverage and positively correlated with patch fragmentation. The longitudinal distribution of *TKE* in the non-vegetated area is minimally affected by changes in length, patch coverage, or degree of fragmentation. In the submerged state, the variation in turbulence intensity in the longitudinal distribution of the free layer within the vegetated area is opposite to that of the non-submerged vegetation layer, and the degree of patch fragmentation has little effect on the turbulence of the free layer flow.

In this study, the heterogeneity of river vegetation landscapes is considered, and the impact of changes in vegetation patch coverage on flow characteristics along the longitudinally discontinuous distribution of the riverbank is explored. It is demonstrated that, under specific conditions of vegetation area and coverage, altering the degree of vegetation patch fragmentation plays an important role in regulating river channel flow structure. Therefore, the strategic arrangement of vegetation patches can provide theoretical guidance for the ecological design and management of riverbank or floodplain wetlands.

It should be noted that this study is based on the premise of controlled variables, and thus its modeling process and numerical simulation assumptions are relatively simplified compared to real river channels, which are influenced by numerous interacting factors. For instance, the choice of cylindrical vegetation as a simplified representation of actual plant geometry may affect the generalizability of the results. Furthermore, this study focuses on selected representative elements of vegetation distribution characteristics, whereas the distribution patterns and growth states of vegetation in real river channels are more diverse and complex, meaning the conclusions may not fully capture the vegetation-flow dynamics of actual rivers. Additionally, vegetation flow is a multidisciplinary issue, and it is undeniable that the conclusions of this study have certain limitations, with many aspects requiring further investigation in the future.

## Data Availability

The raw data supporting the conclusions of this article will be made available by the authors, without undue reservation.
