# Supplementary material for: Numerical simulation of landscape ecological river flow structure based on vegetation patch distribution and fragmentation
Source: Front Plant Sci. 2024 Oct 10;15:1424566. doi: 10.3389/fpls.2024.1424566 (PMC11499190; doi:10.3389/fpls.2024.1424566)
Supplement: Supplementary file 1 [file Table1.docx]

**Table. 1** Simulated values of P_2_ under different water depth conditions under submerged state.

| Hydraulic parameters | Flow velocity U (m/s) | | | | | |
| --- | --- | --- | --- | --- | --- | --- |
| Water depth h (m) | *Cr*=2.09% | *Cr*=4.71% | Fragmentation I (Cr=8.37%) | Fragmentation II (Cr=8.37%) | Fragmentation III (Cr=8.37%) | Fragmentation V (Cr=8.37%) |
| 0.000 | 0.0000 | 0.0000 | 0.0000 | 0.0000 | 0.0000 | 0.0000 |
| 0.001 | 0.0163 | 0.0025 | 0.0084 | 0.0168 | 0.0163 | 0.0204 |
| 0.002 | 0.0327 | 0.0050 | 0.0167 | 0.0336 | 0.0327 | 0.0422 |
| 0.003 | 0.0489 | 0.0076 | 0.0237 | 0.0481 | 0.0489 | 0.0611 |
| 0.004 | 0.0646 | 0.0100 | 0.0282 | 0.0607 | 0.0646 | 0.0686 |
| 0.005 | 0.0803 | 0.0123 | 0.0328 | 0.0734 | 0.0803 | 0.0762 |
| 0.006 | 0.0960 | 0.0144 | 0.0392 | 0.0861 | 0.0960 | 0.0841 |
| 0.007 | 0.1120 | 0.0165 | 0.0485 | 0.0987 | 0.1120 | 0.0944 |
| 0.008 | 0.1270 | 0.0186 | 0.0487 | 0.1110 | 0.1270 | 0.1020 |
| 0.009 | 0.1340 | 0.0199 | 0.0480 | 0.1180 | 0.1340 | 0.1090 |
| 0.010 | 0.1380 | 0.0212 | 0.0472 | 0.1220 | 0.1380 | 0.1160 |
| 0.011 | 0.1420 | 0.0225 | 0.0463 | 0.1250 | 0.1420 | 0.1220 |
| 0.012 | 0.1460 | 0.0237 | 0.0455 | 0.1270 | 0.1460 | 0.1240 |
| 0.013 | 0.1500 | 0.0246 | 0.0447 | 0.1300 | 0.1500 | 0.1250 |
| 0.014 | 0.1550 | 0.0255 | 0.0438 | 0.1320 | 0.1550 | 0.1250 |
| 0.015 | 0.1590 | 0.0265 | 0.0430 | 0.1340 | 0.1590 | 0.1250 |
| 0.016 | 0.1620 | 0.0274 | 0.0422 | 0.1360 | 0.1620 | 0.1240 |
| 0.017 | 0.1640 | 0.0283 | 0.0413 | 0.1360 | 0.1640 | 0.1230 |
| 0.018 | 0.1650 | 0.0292 | 0.0404 | 0.1360 | 0.1650 | 0.1220 |
| 0.019 | 0.1660 | 0.0301 | 0.0391 | 0.1350 | 0.1660 | 0.1210 |
| 0.020 | 0.1670 | 0.0316 | 0.0376 | 0.1340 | 0.1670 | 0.1210 |
| 0.021 | 0.1680 | 0.0334 | 0.0361 | 0.1340 | 0.1680 | 0.1210 |
| 0.022 | 0.1690 | 0.0348 | 0.0346 | 0.1330 | 0.1690 | 0.1210 |
| 0.023 | 0.1700 | 0.0359 | 0.0331 | 0.1320 | 0.1700 | 0.1210 |
| 0.024 | 0.1710 | 0.0370 | 0.0316 | 0.1310 | 0.1710 | 0.1210 |
| 0.025 | 0.1720 | 0.0376 | 0.0301 | 0.1310 | 0.1720 | 0.1180 |
| 0.026 | 0.1730 | 0.0382 | 0.0287 | 0.1310 | 0.1730 | 0.1140 |
| 0.027 | 0.1730 | 0.0396 | 0.0272 | 0.1300 | 0.1730 | 0.1130 |
| 0.028 | 0.1730 | 0.0411 | 0.0260 | 0.1300 | 0.1730 | 0.1120 |
| 0.029 | 0.1730 | 0.0426 | 0.0248 | 0.1290 | 0.1730 | 0.1110 |
| 0.030 | 0.1730 | 0.0441 | 0.0236 | 0.1280 | 0.1730 | 0.1090 |
| 0.031 | 0.1730 | 0.0451 | 0.0225 | 0.1280 | 0.1730 | 0.1080 |
| 0.032 | 0.1720 | 0.0455 | 0.0213 | 0.1280 | 0.1720 | 0.1060 |
| 0.033 | 0.1720 | 0.0460 | 0.0202 | 0.1290 | 0.1720 | 0.1050 |
| 0.034 | 0.1710 | 0.0464 | 0.0190 | 0.1310 | 0.1710 | 0.1040 |
| 0.035 | 0.1710 | 0.0468 | 0.0179 | 0.1330 | 0.1710 | 0.1050 |
| 0.036 | 0.1700 | 0.0473 | 0.0168 | 0.1350 | 0.1700 | 0.1060 |
| 0.037 | 0.1700 | 0.0477 | 0.0155 | 0.1370 | 0.1700 | 0.1080 |
| 0.038 | 0.1690 | 0.0482 | 0.0140 | 0.1390 | 0.1690 | 0.1100 |
| 0.039 | 0.1690 | 0.0487 | 0.0126 | 0.1410 | 0.1690 | 0.1140 |
| 0.040 | 0.1680 | 0.0493 | 0.0113 | 0.1430 | 0.1680 | 0.1180 |
| 0.041 | 0.1680 | 0.0498 | 0.0101 | 0.1450 | 0.1680 | 0.1220 |
| 0.042 | 0.1670 | 0.0503 | 0.0090 | 0.1490 | 0.1670 | 0.1270 |
| 0.043 | 0.1670 | 0.0507 | 0.0081 | 0.1530 | 0.1670 | 0.1290 |
| 0.044 | 0.1660 | 0.0512 | 0.0074 | 0.1590 | 0.1660 | 0.1290 |
| 0.045 | 0.1660 | 0.0516 | 0.0069 | 0.1650 | 0.1660 | 0.1290 |
| 0.047 | 0.1670 | 0.0516 | 0.0069 | 0.1680 | 0.1670 | 0.1310 |
| 0.048 | 0.1680 | 0.0513 | 0.0073 | 0.1710 | 0.1680 | 0.1300 |
| 0.049 | 0.1690 | 0.0510 | 0.0081 | 0.1750 | 0.1690 | 0.1300 |
| 0.050 | 0.1690 | 0.0511 | 0.0082 | 0.1780 | 0.1690 | 0.1300 |
| 0.051 | 0.1700 | 0.0516 | 0.0087 | 0.1820 | 0.1700 | 0.1300 |
| 0.052 | 0.1710 | 0.0521 | 0.0095 | 0.1850 | 0.1710 | 0.1300 |
| 0.053 | 0.1710 | 0.0528 | 0.0105 | 0.1890 | 0.1710 | 0.1310 |
| 0.054 | 0.1720 | 0.0535 | 0.0117 | 0.1920 | 0.1720 | 0.1310 |
| 0.055 | 0.1720 | 0.0542 | 0.0129 | 0.1960 | 0.1720 | 0.1320 |
| 0.056 | 0.1730 | 0.0549 | 0.0142 | 0.1990 | 0.1730 | 0.1310 |
| 0.057 | 0.1730 | 0.0556 | 0.0156 | 0.2030 | 0.1730 | 0.1300 |
| 0.058 | 0.1740 | 0.0563 | 0.0170 | 0.2070 | 0.1740 | 0.1290 |
| 0.059 | 0.1740 | 0.0570 | 0.0184 | 0.2120 | 0.1740 | 0.1280 |
| 0.060 | 0.1750 | 0.0578 | 0.0199 | 0.2180 | 0.1750 | 0.1280 |
| 0.061 | 0.1770 | 0.0613 | 0.0213 | 0.2240 | 0.1770 | 0.1270 |
| 0.062 | 0.1790 | 0.0652 | 0.0235 | 0.2310 | 0.1790 | 0.1250 |
| 0.063 | 0.1830 | 0.0691 | 0.0259 | 0.2370 | 0.1830 | 0.1250 |
| 0.064 | 0.1870 | 0.0731 | 0.0284 | 0.2430 | 0.1870 | 0.1260 |
| 0.065 | 0.1910 | 0.0770 | 0.0309 | 0.2500 | 0.1910 | 0.1270 |
| 0.066 | 0.1950 | 0.0809 | 0.0334 | 0.2560 | 0.1950 | 0.1280 |
| 0.067 | 0.1990 | 0.0848 | 0.0359 | 0.2620 | 0.1990 | 0.1320 |
| 0.068 | 0.2040 | 0.0885 | 0.0384 | 0.2680 | 0.2040 | 0.1370 |
| 0.069 | 0.2080 | 0.0937 | 0.0410 | 0.2740 | 0.2080 | 0.1420 |
| 0.070 | 0.2120 | 0.0994 | 0.0436 | 0.2800 | 0.2120 | 0.1470 |
| 0.071 | 0.2160 | 0.1050 | 0.0466 | 0.2870 | 0.2160 | 0.1560 |
| 0.072 | 0.2210 | 0.1110 | 0.0514 | 0.2950 | 0.2210 | 0.1640 |
| 0.073 | 0.2260 | 0.1170 | 0.0567 | 0.3020 | 0.2260 | 0.1740 |
| 0.074 | 0.2310 | 0.1220 | 0.0619 | 0.3090 | 0.2310 | 0.1870 |
| 0.075 | 0.2370 | 0.1280 | 0.0671 | 0.3170 | 0.2370 | 0.2030 |
| 0.076 | 0.2430 | 0.1350 | 0.0723 | 0.3240 | 0.2430 | 0.2170 |
| 0.077 | 0.2500 | 0.1430 | 0.0775 | 0.3310 | 0.2500 | 0.2310 |
| 0.078 | 0.2560 | 0.1500 | 0.0827 | 0.3360 | 0.2560 | 0.2450 |
| 0.079 | 0.2630 | 0.1570 | 0.0879 | 0.3410 | 0.2630 | 0.2580 |
| 0.080 | 0.2690 | 0.1650 | 0.0933 | 0.3450 | 0.2690 | 0.2700 |
| 0.081 | 0.2760 | 0.1720 | 0.0996 | 0.3500 | 0.2760 | 0.2810 |
| 0.082 | 0.2820 | 0.1790 | 0.1060 | 0.3550 | 0.2820 | 0.2920 |
| 0.083 | 0.2880 | 0.1870 | 0.1120 | 0.3590 | 0.2880 | 0.3040 |
| 0.084 | 0.2940 | 0.1950 | 0.1180 | 0.3630 | 0.2940 | 0.3140 |
| 0.085 | 0.2990 | 0.2030 | 0.1240 | 0.3670 | 0.2990 | 0.3220 |
| 0.086 | 0.3050 | 0.2100 | 0.1300 | 0.3720 | 0.3050 | 0.3320 |
| 0.087 | 0.3100 | 0.2180 | 0.1370 | 0.3760 | 0.3100 | 0.3420 |
| 0.088 | 0.3150 | 0.2240 | 0.1430 | 0.3800 | 0.3150 | 0.3510 |
| 0.089 | 0.3210 | 0.2300 | 0.1490 | 0.3850 | 0.3210 | 0.3600 |
| 0.090 | 0.3260 | 0.2350 | 0.1540 | 0.3890 | 0.3260 | 0.3670 |
| 0.091 | 0.3290 | 0.2400 | 0.1570 | 0.3930 | 0.3290 | 0.3730 |
| 0.092 | 0.3320 | 0.2450 | 0.1600 | 0.3950 | 0.3320 | 0.3800 |
| 0.093 | 0.3350 | 0.2500 | 0.1620 | 0.3970 | 0.3350 | 0.3860 |
| 0.094 | 0.3390 | 0.2540 | 0.1650 | 0.3980 | 0.3390 | 0.3940 |
| 0.095 | 0.3410 | 0.2590 | 0.1680 | 0.4000 | 0.3410 | 0.3980 |
| 0.096 | 0.3440 | 0.2610 | 0.1710 | 0.4020 | 0.3440 | 0.4020 |
| 0.097 | 0.3470 | 0.2630 | 0.1740 | 0.4040 | 0.3470 | 0.4050 |
| 0.098 | 0.3500 | 0.2650 | 0.1770 | 0.4060 | 0.3500 | 0.4080 |
| 0.099 | 0.3530 | 0.2670 | 0.1800 | 0.4080 | 0.3530 | 0.4110 |
| 0.100 | 0.3560 | 0.2700 | 0.1830 | 0.4090 | 0.3560 | 0.4130 |

**Table. 2** Measured values of P_2_ under different water depth conditions under submerged state.

| **Hydraulic parameters** | **Flow velocity *U* (m/s)** | | | | | |
| --- | --- | --- | --- | --- | --- | --- |
| **Water depth *h* (m)** | ***Cr*=2.09%** | ***Cr*=4.71%** | **Fragmentation I (*Cr*=8.37% )** | **Fragmentation II (*Cr*=8.37% )** | **Fragmentation III (*Cr*=8.37% )** | **Fragmentation IV (*Cr*=8.37% )** |
| 0.025 | 0.165 | 0.039 | 0.033 | 0.142 | 0.166 | 0.122 |
| 0.030 | 0.167 | 0.045 | 0.020 | 0.131 | 0.164 | 0.125 |
| 0.035 | 0.165 | 0.045 | 0.014 | 0.145 | 0.175 | 0.117 |
| 0.041 | 0.155 | 0.048 | 0.011 | 0.142 | 0.162 | 0.129 |
| 0.047 | 0.158 | 0.051 | 0.008 | 0.168 | 0.161 | 0.116 |
| 0.054 | 0.161 | 0.050 | 0.014 | 0.181 | 0.164 | 0.125 |
| 0.058 | 0.153 | 0.050 | 0.011 | 0.199 | 0.161 | 0.136 |
| 0.063 | 0.164 | 0.055 | 0.027 | 0.210 | 0.177 | 0.137 |
| 0.067 | 0.185 | 0.071 | 0.035 | 0.235 | 0.188 | 0.133 |
| 0.071 | 0.202 | 0.092 | 0.052 | 0.256 | 0.205 | 0.164 |
| 0.076 | 0.226 | 0.115 | 0.070 | 0.289 | 0.225 | 0.185 |
| 0.082 | 0.261 | 0.155 | 0.123 | 0.320 | 0.258 | 0.239 |
| 0.087 | 0.289 | 0.189 | 0.149 | 0.368 | 0.289 | 0.320 |
| 0.092 | 0.309 | 0.219 | 0.178 | 0.399 | 0.312 | 0.399 |
| 0.097 | 0.333 | 0.245 | 0.195 | 0.408 | 0.342 | 0.432 |

**Table. 3** In the non-submerged state (*h*=0.05 m), the streamwise velocity *u* of the three vegetation patch coverage conditions changes along the *x* direction.

| **Hydraulic parameters** | **Streamwise velocity *u* (m/s)** | | | | | | | | | | | |
| --- | --- | --- | --- | --- | --- | --- | --- | --- | --- | --- | --- | --- |
| ***x* (m)** | **L_1-1_** | | | **L_2-2_** | | | **L_3-3_** | | | **L_4-4_** | | |
|  | ***Cr*=2.09%** | ***Cr*=4.71%** | ***Cr*=8.37%** | ***Cr*=2.09%** | ***Cr*=4.71%** | ***Cr*=8.37%** | ***Cr*=2.09%** | ***Cr*=4.71%** | ***Cr*=8.37%** | ***Cr*=2.09%** | ***Cr*=4.71%** | ***Cr*=8.37%** |
| 0.000 | 0.300 | 0.300 | 0.300 | 0.300 | 0.300 | 0.300 | 0.300 | 0.300 | 0.300 | 0.300 | 0.300 | 0.300 |
| 0.009 | 0.300 | 0.300 | 0.300 | 0.300 | 0.300 | 0.300 | 0.300 | 0.300 | 0.300 | 0.300 | 0.300 | 0.300 |
| 0.017 | 0.300 | 0.300 | 0.300 | 0.300 | 0.300 | 0.300 | 0.300 | 0.300 | 0.300 | 0.300 | 0.300 | 0.300 |
| 0.026 | 0.300 | 0.300 | 0.300 | 0.300 | 0.300 | 0.300 | 0.300 | 0.300 | 0.300 | 0.300 | 0.300 | 0.301 |
| 0.035 | 0.300 | 0.300 | 0.300 | 0.300 | 0.300 | 0.300 | 0.300 | 0.300 | 0.301 | 0.301 | 0.301 | 0.301 |
| 0.043 | 0.301 | 0.300 | 0.300 | 0.301 | 0.300 | 0.300 | 0.301 | 0.301 | 0.301 | 0.301 | 0.301 | 0.301 |
| 0.052 | 0.301 | 0.300 | 0.300 | 0.301 | 0.300 | 0.300 | 0.301 | 0.301 | 0.301 | 0.301 | 0.301 | 0.302 |
| 0.061 | 0.301 | 0.301 | 0.300 | 0.301 | 0.301 | 0.300 | 0.301 | 0.301 | 0.301 | 0.302 | 0.302 | 0.302 |
| 0.069 | 0.301 | 0.301 | 0.300 | 0.301 | 0.301 | 0.300 | 0.301 | 0.301 | 0.301 | 0.302 | 0.302 | 0.303 |
| 0.078 | 0.301 | 0.301 | 0.300 | 0.301 | 0.301 | 0.300 | 0.302 | 0.302 | 0.302 | 0.302 | 0.303 | 0.304 |
| 0.086 | 0.301 | 0.301 | 0.300 | 0.301 | 0.301 | 0.300 | 0.302 | 0.302 | 0.302 | 0.303 | 0.303 | 0.304 |
| 0.095 | 0.301 | 0.301 | 0.299 | 0.301 | 0.301 | 0.299 | 0.302 | 0.302 | 0.302 | 0.303 | 0.304 | 0.305 |
| 0.104 | 0.301 | 0.301 | 0.299 | 0.301 | 0.301 | 0.299 | 0.302 | 0.302 | 0.302 | 0.304 | 0.304 | 0.306 |
| 0.112 | 0.302 | 0.300 | 0.299 | 0.302 | 0.301 | 0.299 | 0.303 | 0.303 | 0.303 | 0.304 | 0.305 | 0.307 |
| 0.121 | 0.302 | 0.300 | 0.298 | 0.302 | 0.300 | 0.298 | 0.303 | 0.303 | 0.303 | 0.305 | 0.306 | 0.308 |
| 0.130 | 0.302 | 0.300 | 0.298 | 0.302 | 0.300 | 0.298 | 0.303 | 0.303 | 0.303 | 0.305 | 0.306 | 0.309 |
| 0.138 | 0.302 | 0.300 | 0.297 | 0.302 | 0.300 | 0.297 | 0.303 | 0.303 | 0.304 | 0.306 | 0.307 | 0.310 |
| 0.147 | 0.302 | 0.300 | 0.296 | 0.302 | 0.300 | 0.297 | 0.304 | 0.304 | 0.304 | 0.306 | 0.308 | 0.311 |
| 0.156 | 0.301 | 0.299 | 0.296 | 0.302 | 0.299 | 0.296 | 0.304 | 0.304 | 0.304 | 0.307 | 0.309 | 0.312 |
| 0.164 | 0.301 | 0.299 | 0.295 | 0.302 | 0.299 | 0.295 | 0.304 | 0.304 | 0.304 | 0.307 | 0.310 | 0.313 |
| 0.173 | 0.301 | 0.298 | 0.294 | 0.301 | 0.299 | 0.294 | 0.305 | 0.304 | 0.305 | 0.308 | 0.311 | 0.315 |
| 0.182 | 0.301 | 0.298 | 0.293 | 0.301 | 0.298 | 0.293 | 0.305 | 0.305 | 0.305 | 0.309 | 0.312 | 0.316 |
| 0.190 | 0.301 | 0.297 | 0.291 | 0.301 | 0.298 | 0.292 | 0.305 | 0.305 | 0.305 | 0.310 | 0.313 | 0.318 |
| 0.199 | 0.301 | 0.297 | 0.290 | 0.301 | 0.297 | 0.291 | 0.305 | 0.305 | 0.306 | 0.310 | 0.314 | 0.320 |
| 0.207 | 0.300 | 0.296 | 0.288 | 0.301 | 0.296 | 0.289 | 0.306 | 0.305 | 0.306 | 0.311 | 0.315 | 0.321 |
| 0.216 | 0.300 | 0.295 | 0.287 | 0.300 | 0.295 | 0.287 | 0.306 | 0.306 | 0.307 | 0.312 | 0.316 | 0.323 |
| 0.225 | 0.299 | 0.294 | 0.285 | 0.300 | 0.294 | 0.285 | 0.306 | 0.306 | 0.307 | 0.313 | 0.317 | 0.325 |
| 0.233 | 0.299 | 0.293 | 0.282 | 0.299 | 0.293 | 0.283 | 0.307 | 0.306 | 0.308 | 0.314 | 0.319 | 0.328 |
| 0.242 | 0.298 | 0.291 | 0.280 | 0.299 | 0.291 | 0.281 | 0.307 | 0.307 | 0.308 | 0.315 | 0.320 | 0.330 |
| 0.251 | 0.297 | 0.290 | 0.277 | 0.298 | 0.290 | 0.278 | 0.308 | 0.307 | 0.309 | 0.316 | 0.322 | 0.332 |
| 0.259 | 0.297 | 0.288 | 0.274 | 0.297 | 0.288 | 0.274 | 0.308 | 0.308 | 0.310 | 0.317 | 0.324 | 0.335 |
| 0.268 | 0.296 | 0.286 | 0.270 | 0.296 | 0.286 | 0.271 | 0.308 | 0.308 | 0.311 | 0.318 | 0.326 | 0.338 |
| 0.277 | 0.294 | 0.283 | 0.265 | 0.295 | 0.283 | 0.266 | 0.309 | 0.309 | 0.312 | 0.319 | 0.327 | 0.341 |
| 0.285 | 0.293 | 0.280 | 0.260 | 0.293 | 0.280 | 0.261 | 0.310 | 0.310 | 0.314 | 0.320 | 0.330 | 0.344 |
| 0.294 | 0.291 | 0.276 | 0.254 | 0.291 | 0.276 | 0.254 | 0.311 | 0.311 | 0.317 | 0.321 | 0.332 | 0.348 |
| 0.303 | 0.289 | 0.272 | 0.247 | 0.289 | 0.271 | 0.247 | 0.312 | 0.312 | 0.319 | 0.323 | 0.334 | 0.352 |
| 0.311 | 0.287 | 0.267 | 0.239 | 0.287 | 0.266 | 0.238 | 0.313 | 0.314 | 0.323 | 0.324 | 0.336 | 0.355 |
| 0.320 | 0.284 | 0.261 | 0.228 | 0.284 | 0.260 | 0.228 | 0.315 | 0.317 | 0.328 | 0.326 | 0.339 | 0.360 |
| 0.328 | 0.281 | 0.254 | 0.216 | 0.281 | 0.252 | 0.215 | 0.317 | 0.321 | 0.334 | 0.327 | 0.342 | 0.364 |
| 0.337 | 0.276 | 0.245 | 0.203 | 0.278 | 0.244 | 0.201 | 0.320 | 0.325 | 0.341 | 0.329 | 0.345 | 0.368 |
| 0.346 | 0.267 | 0.234 | 0.185 | 0.278 | 0.236 | 0.183 | 0.324 | 0.331 | 0.350 | 0.331 | 0.347 | 0.373 |
| 0.354 | 0.240 | 0.214 | 0.164 | 0.289 | 0.237 | 0.172 | 0.328 | 0.338 | 0.361 | 0.332 | 0.350 | 0.378 |
| 0.363 | 0.000 | 0.000 | 0.000 | 0.329 | 0.322 | 0.263 | 0.333 | 0.345 | 0.373 | 0.334 | 0.354 | 0.383 |
| 0.372 | 0.000 | 0.000 | 0.000 | 0.365 | 0.403 | 0.371 | 0.339 | 0.353 | 0.386 | 0.336 | 0.357 | 0.388 |
| 0.380 | -0.019 | 0.007 | 0.000 | 0.390 | 0.372 | 0.376 | 0.344 | 0.361 | 0.399 | 0.337 | 0.360 | 0.393 |
| 0.389 | 0.015 | 0.000 | 0.007 | 0.388 | 0.401 | 0.321 | 0.348 | 0.369 | 0.412 | 0.339 | 0.363 | 0.398 |
| 0.398 | 0.004 | 0.008 | 0.000 | 0.384 | 0.395 | 0.353 | 0.352 | 0.376 | 0.424 | 0.341 | 0.366 | 0.403 |
| 0.406 | 0.000 | 0.000 | 0.035 | 0.365 | 0.385 | 0.265 | 0.355 | 0.383 | 0.435 | 0.342 | 0.370 | 0.408 |
| 0.415 | 0.064 | -0.010 | 0.000 | 0.362 | 0.371 | 0.293 | 0.358 | 0.389 | 0.445 | 0.344 | 0.373 | 0.413 |
| 0.424 | 0.089 | 0.013 | 0.000 | 0.360 | 0.347 | 0.255 | 0.361 | 0.395 | 0.452 | 0.345 | 0.376 | 0.417 |
| 0.432 | 0.025 | 0.000 | 0.000 | 0.374 | 0.365 | 0.235 | 0.364 | 0.401 | 0.455 | 0.347 | 0.379 | 0.422 |
| 0.441 | 0.000 | 0.013 | 0.000 | 0.389 | 0.323 | 0.261 | 0.366 | 0.406 | 0.455 | 0.348 | 0.382 | 0.427 |
| 0.449 | 0.029 | 0.000 | 0.008 | 0.385 | 0.348 | 0.218 | 0.367 | 0.410 | 0.452 | 0.350 | 0.385 | 0.431 |
| 0.458 | 0.071 | 0.023 | 0.000 | 0.381 | 0.343 | 0.254 | 0.369 | 0.414 | 0.446 | 0.351 | 0.388 | 0.435 |
| 0.467 | 0.039 | 0.041 | 0.018 | 0.387 | 0.340 | 0.182 | 0.370 | 0.418 | 0.436 | 0.352 | 0.390 | 0.439 |
| 0.475 | 0.000 | 0.000 | 0.000 | 0.394 | 0.355 | 0.206 | 0.370 | 0.420 | 0.425 | 0.353 | 0.393 | 0.443 |
| 0.484 | 0.064 | 0.067 | 0.077 | 0.379 | 0.294 | 0.129 | 0.370 | 0.421 | 0.414 | 0.354 | 0.395 | 0.447 |
| 0.493 | 0.104 | 0.141 | 0.114 | 0.362 | 0.247 | 0.107 | 0.369 | 0.422 | 0.400 | 0.355 | 0.398 | 0.450 |
| 0.501 | 0.129 | 0.169 | 0.116 | 0.353 | 0.239 | 0.105 | 0.368 | 0.421 | 0.388 | 0.356 | 0.400 | 0.453 |
| 0.510 | 0.145 | 0.178 | 0.115 | 0.345 | 0.232 | 0.107 | 0.366 | 0.419 | 0.373 | 0.357 | 0.402 | 0.457 |
| 0.519 | 0.159 | 0.184 | 0.114 | 0.337 | 0.226 | 0.109 | 0.365 | 0.417 | 0.360 | 0.358 | 0.404 | 0.459 |
| 0.527 | 0.170 | 0.187 | 0.112 | 0.330 | 0.220 | 0.108 | 0.364 | 0.414 | 0.347 | 0.359 | 0.406 | 0.462 |
| 0.536 | 0.179 | 0.189 | 0.112 | 0.322 | 0.215 | 0.106 | 0.363 | 0.410 | 0.335 | 0.359 | 0.408 | 0.465 |
| 0.545 | 0.185 | 0.190 | 0.111 | 0.314 | 0.210 | 0.107 | 0.362 | 0.406 | 0.323 | 0.360 | 0.410 | 0.467 |
| 0.553 | 0.191 | 0.190 | 0.109 | 0.306 | 0.206 | 0.107 | 0.361 | 0.400 | 0.313 | 0.361 | 0.412 | 0.470 |
| 0.562 | 0.196 | 0.190 | 0.107 | 0.298 | 0.202 | 0.106 | 0.360 | 0.394 | 0.302 | 0.362 | 0.414 | 0.472 |
| 0.570 | 0.201 | 0.190 | 0.104 | 0.290 | 0.198 | 0.105 | 0.360 | 0.387 | 0.293 | 0.363 | 0.416 | 0.474 |
| 0.579 | 0.205 | 0.188 | 0.101 | 0.282 | 0.195 | 0.103 | 0.360 | 0.380 | 0.284 | 0.364 | 0.417 | 0.476 |
| 0.588 | 0.209 | 0.187 | 0.098 | 0.275 | 0.191 | 0.100 | 0.360 | 0.372 | 0.276 | 0.364 | 0.419 | 0.478 |
| 0.596 | 0.213 | 0.185 | 0.095 | 0.269 | 0.187 | 0.097 | 0.360 | 0.364 | 0.268 | 0.365 | 0.421 | 0.480 |
| 0.605 | 0.215 | 0.182 | 0.090 | 0.263 | 0.184 | 0.092 | 0.360 | 0.355 | 0.261 | 0.366 | 0.423 | 0.482 |
| 0.614 | 0.217 | 0.178 | 0.083 | 0.257 | 0.180 | 0.089 | 0.361 | 0.346 | 0.253 | 0.367 | 0.424 | 0.484 |
| 0.622 | 0.219 | 0.174 | 0.075 | 0.251 | 0.175 | 0.083 | 0.362 | 0.338 | 0.246 | 0.368 | 0.426 | 0.485 |
| 0.631 | 0.219 | 0.168 | 0.067 | 0.246 | 0.170 | 0.076 | 0.363 | 0.329 | 0.239 | 0.369 | 0.428 | 0.487 |
| 0.640 | 0.219 | 0.162 | 0.058 | 0.241 | 0.164 | 0.067 | 0.366 | 0.321 | 0.233 | 0.371 | 0.430 | 0.489 |
| 0.648 | 0.213 | 0.152 | 0.047 | 0.241 | 0.156 | 0.058 | 0.369 | 0.314 | 0.229 | 0.372 | 0.432 | 0.490 |
| 0.657 | 0.177 | 0.128 | 0.036 | 0.256 | 0.160 | 0.050 | 0.372 | 0.307 | 0.226 | 0.373 | 0.433 | 0.492 |
| 0.666 | 0.000 | 0.000 | 0.000 | 0.290 | 0.232 | 0.096 | 0.375 | 0.302 | 0.224 | 0.374 | 0.435 | 0.493 |
| 0.674 | -0.014 | -0.005 | 0.015 | 0.310 | 0.253 | 0.071 | 0.378 | 0.297 | 0.224 | 0.375 | 0.437 | 0.495 |
| 0.683 | 0.041 | 0.026 | 0.000 | 0.290 | 0.223 | 0.096 | 0.380 | 0.294 | 0.225 | 0.377 | 0.439 | 0.496 |
| 0.691 | 0.079 | 0.000 | 0.022 | 0.290 | 0.240 | 0.055 | 0.380 | 0.291 | 0.225 | 0.378 | 0.441 | 0.497 |
| 0.700 | 0.000 | 0.033 | 0.000 | 0.317 | 0.207 | 0.087 | 0.380 | 0.288 | 0.224 | 0.379 | 0.442 | 0.499 |
| 0.709 | 0.005 | 0.000 | 0.000 | 0.305 | 0.230 | 0.064 | 0.378 | 0.287 | 0.228 | 0.380 | 0.444 | 0.500 |
| 0.717 | 0.064 | 0.035 | 0.000 | 0.299 | 0.219 | 0.075 | 0.376 | 0.286 | 0.230 | 0.381 | 0.446 | 0.501 |
| 0.726 | 0.086 | 0.000 | 0.000 | 0.282 | 0.199 | 0.064 | 0.373 | 0.285 | 0.231 | 0.383 | 0.448 | 0.503 |
| 0.735 | 0.000 | 0.000 | 0.012 | 0.302 | 0.217 | 0.053 | 0.371 | 0.285 | 0.232 | 0.384 | 0.449 | 0.504 |
| 0.743 | -0.014 | 0.042 | 0.000 | 0.307 | 0.188 | 0.077 | 0.369 | 0.285 | 0.234 | 0.385 | 0.451 | 0.505 |
| 0.752 | 0.057 | 0.000 | 0.007 | 0.299 | 0.210 | 0.057 | 0.367 | 0.286 | 0.235 | 0.386 | 0.452 | 0.506 |
| 0.761 | 0.086 | 0.007 | 0.000 | 0.296 | 0.183 | 0.073 | 0.365 | 0.286 | 0.237 | 0.387 | 0.454 | 0.507 |
| 0.769 | 0.000 | 0.000 | 0.000 | 0.301 | 0.198 | 0.067 | 0.363 | 0.287 | 0.238 | 0.388 | 0.455 | 0.508 |
| 0.778 | -0.011 | 0.001 | -0.004 | 0.303 | 0.188 | 0.058 | 0.361 | 0.286 | 0.238 | 0.389 | 0.456 | 0.508 |
| 0.787 | 0.062 | 0.059 | 0.020 | 0.291 | 0.161 | 0.035 | 0.358 | 0.286 | 0.239 | 0.389 | 0.458 | 0.509 |
| 0.795 | 0.098 | 0.099 | 0.026 | 0.278 | 0.136 | 0.025 | 0.356 | 0.285 | 0.239 | 0.390 | 0.459 | 0.510 |
| 0.804 | 0.121 | 0.107 | 0.025 | 0.268 | 0.119 | 0.023 | 0.354 | 0.284 | 0.240 | 0.391 | 0.460 | 0.511 |
| 0.812 | 0.137 | 0.110 | 0.023 | 0.260 | 0.110 | 0.022 | 0.352 | 0.283 | 0.240 | 0.392 | 0.461 | 0.511 |
| 0.821 | 0.148 | 0.111 | 0.021 | 0.253 | 0.102 | 0.020 | 0.350 | 0.282 | 0.241 | 0.392 | 0.462 | 0.512 |
| 0.830 | 0.157 | 0.112 | 0.020 | 0.247 | 0.098 | 0.018 | 0.348 | 0.281 | 0.241 | 0.393 | 0.463 | 0.512 |
| 0.838 | 0.164 | 0.112 | 0.018 | 0.241 | 0.095 | 0.016 | 0.346 | 0.280 | 0.241 | 0.394 | 0.464 | 0.513 |
| 0.847 | 0.169 | 0.111 | 0.017 | 0.236 | 0.093 | 0.015 | 0.345 | 0.279 | 0.242 | 0.395 | 0.465 | 0.514 |
| 0.856 | 0.174 | 0.111 | 0.016 | 0.231 | 0.091 | 0.014 | 0.343 | 0.278 | 0.242 | 0.395 | 0.466 | 0.514 |
| 0.864 | 0.178 | 0.110 | 0.014 | 0.227 | 0.091 | 0.013 | 0.342 | 0.277 | 0.242 | 0.396 | 0.467 | 0.514 |
| 0.873 | 0.181 | 0.109 | 0.013 | 0.223 | 0.090 | 0.012 | 0.341 | 0.276 | 0.242 | 0.397 | 0.468 | 0.515 |
| 0.882 | 0.184 | 0.108 | 0.012 | 0.219 | 0.090 | 0.011 | 0.340 | 0.275 | 0.242 | 0.397 | 0.469 | 0.515 |
| 0.890 | 0.186 | 0.107 | 0.012 | 0.215 | 0.090 | 0.010 | 0.338 | 0.274 | 0.242 | 0.398 | 0.470 | 0.516 |
| 0.899 | 0.187 | 0.105 | 0.011 | 0.212 | 0.090 | 0.009 | 0.337 | 0.273 | 0.242 | 0.399 | 0.471 | 0.516 |
| 0.908 | 0.189 | 0.103 | 0.009 | 0.208 | 0.089 | 0.007 | 0.336 | 0.272 | 0.242 | 0.400 | 0.472 | 0.517 |
| 0.916 | 0.190 | 0.101 | 0.008 | 0.205 | 0.089 | 0.005 | 0.336 | 0.271 | 0.241 | 0.401 | 0.473 | 0.517 |
| 0.925 | 0.190 | 0.098 | 0.006 | 0.202 | 0.089 | 0.002 | 0.335 | 0.269 | 0.241 | 0.402 | 0.474 | 0.517 |
| 0.933 | 0.190 | 0.095 | 0.002 | 0.200 | 0.088 | -0.003 | 0.335 | 0.268 | 0.240 | 0.403 | 0.475 | 0.518 |
| 0.942 | 0.188 | 0.092 | -0.003 | 0.198 | 0.087 | -0.009 | 0.334 | 0.268 | 0.240 | 0.403 | 0.476 | 0.518 |
| 0.951 | 0.181 | 0.086 | -0.009 | 0.201 | 0.086 | -0.013 | 0.335 | 0.267 | 0.240 | 0.404 | 0.477 | 0.519 |
| 0.959 | 0.124 | 0.060 | -0.013 | 0.217 | 0.099 | -0.009 | 0.335 | 0.267 | 0.239 | 0.405 | 0.478 | 0.519 |
| 0.968 | 0.000 | 0.000 | 0.000 | 0.235 | 0.147 | 0.002 | 0.336 | 0.267 | 0.240 | 0.406 | 0.479 | 0.520 |
| 0.977 | 0.059 | 0.022 | -0.007 | 0.253 | 0.125 | 0.013 | 0.337 | 0.267 | 0.240 | 0.407 | 0.480 | 0.520 |
| 0.985 | 0.092 | 0.000 | 0.000 | 0.239 | 0.143 | 0.022 | 0.337 | 0.267 | 0.240 | 0.408 | 0.481 | 0.520 |
| 0.994 | 0.091 | -0.006 | 0.000 | 0.254 | 0.129 | 0.040 | 0.337 | 0.267 | 0.240 | 0.409 | 0.482 | 0.521 |
| 1.000 | 0.000 | 0.029 | 0.005 | 0.264 | 0.109 | 0.030 | 0.337 | 0.267 | 0.242 | 0.410 | 0.483 | 0.521 |
| 1.010 | 0.036 | 0.000 | 0.000 | 0.255 | 0.133 | 0.051 | 0.336 | 0.268 | 0.243 | 0.411 | 0.484 | 0.522 |
| 1.020 | 0.076 | 0.039 | 0.018 | 0.251 | 0.110 | 0.027 | 0.336 | 0.268 | 0.244 | 0.412 | 0.485 | 0.522 |
| 1.030 | 0.076 | 0.000 | 0.000 | 0.252 | 0.125 | 0.058 | 0.336 | 0.269 | 0.244 | 0.413 | 0.486 | 0.522 |
| 1.040 | 0.000 | -0.009 | 0.006 | 0.259 | 0.131 | 0.033 | 0.335 | 0.269 | 0.245 | 0.414 | 0.486 | 0.523 |
| 1.050 | 0.050 | 0.011 | 0.000 | 0.252 | 0.132 | 0.042 | 0.335 | 0.270 | 0.248 | 0.415 | 0.487 | 0.523 |
| 1.050 | 0.080 | 0.000 | 0.000 | 0.249 | 0.134 | 0.049 | 0.335 | 0.270 | 0.249 | 0.416 | 0.488 | 0.523 |
| 1.060 | 0.075 | 0.010 | 0.002 | 0.247 | 0.119 | 0.044 | 0.335 | 0.271 | 0.249 | 0.417 | 0.489 | 0.524 |
| 1.070 | 0.000 | 0.000 | 0.000 | 0.259 | 0.135 | 0.048 | 0.334 | 0.271 | 0.252 | 0.417 | 0.489 | 0.524 |
| 1.080 | -0.009 | 0.013 | 0.004 | 0.247 | 0.116 | 0.018 | 0.333 | 0.271 | 0.252 | 0.418 | 0.490 | 0.524 |
| 1.090 | 0.055 | 0.054 | 0.016 | 0.241 | 0.099 | 0.018 | 0.332 | 0.271 | 0.254 | 0.419 | 0.491 | 0.525 |
| 1.100 | 0.081 | 0.068 | 0.015 | 0.230 | 0.082 | 0.010 | 0.331 | 0.270 | 0.255 | 0.419 | 0.491 | 0.525 |
| 1.110 | 0.100 | 0.069 | 0.010 | 0.221 | 0.075 | 0.007 | 0.330 | 0.270 | 0.256 | 0.420 | 0.492 | 0.525 |
| 1.110 | 0.113 | 0.070 | 0.005 | 0.214 | 0.071 | 0.003 | 0.329 | 0.269 | 0.257 | 0.421 | 0.493 | 0.525 |
| 1.120 | 0.123 | 0.070 | 0.001 | 0.208 | 0.067 | 0.000 | 0.328 | 0.269 | 0.258 | 0.421 | 0.493 | 0.525 |
| 1.130 | 0.131 | 0.070 | -0.002 | 0.203 | 0.065 | -0.003 | 0.327 | 0.268 | 0.259 | 0.422 | 0.494 | 0.526 |
| 1.140 | 0.137 | 0.069 | -0.004 | 0.198 | 0.063 | -0.006 | 0.326 | 0.268 | 0.260 | 0.422 | 0.494 | 0.526 |
| 1.150 | 0.142 | 0.068 | -0.006 | 0.193 | 0.061 | -0.008 | 0.326 | 0.267 | 0.261 | 0.423 | 0.494 | 0.526 |
| 1.160 | 0.146 | 0.067 | -0.008 | 0.189 | 0.060 | -0.010 | 0.325 | 0.267 | 0.262 | 0.424 | 0.495 | 0.526 |
| 1.170 | 0.149 | 0.066 | -0.010 | 0.186 | 0.058 | -0.012 | 0.325 | 0.267 | 0.262 | 0.424 | 0.495 | 0.526 |
| 1.180 | 0.152 | 0.065 | -0.012 | 0.182 | 0.057 | -0.014 | 0.324 | 0.266 | 0.263 | 0.425 | 0.496 | 0.526 |
| 1.180 | 0.155 | 0.063 | -0.014 | 0.179 | 0.056 | -0.016 | 0.323 | 0.266 | 0.264 | 0.425 | 0.496 | 0.527 |
| 1.190 | 0.157 | 0.062 | -0.015 | 0.177 | 0.054 | -0.018 | 0.323 | 0.266 | 0.264 | 0.426 | 0.497 | 0.527 |
| 1.200 | 0.158 | 0.060 | -0.017 | 0.174 | 0.053 | -0.020 | 0.323 | 0.265 | 0.264 | 0.427 | 0.497 | 0.527 |
| 1.210 | 0.159 | 0.058 | -0.019 | 0.172 | 0.052 | -0.023 | 0.322 | 0.264 | 0.265 | 0.427 | 0.498 | 0.527 |
| 1.220 | 0.160 | 0.056 | -0.021 | 0.170 | 0.051 | -0.027 | 0.322 | 0.264 | 0.265 | 0.428 | 0.498 | 0.527 |
| 1.230 | 0.160 | 0.054 | -0.022 | 0.168 | 0.049 | -0.030 | 0.322 | 0.263 | 0.265 | 0.429 | 0.499 | 0.528 |
| 1.240 | 0.160 | 0.052 | -0.021 | 0.166 | 0.048 | -0.031 | 0.322 | 0.263 | 0.266 | 0.429 | 0.500 | 0.528 |
| 1.240 | 0.158 | 0.050 | -0.016 | 0.165 | 0.048 | -0.028 | 0.323 | 0.263 | 0.267 | 0.430 | 0.500 | 0.528 |
| 1.250 | 0.148 | 0.047 | -0.009 | 0.169 | 0.050 | -0.018 | 0.324 | 0.264 | 0.268 | 0.431 | 0.501 | 0.528 |
| 1.260 | 0.066 | 0.018 | -0.011 | 0.188 | 0.067 | 0.000 | 0.325 | 0.265 | 0.270 | 0.432 | 0.501 | 0.529 |
| 1.270 | 0.000 | 0.000 | 0.000 | 0.213 | 0.099 | 0.004 | 0.326 | 0.266 | 0.273 | 0.432 | 0.502 | 0.529 |
| 1.280 | 0.065 | 0.040 | 0.000 | 0.203 | 0.078 | 0.038 | 0.327 | 0.267 | 0.273 | 0.433 | 0.503 | 0.529 |
| 1.290 | 0.091 | 0.000 | 0.011 | 0.211 | 0.096 | 0.033 | 0.327 | 0.269 | 0.275 | 0.434 | 0.503 | 0.530 |
| 1.300 | 0.045 | 0.015 | 0.000 | 0.215 | 0.080 | 0.070 | 0.328 | 0.270 | 0.276 | 0.435 | 0.504 | 0.530 |
| 1.310 | 0.000 | 0.000 | 0.013 | 0.221 | 0.099 | 0.049 | 0.328 | 0.271 | 0.278 | 0.435 | 0.504 | 0.530 |
| 1.310 | 0.047 | 0.000 | 0.000 | 0.224 | 0.101 | 0.067 | 0.329 | 0.272 | 0.281 | 0.436 | 0.505 | 0.530 |
| 1.320 | 0.077 | 0.029 | 0.003 | 0.211 | 0.087 | 0.049 | 0.329 | 0.274 | 0.284 | 0.437 | 0.505 | 0.531 |
| 1.330 | 0.055 | 0.000 | 0.000 | 0.224 | 0.103 | 0.067 | 0.329 | 0.275 | 0.286 | 0.437 | 0.506 | 0.531 |
| 1.340 | 0.000 | 0.013 | 0.000 | 0.229 | 0.086 | 0.063 | 0.330 | 0.276 | 0.287 | 0.438 | 0.506 | 0.531 |
| 1.350 | 0.035 | 0.000 | 0.004 | 0.222 | 0.100 | 0.059 | 0.330 | 0.277 | 0.289 | 0.439 | 0.507 | 0.531 |
| 1.360 | 0.071 | 0.011 | 0.000 | 0.218 | 0.088 | 0.073 | 0.330 | 0.278 | 0.291 | 0.439 | 0.507 | 0.531 |
| 1.370 | 0.050 | 0.021 | -0.003 | 0.229 | 0.082 | 0.051 | 0.330 | 0.279 | 0.294 | 0.440 | 0.507 | 0.531 |
| 1.370 | 0.000 | 0.000 | 0.000 | 0.225 | 0.080 | 0.061 | 0.330 | 0.280 | 0.295 | 0.440 | 0.508 | 0.531 |
| 1.380 | 0.051 | 0.017 | 0.015 | 0.207 | 0.097 | 0.039 | 0.329 | 0.280 | 0.296 | 0.440 | 0.508 | 0.531 |
| 1.390 | 0.077 | 0.039 | 0.021 | 0.203 | 0.081 | 0.026 | 0.329 | 0.281 | 0.298 | 0.441 | 0.508 | 0.531 |
| 1.400 | 0.094 | 0.046 | 0.018 | 0.198 | 0.072 | 0.019 | 0.328 | 0.281 | 0.299 | 0.441 | 0.509 | 0.531 |
| 1.410 | 0.105 | 0.049 | 0.014 | 0.191 | 0.066 | 0.014 | 0.328 | 0.282 | 0.302 | 0.441 | 0.509 | 0.531 |
| 1.420 | 0.113 | 0.050 | 0.008 | 0.185 | 0.062 | 0.008 | 0.327 | 0.282 | 0.304 | 0.442 | 0.509 | 0.531 |
| 1.430 | 0.118 | 0.051 | 0.003 | 0.180 | 0.059 | 0.003 | 0.327 | 0.282 | 0.305 | 0.442 | 0.509 | 0.531 |
| 1.430 | 0.123 | 0.052 | 0.000 | 0.176 | 0.056 | -0.001 | 0.326 | 0.283 | 0.306 | 0.442 | 0.509 | 0.531 |
| 1.440 | 0.126 | 0.051 | -0.003 | 0.172 | 0.053 | -0.004 | 0.326 | 0.284 | 0.307 | 0.442 | 0.509 | 0.531 |
| 1.450 | 0.129 | 0.051 | -0.005 | 0.168 | 0.051 | -0.006 | 0.326 | 0.285 | 0.308 | 0.442 | 0.509 | 0.530 |
| 1.460 | 0.132 | 0.050 | -0.007 | 0.165 | 0.049 | -0.008 | 0.326 | 0.286 | 0.310 | 0.443 | 0.509 | 0.530 |
| 1.470 | 0.134 | 0.049 | -0.008 | 0.162 | 0.047 | -0.010 | 0.326 | 0.287 | 0.311 | 0.443 | 0.509 | 0.530 |
| 1.480 | 0.136 | 0.048 | -0.010 | 0.160 | 0.046 | -0.012 | 0.326 | 0.289 | 0.312 | 0.443 | 0.509 | 0.530 |
| 1.490 | 0.138 | 0.047 | -0.012 | 0.158 | 0.044 | -0.014 | 0.326 | 0.290 | 0.311 | 0.443 | 0.509 | 0.529 |
| 1.500 | 0.140 | 0.045 | -0.013 | 0.156 | 0.043 | -0.015 | 0.326 | 0.291 | 0.313 | 0.443 | 0.509 | 0.529 |
| 1.500 | 0.142 | 0.044 | -0.014 | 0.154 | 0.041 | -0.016 | 0.326 | 0.292 | 0.313 | 0.443 | 0.509 | 0.528 |
| 1.510 | 0.143 | 0.042 | -0.014 | 0.153 | 0.039 | -0.017 | 0.326 | 0.293 | 0.314 | 0.443 | 0.509 | 0.528 |
| 1.520 | 0.144 | 0.040 | -0.014 | 0.152 | 0.037 | -0.017 | 0.326 | 0.294 | 0.314 | 0.443 | 0.509 | 0.528 |
| 1.530 | 0.145 | 0.038 | -0.014 | 0.151 | 0.035 | -0.016 | 0.326 | 0.295 | 0.314 | 0.443 | 0.509 | 0.527 |
| 1.540 | 0.146 | 0.035 | -0.013 | 0.150 | 0.033 | -0.015 | 0.326 | 0.296 | 0.315 | 0.443 | 0.508 | 0.527 |
| 1.550 | 0.147 | 0.032 | -0.011 | 0.149 | 0.029 | -0.014 | 0.326 | 0.296 | 0.316 | 0.443 | 0.508 | 0.526 |
| 1.560 | 0.148 | 0.028 | -0.009 | 0.149 | 0.025 | -0.012 | 0.326 | 0.297 | 0.317 | 0.443 | 0.508 | 0.526 |
| 1.560 | 0.148 | 0.023 | -0.006 | 0.148 | 0.019 | -0.009 | 0.326 | 0.297 | 0.318 | 0.443 | 0.508 | 0.525 |
| 1.570 | 0.149 | 0.017 | -0.003 | 0.148 | 0.013 | -0.006 | 0.326 | 0.297 | 0.320 | 0.443 | 0.508 | 0.525 |
| 1.580 | 0.149 | 0.011 | 0.001 | 0.147 | 0.007 | -0.002 | 0.326 | 0.297 | 0.318 | 0.444 | 0.508 | 0.524 |
| 1.590 | 0.150 | 0.008 | 0.004 | 0.147 | 0.004 | 0.002 | 0.326 | 0.297 | 0.316 | 0.444 | 0.507 | 0.524 |
| 1.600 | 0.150 | 0.007 | 0.009 | 0.147 | 0.003 | 0.006 | 0.326 | 0.297 | 0.316 | 0.444 | 0.507 | 0.523 |
| 1.610 | 0.150 | 0.008 | 0.013 | 0.147 | 0.003 | 0.011 | 0.326 | 0.297 | 0.316 | 0.444 | 0.507 | 0.522 |
| 1.620 | 0.151 | 0.010 | 0.017 | 0.146 | 0.005 | 0.015 | 0.326 | 0.297 | 0.315 | 0.444 | 0.507 | 0.522 |
| 1.620 | 0.151 | 0.012 | 0.022 | 0.146 | 0.007 | 0.020 | 0.326 | 0.298 | 0.315 | 0.444 | 0.506 | 0.521 |
| 1.630 | 0.151 | 0.015 | 0.026 | 0.146 | 0.010 | 0.025 | 0.325 | 0.298 | 0.314 | 0.444 | 0.506 | 0.521 |
| 1.640 | 0.151 | 0.018 | 0.030 | 0.146 | 0.013 | 0.029 | 0.325 | 0.298 | 0.314 | 0.444 | 0.506 | 0.520 |
| 1.650 | 0.151 | 0.021 | 0.035 | 0.146 | 0.016 | 0.034 | 0.325 | 0.298 | 0.313 | 0.444 | 0.506 | 0.520 |
| 1.660 | 0.151 | 0.025 | 0.039 | 0.146 | 0.020 | 0.038 | 0.325 | 0.298 | 0.313 | 0.444 | 0.506 | 0.519 |
| 1.670 | 0.152 | 0.028 | 0.043 | 0.146 | 0.023 | 0.042 | 0.325 | 0.298 | 0.312 | 0.444 | 0.505 | 0.519 |
| 1.680 | 0.152 | 0.032 | 0.047 | 0.146 | 0.027 | 0.047 | 0.325 | 0.298 | 0.311 | 0.444 | 0.505 | 0.518 |
| 1.690 | 0.152 | 0.035 | 0.052 | 0.146 | 0.031 | 0.051 | 0.324 | 0.297 | 0.310 | 0.444 | 0.505 | 0.518 |
| 1.690 | 0.152 | 0.039 | 0.056 | 0.146 | 0.035 | 0.055 | 0.324 | 0.297 | 0.309 | 0.444 | 0.505 | 0.518 |
| 1.700 | 0.152 | 0.043 | 0.060 | 0.146 | 0.040 | 0.059 | 0.323 | 0.296 | 0.308 | 0.444 | 0.505 | 0.517 |
| 1.710 | 0.152 | 0.048 | 0.065 | 0.147 | 0.045 | 0.064 | 0.323 | 0.295 | 0.307 | 0.445 | 0.505 | 0.517 |
| 1.720 | 0.153 | 0.053 | 0.069 | 0.147 | 0.051 | 0.068 | 0.322 | 0.294 | 0.305 | 0.445 | 0.505 | 0.517 |

**Table. 4** In the submerged state (*h*=0.09 m), the streamwise velocity *u* of the three vegetation patch coverage conditions changes along the *x* direction.

| **Hydraulic parameters** | **Streamwise velocity *u* (m/s)** | | | | | | | | | | | |
| --- | --- | --- | --- | --- | --- | --- | --- | --- | --- | --- | --- | --- |
| ***x* (m)** | **L_1-1_** | | | **L_2-2_** | | | **L_3-3_** | | | **L_4-4_** | | |
|  | **Cr=2.09%** | **Cr=4.71%** | **Cr=8.37%** | **Cr=2.09%** | **Cr=4.71%** | **Cr=8.37%** | **Cr=2.09%** | **Cr=4.71%** | **Cr=8.37%** | **Cr=2.09%** | **Cr=4.71%** | **Cr=8.37%** |
| 0.000 | 0.300 | 0.300 | 0.300 | 0.300 | 0.300 | 0.300 | 0.300 | 0.300 | 0.300 | 0.300 | 0.300 | 0.300 |
| 0.009 | 0.300 | 0.300 | 0.300 | 0.300 | 0.300 | 0.300 | 0.300 | 0.300 | 0.300 | 0.300 | 0.300 | 0.300 |
| 0.017 | 0.300 | 0.300 | 0.300 | 0.300 | 0.300 | 0.300 | 0.300 | 0.300 | 0.300 | 0.300 | 0.300 | 0.300 |
| 0.026 | 0.300 | 0.300 | 0.300 | 0.300 | 0.300 | 0.300 | 0.300 | 0.300 | 0.300 | 0.300 | 0.300 | 0.300 |
| 0.035 | 0.300 | 0.300 | 0.300 | 0.300 | 0.300 | 0.300 | 0.300 | 0.300 | 0.300 | 0.300 | 0.300 | 0.300 |
| 0.043 | 0.300 | 0.300 | 0.300 | 0.300 | 0.300 | 0.300 | 0.300 | 0.300 | 0.300 | 0.300 | 0.300 | 0.301 |
| 0.052 | 0.300 | 0.300 | 0.300 | 0.300 | 0.300 | 0.300 | 0.300 | 0.300 | 0.300 | 0.300 | 0.301 | 0.301 |
| 0.061 | 0.300 | 0.300 | 0.300 | 0.300 | 0.300 | 0.300 | 0.300 | 0.300 | 0.300 | 0.301 | 0.301 | 0.301 |
| 0.069 | 0.300 | 0.300 | 0.300 | 0.300 | 0.300 | 0.300 | 0.300 | 0.301 | 0.300 | 0.301 | 0.301 | 0.302 |
| 0.078 | 0.300 | 0.300 | 0.299 | 0.300 | 0.300 | 0.299 | 0.301 | 0.301 | 0.301 | 0.301 | 0.301 | 0.302 |
| 0.086 | 0.300 | 0.300 | 0.299 | 0.300 | 0.300 | 0.299 | 0.301 | 0.301 | 0.301 | 0.301 | 0.302 | 0.302 |
| 0.095 | 0.300 | 0.300 | 0.299 | 0.300 | 0.300 | 0.299 | 0.301 | 0.301 | 0.301 | 0.301 | 0.302 | 0.303 |
| 0.104 | 0.300 | 0.300 | 0.299 | 0.300 | 0.300 | 0.299 | 0.301 | 0.301 | 0.301 | 0.302 | 0.302 | 0.303 |
| 0.112 | 0.300 | 0.300 | 0.299 | 0.300 | 0.300 | 0.299 | 0.301 | 0.301 | 0.301 | 0.302 | 0.303 | 0.304 |
| 0.121 | 0.300 | 0.299 | 0.298 | 0.300 | 0.299 | 0.298 | 0.301 | 0.301 | 0.301 | 0.302 | 0.303 | 0.304 |
| 0.130 | 0.300 | 0.299 | 0.298 | 0.300 | 0.299 | 0.298 | 0.301 | 0.301 | 0.301 | 0.303 | 0.304 | 0.305 |
| 0.138 | 0.300 | 0.299 | 0.297 | 0.300 | 0.299 | 0.298 | 0.301 | 0.302 | 0.302 | 0.303 | 0.304 | 0.306 |
| 0.147 | 0.300 | 0.299 | 0.297 | 0.300 | 0.299 | 0.297 | 0.302 | 0.302 | 0.302 | 0.303 | 0.305 | 0.306 |
| 0.156 | 0.300 | 0.298 | 0.296 | 0.300 | 0.299 | 0.297 | 0.302 | 0.302 | 0.302 | 0.303 | 0.305 | 0.307 |
| 0.164 | 0.300 | 0.298 | 0.296 | 0.300 | 0.298 | 0.296 | 0.302 | 0.302 | 0.302 | 0.304 | 0.306 | 0.308 |
| 0.173 | 0.300 | 0.298 | 0.295 | 0.300 | 0.298 | 0.295 | 0.302 | 0.302 | 0.302 | 0.304 | 0.306 | 0.309 |
| 0.182 | 0.300 | 0.297 | 0.294 | 0.300 | 0.298 | 0.295 | 0.302 | 0.302 | 0.302 | 0.305 | 0.307 | 0.310 |
| 0.190 | 0.300 | 0.297 | 0.293 | 0.300 | 0.297 | 0.294 | 0.302 | 0.302 | 0.303 | 0.305 | 0.308 | 0.311 |
| 0.199 | 0.300 | 0.296 | 0.293 | 0.300 | 0.297 | 0.293 | 0.303 | 0.303 | 0.303 | 0.305 | 0.308 | 0.312 |
| 0.207 | 0.300 | 0.296 | 0.292 | 0.300 | 0.296 | 0.292 | 0.303 | 0.303 | 0.303 | 0.306 | 0.309 | 0.313 |
| 0.216 | 0.299 | 0.295 | 0.290 | 0.299 | 0.296 | 0.291 | 0.303 | 0.303 | 0.304 | 0.306 | 0.310 | 0.314 |
| 0.225 | 0.299 | 0.294 | 0.289 | 0.299 | 0.295 | 0.290 | 0.303 | 0.303 | 0.304 | 0.307 | 0.311 | 0.316 |
| 0.233 | 0.299 | 0.294 | 0.288 | 0.299 | 0.294 | 0.288 | 0.303 | 0.304 | 0.304 | 0.307 | 0.312 | 0.317 |
| 0.242 | 0.299 | 0.293 | 0.286 | 0.299 | 0.293 | 0.287 | 0.303 | 0.304 | 0.305 | 0.308 | 0.313 | 0.319 |
| 0.251 | 0.298 | 0.292 | 0.285 | 0.298 | 0.292 | 0.285 | 0.304 | 0.304 | 0.305 | 0.308 | 0.314 | 0.320 |
| 0.259 | 0.298 | 0.291 | 0.283 | 0.298 | 0.291 | 0.283 | 0.304 | 0.305 | 0.306 | 0.309 | 0.315 | 0.322 |
| 0.268 | 0.297 | 0.290 | 0.281 | 0.298 | 0.290 | 0.281 | 0.304 | 0.305 | 0.307 | 0.310 | 0.317 | 0.324 |
| 0.277 | 0.297 | 0.288 | 0.278 | 0.297 | 0.289 | 0.279 | 0.305 | 0.306 | 0.308 | 0.310 | 0.318 | 0.326 |
| 0.285 | 0.297 | 0.287 | 0.276 | 0.297 | 0.287 | 0.276 | 0.305 | 0.307 | 0.309 | 0.311 | 0.319 | 0.328 |
| 0.294 | 0.296 | 0.286 | 0.273 | 0.296 | 0.286 | 0.274 | 0.306 | 0.308 | 0.311 | 0.312 | 0.321 | 0.331 |
| 0.303 | 0.296 | 0.284 | 0.270 | 0.296 | 0.284 | 0.271 | 0.307 | 0.309 | 0.313 | 0.313 | 0.322 | 0.333 |
| 0.311 | 0.295 | 0.283 | 0.267 | 0.295 | 0.283 | 0.268 | 0.308 | 0.311 | 0.315 | 0.314 | 0.324 | 0.336 |
| 0.320 | 0.296 | 0.282 | 0.264 | 0.295 | 0.282 | 0.265 | 0.309 | 0.313 | 0.318 | 0.314 | 0.326 | 0.338 |
| 0.328 | 0.296 | 0.282 | 0.263 | 0.296 | 0.282 | 0.263 | 0.310 | 0.316 | 0.322 | 0.315 | 0.327 | 0.341 |
| 0.337 | 0.298 | 0.283 | 0.263 | 0.297 | 0.283 | 0.263 | 0.312 | 0.319 | 0.328 | 0.316 | 0.329 | 0.344 |
| 0.346 | 0.300 | 0.288 | 0.268 | 0.302 | 0.288 | 0.267 | 0.314 | 0.322 | 0.334 | 0.317 | 0.331 | 0.347 |
| 0.354 | 0.307 | 0.299 | 0.283 | 0.310 | 0.299 | 0.283 | 0.316 | 0.327 | 0.340 | 0.318 | 0.333 | 0.350 |
| 0.363 | 0.322 | 0.326 | 0.317 | 0.322 | 0.325 | 0.320 | 0.319 | 0.332 | 0.347 | 0.319 | 0.335 | 0.353 |
| 0.372 | 0.333 | 0.345 | 0.352 | 0.330 | 0.344 | 0.353 | 0.322 | 0.337 | 0.355 | 0.320 | 0.337 | 0.357 |
| 0.380 | 0.329 | 0.352 | 0.369 | 0.331 | 0.353 | 0.371 | 0.324 | 0.342 | 0.363 | 0.321 | 0.340 | 0.360 |
| 0.389 | 0.329 | 0.362 | 0.372 | 0.333 | 0.362 | 0.377 | 0.327 | 0.346 | 0.370 | 0.322 | 0.342 | 0.363 |
| 0.398 | 0.339 | 0.365 | 0.355 | 0.338 | 0.365 | 0.371 | 0.329 | 0.351 | 0.377 | 0.323 | 0.344 | 0.366 |
| 0.406 | 0.345 | 0.369 | 0.360 | 0.342 | 0.370 | 0.360 | 0.331 | 0.355 | 0.384 | 0.324 | 0.346 | 0.370 |
| 0.415 | 0.339 | 0.373 | 0.370 | 0.342 | 0.373 | 0.356 | 0.332 | 0.359 | 0.389 | 0.325 | 0.348 | 0.373 |
| 0.424 | 0.336 | 0.375 | 0.361 | 0.342 | 0.374 | 0.341 | 0.334 | 0.363 | 0.395 | 0.326 | 0.350 | 0.376 |
| 0.432 | 0.343 | 0.377 | 0.354 | 0.347 | 0.380 | 0.332 | 0.335 | 0.366 | 0.401 | 0.327 | 0.352 | 0.379 |
| 0.441 | 0.346 | 0.376 | 0.346 | 0.351 | 0.381 | 0.335 | 0.336 | 0.369 | 0.405 | 0.328 | 0.354 | 0.382 |
| 0.449 | 0.339 | 0.382 | 0.341 | 0.350 | 0.387 | 0.329 | 0.337 | 0.372 | 0.410 | 0.329 | 0.356 | 0.385 |
| 0.458 | 0.332 | 0.381 | 0.323 | 0.350 | 0.389 | 0.334 | 0.338 | 0.374 | 0.414 | 0.330 | 0.358 | 0.387 |
| 0.467 | 0.331 | 0.385 | 0.305 | 0.354 | 0.380 | 0.331 | 0.339 | 0.376 | 0.417 | 0.331 | 0.359 | 0.390 |
| 0.475 | 0.335 | 0.390 | 0.299 | 0.356 | 0.375 | 0.324 | 0.339 | 0.377 | 0.419 | 0.331 | 0.361 | 0.393 |
| 0.484 | 0.321 | 0.383 | 0.289 | 0.352 | 0.350 | 0.313 | 0.339 | 0.378 | 0.421 | 0.332 | 0.362 | 0.395 |
| 0.493 | 0.316 | 0.368 | 0.286 | 0.347 | 0.326 | 0.296 | 0.339 | 0.379 | 0.423 | 0.333 | 0.364 | 0.397 |
| 0.501 | 0.310 | 0.356 | 0.271 | 0.343 | 0.334 | 0.285 | 0.338 | 0.379 | 0.423 | 0.333 | 0.365 | 0.400 |
| 0.510 | 0.308 | 0.352 | 0.271 | 0.340 | 0.331 | 0.274 | 0.337 | 0.379 | 0.425 | 0.334 | 0.367 | 0.402 |
| 0.519 | 0.308 | 0.344 | 0.260 | 0.337 | 0.329 | 0.267 | 0.337 | 0.379 | 0.425 | 0.334 | 0.368 | 0.404 |
| 0.527 | 0.308 | 0.341 | 0.255 | 0.335 | 0.328 | 0.257 | 0.337 | 0.379 | 0.425 | 0.335 | 0.369 | 0.406 |
| 0.536 | 0.307 | 0.339 | 0.278 | 0.332 | 0.330 | 0.264 | 0.336 | 0.379 | 0.426 | 0.336 | 0.371 | 0.407 |
| 0.545 | 0.307 | 0.336 | 0.278 | 0.331 | 0.328 | 0.253 | 0.336 | 0.379 | 0.427 | 0.336 | 0.372 | 0.409 |
| 0.553 | 0.310 | 0.339 | 0.261 | 0.330 | 0.326 | 0.236 | 0.336 | 0.379 | 0.426 | 0.337 | 0.373 | 0.411 |
| 0.562 | 0.309 | 0.334 | 0.257 | 0.329 | 0.325 | 0.235 | 0.336 | 0.379 | 0.424 | 0.337 | 0.375 | 0.412 |
| 0.570 | 0.311 | 0.330 | 0.271 | 0.328 | 0.330 | 0.244 | 0.336 | 0.380 | 0.425 | 0.338 | 0.376 | 0.414 |
| 0.579 | 0.309 | 0.330 | 0.267 | 0.327 | 0.326 | 0.246 | 0.336 | 0.380 | 0.426 | 0.339 | 0.377 | 0.415 |
| 0.588 | 0.310 | 0.328 | 0.263 | 0.326 | 0.324 | 0.248 | 0.336 | 0.380 | 0.425 | 0.339 | 0.378 | 0.417 |
| 0.596 | 0.309 | 0.323 | 0.259 | 0.327 | 0.322 | 0.235 | 0.337 | 0.380 | 0.424 | 0.340 | 0.379 | 0.418 |
| 0.605 | 0.311 | 0.307 | 0.256 | 0.324 | 0.299 | 0.235 | 0.337 | 0.381 | 0.423 | 0.341 | 0.381 | 0.419 |
| 0.614 | 0.310 | 0.306 | 0.254 | 0.324 | 0.302 | 0.234 | 0.338 | 0.382 | 0.422 | 0.342 | 0.382 | 0.420 |
| 0.622 | 0.309 | 0.307 | 0.244 | 0.323 | 0.305 | 0.224 | 0.339 | 0.382 | 0.419 | 0.342 | 0.383 | 0.422 |
| 0.631 | 0.305 | 0.316 | 0.238 | 0.324 | 0.298 | 0.212 | 0.340 | 0.384 | 0.419 | 0.343 | 0.384 | 0.423 |
| 0.640 | 0.309 | 0.307 | 0.225 | 0.326 | 0.297 | 0.207 | 0.342 | 0.385 | 0.415 | 0.344 | 0.386 | 0.424 |
| 0.648 | 0.309 | 0.314 | 0.219 | 0.330 | 0.298 | 0.201 | 0.343 | 0.386 | 0.410 | 0.345 | 0.387 | 0.425 |
| 0.657 | 0.314 | 0.314 | 0.220 | 0.337 | 0.300 | 0.207 | 0.346 | 0.388 | 0.410 | 0.346 | 0.388 | 0.426 |
| 0.666 | 0.329 | 0.322 | 0.226 | 0.346 | 0.310 | 0.215 | 0.348 | 0.390 | 0.410 | 0.347 | 0.389 | 0.427 |
| 0.674 | 0.332 | 0.322 | 0.227 | 0.350 | 0.312 | 0.215 | 0.350 | 0.392 | 0.405 | 0.347 | 0.391 | 0.428 |
| 0.683 | 0.329 | 0.322 | 0.230 | 0.349 | 0.312 | 0.218 | 0.352 | 0.393 | 0.404 | 0.348 | 0.392 | 0.429 |
| 0.691 | 0.330 | 0.325 | 0.234 | 0.351 | 0.314 | 0.217 | 0.353 | 0.396 | 0.400 | 0.349 | 0.393 | 0.430 |
| 0.700 | 0.340 | 0.320 | 0.239 | 0.355 | 0.310 | 0.221 | 0.355 | 0.397 | 0.397 | 0.350 | 0.394 | 0.431 |
| 0.709 | 0.341 | 0.323 | 0.242 | 0.356 | 0.311 | 0.225 | 0.356 | 0.399 | 0.395 | 0.351 | 0.396 | 0.432 |
| 0.717 | 0.337 | 0.321 | 0.247 | 0.354 | 0.312 | 0.228 | 0.358 | 0.402 | 0.390 | 0.351 | 0.397 | 0.433 |
| 0.726 | 0.337 | 0.320 | 0.247 | 0.356 | 0.311 | 0.230 | 0.359 | 0.402 | 0.387 | 0.352 | 0.398 | 0.434 |
| 0.735 | 0.346 | 0.317 | 0.248 | 0.359 | 0.311 | 0.231 | 0.360 | 0.405 | 0.382 | 0.353 | 0.399 | 0.435 |
| 0.743 | 0.348 | 0.318 | 0.248 | 0.359 | 0.310 | 0.231 | 0.361 | 0.405 | 0.376 | 0.354 | 0.400 | 0.436 |
| 0.752 | 0.342 | 0.317 | 0.249 | 0.358 | 0.312 | 0.233 | 0.362 | 0.406 | 0.370 | 0.354 | 0.401 | 0.436 |
| 0.761 | 0.344 | 0.308 | 0.248 | 0.359 | 0.308 | 0.231 | 0.362 | 0.405 | 0.368 | 0.355 | 0.402 | 0.437 |
| 0.769 | 0.350 | 0.308 | 0.243 | 0.361 | 0.308 | 0.227 | 0.363 | 0.405 | 0.364 | 0.356 | 0.403 | 0.438 |
| 0.778 | 0.352 | 0.306 | 0.240 | 0.362 | 0.302 | 0.219 | 0.363 | 0.407 | 0.358 | 0.356 | 0.404 | 0.439 |
| 0.787 | 0.343 | 0.296 | 0.238 | 0.358 | 0.300 | 0.215 | 0.363 | 0.408 | 0.354 | 0.357 | 0.405 | 0.439 |
| 0.795 | 0.338 | 0.297 | 0.230 | 0.353 | 0.294 | 0.212 | 0.362 | 0.408 | 0.352 | 0.357 | 0.406 | 0.440 |
| 0.804 | 0.334 | 0.287 | 0.224 | 0.349 | 0.293 | 0.202 | 0.362 | 0.406 | 0.348 | 0.358 | 0.407 | 0.440 |
| 0.812 | 0.328 | 0.285 | 0.209 | 0.342 | 0.292 | 0.198 | 0.361 | 0.404 | 0.350 | 0.358 | 0.408 | 0.441 |
| 0.821 | 0.329 | 0.281 | 0.212 | 0.339 | 0.291 | 0.199 | 0.361 | 0.406 | 0.343 | 0.359 | 0.408 | 0.442 |
| 0.830 | 0.328 | 0.290 | 0.206 | 0.339 | 0.294 | 0.200 | 0.360 | 0.404 | 0.339 | 0.359 | 0.409 | 0.442 |
| 0.838 | 0.332 | 0.285 | 0.213 | 0.337 | 0.295 | 0.200 | 0.360 | 0.403 | 0.338 | 0.360 | 0.410 | 0.443 |
| 0.847 | 0.327 | 0.293 | 0.212 | 0.335 | 0.287 | 0.198 | 0.360 | 0.399 | 0.330 | 0.360 | 0.410 | 0.443 |
| 0.856 | 0.324 | 0.288 | 0.204 | 0.334 | 0.287 | 0.199 | 0.360 | 0.402 | 0.330 | 0.361 | 0.411 | 0.444 |
| 0.864 | 0.329 | 0.279 | 0.207 | 0.333 | 0.280 | 0.196 | 0.360 | 0.402 | 0.325 | 0.361 | 0.412 | 0.444 |
| 0.873 | 0.325 | 0.281 | 0.205 | 0.331 | 0.282 | 0.194 | 0.360 | 0.400 | 0.324 | 0.362 | 0.412 | 0.444 |
| 0.882 | 0.328 | 0.276 | 0.200 | 0.328 | 0.279 | 0.189 | 0.360 | 0.397 | 0.319 | 0.362 | 0.413 | 0.445 |
| 0.890 | 0.324 | 0.272 | 0.194 | 0.325 | 0.277 | 0.187 | 0.360 | 0.400 | 0.317 | 0.363 | 0.414 | 0.445 |
| 0.899 | 0.327 | 0.266 | 0.194 | 0.324 | 0.272 | 0.184 | 0.360 | 0.398 | 0.314 | 0.364 | 0.414 | 0.446 |
| 0.908 | 0.324 | 0.265 | 0.190 | 0.326 | 0.267 | 0.179 | 0.361 | 0.396 | 0.312 | 0.364 | 0.415 | 0.446 |
| 0.916 | 0.321 | 0.256 | 0.184 | 0.324 | 0.264 | 0.173 | 0.360 | 0.395 | 0.310 | 0.365 | 0.416 | 0.447 |
| 0.925 | 0.323 | 0.254 | 0.176 | 0.322 | 0.260 | 0.168 | 0.362 | 0.394 | 0.307 | 0.365 | 0.416 | 0.447 |
| 0.933 | 0.324 | 0.257 | 0.166 | 0.321 | 0.255 | 0.157 | 0.363 | 0.393 | 0.305 | 0.366 | 0.417 | 0.448 |
| 0.942 | 0.325 | 0.258 | 0.160 | 0.326 | 0.260 | 0.154 | 0.364 | 0.395 | 0.304 | 0.367 | 0.418 | 0.448 |
| 0.951 | 0.330 | 0.257 | 0.159 | 0.328 | 0.260 | 0.151 | 0.365 | 0.394 | 0.302 | 0.367 | 0.418 | 0.449 |
| 0.959 | 0.337 | 0.258 | 0.162 | 0.336 | 0.265 | 0.154 | 0.367 | 0.394 | 0.300 | 0.368 | 0.419 | 0.449 |
| 0.968 | 0.348 | 0.263 | 0.168 | 0.341 | 0.270 | 0.157 | 0.369 | 0.394 | 0.302 | 0.369 | 0.420 | 0.450 |
| 0.977 | 0.346 | 0.264 | 0.172 | 0.343 | 0.269 | 0.159 | 0.370 | 0.392 | 0.299 | 0.369 | 0.421 | 0.450 |
| 0.985 | 0.344 | 0.267 | 0.175 | 0.338 | 0.268 | 0.163 | 0.372 | 0.390 | 0.299 | 0.370 | 0.421 | 0.451 |
| 0.994 | 0.347 | 0.266 | 0.181 | 0.342 | 0.268 | 0.168 | 0.373 | 0.391 | 0.298 | 0.371 | 0.422 | 0.451 |
| 1.000 | 0.354 | 0.268 | 0.185 | 0.347 | 0.268 | 0.172 | 0.374 | 0.393 | 0.300 | 0.372 | 0.423 | 0.452 |
| 1.010 | 0.352 | 0.271 | 0.189 | 0.345 | 0.269 | 0.176 | 0.375 | 0.392 | 0.301 | 0.372 | 0.423 | 0.453 |
| 1.020 | 0.350 | 0.271 | 0.192 | 0.345 | 0.270 | 0.179 | 0.377 | 0.390 | 0.305 | 0.373 | 0.424 | 0.453 |
| 1.030 | 0.351 | 0.273 | 0.196 | 0.346 | 0.271 | 0.182 | 0.377 | 0.392 | 0.301 | 0.374 | 0.425 | 0.454 |
| 1.040 | 0.357 | 0.274 | 0.198 | 0.349 | 0.272 | 0.184 | 0.378 | 0.390 | 0.302 | 0.374 | 0.425 | 0.454 |
| 1.050 | 0.355 | 0.273 | 0.200 | 0.349 | 0.272 | 0.186 | 0.379 | 0.389 | 0.303 | 0.375 | 0.426 | 0.455 |
| 1.050 | 0.354 | 0.274 | 0.201 | 0.347 | 0.274 | 0.187 | 0.380 | 0.389 | 0.302 | 0.375 | 0.426 | 0.455 |
| 1.060 | 0.354 | 0.271 | 0.200 | 0.347 | 0.272 | 0.188 | 0.380 | 0.387 | 0.302 | 0.376 | 0.427 | 0.456 |
| 1.070 | 0.360 | 0.274 | 0.200 | 0.349 | 0.273 | 0.186 | 0.380 | 0.387 | 0.301 | 0.376 | 0.427 | 0.456 |
| 1.080 | 0.358 | 0.271 | 0.198 | 0.349 | 0.270 | 0.186 | 0.380 | 0.387 | 0.300 | 0.377 | 0.428 | 0.457 |
| 1.090 | 0.352 | 0.264 | 0.193 | 0.344 | 0.266 | 0.182 | 0.380 | 0.386 | 0.299 | 0.377 | 0.428 | 0.457 |
| 1.100 | 0.343 | 0.262 | 0.186 | 0.339 | 0.255 | 0.176 | 0.380 | 0.383 | 0.298 | 0.378 | 0.429 | 0.458 |
| 1.110 | 0.335 | 0.257 | 0.183 | 0.335 | 0.255 | 0.172 | 0.379 | 0.383 | 0.296 | 0.378 | 0.429 | 0.458 |
| 1.110 | 0.334 | 0.255 | 0.180 | 0.333 | 0.253 | 0.170 | 0.379 | 0.382 | 0.296 | 0.379 | 0.430 | 0.459 |
| 1.120 | 0.332 | 0.253 | 0.169 | 0.335 | 0.248 | 0.167 | 0.378 | 0.379 | 0.294 | 0.379 | 0.430 | 0.459 |
| 1.130 | 0.334 | 0.249 | 0.168 | 0.326 | 0.245 | 0.163 | 0.378 | 0.378 | 0.293 | 0.379 | 0.431 | 0.459 |
| 1.140 | 0.332 | 0.244 | 0.166 | 0.329 | 0.241 | 0.161 | 0.378 | 0.378 | 0.292 | 0.380 | 0.431 | 0.460 |
| 1.150 | 0.330 | 0.244 | 0.165 | 0.330 | 0.241 | 0.159 | 0.378 | 0.376 | 0.291 | 0.380 | 0.431 | 0.460 |
| 1.160 | 0.329 | 0.241 | 0.163 | 0.327 | 0.240 | 0.157 | 0.377 | 0.373 | 0.290 | 0.381 | 0.432 | 0.461 |
| 1.170 | 0.331 | 0.241 | 0.160 | 0.324 | 0.237 | 0.154 | 0.377 | 0.375 | 0.289 | 0.381 | 0.432 | 0.461 |
| 1.180 | 0.331 | 0.239 | 0.158 | 0.328 | 0.231 | 0.152 | 0.377 | 0.372 | 0.287 | 0.381 | 0.433 | 0.461 |
| 1.180 | 0.332 | 0.238 | 0.158 | 0.328 | 0.236 | 0.153 | 0.377 | 0.369 | 0.285 | 0.382 | 0.433 | 0.462 |
| 1.190 | 0.333 | 0.231 | 0.154 | 0.326 | 0.230 | 0.148 | 0.377 | 0.370 | 0.284 | 0.382 | 0.433 | 0.462 |
| 1.200 | 0.334 | 0.231 | 0.152 | 0.322 | 0.225 | 0.145 | 0.378 | 0.369 | 0.282 | 0.383 | 0.434 | 0.463 |
| 1.210 | 0.334 | 0.223 | 0.149 | 0.325 | 0.220 | 0.140 | 0.377 | 0.368 | 0.281 | 0.383 | 0.434 | 0.463 |
| 1.220 | 0.329 | 0.216 | 0.142 | 0.319 | 0.219 | 0.137 | 0.378 | 0.368 | 0.278 | 0.384 | 0.435 | 0.464 |
| 1.230 | 0.327 | 0.217 | 0.135 | 0.320 | 0.216 | 0.129 | 0.378 | 0.365 | 0.278 | 0.384 | 0.435 | 0.464 |
| 1.240 | 0.329 | 0.219 | 0.133 | 0.319 | 0.217 | 0.123 | 0.379 | 0.363 | 0.276 | 0.385 | 0.435 | 0.465 |
| 1.240 | 0.328 | 0.217 | 0.132 | 0.320 | 0.217 | 0.121 | 0.379 | 0.365 | 0.277 | 0.385 | 0.436 | 0.465 |
| 1.250 | 0.334 | 0.217 | 0.127 | 0.327 | 0.217 | 0.120 | 0.380 | 0.363 | 0.277 | 0.386 | 0.436 | 0.466 |
| 1.260 | 0.342 | 0.222 | 0.134 | 0.331 | 0.222 | 0.127 | 0.382 | 0.361 | 0.276 | 0.386 | 0.437 | 0.466 |
| 1.270 | 0.349 | 0.223 | 0.139 | 0.334 | 0.225 | 0.130 | 0.383 | 0.359 | 0.277 | 0.387 | 0.437 | 0.467 |
| 1.280 | 0.345 | 0.222 | 0.143 | 0.334 | 0.224 | 0.134 | 0.384 | 0.360 | 0.275 | 0.387 | 0.438 | 0.467 |
| 1.290 | 0.344 | 0.226 | 0.148 | 0.334 | 0.224 | 0.140 | 0.385 | 0.362 | 0.276 | 0.388 | 0.438 | 0.468 |
| 1.300 | 0.348 | 0.226 | 0.155 | 0.335 | 0.226 | 0.146 | 0.386 | 0.362 | 0.276 | 0.388 | 0.439 | 0.468 |
| 1.310 | 0.350 | 0.229 | 0.160 | 0.337 | 0.226 | 0.153 | 0.387 | 0.362 | 0.275 | 0.389 | 0.439 | 0.469 |
| 1.310 | 0.348 | 0.231 | 0.166 | 0.334 | 0.229 | 0.157 | 0.388 | 0.361 | 0.273 | 0.389 | 0.440 | 0.469 |
| 1.320 | 0.347 | 0.230 | 0.169 | 0.335 | 0.229 | 0.161 | 0.387 | 0.361 | 0.273 | 0.390 | 0.440 | 0.470 |
| 1.330 | 0.352 | 0.234 | 0.172 | 0.337 | 0.231 | 0.163 | 0.388 | 0.360 | 0.274 | 0.390 | 0.440 | 0.470 |
| 1.340 | 0.356 | 0.232 | 0.176 | 0.339 | 0.231 | 0.166 | 0.388 | 0.362 | 0.274 | 0.391 | 0.441 | 0.470 |
| 1.350 | 0.354 | 0.235 | 0.178 | 0.337 | 0.233 | 0.167 | 0.390 | 0.358 | 0.274 | 0.391 | 0.441 | 0.471 |
| 1.360 | 0.348 | 0.236 | 0.180 | 0.335 | 0.233 | 0.171 | 0.391 | 0.356 | 0.271 | 0.391 | 0.441 | 0.471 |
| 1.370 | 0.354 | 0.234 | 0.180 | 0.340 | 0.233 | 0.172 | 0.390 | 0.359 | 0.271 | 0.392 | 0.442 | 0.471 |
| 1.370 | 0.357 | 0.236 | 0.180 | 0.339 | 0.232 | 0.170 | 0.390 | 0.357 | 0.271 | 0.392 | 0.442 | 0.472 |
| 1.380 | 0.353 | 0.232 | 0.176 | 0.338 | 0.227 | 0.165 | 0.388 | 0.360 | 0.271 | 0.392 | 0.442 | 0.472 |
| 1.390 | 0.347 | 0.229 | 0.168 | 0.335 | 0.222 | 0.159 | 0.389 | 0.360 | 0.270 | 0.392 | 0.443 | 0.472 |
| 1.400 | 0.336 | 0.222 | 0.161 | 0.331 | 0.210 | 0.153 | 0.390 | 0.360 | 0.267 | 0.392 | 0.443 | 0.472 |
| 1.410 | 0.336 | 0.209 | 0.155 | 0.329 | 0.207 | 0.153 | 0.389 | 0.358 | 0.266 | 0.393 | 0.443 | 0.472 |
| 1.420 | 0.337 | 0.207 | 0.147 | 0.329 | 0.206 | 0.144 | 0.388 | 0.356 | 0.265 | 0.393 | 0.443 | 0.472 |
| 1.430 | 0.339 | 0.206 | 0.143 | 0.327 | 0.202 | 0.145 | 0.387 | 0.358 | 0.265 | 0.393 | 0.443 | 0.472 |
| 1.430 | 0.337 | 0.204 | 0.140 | 0.326 | 0.200 | 0.146 | 0.387 | 0.356 | 0.263 | 0.393 | 0.443 | 0.472 |
| 1.440 | 0.335 | 0.203 | 0.137 | 0.324 | 0.198 | 0.138 | 0.387 | 0.355 | 0.261 | 0.393 | 0.443 | 0.472 |
| 1.450 | 0.336 | 0.198 | 0.136 | 0.324 | 0.198 | 0.133 | 0.387 | 0.354 | 0.261 | 0.393 | 0.443 | 0.472 |
| 1.460 | 0.334 | 0.197 | 0.132 | 0.324 | 0.201 | 0.130 | 0.387 | 0.354 | 0.258 | 0.393 | 0.443 | 0.472 |
| 1.470 | 0.335 | 0.198 | 0.130 | 0.323 | 0.200 | 0.127 | 0.386 | 0.355 | 0.258 | 0.393 | 0.443 | 0.472 |
| 1.480 | 0.332 | 0.196 | 0.126 | 0.322 | 0.199 | 0.119 | 0.386 | 0.352 | 0.257 | 0.393 | 0.443 | 0.471 |
| 1.490 | 0.332 | 0.193 | 0.120 | 0.319 | 0.197 | 0.113 | 0.386 | 0.353 | 0.257 | 0.393 | 0.443 | 0.471 |
| 1.500 | 0.334 | 0.193 | 0.118 | 0.318 | 0.195 | 0.111 | 0.386 | 0.352 | 0.254 | 0.393 | 0.443 | 0.471 |
| 1.500 | 0.332 | 0.191 | 0.116 | 0.317 | 0.193 | 0.110 | 0.386 | 0.353 | 0.251 | 0.394 | 0.443 | 0.471 |
| 1.510 | 0.331 | 0.185 | 0.105 | 0.316 | 0.186 | 0.099 | 0.384 | 0.353 | 0.252 | 0.394 | 0.443 | 0.471 |
| 1.520 | 0.329 | 0.184 | 0.097 | 0.315 | 0.188 | 0.093 | 0.384 | 0.351 | 0.250 | 0.394 | 0.443 | 0.470 |
| 1.530 | 0.328 | 0.184 | 0.091 | 0.315 | 0.186 | 0.086 | 0.384 | 0.351 | 0.249 | 0.394 | 0.443 | 0.470 |
| 1.540 | 0.328 | 0.182 | 0.089 | 0.316 | 0.183 | 0.083 | 0.384 | 0.351 | 0.248 | 0.394 | 0.443 | 0.470 |
| 1.550 | 0.327 | 0.183 | 0.085 | 0.315 | 0.182 | 0.077 | 0.384 | 0.349 | 0.247 | 0.394 | 0.443 | 0.469 |
| 1.560 | 0.329 | 0.180 | 0.079 | 0.312 | 0.179 | 0.072 | 0.385 | 0.349 | 0.245 | 0.394 | 0.443 | 0.469 |
| 1.560 | 0.329 | 0.179 | 0.074 | 0.313 | 0.178 | 0.068 | 0.384 | 0.349 | 0.245 | 0.394 | 0.442 | 0.469 |
| 1.570 | 0.327 | 0.176 | 0.068 | 0.312 | 0.177 | 0.063 | 0.385 | 0.349 | 0.243 | 0.394 | 0.442 | 0.468 |
| 1.580 | 0.324 | 0.174 | 0.064 | 0.309 | 0.173 | 0.059 | 0.383 | 0.349 | 0.243 | 0.394 | 0.442 | 0.468 |
| 1.590 | 0.324 | 0.170 | 0.059 | 0.309 | 0.169 | 0.055 | 0.384 | 0.349 | 0.242 | 0.394 | 0.442 | 0.467 |
| 1.600 | 0.325 | 0.168 | 0.055 | 0.308 | 0.167 | 0.050 | 0.383 | 0.347 | 0.241 | 0.394 | 0.442 | 0.467 |
| 1.610 | 0.325 | 0.168 | 0.052 | 0.307 | 0.166 | 0.048 | 0.383 | 0.347 | 0.239 | 0.394 | 0.442 | 0.467 |
| 1.620 | 0.324 | 0.165 | 0.049 | 0.308 | 0.163 | 0.045 | 0.383 | 0.347 | 0.238 | 0.394 | 0.442 | 0.466 |
| 1.620 | 0.324 | 0.164 | 0.047 | 0.307 | 0.161 | 0.042 | 0.383 | 0.346 | 0.237 | 0.394 | 0.441 | 0.466 |
| 1.630 | 0.323 | 0.162 | 0.045 | 0.307 | 0.159 | 0.041 | 0.382 | 0.346 | 0.235 | 0.394 | 0.441 | 0.466 |
| 1.640 | 0.321 | 0.160 | 0.044 | 0.306 | 0.157 | 0.040 | 0.383 | 0.346 | 0.235 | 0.394 | 0.441 | 0.465 |
| 1.650 | 0.322 | 0.157 | 0.043 | 0.305 | 0.156 | 0.039 | 0.383 | 0.343 | 0.235 | 0.394 | 0.441 | 0.465 |
| 1.660 | 0.321 | 0.154 | 0.043 | 0.306 | 0.153 | 0.039 | 0.384 | 0.341 | 0.234 | 0.394 | 0.441 | 0.465 |
| 1.670 | 0.322 | 0.151 | 0.044 | 0.305 | 0.149 | 0.039 | 0.383 | 0.341 | 0.232 | 0.394 | 0.441 | 0.465 |
| 1.680 | 0.321 | 0.149 | 0.045 | 0.304 | 0.148 | 0.041 | 0.382 | 0.342 | 0.230 | 0.394 | 0.440 | 0.464 |
| 1.690 | 0.318 | 0.145 | 0.046 | 0.302 | 0.146 | 0.043 | 0.382 | 0.342 | 0.230 | 0.394 | 0.440 | 0.464 |
| 1.690 | 0.319 | 0.143 | 0.048 | 0.301 | 0.144 | 0.045 | 0.382 | 0.341 | 0.229 | 0.394 | 0.440 | 0.464 |
| 1.700 | 0.319 | 0.142 | 0.051 | 0.299 | 0.139 | 0.048 | 0.382 | 0.341 | 0.228 | 0.394 | 0.440 | 0.464 |
| 1.710 | 0.315 | 0.139 | 0.055 | 0.300 | 0.137 | 0.052 | 0.381 | 0.339 | 0.225 | 0.394 | 0.440 | 0.464 |
| 1.720 | 0.314 | 0.135 | 0.058 | 0.299 | 0.135 | 0.056 | 0.382 | 0.341 | 0.223 | 0.394 | 0.440 | 0.464 |

**Table. 5** In the non-submerged state (*h*=0.05 m), the streamwise velocity *u* of four different fragmentation conditions changes along the *x* direction.

| **Hydraulic parameters** | **Streamwise velocity u (m/s)** | | | | | | | | | | | | | | | |
| --- | --- | --- | --- | --- | --- | --- | --- | --- | --- | --- | --- | --- | --- | --- | --- | --- |
| ***x* (m)** | **L_1-1_** | | | | **L_2-2_** | | | | **L_3-3_** | | | | **L_4-4_** | | | |
|  | **Fragmentation I** | **Fragmentation II** | **Fragmentation III** | **Fragmentation IV** | **Fragmentation I** | **Fragmentation II** | **Fragmentation III** | **Fragmentation IV** | **Fragmentation I** | **Fragmentation II** | **Fragmentation III** | **Fragmentation IV** | **Fragmentation I** | **Fragmentation II** | **Fragmentation III** | **Fragmentation IV** |
| 0.000 | 0.300 | 0.300 | 0.300 | 0.300 | 0.300 | 0.300 | 0.300 | 0.300 | 0.300 | 0.300 | 0.300 | 0.300 | 0.300 | 0.300 | 0.300 | 0.300 |
| 0.009 | 0.300 | 0.300 | 0.300 | 0.300 | 0.300 | 0.300 | 0.300 | 0.300 | 0.300 | 0.300 | 0.300 | 0.300 | 0.300 | 0.300 | 0.300 | 0.300 |
| 0.017 | 0.300 | 0.300 | 0.301 | 0.300 | 0.300 | 0.300 | 0.301 | 0.300 | 0.300 | 0.300 | 0.301 | 0.300 | 0.300 | 0.300 | 0.301 | 0.300 |
| 0.026 | 0.300 | 0.300 | 0.301 | 0.300 | 0.300 | 0.300 | 0.301 | 0.300 | 0.300 | 0.300 | 0.301 | 0.300 | 0.301 | 0.301 | 0.302 | 0.301 |
| 0.035 | 0.300 | 0.300 | 0.301 | 0.300 | 0.300 | 0.300 | 0.301 | 0.300 | 0.301 | 0.301 | 0.302 | 0.300 | 0.301 | 0.301 | 0.302 | 0.301 |
| 0.043 | 0.300 | 0.300 | 0.301 | 0.300 | 0.300 | 0.300 | 0.301 | 0.300 | 0.301 | 0.301 | 0.302 | 0.301 | 0.301 | 0.302 | 0.303 | 0.302 |
| 0.052 | 0.300 | 0.300 | 0.301 | 0.300 | 0.300 | 0.300 | 0.301 | 0.300 | 0.301 | 0.301 | 0.302 | 0.301 | 0.302 | 0.303 | 0.303 | 0.302 |
| 0.061 | 0.300 | 0.300 | 0.301 | 0.299 | 0.300 | 0.300 | 0.301 | 0.299 | 0.301 | 0.301 | 0.303 | 0.301 | 0.302 | 0.303 | 0.304 | 0.303 |
| 0.069 | 0.300 | 0.300 | 0.301 | 0.299 | 0.300 | 0.300 | 0.301 | 0.299 | 0.301 | 0.302 | 0.303 | 0.301 | 0.303 | 0.304 | 0.305 | 0.304 |
| 0.078 | 0.300 | 0.299 | 0.301 | 0.299 | 0.300 | 0.299 | 0.301 | 0.299 | 0.302 | 0.302 | 0.303 | 0.302 | 0.304 | 0.305 | 0.306 | 0.305 |
| 0.086 | 0.300 | 0.299 | 0.300 | 0.298 | 0.300 | 0.299 | 0.300 | 0.298 | 0.302 | 0.302 | 0.303 | 0.302 | 0.304 | 0.306 | 0.306 | 0.306 |
| 0.095 | 0.299 | 0.299 | 0.300 | 0.298 | 0.299 | 0.298 | 0.300 | 0.298 | 0.302 | 0.303 | 0.303 | 0.302 | 0.305 | 0.307 | 0.307 | 0.307 |
| 0.104 | 0.299 | 0.298 | 0.299 | 0.297 | 0.299 | 0.298 | 0.299 | 0.297 | 0.302 | 0.303 | 0.304 | 0.302 | 0.306 | 0.309 | 0.308 | 0.308 |
| 0.112 | 0.299 | 0.297 | 0.299 | 0.296 | 0.299 | 0.297 | 0.299 | 0.297 | 0.303 | 0.303 | 0.304 | 0.303 | 0.307 | 0.310 | 0.309 | 0.309 |
| 0.121 | 0.298 | 0.297 | 0.298 | 0.295 | 0.298 | 0.297 | 0.298 | 0.296 | 0.303 | 0.304 | 0.304 | 0.303 | 0.308 | 0.311 | 0.310 | 0.311 |
| 0.130 | 0.298 | 0.296 | 0.297 | 0.294 | 0.298 | 0.296 | 0.297 | 0.295 | 0.303 | 0.304 | 0.304 | 0.303 | 0.309 | 0.313 | 0.312 | 0.312 |
| 0.138 | 0.297 | 0.295 | 0.296 | 0.293 | 0.297 | 0.295 | 0.296 | 0.294 | 0.304 | 0.304 | 0.304 | 0.303 | 0.310 | 0.314 | 0.313 | 0.314 |
| 0.147 | 0.296 | 0.294 | 0.295 | 0.292 | 0.297 | 0.294 | 0.295 | 0.293 | 0.304 | 0.305 | 0.304 | 0.304 | 0.311 | 0.316 | 0.314 | 0.315 |
| 0.156 | 0.296 | 0.293 | 0.294 | 0.290 | 0.296 | 0.293 | 0.294 | 0.291 | 0.304 | 0.305 | 0.305 | 0.304 | 0.312 | 0.317 | 0.316 | 0.317 |
| 0.164 | 0.295 | 0.292 | 0.293 | 0.289 | 0.295 | 0.292 | 0.292 | 0.290 | 0.304 | 0.306 | 0.305 | 0.304 | 0.313 | 0.319 | 0.317 | 0.319 |
| 0.173 | 0.294 | 0.290 | 0.291 | 0.287 | 0.294 | 0.290 | 0.291 | 0.288 | 0.305 | 0.306 | 0.305 | 0.304 | 0.315 | 0.321 | 0.319 | 0.322 |
| 0.182 | 0.293 | 0.289 | 0.290 | 0.285 | 0.293 | 0.289 | 0.289 | 0.286 | 0.305 | 0.307 | 0.305 | 0.305 | 0.316 | 0.323 | 0.321 | 0.324 |
| 0.190 | 0.291 | 0.287 | 0.288 | 0.283 | 0.292 | 0.287 | 0.288 | 0.284 | 0.305 | 0.307 | 0.305 | 0.305 | 0.318 | 0.325 | 0.323 | 0.326 |
| 0.199 | 0.290 | 0.286 | 0.286 | 0.281 | 0.291 | 0.286 | 0.286 | 0.282 | 0.306 | 0.308 | 0.305 | 0.305 | 0.320 | 0.328 | 0.325 | 0.329 |
| 0.207 | 0.288 | 0.284 | 0.284 | 0.278 | 0.289 | 0.284 | 0.284 | 0.280 | 0.306 | 0.308 | 0.305 | 0.305 | 0.321 | 0.330 | 0.328 | 0.332 |
| 0.216 | 0.287 | 0.282 | 0.282 | 0.276 | 0.287 | 0.282 | 0.282 | 0.277 | 0.307 | 0.309 | 0.304 | 0.305 | 0.323 | 0.333 | 0.330 | 0.335 |
| 0.225 | 0.285 | 0.280 | 0.280 | 0.273 | 0.285 | 0.280 | 0.280 | 0.274 | 0.307 | 0.310 | 0.304 | 0.305 | 0.325 | 0.335 | 0.333 | 0.339 |
| 0.233 | 0.282 | 0.277 | 0.278 | 0.269 | 0.283 | 0.277 | 0.277 | 0.270 | 0.308 | 0.311 | 0.304 | 0.305 | 0.328 | 0.338 | 0.336 | 0.342 |
| 0.242 | 0.280 | 0.275 | 0.275 | 0.266 | 0.281 | 0.274 | 0.275 | 0.267 | 0.308 | 0.312 | 0.304 | 0.305 | 0.330 | 0.341 | 0.339 | 0.346 |
| 0.251 | 0.277 | 0.272 | 0.273 | 0.261 | 0.278 | 0.271 | 0.272 | 0.262 | 0.309 | 0.314 | 0.304 | 0.305 | 0.332 | 0.344 | 0.342 | 0.350 |
| 0.259 | 0.274 | 0.268 | 0.270 | 0.256 | 0.274 | 0.267 | 0.269 | 0.258 | 0.310 | 0.315 | 0.305 | 0.305 | 0.335 | 0.347 | 0.346 | 0.355 |
| 0.268 | 0.270 | 0.264 | 0.266 | 0.249 | 0.271 | 0.262 | 0.265 | 0.256 | 0.311 | 0.318 | 0.306 | 0.307 | 0.338 | 0.350 | 0.350 | 0.360 |
| 0.277 | 0.265 | 0.257 | 0.259 | 0.224 | 0.266 | 0.255 | 0.257 | 0.268 | 0.312 | 0.322 | 0.311 | 0.317 | 0.341 | 0.354 | 0.354 | 0.364 |
| 0.285 | 0.260 | 0.249 | 0.240 | 0.000 | 0.261 | 0.246 | 0.238 | 0.348 | 0.314 | 0.326 | 0.323 | 0.358 | 0.344 | 0.357 | 0.358 | 0.369 |
| 0.294 | 0.254 | 0.236 | 0.141 | 0.015 | 0.254 | 0.232 | 0.273 | 0.405 | 0.317 | 0.333 | 0.348 | 0.352 | 0.348 | 0.361 | 0.362 | 0.374 |
| 0.303 | 0.247 | 0.217 | 0.102 | 0.148 | 0.247 | 0.212 | 0.447 | 0.391 | 0.319 | 0.343 | 0.377 | 0.265 | 0.352 | 0.364 | 0.367 | 0.380 |
| 0.311 | 0.239 | 0.187 | 0.000 | 0.199 | 0.238 | 0.200 | 0.355 | 0.361 | 0.323 | 0.355 | 0.390 | 0.200 | 0.355 | 0.368 | 0.371 | 0.385 |
| 0.320 | 0.228 | 0.000 | 0.035 | 0.143 | 0.228 | 0.362 | 0.342 | 0.349 | 0.328 | 0.369 | 0.363 | 0.220 | 0.360 | 0.372 | 0.376 | 0.390 |
| 0.328 | 0.216 | -0.031 | 0.198 | 0.000 | 0.215 | 0.383 | 0.258 | 0.388 | 0.334 | 0.383 | 0.308 | 0.331 | 0.364 | 0.376 | 0.381 | 0.396 |
| 0.337 | 0.203 | 0.000 | 0.186 | 0.148 | 0.201 | 0.418 | 0.205 | 0.357 | 0.341 | 0.396 | 0.232 | 0.273 | 0.368 | 0.380 | 0.385 | 0.401 |
| 0.346 | 0.185 | 0.026 | 0.181 | 0.210 | 0.183 | 0.291 | 0.166 | 0.298 | 0.350 | 0.407 | 0.170 | 0.216 | 0.373 | 0.384 | 0.390 | 0.406 |
| 0.354 | 0.164 | 0.000 | 0.160 | 0.202 | 0.172 | 0.352 | 0.140 | 0.268 | 0.361 | 0.418 | 0.134 | 0.203 | 0.378 | 0.387 | 0.394 | 0.412 |
| 0.363 | 0.000 | 0.000 | 0.134 | 0.000 | 0.263 | 0.310 | 0.121 | 0.318 | 0.373 | 0.428 | 0.128 | 0.272 | 0.383 | 0.391 | 0.399 | 0.417 |
| 0.372 | 0.000 | 0.000 | 0.000 | 0.115 | 0.364 | 0.293 | 0.234 | 0.306 | 0.386 | 0.435 | 0.172 | 0.261 | 0.388 | 0.395 | 0.403 | 0.422 |
| 0.380 | 0.000 | 0.078 | 0.036 | 0.194 | 0.376 | 0.186 | 0.279 | 0.253 | 0.399 | 0.438 | 0.225 | 0.215 | 0.393 | 0.399 | 0.408 | 0.427 |
| 0.389 | 0.007 | 0.116 | 0.000 | 0.221 | 0.321 | 0.146 | 0.286 | 0.223 | 0.412 | 0.437 | 0.252 | 0.185 | 0.398 | 0.403 | 0.412 | 0.432 |
| 0.398 | 0.000 | 0.123 | 0.122 | 0.000 | 0.353 | 0.141 | 0.166 | 0.254 | 0.424 | 0.431 | 0.261 | 0.213 | 0.403 | 0.407 | 0.416 | 0.437 |
| 0.406 | 0.035 | 0.122 | 0.100 | 0.001 | 0.265 | 0.135 | 0.087 | 0.305 | 0.435 | 0.420 | 0.244 | 0.243 | 0.408 | 0.411 | 0.420 | 0.442 |
| 0.415 | 0.000 | 0.119 | 0.076 | 0.125 | 0.293 | 0.129 | 0.072 | 0.279 | 0.445 | 0.404 | 0.207 | 0.202 | 0.413 | 0.414 | 0.424 | 0.447 |
| 0.424 | 0.000 | 0.115 | 0.074 | 0.174 | 0.248 | 0.122 | 0.094 | 0.242 | 0.452 | 0.383 | 0.177 | 0.176 | 0.417 | 0.418 | 0.428 | 0.452 |
| 0.432 | 0.000 | 0.110 | 0.069 | 0.143 | 0.235 | 0.116 | 0.093 | 0.243 | 0.455 | 0.359 | 0.154 | 0.188 | 0.422 | 0.422 | 0.432 | 0.457 |
| 0.441 | 0.000 | 0.104 | 0.065 | 0.000 | 0.261 | 0.108 | 0.108 | 0.282 | 0.455 | 0.332 | 0.141 | 0.225 | 0.427 | 0.425 | 0.436 | 0.461 |
| 0.449 | 0.008 | 0.096 | 0.000 | 0.116 | 0.218 | 0.099 | 0.255 | 0.250 | 0.452 | 0.306 | 0.177 | 0.209 | 0.431 | 0.429 | 0.440 | 0.466 |
| 0.458 | 0.000 | 0.086 | 0.078 | 0.181 | 0.254 | 0.089 | 0.178 | 0.208 | 0.446 | 0.283 | 0.212 | 0.178 | 0.435 | 0.432 | 0.444 | 0.470 |
| 0.467 | 0.018 | 0.038 | 0.000 | 0.160 | 0.182 | 0.121 | 0.236 | 0.201 | 0.436 | 0.264 | 0.231 | 0.168 | 0.439 | 0.436 | 0.447 | 0.475 |
| 0.475 | 0.000 | 0.000 | 0.076 | 0.000 | 0.206 | 0.207 | 0.138 | 0.264 | 0.425 | 0.251 | 0.231 | 0.210 | 0.443 | 0.439 | 0.451 | 0.479 |
| 0.484 | 0.077 | 0.000 | 0.097 | 0.041 | 0.129 | 0.163 | 0.100 | 0.271 | 0.414 | 0.243 | 0.216 | 0.207 | 0.447 | 0.442 | 0.454 | 0.483 |
| 0.493 | 0.114 | 0.003 | 0.084 | 0.141 | 0.107 | 0.160 | 0.105 | 0.241 | 0.400 | 0.240 | 0.194 | 0.174 | 0.450 | 0.445 | 0.457 | 0.487 |
| 0.501 | 0.116 | 0.000 | 0.087 | 0.146 | 0.105 | 0.166 | 0.116 | 0.204 | 0.388 | 0.240 | 0.175 | 0.155 | 0.453 | 0.448 | 0.460 | 0.491 |
| 0.510 | 0.115 | 0.000 | 0.090 | 0.000 | 0.107 | 0.125 | 0.133 | 0.237 | 0.373 | 0.241 | 0.159 | 0.181 | 0.457 | 0.451 | 0.464 | 0.495 |
| 0.519 | 0.114 | 0.000 | 0.054 | -0.032 | 0.109 | 0.150 | 0.160 | 0.268 | 0.360 | 0.242 | 0.166 | 0.206 | 0.459 | 0.454 | 0.467 | 0.499 |
| 0.527 | 0.112 | 0.014 | 0.000 | 0.072 | 0.108 | 0.097 | 0.298 | 0.229 | 0.347 | 0.243 | 0.186 | 0.182 | 0.462 | 0.457 | 0.470 | 0.502 |
| 0.536 | 0.112 | 0.052 | 0.000 | 0.134 | 0.106 | 0.060 | 0.211 | 0.204 | 0.335 | 0.243 | 0.201 | 0.160 | 0.465 | 0.460 | 0.472 | 0.506 |
| 0.545 | 0.111 | 0.066 | 0.004 | 0.108 | 0.107 | 0.043 | 0.230 | 0.215 | 0.323 | 0.241 | 0.208 | 0.163 | 0.467 | 0.462 | 0.475 | 0.510 |
| 0.553 | 0.109 | 0.065 | 0.095 | 0.000 | 0.107 | 0.035 | 0.140 | 0.257 | 0.313 | 0.239 | 0.201 | 0.196 | 0.470 | 0.465 | 0.478 | 0.513 |
| 0.562 | 0.107 | 0.061 | 0.099 | 0.094 | 0.106 | 0.030 | 0.106 | 0.223 | 0.302 | 0.237 | 0.187 | 0.182 | 0.472 | 0.468 | 0.481 | 0.517 |
| 0.570 | 0.104 | 0.057 | 0.088 | 0.136 | 0.105 | 0.025 | 0.103 | 0.193 | 0.293 | 0.234 | 0.175 | 0.163 | 0.474 | 0.470 | 0.483 | 0.520 |
| 0.579 | 0.101 | 0.051 | 0.085 | 0.128 | 0.103 | 0.020 | 0.103 | 0.195 | 0.284 | 0.230 | 0.165 | 0.153 | 0.476 | 0.472 | 0.486 | 0.523 |
| 0.588 | 0.098 | 0.046 | 0.078 | 0.000 | 0.100 | 0.014 | 0.106 | 0.239 | 0.276 | 0.227 | 0.163 | 0.178 | 0.478 | 0.475 | 0.489 | 0.526 |
| 0.596 | 0.095 | 0.042 | 0.000 | 0.063 | 0.097 | 0.009 | 0.210 | 0.233 | 0.268 | 0.223 | 0.177 | 0.172 | 0.480 | 0.477 | 0.491 | 0.530 |
| 0.605 | 0.090 | 0.041 | 0.043 | 0.116 | 0.092 | 0.010 | 0.207 | 0.197 | 0.261 | 0.220 | 0.196 | 0.155 | 0.482 | 0.479 | 0.493 | 0.533 |
| 0.614 | 0.082 | 0.038 | 0.000 | 0.123 | 0.089 | 0.028 | 0.228 | 0.179 | 0.253 | 0.217 | 0.206 | 0.148 | 0.484 | 0.481 | 0.496 | 0.536 |
| 0.622 | 0.075 | 0.000 | -0.006 | 0.000 | 0.083 | 0.091 | 0.155 | 0.208 | 0.246 | 0.216 | 0.205 | 0.171 | 0.485 | 0.483 | 0.498 | 0.539 |
| 0.631 | 0.067 | 0.054 | 0.026 | 0.018 | 0.076 | 0.045 | 0.064 | 0.229 | 0.239 | 0.215 | 0.195 | 0.191 | 0.487 | 0.485 | 0.501 | 0.542 |
| 0.640 | 0.058 | 0.000 | 0.039 | 0.111 | 0.067 | 0.103 | 0.039 | 0.198 | 0.233 | 0.214 | 0.183 | 0.172 | 0.489 | 0.487 | 0.503 | 0.545 |
| 0.648 | 0.047 | 0.000 | 0.053 | 0.129 | 0.058 | 0.074 | 0.049 | 0.169 | 0.229 | 0.214 | 0.172 | 0.154 | 0.490 | 0.489 | 0.505 | 0.547 |
| 0.657 | 0.036 | 0.000 | 0.062 | 0.094 | 0.050 | 0.091 | 0.064 | 0.181 | 0.226 | 0.215 | 0.165 | 0.150 | 0.492 | 0.491 | 0.507 | 0.550 |
| 0.666 | 0.000 | 0.000 | 0.053 | 0.000 | 0.096 | 0.089 | 0.080 | 0.219 | 0.224 | 0.216 | 0.164 | 0.171 | 0.493 | 0.493 | 0.509 | 0.553 |
| 0.674 | 0.015 | -0.009 | 0.000 | 0.092 | 0.071 | 0.070 | 0.223 | 0.199 | 0.224 | 0.216 | 0.174 | 0.160 | 0.495 | 0.495 | 0.511 | 0.555 |
| 0.683 | 0.000 | -0.005 | 0.078 | 0.127 | 0.096 | 0.036 | 0.152 | 0.172 | 0.225 | 0.216 | 0.189 | 0.139 | 0.496 | 0.496 | 0.513 | 0.558 |
| 0.691 | 0.022 | 0.001 | 0.000 | 0.113 | 0.055 | 0.021 | 0.198 | 0.158 | 0.225 | 0.216 | 0.198 | 0.133 | 0.497 | 0.498 | 0.515 | 0.561 |
| 0.700 | 0.000 | 0.005 | 0.077 | 0.000 | 0.087 | 0.016 | 0.128 | 0.197 | 0.224 | 0.215 | 0.195 | 0.164 | 0.499 | 0.499 | 0.517 | 0.563 |
| 0.709 | 0.000 | 0.010 | 0.094 | 0.029 | 0.064 | 0.011 | 0.084 | 0.199 | 0.228 | 0.214 | 0.191 | 0.165 | 0.500 | 0.501 | 0.519 | 0.566 |
| 0.717 | 0.000 | 0.013 | 0.091 | 0.108 | 0.075 | 0.009 | 0.083 | 0.174 | 0.230 | 0.212 | 0.184 | 0.148 | 0.501 | 0.502 | 0.520 | 0.568 |
| 0.726 | 0.000 | 0.015 | 0.088 | 0.118 | 0.073 | 0.006 | 0.085 | 0.156 | 0.231 | 0.210 | 0.176 | 0.134 | 0.503 | 0.504 | 0.522 | 0.570 |
| 0.735 | 0.011 | 0.016 | 0.081 | 0.000 | 0.053 | 0.003 | 0.088 | 0.182 | 0.232 | 0.207 | 0.173 | 0.149 | 0.504 | 0.505 | 0.524 | 0.573 |
| 0.743 | 0.000 | 0.017 | 0.054 | -0.001 | 0.077 | 0.002 | 0.130 | 0.205 | 0.234 | 0.204 | 0.171 | 0.166 | 0.505 | 0.507 | 0.525 | 0.575 |
| 0.752 | 0.007 | 0.023 | 0.000 | 0.069 | 0.057 | 0.002 | 0.236 | 0.188 | 0.235 | 0.201 | 0.179 | 0.152 | 0.506 | 0.508 | 0.527 | 0.577 |
| 0.761 | 0.000 | 0.025 | 0.001 | 0.100 | 0.073 | 0.011 | 0.192 | 0.165 | 0.237 | 0.200 | 0.185 | 0.139 | 0.507 | 0.509 | 0.528 | 0.579 |
| 0.769 | 0.000 | 0.000 | 0.000 | 0.087 | 0.065 | 0.058 | 0.170 | 0.171 | 0.238 | 0.200 | 0.190 | 0.137 | 0.508 | 0.511 | 0.530 | 0.581 |
| 0.778 | -0.004 | 0.019 | 0.089 | 0.000 | 0.058 | 0.043 | 0.107 | 0.199 | 0.238 | 0.202 | 0.188 | 0.157 | 0.508 | 0.512 | 0.531 | 0.583 |
| 0.787 | 0.020 | 0.000 | 0.100 | 0.045 | 0.035 | 0.086 | 0.082 | 0.185 | 0.239 | 0.203 | 0.177 | 0.154 | 0.509 | 0.513 | 0.532 | 0.585 |
| 0.795 | 0.026 | 0.028 | 0.096 | 0.093 | 0.025 | 0.073 | 0.079 | 0.162 | 0.239 | 0.206 | 0.168 | 0.140 | 0.510 | 0.514 | 0.533 | 0.587 |
| 0.804 | 0.025 | 0.000 | 0.097 | 0.095 | 0.023 | 0.115 | 0.087 | 0.161 | 0.240 | 0.209 | 0.161 | 0.134 | 0.511 | 0.516 | 0.535 | 0.589 |
| 0.812 | 0.023 | 0.000 | 0.088 | 0.000 | 0.022 | 0.091 | 0.086 | 0.195 | 0.240 | 0.211 | 0.156 | 0.152 | 0.511 | 0.517 | 0.536 | 0.591 |
| 0.821 | 0.021 | 0.000 | 0.000 | 0.060 | 0.020 | 0.110 | 0.168 | 0.190 | 0.241 | 0.213 | 0.160 | 0.150 | 0.512 | 0.518 | 0.537 | 0.593 |
| 0.830 | 0.020 | 0.014 | 0.026 | 0.104 | 0.018 | 0.074 | 0.188 | 0.165 | 0.241 | 0.213 | 0.169 | 0.139 | 0.512 | 0.519 | 0.538 | 0.595 |
| 0.838 | 0.018 | 0.030 | 0.000 | 0.105 | 0.016 | 0.055 | 0.200 | 0.153 | 0.241 | 0.211 | 0.175 | 0.133 | 0.513 | 0.520 | 0.539 | 0.597 |
| 0.847 | 0.017 | 0.033 | 0.019 | 0.000 | 0.015 | 0.046 | 0.155 | 0.174 | 0.242 | 0.208 | 0.179 | 0.142 | 0.514 | 0.521 | 0.540 | 0.598 |
| 0.856 | 0.015 | 0.031 | 0.065 | 0.005 | 0.014 | 0.037 | 0.096 | 0.189 | 0.242 | 0.203 | 0.178 | 0.152 | 0.514 | 0.522 | 0.541 | 0.600 |
| 0.864 | 0.014 | 0.029 | 0.061 | 0.080 | 0.013 | 0.028 | 0.069 | 0.169 | 0.242 | 0.199 | 0.174 | 0.143 | 0.514 | 0.523 | 0.542 | 0.602 |
| 0.873 | 0.013 | 0.023 | 0.056 | 0.097 | 0.012 | 0.017 | 0.053 | 0.151 | 0.242 | 0.196 | 0.167 | 0.132 | 0.515 | 0.524 | 0.543 | 0.603 |
| 0.882 | 0.012 | 0.017 | 0.052 | 0.071 | 0.011 | 0.006 | 0.046 | 0.158 | 0.242 | 0.195 | 0.161 | 0.132 | 0.515 | 0.525 | 0.544 | 0.605 |
| 0.890 | 0.012 | 0.013 | 0.047 | 0.000 | 0.010 | 0.000 | 0.063 | 0.185 | 0.242 | 0.197 | 0.162 | 0.149 | 0.516 | 0.526 | 0.545 | 0.607 |
| 0.899 | 0.011 | 0.012 | 0.000 | 0.080 | 0.009 | -0.003 | 0.166 | 0.166 | 0.242 | 0.202 | 0.166 | 0.141 | 0.516 | 0.527 | 0.546 | 0.608 |
| 0.908 | 0.009 | 0.016 | 0.048 | 0.108 | 0.007 | 0.002 | 0.125 | 0.141 | 0.242 | 0.207 | 0.172 | 0.131 | 0.517 | 0.528 | 0.547 | 0.610 |
| 0.916 | 0.008 | 0.023 | 0.000 | 0.099 | 0.005 | 0.036 | 0.175 | 0.136 | 0.241 | 0.211 | 0.180 | 0.128 | 0.517 | 0.529 | 0.548 | 0.612 |
| 0.925 | 0.006 | 0.000 | 0.066 | 0.000 | 0.002 | 0.070 | 0.114 | 0.170 | 0.241 | 0.211 | 0.180 | 0.149 | 0.517 | 0.530 | 0.548 | 0.613 |
| 0.933 | 0.002 | 0.000 | 0.078 | 0.028 | -0.003 | 0.066 | 0.067 | 0.171 | 0.240 | 0.208 | 0.177 | 0.153 | 0.518 | 0.531 | 0.549 | 0.615 |
| 0.942 | -0.003 | 0.000 | 0.074 | 0.079 | -0.009 | 0.086 | 0.062 | 0.143 | 0.240 | 0.204 | 0.173 | 0.139 | 0.518 | 0.532 | 0.550 | 0.616 |
| 0.951 | -0.009 | 0.000 | 0.069 | 0.084 | -0.013 | 0.104 | 0.067 | 0.135 | 0.240 | 0.201 | 0.170 | 0.130 | 0.519 | 0.533 | 0.551 | 0.618 |
| 0.959 | -0.013 | 0.021 | 0.073 | 0.006 | -0.009 | 0.085 | 0.077 | 0.154 | 0.239 | 0.199 | 0.169 | 0.138 | 0.519 | 0.534 | 0.552 | 0.619 |
| 0.968 | 0.000 | 0.000 | 0.054 | 0.000 | 0.002 | 0.121 | 0.102 | 0.170 | 0.240 | 0.201 | 0.172 | 0.150 | 0.520 | 0.535 | 0.552 | 0.621 |
| 0.977 | -0.007 | -0.001 | 0.000 | 0.067 | 0.013 | 0.058 | 0.206 | 0.151 | 0.240 | 0.206 | 0.179 | 0.144 | 0.520 | 0.536 | 0.553 | 0.622 |
| 0.985 | 0.000 | 0.008 | 0.018 | 0.088 | 0.022 | 0.027 | 0.134 | 0.133 | 0.240 | 0.213 | 0.186 | 0.133 | 0.520 | 0.537 | 0.554 | 0.624 |
| 0.994 | 0.000 | 0.008 | 0.000 | 0.063 | 0.040 | 0.017 | 0.144 | 0.138 | 0.240 | 0.217 | 0.190 | 0.132 | 0.521 | 0.538 | 0.555 | 0.625 |
| 1.000 | 0.005 | 0.010 | 0.054 | 0.000 | 0.030 | 0.012 | 0.088 | 0.168 | 0.242 | 0.216 | 0.190 | 0.144 | 0.521 | 0.539 | 0.555 | 0.627 |
| 1.010 | 0.000 | 0.012 | 0.062 | 0.048 | 0.051 | 0.010 | 0.059 | 0.158 | 0.243 | 0.211 | 0.186 | 0.139 | 0.522 | 0.540 | 0.556 | 0.628 |
| 1.020 | 0.018 | 0.014 | 0.060 | 0.077 | 0.027 | 0.007 | 0.055 | 0.135 | 0.244 | 0.205 | 0.178 | 0.130 | 0.522 | 0.540 | 0.557 | 0.629 |
| 1.030 | 0.000 | 0.014 | 0.062 | 0.078 | 0.058 | 0.003 | 0.058 | 0.127 | 0.244 | 0.201 | 0.173 | 0.125 | 0.522 | 0.541 | 0.558 | 0.631 |
| 1.040 | 0.006 | 0.014 | 0.056 | 0.000 | 0.033 | 0.000 | 0.062 | 0.153 | 0.245 | 0.200 | 0.170 | 0.139 | 0.523 | 0.542 | 0.558 | 0.632 |
| 1.050 | 0.000 | 0.018 | 0.000 | 0.034 | 0.049 | -0.003 | 0.112 | 0.156 | 0.248 | 0.204 | 0.174 | 0.139 | 0.523 | 0.543 | 0.559 | 0.633 |
| 1.050 | 0.000 | 0.019 | 0.004 | 0.075 | 0.042 | -0.001 | 0.145 | 0.136 | 0.249 | 0.210 | 0.182 | 0.130 | 0.523 | 0.544 | 0.560 | 0.635 |
| 1.060 | 0.002 | 0.021 | 0.000 | 0.087 | 0.044 | 0.011 | 0.141 | 0.124 | 0.249 | 0.213 | 0.185 | 0.123 | 0.524 | 0.545 | 0.560 | 0.636 |
| 1.070 | 0.000 | 0.000 | 0.015 | 0.012 | 0.048 | 0.065 | 0.111 | 0.142 | 0.252 | 0.212 | 0.186 | 0.129 | 0.524 | 0.546 | 0.561 | 0.637 |
| 1.080 | 0.004 | 0.049 | 0.059 | 0.000 | 0.038 | 0.037 | 0.068 | 0.157 | 0.252 | 0.210 | 0.183 | 0.147 | 0.524 | 0.547 | 0.562 | 0.638 |
| 1.090 | 0.016 | 0.000 | 0.068 | 0.062 | 0.018 | 0.103 | 0.064 | 0.140 | 0.254 | 0.209 | 0.179 | 0.138 | 0.525 | 0.548 | 0.562 | 0.639 |
| 1.100 | 0.015 | 0.001 | 0.071 | 0.079 | 0.010 | 0.078 | 0.071 | 0.126 | 0.255 | 0.210 | 0.176 | 0.126 | 0.525 | 0.549 | 0.563 | 0.640 |
| 1.110 | 0.010 | 0.000 | 0.073 | 0.060 | 0.007 | 0.104 | 0.081 | 0.134 | 0.256 | 0.214 | 0.175 | 0.124 | 0.525 | 0.549 | 0.564 | 0.642 |
| 1.110 | 0.005 | 0.000 | 0.059 | 0.000 | 0.003 | 0.101 | 0.078 | 0.159 | 0.257 | 0.218 | 0.177 | 0.139 | 0.525 | 0.550 | 0.564 | 0.643 |
| 1.120 | 0.001 | -0.009 | 0.000 | 0.031 | 0.000 | 0.096 | 0.169 | 0.143 | 0.258 | 0.220 | 0.183 | 0.134 | 0.525 | 0.551 | 0.565 | 0.644 |
| 1.130 | -0.002 | 0.026 | 0.038 | 0.050 | -0.003 | 0.060 | 0.143 | 0.123 | 0.259 | 0.219 | 0.189 | 0.125 | 0.526 | 0.552 | 0.566 | 0.645 |
| 1.140 | -0.004 | 0.047 | 0.000 | 0.058 | -0.006 | 0.038 | 0.163 | 0.123 | 0.260 | 0.217 | 0.193 | 0.122 | 0.526 | 0.553 | 0.566 | 0.646 |
| 1.150 | -0.006 | 0.044 | 0.051 | 0.000 | -0.008 | 0.027 | 0.106 | 0.151 | 0.261 | 0.215 | 0.192 | 0.131 | 0.526 | 0.554 | 0.567 | 0.647 |
| 1.160 | -0.008 | 0.039 | 0.078 | 0.006 | -0.010 | 0.016 | 0.074 | 0.145 | 0.262 | 0.215 | 0.190 | 0.132 | 0.526 | 0.555 | 0.567 | 0.648 |
| 1.170 | -0.010 | 0.032 | 0.073 | 0.065 | -0.012 | 0.008 | 0.071 | 0.127 | 0.262 | 0.217 | 0.187 | 0.124 | 0.526 | 0.555 | 0.568 | 0.649 |
| 1.180 | -0.012 | 0.028 | 0.076 | 0.071 | -0.014 | 0.003 | 0.077 | 0.118 | 0.263 | 0.218 | 0.186 | 0.119 | 0.526 | 0.556 | 0.569 | 0.650 |
| 1.180 | -0.014 | 0.028 | 0.077 | 0.014 | -0.016 | 0.000 | 0.078 | 0.134 | 0.264 | 0.217 | 0.187 | 0.126 | 0.527 | 0.557 | 0.569 | 0.651 |
| 1.190 | -0.015 | 0.031 | 0.057 | 0.000 | -0.018 | -0.002 | 0.094 | 0.146 | 0.264 | 0.215 | 0.191 | 0.137 | 0.527 | 0.558 | 0.570 | 0.652 |
| 1.200 | -0.017 | 0.034 | 0.000 | 0.054 | -0.020 | 0.001 | 0.183 | 0.127 | 0.264 | 0.213 | 0.197 | 0.128 | 0.527 | 0.559 | 0.571 | 0.652 |
| 1.210 | -0.019 | 0.041 | 0.027 | 0.075 | -0.023 | 0.017 | 0.127 | 0.114 | 0.265 | 0.215 | 0.201 | 0.121 | 0.527 | 0.560 | 0.571 | 0.653 |
| 1.220 | -0.021 | 0.000 | 0.000 | 0.068 | -0.027 | 0.077 | 0.143 | 0.115 | 0.265 | 0.218 | 0.204 | 0.121 | 0.527 | 0.560 | 0.572 | 0.654 |
| 1.230 | -0.022 | 0.002 | 0.065 | 0.000 | -0.030 | 0.070 | 0.102 | 0.138 | 0.265 | 0.220 | 0.201 | 0.134 | 0.528 | 0.561 | 0.572 | 0.655 |
| 1.240 | -0.021 | 0.000 | 0.075 | 0.059 | -0.031 | 0.116 | 0.077 | 0.133 | 0.266 | 0.221 | 0.198 | 0.131 | 0.528 | 0.562 | 0.573 | 0.656 |
| 1.240 | -0.016 | 0.020 | 0.076 | 0.083 | -0.028 | 0.089 | 0.067 | 0.114 | 0.267 | 0.222 | 0.196 | 0.122 | 0.528 | 0.563 | 0.574 | 0.657 |
| 1.250 | -0.009 | 0.000 | 0.075 | 0.080 | -0.018 | 0.120 | 0.064 | 0.110 | 0.268 | 0.224 | 0.194 | 0.118 | 0.528 | 0.564 | 0.574 | 0.657 |
| 1.260 | -0.011 | 0.022 | 0.067 | 0.000 | 0.000 | 0.087 | 0.063 | 0.133 | 0.270 | 0.227 | 0.195 | 0.132 | 0.529 | 0.564 | 0.575 | 0.658 |
| 1.270 | 0.000 | 0.000 | 0.003 | 0.007 | 0.004 | 0.114 | 0.109 | 0.138 | 0.273 | 0.230 | 0.201 | 0.138 | 0.529 | 0.565 | 0.575 | 0.659 |
| 1.280 | 0.000 | 0.004 | 0.000 | 0.060 | 0.038 | 0.061 | 0.141 | 0.118 | 0.273 | 0.232 | 0.206 | 0.128 | 0.529 | 0.566 | 0.576 | 0.660 |
| 1.290 | 0.011 | 0.011 | 0.000 | 0.069 | 0.033 | 0.041 | 0.141 | 0.108 | 0.275 | 0.232 | 0.209 | 0.118 | 0.530 | 0.566 | 0.576 | 0.660 |
| 1.300 | 0.000 | 0.011 | 0.012 | 0.018 | 0.070 | 0.028 | 0.111 | 0.121 | 0.276 | 0.232 | 0.210 | 0.123 | 0.530 | 0.567 | 0.577 | 0.661 |
| 1.310 | 0.013 | 0.015 | 0.050 | 0.000 | 0.048 | 0.021 | 0.070 | 0.132 | 0.278 | 0.232 | 0.209 | 0.134 | 0.530 | 0.568 | 0.577 | 0.662 |
| 1.310 | 0.000 | 0.018 | 0.064 | 0.059 | 0.073 | 0.017 | 0.059 | 0.117 | 0.281 | 0.232 | 0.206 | 0.120 | 0.530 | 0.568 | 0.578 | 0.662 |
| 1.320 | 0.003 | 0.021 | 0.068 | 0.068 | 0.049 | 0.014 | 0.057 | 0.105 | 0.284 | 0.233 | 0.204 | 0.114 | 0.531 | 0.569 | 0.578 | 0.663 |
| 1.330 | 0.000 | 0.023 | 0.065 | 0.063 | 0.067 | 0.010 | 0.055 | 0.111 | 0.286 | 0.233 | 0.204 | 0.117 | 0.531 | 0.570 | 0.579 | 0.663 |
| 1.340 | 0.000 | 0.023 | 0.061 | 0.000 | 0.065 | 0.006 | 0.059 | 0.134 | 0.287 | 0.233 | 0.207 | 0.136 | 0.531 | 0.570 | 0.579 | 0.664 |
| 1.350 | 0.004 | 0.022 | 0.000 | 0.037 | 0.059 | 0.003 | 0.133 | 0.130 | 0.289 | 0.234 | 0.213 | 0.131 | 0.531 | 0.571 | 0.579 | 0.664 |
| 1.360 | 0.000 | 0.026 | 0.019 | 0.059 | 0.073 | 0.005 | 0.118 | 0.113 | 0.291 | 0.236 | 0.216 | 0.118 | 0.531 | 0.571 | 0.580 | 0.665 |
| 1.370 | -0.003 | 0.022 | 0.000 | 0.060 | 0.061 | 0.028 | 0.146 | 0.107 | 0.294 | 0.239 | 0.221 | 0.114 | 0.531 | 0.572 | 0.580 | 0.665 |
| 1.370 | 0.000 | 0.000 | 0.042 | 0.000 | 0.068 | 0.069 | 0.096 | 0.128 | 0.295 | 0.242 | 0.219 | 0.125 | 0.531 | 0.572 | 0.580 | 0.666 |
| 1.380 | 0.015 | 0.000 | 0.067 | 0.008 | 0.039 | 0.060 | 0.055 | 0.128 | 0.296 | 0.244 | 0.217 | 0.128 | 0.531 | 0.573 | 0.581 | 0.666 |
| 1.390 | 0.021 | 0.000 | 0.073 | 0.043 | 0.026 | 0.088 | 0.055 | 0.112 | 0.298 | 0.245 | 0.216 | 0.118 | 0.531 | 0.573 | 0.581 | 0.666 |
| 1.400 | 0.018 | 0.000 | 0.071 | 0.056 | 0.019 | 0.102 | 0.056 | 0.102 | 0.299 | 0.246 | 0.216 | 0.110 | 0.531 | 0.574 | 0.581 | 0.667 |
| 1.410 | 0.013 | 0.002 | 0.067 | 0.025 | 0.014 | 0.095 | 0.059 | 0.119 | 0.302 | 0.248 | 0.219 | 0.113 | 0.531 | 0.574 | 0.581 | 0.667 |
| 1.420 | 0.008 | 0.000 | 0.058 | 0.000 | 0.008 | 0.106 | 0.069 | 0.128 | 0.304 | 0.250 | 0.226 | 0.123 | 0.531 | 0.574 | 0.581 | 0.667 |
| 1.430 | 0.003 | 0.004 | 0.000 | 0.047 | 0.003 | 0.075 | 0.159 | 0.115 | 0.305 | 0.252 | 0.233 | 0.116 | 0.531 | 0.574 | 0.581 | 0.667 |
| 1.430 | 0.000 | 0.043 | 0.019 | 0.062 | -0.001 | 0.042 | 0.112 | 0.104 | 0.306 | 0.254 | 0.236 | 0.102 | 0.531 | 0.575 | 0.581 | 0.667 |
| 1.440 | -0.003 | 0.042 | 0.000 | 0.052 | -0.004 | 0.032 | 0.124 | 0.110 | 0.307 | 0.256 | 0.237 | 0.102 | 0.531 | 0.575 | 0.581 | 0.668 |
| 1.450 | -0.005 | 0.039 | 0.038 | 0.000 | -0.006 | 0.024 | 0.083 | 0.131 | 0.308 | 0.257 | 0.233 | 0.118 | 0.530 | 0.575 | 0.581 | 0.668 |
| 1.460 | -0.007 | 0.035 | 0.060 | 0.032 | -0.008 | 0.018 | 0.062 | 0.119 | 0.310 | 0.258 | 0.231 | 0.113 | 0.530 | 0.575 | 0.581 | 0.668 |
| 1.470 | -0.008 | 0.029 | 0.070 | 0.054 | -0.010 | 0.009 | 0.061 | 0.103 | 0.311 | 0.259 | 0.231 | 0.102 | 0.530 | 0.575 | 0.580 | 0.668 |
| 1.480 | -0.010 | 0.021 | 0.070 | 0.066 | -0.012 | -0.001 | 0.064 | 0.093 | 0.312 | 0.260 | 0.231 | 0.097 | 0.530 | 0.575 | 0.580 | 0.667 |
| 1.490 | -0.012 | 0.015 | 0.074 | 0.071 | -0.014 | -0.005 | 0.070 | 0.086 | 0.311 | 0.262 | 0.232 | 0.098 | 0.529 | 0.575 | 0.579 | 0.667 |
| 1.500 | -0.013 | 0.013 | 0.084 | 0.075 | -0.015 | -0.007 | 0.076 | 0.082 | 0.313 | 0.263 | 0.233 | 0.103 | 0.529 | 0.575 | 0.579 | 0.667 |
| 1.500 | -0.014 | 0.014 | 0.086 | 0.077 | -0.016 | -0.007 | 0.080 | 0.079 | 0.313 | 0.264 | 0.234 | 0.107 | 0.528 | 0.575 | 0.579 | 0.667 |
| 1.510 | -0.014 | 0.017 | 0.088 | 0.078 | -0.017 | -0.006 | 0.083 | 0.077 | 0.314 | 0.264 | 0.235 | 0.111 | 0.528 | 0.575 | 0.578 | 0.667 |
| 1.520 | -0.014 | 0.021 | 0.090 | 0.078 | -0.017 | -0.004 | 0.085 | 0.075 | 0.314 | 0.264 | 0.235 | 0.113 | 0.528 | 0.575 | 0.578 | 0.667 |
| 1.530 | -0.014 | 0.025 | 0.092 | 0.078 | -0.016 | -0.001 | 0.085 | 0.074 | 0.314 | 0.264 | 0.236 | 0.116 | 0.527 | 0.575 | 0.577 | 0.666 |
| 1.540 | -0.013 | 0.030 | 0.093 | 0.078 | -0.015 | 0.003 | 0.085 | 0.074 | 0.315 | 0.264 | 0.236 | 0.118 | 0.527 | 0.575 | 0.577 | 0.666 |
| 1.550 | -0.011 | 0.035 | 0.094 | 0.078 | -0.014 | 0.008 | 0.085 | 0.073 | 0.316 | 0.265 | 0.237 | 0.120 | 0.526 | 0.575 | 0.576 | 0.666 |
| 1.560 | -0.009 | 0.041 | 0.094 | 0.077 | -0.012 | 0.013 | 0.085 | 0.073 | 0.317 | 0.266 | 0.237 | 0.122 | 0.526 | 0.574 | 0.575 | 0.666 |
| 1.560 | -0.006 | 0.046 | 0.094 | 0.077 | -0.009 | 0.019 | 0.084 | 0.073 | 0.318 | 0.268 | 0.237 | 0.125 | 0.525 | 0.574 | 0.575 | 0.665 |
| 1.570 | -0.003 | 0.051 | 0.093 | 0.077 | -0.006 | 0.025 | 0.084 | 0.073 | 0.320 | 0.269 | 0.237 | 0.128 | 0.525 | 0.574 | 0.574 | 0.665 |
| 1.580 | 0.001 | 0.056 | 0.093 | 0.076 | -0.002 | 0.031 | 0.083 | 0.073 | 0.318 | 0.270 | 0.238 | 0.131 | 0.524 | 0.574 | 0.574 | 0.665 |
| 1.590 | 0.004 | 0.061 | 0.092 | 0.076 | 0.002 | 0.036 | 0.083 | 0.072 | 0.316 | 0.272 | 0.238 | 0.135 | 0.524 | 0.574 | 0.573 | 0.664 |
| 1.600 | 0.009 | 0.065 | 0.092 | 0.075 | 0.006 | 0.042 | 0.083 | 0.072 | 0.316 | 0.273 | 0.238 | 0.138 | 0.523 | 0.573 | 0.573 | 0.664 |
| 1.610 | 0.013 | 0.069 | 0.091 | 0.075 | 0.011 | 0.047 | 0.082 | 0.072 | 0.316 | 0.274 | 0.237 | 0.141 | 0.522 | 0.573 | 0.572 | 0.664 |
| 1.620 | 0.017 | 0.072 | 0.091 | 0.075 | 0.015 | 0.052 | 0.082 | 0.072 | 0.315 | 0.275 | 0.237 | 0.144 | 0.522 | 0.573 | 0.571 | 0.663 |
| 1.620 | 0.022 | 0.076 | 0.090 | 0.075 | 0.020 | 0.057 | 0.082 | 0.072 | 0.315 | 0.277 | 0.237 | 0.147 | 0.521 | 0.573 | 0.571 | 0.663 |
| 1.630 | 0.026 | 0.080 | 0.089 | 0.074 | 0.025 | 0.061 | 0.081 | 0.072 | 0.314 | 0.278 | 0.237 | 0.150 | 0.521 | 0.572 | 0.570 | 0.663 |
| 1.640 | 0.030 | 0.083 | 0.089 | 0.074 | 0.029 | 0.065 | 0.081 | 0.072 | 0.314 | 0.279 | 0.237 | 0.154 | 0.520 | 0.572 | 0.570 | 0.662 |
| 1.650 | 0.035 | 0.085 | 0.088 | 0.074 | 0.034 | 0.069 | 0.081 | 0.072 | 0.313 | 0.279 | 0.236 | 0.157 | 0.520 | 0.572 | 0.569 | 0.662 |
| 1.660 | 0.039 | 0.088 | 0.088 | 0.074 | 0.038 | 0.073 | 0.081 | 0.072 | 0.313 | 0.280 | 0.236 | 0.159 | 0.519 | 0.572 | 0.569 | 0.661 |
| 1.670 | 0.043 | 0.090 | 0.088 | 0.074 | 0.042 | 0.076 | 0.080 | 0.071 | 0.312 | 0.281 | 0.236 | 0.162 | 0.519 | 0.572 | 0.568 | 0.661 |
| 1.680 | 0.047 | 0.092 | 0.087 | 0.074 | 0.047 | 0.080 | 0.080 | 0.071 | 0.311 | 0.281 | 0.235 | 0.164 | 0.518 | 0.571 | 0.568 | 0.661 |
| 1.690 | 0.052 | 0.094 | 0.087 | 0.074 | 0.051 | 0.083 | 0.080 | 0.071 | 0.310 | 0.282 | 0.235 | 0.166 | 0.518 | 0.571 | 0.567 | 0.660 |
| 1.690 | 0.056 | 0.095 | 0.086 | 0.074 | 0.055 | 0.085 | 0.080 | 0.071 | 0.309 | 0.282 | 0.234 | 0.169 | 0.518 | 0.571 | 0.567 | 0.660 |
| 1.700 | 0.060 | 0.097 | 0.086 | 0.074 | 0.059 | 0.088 | 0.080 | 0.071 | 0.308 | 0.282 | 0.234 | 0.171 | 0.517 | 0.571 | 0.566 | 0.660 |
| 1.710 | 0.065 | 0.099 | 0.086 | 0.074 | 0.064 | 0.090 | 0.080 | 0.072 | 0.307 | 0.281 | 0.233 | 0.172 | 0.517 | 0.571 | 0.566 | 0.659 |
| 1.720 | 0.069 | 0.101 | 0.086 | 0.074 | 0.068 | 0.092 | 0.080 | 0.072 | 0.305 | 0.280 | 0.231 | 0.172 | 0.517 | 0.571 | 0.566 | 0.660 |

**Table. 6** In the submerged state (*h*=0.09 m), the streamwise velocity *u* of four different fragmentation conditions changes along the *x* direction.

| **Hydraulic parameters** | **Streamwise velocity u (m/s)** | | | | | | | | | | | | | | | |
| --- | --- | --- | --- | --- | --- | --- | --- | --- | --- | --- | --- | --- | --- | --- | --- | --- |
| ***x* (m)** | **L_1-1_** | | | | **L_2-2_** | | | | **L_3-3_** | | | | **L_4-4_** | | | |
|  | **Fragmentation I** | **Fragmentation II** | **Fragmentation III** | **Fragmentation IV** | **Fragmentation I** | **Fragmentation II** | **Fragmentation III** | **Fragmentation IV** | **Fragmentation I** | **Fragmentation II** | **Fragmentation III** | **Fragmentation IV** | **Fragmentation I** | **Fragmentation II** | **Fragmentation III** | **Fragmentation IV** |
| 0.000 | 0.300 | 0.300 | 0.300 | 0.300 | 0.300 | 0.300 | 0.300 | 0.300 | 0.300 | 0.300 | 0.300 | 0.300 | 0.300 | 0.300 | 0.300 | 0.300 |
| 0.009 | 0.300 | 0.300 | 0.300 | 0.300 | 0.300 | 0.300 | 0.300 | 0.300 | 0.300 | 0.300 | 0.300 | 0.300 | 0.300 | 0.300 | 0.300 | 0.300 |
| 0.017 | 0.300 | 0.300 | 0.300 | 0.300 | 0.300 | 0.300 | 0.300 | 0.300 | 0.300 | 0.300 | 0.300 | 0.300 | 0.300 | 0.300 | 0.300 | 0.300 |
| 0.026 | 0.300 | 0.300 | 0.300 | 0.300 | 0.300 | 0.300 | 0.300 | 0.300 | 0.300 | 0.300 | 0.300 | 0.300 | 0.300 | 0.300 | 0.300 | 0.300 |
| 0.035 | 0.300 | 0.300 | 0.300 | 0.300 | 0.300 | 0.300 | 0.300 | 0.300 | 0.300 | 0.300 | 0.300 | 0.300 | 0.300 | 0.300 | 0.300 | 0.300 |
| 0.043 | 0.300 | 0.300 | 0.300 | 0.300 | 0.300 | 0.300 | 0.300 | 0.300 | 0.300 | 0.300 | 0.300 | 0.300 | 0.301 | 0.301 | 0.301 | 0.301 |
| 0.052 | 0.300 | 0.300 | 0.300 | 0.300 | 0.300 | 0.300 | 0.300 | 0.300 | 0.300 | 0.300 | 0.300 | 0.300 | 0.301 | 0.301 | 0.301 | 0.301 |
| 0.061 | 0.300 | 0.300 | 0.299 | 0.299 | 0.300 | 0.300 | 0.299 | 0.299 | 0.300 | 0.300 | 0.300 | 0.300 | 0.301 | 0.301 | 0.301 | 0.301 |
| 0.069 | 0.300 | 0.299 | 0.299 | 0.299 | 0.300 | 0.299 | 0.299 | 0.299 | 0.300 | 0.300 | 0.300 | 0.301 | 0.302 | 0.302 | 0.302 | 0.302 |
| 0.078 | 0.299 | 0.299 | 0.299 | 0.299 | 0.299 | 0.299 | 0.299 | 0.299 | 0.301 | 0.301 | 0.300 | 0.301 | 0.302 | 0.302 | 0.302 | 0.302 |
| 0.086 | 0.299 | 0.299 | 0.299 | 0.299 | 0.299 | 0.299 | 0.299 | 0.299 | 0.301 | 0.301 | 0.301 | 0.301 | 0.302 | 0.303 | 0.303 | 0.303 |
| 0.095 | 0.299 | 0.299 | 0.298 | 0.298 | 0.299 | 0.299 | 0.298 | 0.298 | 0.301 | 0.301 | 0.301 | 0.301 | 0.303 | 0.303 | 0.303 | 0.304 |
| 0.104 | 0.299 | 0.298 | 0.298 | 0.298 | 0.299 | 0.298 | 0.298 | 0.298 | 0.301 | 0.301 | 0.301 | 0.301 | 0.303 | 0.304 | 0.304 | 0.304 |
| 0.112 | 0.299 | 0.298 | 0.297 | 0.297 | 0.299 | 0.298 | 0.297 | 0.298 | 0.301 | 0.301 | 0.301 | 0.301 | 0.304 | 0.304 | 0.304 | 0.305 |
| 0.121 | 0.298 | 0.297 | 0.297 | 0.297 | 0.298 | 0.297 | 0.297 | 0.297 | 0.301 | 0.301 | 0.301 | 0.301 | 0.304 | 0.305 | 0.305 | 0.306 |
| 0.130 | 0.298 | 0.297 | 0.297 | 0.296 | 0.298 | 0.297 | 0.296 | 0.297 | 0.301 | 0.301 | 0.301 | 0.302 | 0.305 | 0.306 | 0.306 | 0.307 |
| 0.138 | 0.297 | 0.296 | 0.296 | 0.296 | 0.298 | 0.296 | 0.296 | 0.296 | 0.302 | 0.302 | 0.301 | 0.302 | 0.306 | 0.306 | 0.306 | 0.307 |
| 0.147 | 0.297 | 0.295 | 0.295 | 0.295 | 0.297 | 0.295 | 0.295 | 0.296 | 0.302 | 0.302 | 0.301 | 0.302 | 0.306 | 0.307 | 0.307 | 0.308 |
| 0.156 | 0.296 | 0.295 | 0.295 | 0.294 | 0.297 | 0.295 | 0.295 | 0.295 | 0.302 | 0.302 | 0.301 | 0.302 | 0.307 | 0.308 | 0.308 | 0.309 |
| 0.164 | 0.296 | 0.294 | 0.294 | 0.294 | 0.296 | 0.294 | 0.294 | 0.294 | 0.302 | 0.302 | 0.302 | 0.303 | 0.308 | 0.309 | 0.309 | 0.311 |
| 0.173 | 0.295 | 0.293 | 0.293 | 0.293 | 0.295 | 0.293 | 0.293 | 0.294 | 0.302 | 0.302 | 0.302 | 0.303 | 0.309 | 0.310 | 0.310 | 0.312 |
| 0.182 | 0.294 | 0.292 | 0.292 | 0.292 | 0.295 | 0.292 | 0.292 | 0.293 | 0.302 | 0.302 | 0.302 | 0.303 | 0.310 | 0.312 | 0.312 | 0.313 |
| 0.190 | 0.293 | 0.291 | 0.292 | 0.291 | 0.294 | 0.291 | 0.292 | 0.292 | 0.303 | 0.303 | 0.302 | 0.304 | 0.311 | 0.313 | 0.313 | 0.315 |
| 0.199 | 0.293 | 0.290 | 0.291 | 0.290 | 0.293 | 0.290 | 0.291 | 0.291 | 0.303 | 0.303 | 0.303 | 0.304 | 0.312 | 0.314 | 0.314 | 0.316 |
| 0.207 | 0.292 | 0.289 | 0.290 | 0.290 | 0.292 | 0.289 | 0.290 | 0.290 | 0.303 | 0.303 | 0.303 | 0.305 | 0.313 | 0.316 | 0.315 | 0.318 |
| 0.216 | 0.290 | 0.288 | 0.289 | 0.289 | 0.291 | 0.288 | 0.289 | 0.290 | 0.304 | 0.304 | 0.304 | 0.305 | 0.314 | 0.317 | 0.317 | 0.319 |
| 0.225 | 0.289 | 0.287 | 0.289 | 0.288 | 0.290 | 0.287 | 0.288 | 0.289 | 0.304 | 0.304 | 0.304 | 0.306 | 0.316 | 0.319 | 0.319 | 0.321 |
| 0.233 | 0.288 | 0.286 | 0.288 | 0.288 | 0.288 | 0.286 | 0.288 | 0.289 | 0.304 | 0.304 | 0.305 | 0.307 | 0.317 | 0.321 | 0.321 | 0.324 |
| 0.242 | 0.286 | 0.285 | 0.288 | 0.289 | 0.287 | 0.285 | 0.288 | 0.289 | 0.305 | 0.305 | 0.306 | 0.309 | 0.319 | 0.323 | 0.323 | 0.326 |
| 0.251 | 0.285 | 0.284 | 0.289 | 0.290 | 0.285 | 0.284 | 0.289 | 0.291 | 0.305 | 0.306 | 0.308 | 0.311 | 0.320 | 0.325 | 0.325 | 0.328 |
| 0.259 | 0.283 | 0.283 | 0.291 | 0.293 | 0.283 | 0.283 | 0.291 | 0.294 | 0.306 | 0.307 | 0.309 | 0.315 | 0.322 | 0.327 | 0.327 | 0.330 |
| 0.268 | 0.281 | 0.283 | 0.294 | 0.299 | 0.281 | 0.283 | 0.293 | 0.300 | 0.307 | 0.309 | 0.312 | 0.320 | 0.324 | 0.330 | 0.330 | 0.333 |
| 0.277 | 0.278 | 0.283 | 0.299 | 0.311 | 0.279 | 0.282 | 0.299 | 0.312 | 0.308 | 0.312 | 0.317 | 0.328 | 0.326 | 0.332 | 0.332 | 0.335 |
| 0.285 | 0.276 | 0.284 | 0.310 | 0.334 | 0.276 | 0.283 | 0.309 | 0.333 | 0.309 | 0.315 | 0.325 | 0.340 | 0.328 | 0.335 | 0.335 | 0.338 |
| 0.294 | 0.273 | 0.286 | 0.335 | 0.345 | 0.274 | 0.284 | 0.333 | 0.344 | 0.311 | 0.320 | 0.336 | 0.346 | 0.331 | 0.337 | 0.338 | 0.341 |
| 0.303 | 0.270 | 0.292 | 0.361 | 0.342 | 0.271 | 0.291 | 0.361 | 0.343 | 0.313 | 0.327 | 0.347 | 0.347 | 0.333 | 0.340 | 0.340 | 0.344 |
| 0.311 | 0.267 | 0.309 | 0.374 | 0.344 | 0.268 | 0.309 | 0.374 | 0.345 | 0.315 | 0.335 | 0.355 | 0.349 | 0.336 | 0.343 | 0.343 | 0.346 |
| 0.320 | 0.264 | 0.348 | 0.378 | 0.356 | 0.265 | 0.345 | 0.380 | 0.354 | 0.318 | 0.346 | 0.358 | 0.357 | 0.338 | 0.346 | 0.345 | 0.349 |
| 0.328 | 0.263 | 0.374 | 0.371 | 0.369 | 0.263 | 0.373 | 0.372 | 0.365 | 0.322 | 0.355 | 0.358 | 0.363 | 0.341 | 0.349 | 0.348 | 0.352 |
| 0.337 | 0.263 | 0.392 | 0.364 | 0.364 | 0.263 | 0.390 | 0.361 | 0.363 | 0.328 | 0.364 | 0.358 | 0.363 | 0.344 | 0.352 | 0.351 | 0.355 |
| 0.346 | 0.268 | 0.402 | 0.362 | 0.361 | 0.267 | 0.400 | 0.353 | 0.358 | 0.334 | 0.371 | 0.358 | 0.362 | 0.347 | 0.355 | 0.354 | 0.357 |
| 0.354 | 0.283 | 0.407 | 0.365 | 0.369 | 0.283 | 0.408 | 0.356 | 0.365 | 0.340 | 0.378 | 0.362 | 0.367 | 0.350 | 0.358 | 0.357 | 0.360 |
| 0.363 | 0.317 | 0.411 | 0.372 | 0.382 | 0.320 | 0.406 | 0.359 | 0.372 | 0.347 | 0.383 | 0.368 | 0.373 | 0.353 | 0.361 | 0.360 | 0.363 |
| 0.372 | 0.352 | 0.414 | 0.386 | 0.381 | 0.353 | 0.402 | 0.374 | 0.367 | 0.355 | 0.386 | 0.376 | 0.375 | 0.357 | 0.364 | 0.362 | 0.366 |
| 0.380 | 0.369 | 0.406 | 0.390 | 0.377 | 0.371 | 0.394 | 0.380 | 0.364 | 0.363 | 0.388 | 0.382 | 0.373 | 0.360 | 0.367 | 0.365 | 0.369 |
| 0.389 | 0.372 | 0.395 | 0.394 | 0.380 | 0.377 | 0.370 | 0.378 | 0.357 | 0.370 | 0.389 | 0.386 | 0.377 | 0.363 | 0.370 | 0.368 | 0.371 |
| 0.398 | 0.355 | 0.384 | 0.387 | 0.392 | 0.371 | 0.364 | 0.376 | 0.364 | 0.377 | 0.389 | 0.385 | 0.385 | 0.366 | 0.372 | 0.371 | 0.374 |
| 0.406 | 0.360 | 0.371 | 0.372 | 0.396 | 0.360 | 0.354 | 0.370 | 0.365 | 0.384 | 0.389 | 0.380 | 0.388 | 0.370 | 0.375 | 0.373 | 0.377 |
| 0.415 | 0.370 | 0.358 | 0.374 | 0.392 | 0.356 | 0.349 | 0.367 | 0.361 | 0.389 | 0.389 | 0.378 | 0.386 | 0.373 | 0.378 | 0.376 | 0.379 |
| 0.424 | 0.361 | 0.345 | 0.369 | 0.392 | 0.341 | 0.346 | 0.359 | 0.356 | 0.395 | 0.390 | 0.373 | 0.385 | 0.376 | 0.380 | 0.379 | 0.382 |
| 0.432 | 0.354 | 0.356 | 0.366 | 0.400 | 0.332 | 0.340 | 0.363 | 0.358 | 0.401 | 0.390 | 0.374 | 0.390 | 0.379 | 0.383 | 0.381 | 0.385 |
| 0.441 | 0.346 | 0.344 | 0.371 | 0.408 | 0.335 | 0.331 | 0.371 | 0.363 | 0.405 | 0.390 | 0.380 | 0.394 | 0.382 | 0.385 | 0.384 | 0.387 |
| 0.449 | 0.341 | 0.355 | 0.386 | 0.405 | 0.329 | 0.330 | 0.387 | 0.363 | 0.410 | 0.392 | 0.384 | 0.390 | 0.385 | 0.387 | 0.386 | 0.390 |
| 0.458 | 0.323 | 0.358 | 0.387 | 0.404 | 0.334 | 0.330 | 0.392 | 0.360 | 0.414 | 0.395 | 0.388 | 0.389 | 0.387 | 0.390 | 0.389 | 0.392 |
| 0.467 | 0.305 | 0.365 | 0.384 | 0.409 | 0.331 | 0.335 | 0.398 | 0.361 | 0.417 | 0.394 | 0.386 | 0.389 | 0.390 | 0.392 | 0.392 | 0.395 |
| 0.475 | 0.299 | 0.371 | 0.381 | 0.418 | 0.324 | 0.340 | 0.395 | 0.372 | 0.419 | 0.394 | 0.375 | 0.396 | 0.393 | 0.394 | 0.394 | 0.397 |
| 0.484 | 0.289 | 0.374 | 0.374 | 0.417 | 0.313 | 0.338 | 0.386 | 0.378 | 0.421 | 0.398 | 0.377 | 0.393 | 0.395 | 0.397 | 0.396 | 0.399 |
| 0.493 | 0.286 | 0.374 | 0.373 | 0.412 | 0.296 | 0.336 | 0.383 | 0.376 | 0.423 | 0.397 | 0.372 | 0.391 | 0.397 | 0.399 | 0.399 | 0.402 |
| 0.501 | 0.271 | 0.372 | 0.369 | 0.413 | 0.285 | 0.334 | 0.379 | 0.377 | 0.423 | 0.393 | 0.371 | 0.396 | 0.400 | 0.401 | 0.401 | 0.404 |
| 0.510 | 0.271 | 0.373 | 0.366 | 0.422 | 0.274 | 0.335 | 0.392 | 0.392 | 0.425 | 0.382 | 0.373 | 0.401 | 0.402 | 0.403 | 0.404 | 0.407 |
| 0.519 | 0.260 | 0.375 | 0.376 | 0.422 | 0.267 | 0.333 | 0.403 | 0.399 | 0.425 | 0.382 | 0.381 | 0.398 | 0.404 | 0.405 | 0.406 | 0.409 |
| 0.527 | 0.255 | 0.366 | 0.386 | 0.418 | 0.257 | 0.325 | 0.414 | 0.396 | 0.425 | 0.376 | 0.384 | 0.395 | 0.406 | 0.407 | 0.408 | 0.411 |
| 0.536 | 0.278 | 0.362 | 0.395 | 0.413 | 0.264 | 0.325 | 0.419 | 0.399 | 0.426 | 0.368 | 0.385 | 0.394 | 0.407 | 0.409 | 0.411 | 0.414 |
| 0.545 | 0.278 | 0.358 | 0.397 | 0.415 | 0.253 | 0.315 | 0.420 | 0.410 | 0.427 | 0.359 | 0.385 | 0.402 | 0.409 | 0.410 | 0.413 | 0.416 |
| 0.553 | 0.261 | 0.351 | 0.388 | 0.422 | 0.245 | 0.316 | 0.410 | 0.416 | 0.426 | 0.360 | 0.387 | 0.400 | 0.411 | 0.412 | 0.415 | 0.418 |
| 0.562 | 0.257 | 0.341 | 0.382 | 0.419 | 0.235 | 0.300 | 0.400 | 0.414 | 0.424 | 0.360 | 0.383 | 0.397 | 0.412 | 0.414 | 0.417 | 0.420 |
| 0.570 | 0.271 | 0.345 | 0.383 | 0.411 | 0.244 | 0.302 | 0.400 | 0.408 | 0.425 | 0.356 | 0.385 | 0.397 | 0.414 | 0.415 | 0.420 | 0.422 |
| 0.579 | 0.267 | 0.344 | 0.380 | 0.411 | 0.241 | 0.305 | 0.398 | 0.416 | 0.426 | 0.358 | 0.383 | 0.400 | 0.415 | 0.417 | 0.422 | 0.425 |
| 0.588 | 0.263 | 0.344 | 0.385 | 0.415 | 0.238 | 0.290 | 0.404 | 0.426 | 0.425 | 0.343 | 0.391 | 0.403 | 0.417 | 0.418 | 0.424 | 0.427 |
| 0.596 | 0.259 | 0.338 | 0.397 | 0.412 | 0.235 | 0.291 | 0.412 | 0.427 | 0.424 | 0.345 | 0.396 | 0.406 | 0.418 | 0.420 | 0.426 | 0.429 |
| 0.605 | 0.256 | 0.338 | 0.403 | 0.408 | 0.235 | 0.291 | 0.418 | 0.426 | 0.423 | 0.345 | 0.398 | 0.402 | 0.419 | 0.422 | 0.428 | 0.431 |
| 0.614 | 0.254 | 0.340 | 0.404 | 0.405 | 0.234 | 0.289 | 0.417 | 0.422 | 0.422 | 0.346 | 0.401 | 0.404 | 0.420 | 0.423 | 0.431 | 0.433 |
| 0.622 | 0.244 | 0.345 | 0.399 | 0.409 | 0.224 | 0.292 | 0.405 | 0.433 | 0.419 | 0.346 | 0.404 | 0.406 | 0.422 | 0.424 | 0.433 | 0.435 |
| 0.631 | 0.238 | 0.346 | 0.387 | 0.407 | 0.212 | 0.293 | 0.400 | 0.435 | 0.419 | 0.346 | 0.401 | 0.404 | 0.423 | 0.426 | 0.434 | 0.437 |
| 0.640 | 0.225 | 0.346 | 0.387 | 0.399 | 0.207 | 0.292 | 0.394 | 0.427 | 0.415 | 0.348 | 0.390 | 0.395 | 0.424 | 0.427 | 0.436 | 0.439 |
| 0.648 | 0.219 | 0.342 | 0.388 | 0.396 | 0.201 | 0.294 | 0.381 | 0.423 | 0.410 | 0.345 | 0.393 | 0.396 | 0.425 | 0.429 | 0.438 | 0.441 |
| 0.657 | 0.220 | 0.341 | 0.368 | 0.398 | 0.207 | 0.293 | 0.384 | 0.432 | 0.410 | 0.349 | 0.397 | 0.398 | 0.426 | 0.430 | 0.440 | 0.443 |
| 0.666 | 0.226 | 0.340 | 0.374 | 0.401 | 0.215 | 0.294 | 0.393 | 0.434 | 0.410 | 0.349 | 0.400 | 0.397 | 0.427 | 0.431 | 0.442 | 0.445 |
| 0.674 | 0.227 | 0.330 | 0.390 | 0.395 | 0.215 | 0.284 | 0.404 | 0.429 | 0.405 | 0.348 | 0.403 | 0.391 | 0.428 | 0.433 | 0.444 | 0.446 |
| 0.683 | 0.230 | 0.323 | 0.392 | 0.390 | 0.218 | 0.280 | 0.402 | 0.429 | 0.404 | 0.347 | 0.407 | 0.391 | 0.429 | 0.434 | 0.446 | 0.448 |
| 0.691 | 0.234 | 0.318 | 0.398 | 0.393 | 0.217 | 0.274 | 0.403 | 0.426 | 0.400 | 0.346 | 0.406 | 0.391 | 0.430 | 0.435 | 0.448 | 0.450 |
| 0.700 | 0.239 | 0.309 | 0.394 | 0.393 | 0.221 | 0.264 | 0.393 | 0.427 | 0.397 | 0.345 | 0.403 | 0.394 | 0.431 | 0.436 | 0.449 | 0.451 |
| 0.709 | 0.242 | 0.298 | 0.394 | 0.388 | 0.225 | 0.264 | 0.379 | 0.423 | 0.395 | 0.345 | 0.396 | 0.390 | 0.432 | 0.437 | 0.451 | 0.453 |
| 0.717 | 0.247 | 0.297 | 0.388 | 0.384 | 0.228 | 0.258 | 0.366 | 0.423 | 0.390 | 0.344 | 0.394 | 0.389 | 0.433 | 0.439 | 0.453 | 0.455 |
| 0.726 | 0.247 | 0.292 | 0.380 | 0.381 | 0.230 | 0.254 | 0.373 | 0.421 | 0.387 | 0.347 | 0.400 | 0.391 | 0.434 | 0.440 | 0.455 | 0.457 |
| 0.735 | 0.248 | 0.278 | 0.386 | 0.387 | 0.231 | 0.247 | 0.370 | 0.420 | 0.382 | 0.341 | 0.399 | 0.394 | 0.435 | 0.441 | 0.457 | 0.458 |
| 0.743 | 0.248 | 0.280 | 0.391 | 0.385 | 0.231 | 0.247 | 0.376 | 0.421 | 0.376 | 0.340 | 0.400 | 0.394 | 0.436 | 0.442 | 0.458 | 0.460 |
| 0.752 | 0.249 | 0.275 | 0.394 | 0.382 | 0.233 | 0.239 | 0.381 | 0.414 | 0.370 | 0.337 | 0.405 | 0.392 | 0.436 | 0.443 | 0.455 | 0.461 |
| 0.761 | 0.248 | 0.272 | 0.392 | 0.378 | 0.231 | 0.238 | 0.385 | 0.414 | 0.368 | 0.334 | 0.405 | 0.398 | 0.437 | 0.444 | 0.454 | 0.463 |
| 0.769 | 0.243 | 0.282 | 0.389 | 0.376 | 0.227 | 0.251 | 0.384 | 0.411 | 0.364 | 0.339 | 0.403 | 0.402 | 0.438 | 0.445 | 0.455 | 0.464 |
| 0.778 | 0.240 | 0.289 | 0.379 | 0.377 | 0.219 | 0.255 | 0.373 | 0.412 | 0.358 | 0.339 | 0.405 | 0.409 | 0.439 | 0.446 | 0.454 | 0.466 |
| 0.787 | 0.238 | 0.294 | 0.376 | 0.376 | 0.215 | 0.265 | 0.364 | 0.404 | 0.354 | 0.338 | 0.400 | 0.410 | 0.439 | 0.448 | 0.456 | 0.467 |
| 0.795 | 0.230 | 0.294 | 0.375 | 0.370 | 0.212 | 0.263 | 0.360 | 0.397 | 0.352 | 0.342 | 0.400 | 0.410 | 0.440 | 0.449 | 0.457 | 0.469 |
| 0.804 | 0.224 | 0.298 | 0.368 | 0.369 | 0.202 | 0.266 | 0.346 | 0.396 | 0.348 | 0.337 | 0.403 | 0.408 | 0.440 | 0.450 | 0.459 | 0.470 |
| 0.812 | 0.209 | 0.299 | 0.372 | 0.372 | 0.198 | 0.261 | 0.345 | 0.391 | 0.350 | 0.339 | 0.402 | 0.414 | 0.441 | 0.451 | 0.460 | 0.472 |
| 0.821 | 0.212 | 0.293 | 0.385 | 0.366 | 0.199 | 0.259 | 0.352 | 0.394 | 0.343 | 0.337 | 0.405 | 0.418 | 0.442 | 0.452 | 0.461 | 0.473 |
| 0.830 | 0.206 | 0.286 | 0.388 | 0.362 | 0.200 | 0.252 | 0.348 | 0.381 | 0.339 | 0.331 | 0.408 | 0.417 | 0.442 | 0.453 | 0.463 | 0.474 |
| 0.838 | 0.213 | 0.274 | 0.385 | 0.364 | 0.200 | 0.240 | 0.348 | 0.377 | 0.338 | 0.331 | 0.414 | 0.415 | 0.443 | 0.454 | 0.464 | 0.475 |
| 0.847 | 0.212 | 0.269 | 0.383 | 0.363 | 0.198 | 0.222 | 0.344 | 0.372 | 0.330 | 0.331 | 0.413 | 0.420 | 0.443 | 0.455 | 0.465 | 0.477 |
| 0.856 | 0.204 | 0.259 | 0.376 | 0.364 | 0.199 | 0.219 | 0.340 | 0.372 | 0.330 | 0.331 | 0.410 | 0.422 | 0.444 | 0.456 | 0.467 | 0.478 |
| 0.864 | 0.207 | 0.256 | 0.373 | 0.364 | 0.196 | 0.220 | 0.338 | 0.364 | 0.325 | 0.328 | 0.409 | 0.418 | 0.444 | 0.457 | 0.468 | 0.479 |
| 0.873 | 0.205 | 0.245 | 0.367 | 0.362 | 0.194 | 0.204 | 0.338 | 0.358 | 0.324 | 0.331 | 0.412 | 0.414 | 0.444 | 0.458 | 0.470 | 0.481 |
| 0.882 | 0.200 | 0.234 | 0.362 | 0.369 | 0.189 | 0.200 | 0.339 | 0.354 | 0.319 | 0.327 | 0.413 | 0.418 | 0.445 | 0.459 | 0.471 | 0.482 |
| 0.890 | 0.194 | 0.226 | 0.367 | 0.377 | 0.187 | 0.197 | 0.342 | 0.360 | 0.317 | 0.322 | 0.419 | 0.423 | 0.445 | 0.460 | 0.472 | 0.483 |
| 0.899 | 0.194 | 0.218 | 0.378 | 0.377 | 0.184 | 0.192 | 0.355 | 0.352 | 0.314 | 0.319 | 0.422 | 0.417 | 0.446 | 0.461 | 0.473 | 0.484 |
| 0.908 | 0.190 | 0.218 | 0.380 | 0.380 | 0.179 | 0.192 | 0.355 | 0.354 | 0.312 | 0.318 | 0.425 | 0.416 | 0.446 | 0.462 | 0.475 | 0.486 |
| 0.916 | 0.184 | 0.228 | 0.381 | 0.379 | 0.173 | 0.205 | 0.349 | 0.350 | 0.310 | 0.318 | 0.425 | 0.416 | 0.447 | 0.464 | 0.476 | 0.487 |
| 0.925 | 0.176 | 0.245 | 0.372 | 0.390 | 0.168 | 0.221 | 0.338 | 0.358 | 0.307 | 0.320 | 0.426 | 0.418 | 0.447 | 0.465 | 0.477 | 0.488 |
| 0.933 | 0.166 | 0.252 | 0.368 | 0.389 | 0.157 | 0.231 | 0.334 | 0.359 | 0.305 | 0.321 | 0.423 | 0.415 | 0.448 | 0.466 | 0.478 | 0.489 |
| 0.942 | 0.160 | 0.257 | 0.361 | 0.387 | 0.154 | 0.236 | 0.323 | 0.357 | 0.304 | 0.321 | 0.424 | 0.410 | 0.448 | 0.467 | 0.479 | 0.490 |
| 0.951 | 0.159 | 0.259 | 0.357 | 0.392 | 0.151 | 0.240 | 0.315 | 0.353 | 0.302 | 0.322 | 0.418 | 0.409 | 0.449 | 0.468 | 0.480 | 0.491 |
| 0.959 | 0.162 | 0.262 | 0.353 | 0.397 | 0.154 | 0.238 | 0.306 | 0.353 | 0.300 | 0.320 | 0.418 | 0.412 | 0.449 | 0.469 | 0.481 | 0.492 |
| 0.968 | 0.168 | 0.258 | 0.352 | 0.398 | 0.157 | 0.232 | 0.301 | 0.354 | 0.302 | 0.322 | 0.422 | 0.408 | 0.450 | 0.470 | 0.482 | 0.493 |
| 0.977 | 0.172 | 0.255 | 0.355 | 0.395 | 0.159 | 0.221 | 0.303 | 0.347 | 0.299 | 0.319 | 0.426 | 0.406 | 0.450 | 0.471 | 0.484 | 0.494 |
| 0.985 | 0.175 | 0.241 | 0.351 | 0.397 | 0.163 | 0.206 | 0.300 | 0.344 | 0.299 | 0.318 | 0.429 | 0.400 | 0.451 | 0.472 | 0.485 | 0.495 |
| 0.994 | 0.181 | 0.231 | 0.350 | 0.399 | 0.168 | 0.197 | 0.302 | 0.341 | 0.298 | 0.317 | 0.431 | 0.402 | 0.451 | 0.473 | 0.486 | 0.496 |
| 1.000 | 0.185 | 0.224 | 0.345 | 0.407 | 0.172 | 0.196 | 0.301 | 0.350 | 0.300 | 0.317 | 0.430 | 0.399 | 0.452 | 0.474 | 0.487 | 0.497 |
| 1.010 | 0.189 | 0.222 | 0.340 | 0.405 | 0.176 | 0.194 | 0.301 | 0.348 | 0.301 | 0.312 | 0.428 | 0.396 | 0.453 | 0.474 | 0.488 | 0.498 |
| 1.020 | 0.192 | 0.216 | 0.341 | 0.405 | 0.179 | 0.184 | 0.298 | 0.349 | 0.305 | 0.311 | 0.429 | 0.389 | 0.453 | 0.475 | 0.489 | 0.499 |
| 1.030 | 0.196 | 0.201 | 0.336 | 0.407 | 0.182 | 0.178 | 0.296 | 0.352 | 0.301 | 0.313 | 0.432 | 0.390 | 0.454 | 0.476 | 0.490 | 0.500 |
| 1.040 | 0.198 | 0.197 | 0.338 | 0.415 | 0.184 | 0.172 | 0.298 | 0.360 | 0.302 | 0.310 | 0.437 | 0.387 | 0.454 | 0.477 | 0.491 | 0.501 |
| 1.050 | 0.200 | 0.193 | 0.345 | 0.416 | 0.186 | 0.167 | 0.302 | 0.360 | 0.303 | 0.310 | 0.439 | 0.386 | 0.455 | 0.478 | 0.491 | 0.502 |
| 1.050 | 0.201 | 0.189 | 0.353 | 0.415 | 0.187 | 0.167 | 0.307 | 0.360 | 0.302 | 0.309 | 0.439 | 0.386 | 0.455 | 0.479 | 0.492 | 0.503 |
| 1.060 | 0.200 | 0.198 | 0.353 | 0.416 | 0.188 | 0.175 | 0.311 | 0.362 | 0.302 | 0.311 | 0.440 | 0.378 | 0.456 | 0.480 | 0.493 | 0.504 |
| 1.070 | 0.200 | 0.216 | 0.346 | 0.425 | 0.186 | 0.197 | 0.304 | 0.366 | 0.301 | 0.311 | 0.437 | 0.377 | 0.456 | 0.481 | 0.494 | 0.505 |
| 1.080 | 0.198 | 0.227 | 0.338 | 0.429 | 0.186 | 0.210 | 0.292 | 0.368 | 0.300 | 0.314 | 0.438 | 0.375 | 0.457 | 0.482 | 0.495 | 0.506 |
| 1.090 | 0.193 | 0.234 | 0.331 | 0.424 | 0.182 | 0.217 | 0.284 | 0.365 | 0.299 | 0.317 | 0.432 | 0.369 | 0.457 | 0.483 | 0.496 | 0.507 |
| 1.100 | 0.186 | 0.240 | 0.326 | 0.423 | 0.176 | 0.221 | 0.280 | 0.364 | 0.298 | 0.318 | 0.434 | 0.369 | 0.458 | 0.484 | 0.497 | 0.508 |
| 1.110 | 0.183 | 0.244 | 0.319 | 0.431 | 0.172 | 0.224 | 0.275 | 0.365 | 0.296 | 0.317 | 0.437 | 0.364 | 0.458 | 0.485 | 0.498 | 0.509 |
| 1.110 | 0.180 | 0.242 | 0.321 | 0.430 | 0.170 | 0.225 | 0.277 | 0.367 | 0.296 | 0.316 | 0.438 | 0.365 | 0.459 | 0.486 | 0.499 | 0.510 |
| 1.120 | 0.169 | 0.239 | 0.331 | 0.429 | 0.167 | 0.216 | 0.288 | 0.364 | 0.294 | 0.314 | 0.444 | 0.361 | 0.459 | 0.486 | 0.500 | 0.511 |
| 1.130 | 0.168 | 0.230 | 0.330 | 0.425 | 0.163 | 0.203 | 0.290 | 0.361 | 0.293 | 0.314 | 0.446 | 0.357 | 0.459 | 0.487 | 0.501 | 0.511 |
| 1.140 | 0.166 | 0.219 | 0.329 | 0.426 | 0.161 | 0.201 | 0.292 | 0.365 | 0.292 | 0.314 | 0.443 | 0.359 | 0.460 | 0.488 | 0.501 | 0.512 |
| 1.150 | 0.165 | 0.207 | 0.322 | 0.437 | 0.159 | 0.188 | 0.286 | 0.369 | 0.291 | 0.313 | 0.437 | 0.361 | 0.460 | 0.489 | 0.502 | 0.513 |
| 1.160 | 0.163 | 0.199 | 0.315 | 0.436 | 0.157 | 0.184 | 0.280 | 0.372 | 0.290 | 0.310 | 0.437 | 0.359 | 0.461 | 0.490 | 0.503 | 0.514 |
| 1.170 | 0.160 | 0.198 | 0.312 | 0.431 | 0.154 | 0.179 | 0.279 | 0.369 | 0.289 | 0.312 | 0.434 | 0.359 | 0.461 | 0.490 | 0.504 | 0.515 |
| 1.180 | 0.158 | 0.193 | 0.309 | 0.435 | 0.152 | 0.172 | 0.272 | 0.375 | 0.288 | 0.310 | 0.434 | 0.357 | 0.461 | 0.491 | 0.504 | 0.516 |
| 1.180 | 0.158 | 0.187 | 0.307 | 0.439 | 0.153 | 0.168 | 0.274 | 0.380 | 0.287 | 0.310 | 0.434 | 0.357 | 0.462 | 0.492 | 0.505 | 0.516 |
| 1.190 | 0.154 | 0.178 | 0.313 | 0.441 | 0.148 | 0.156 | 0.278 | 0.381 | 0.284 | 0.311 | 0.440 | 0.359 | 0.462 | 0.493 | 0.506 | 0.517 |
| 1.200 | 0.152 | 0.169 | 0.320 | 0.434 | 0.145 | 0.150 | 0.284 | 0.377 | 0.282 | 0.312 | 0.440 | 0.357 | 0.463 | 0.494 | 0.507 | 0.518 |
| 1.210 | 0.149 | 0.169 | 0.321 | 0.430 | 0.140 | 0.152 | 0.283 | 0.375 | 0.281 | 0.311 | 0.437 | 0.360 | 0.463 | 0.494 | 0.508 | 0.519 |
| 1.220 | 0.142 | 0.183 | 0.315 | 0.433 | 0.137 | 0.166 | 0.279 | 0.376 | 0.278 | 0.312 | 0.438 | 0.360 | 0.464 | 0.495 | 0.509 | 0.519 |
| 1.230 | 0.135 | 0.190 | 0.307 | 0.439 | 0.129 | 0.178 | 0.271 | 0.383 | 0.278 | 0.312 | 0.436 | 0.362 | 0.464 | 0.496 | 0.509 | 0.520 |
| 1.240 | 0.134 | 0.196 | 0.299 | 0.434 | 0.123 | 0.186 | 0.266 | 0.380 | 0.276 | 0.316 | 0.433 | 0.358 | 0.465 | 0.497 | 0.510 | 0.521 |
| 1.240 | 0.132 | 0.202 | 0.299 | 0.429 | 0.121 | 0.190 | 0.263 | 0.378 | 0.277 | 0.315 | 0.431 | 0.356 | 0.465 | 0.497 | 0.511 | 0.522 |
| 1.250 | 0.127 | 0.209 | 0.299 | 0.427 | 0.120 | 0.191 | 0.265 | 0.372 | 0.277 | 0.315 | 0.428 | 0.358 | 0.466 | 0.498 | 0.511 | 0.522 |
| 1.260 | 0.134 | 0.207 | 0.303 | 0.435 | 0.127 | 0.189 | 0.273 | 0.377 | 0.276 | 0.316 | 0.431 | 0.360 | 0.466 | 0.499 | 0.512 | 0.523 |
| 1.270 | 0.139 | 0.205 | 0.312 | 0.432 | 0.130 | 0.185 | 0.283 | 0.377 | 0.277 | 0.315 | 0.433 | 0.361 | 0.467 | 0.499 | 0.512 | 0.524 |
| 1.280 | 0.143 | 0.194 | 0.319 | 0.423 | 0.134 | 0.172 | 0.287 | 0.372 | 0.275 | 0.315 | 0.436 | 0.359 | 0.467 | 0.500 | 0.513 | 0.524 |
| 1.290 | 0.148 | 0.184 | 0.321 | 0.419 | 0.140 | 0.154 | 0.287 | 0.373 | 0.276 | 0.313 | 0.437 | 0.359 | 0.468 | 0.501 | 0.513 | 0.525 |
| 1.300 | 0.155 | 0.177 | 0.318 | 0.428 | 0.146 | 0.146 | 0.284 | 0.377 | 0.276 | 0.309 | 0.435 | 0.362 | 0.468 | 0.501 | 0.514 | 0.525 |
| 1.310 | 0.161 | 0.171 | 0.312 | 0.424 | 0.151 | 0.145 | 0.279 | 0.380 | 0.275 | 0.312 | 0.429 | 0.363 | 0.469 | 0.502 | 0.515 | 0.526 |
| 1.310 | 0.166 | 0.174 | 0.305 | 0.420 | 0.157 | 0.148 | 0.274 | 0.379 | 0.275 | 0.310 | 0.428 | 0.364 | 0.469 | 0.503 | 0.515 | 0.527 |
| 1.320 | 0.169 | 0.169 | 0.300 | 0.412 | 0.161 | 0.147 | 0.264 | 0.375 | 0.273 | 0.312 | 0.426 | 0.361 | 0.470 | 0.503 | 0.514 | 0.527 |
| 1.330 | 0.172 | 0.167 | 0.299 | 0.415 | 0.163 | 0.141 | 0.261 | 0.380 | 0.274 | 0.311 | 0.429 | 0.364 | 0.470 | 0.504 | 0.516 | 0.528 |
| 1.340 | 0.176 | 0.168 | 0.301 | 0.421 | 0.166 | 0.144 | 0.262 | 0.384 | 0.274 | 0.310 | 0.430 | 0.368 | 0.470 | 0.504 | 0.517 | 0.529 |
| 1.350 | 0.178 | 0.170 | 0.315 | 0.414 | 0.167 | 0.143 | 0.274 | 0.382 | 0.274 | 0.311 | 0.433 | 0.366 | 0.471 | 0.505 | 0.518 | 0.529 |
| 1.360 | 0.180 | 0.172 | 0.318 | 0.409 | 0.171 | 0.147 | 0.278 | 0.381 | 0.271 | 0.312 | 0.436 | 0.366 | 0.471 | 0.505 | 0.518 | 0.530 |
| 1.370 | 0.180 | 0.186 | 0.317 | 0.413 | 0.172 | 0.161 | 0.281 | 0.386 | 0.271 | 0.314 | 0.437 | 0.366 | 0.471 | 0.506 | 0.519 | 0.530 |
| 1.370 | 0.180 | 0.204 | 0.316 | 0.416 | 0.170 | 0.175 | 0.279 | 0.390 | 0.271 | 0.315 | 0.438 | 0.366 | 0.472 | 0.506 | 0.520 | 0.531 |
| 1.380 | 0.176 | 0.211 | 0.311 | 0.414 | 0.165 | 0.183 | 0.275 | 0.392 | 0.271 | 0.316 | 0.428 | 0.371 | 0.472 | 0.507 | 0.520 | 0.531 |
| 1.390 | 0.168 | 0.218 | 0.308 | 0.404 | 0.159 | 0.188 | 0.274 | 0.390 | 0.270 | 0.318 | 0.425 | 0.369 | 0.472 | 0.507 | 0.520 | 0.531 |
| 1.400 | 0.161 | 0.224 | 0.305 | 0.403 | 0.153 | 0.191 | 0.273 | 0.389 | 0.267 | 0.317 | 0.423 | 0.370 | 0.472 | 0.508 | 0.521 | 0.532 |
| 1.410 | 0.155 | 0.226 | 0.307 | 0.406 | 0.153 | 0.189 | 0.273 | 0.398 | 0.266 | 0.319 | 0.424 | 0.374 | 0.472 | 0.508 | 0.520 | 0.532 |
| 1.420 | 0.147 | 0.228 | 0.314 | 0.408 | 0.144 | 0.183 | 0.279 | 0.399 | 0.265 | 0.318 | 0.423 | 0.375 | 0.472 | 0.508 | 0.521 | 0.533 |
| 1.430 | 0.145 | 0.223 | 0.320 | 0.399 | 0.142 | 0.177 | 0.281 | 0.395 | 0.264 | 0.318 | 0.425 | 0.375 | 0.472 | 0.509 | 0.522 | 0.533 |
| 1.430 | 0.143 | 0.219 | 0.325 | 0.391 | 0.142 | 0.172 | 0.284 | 0.391 | 0.263 | 0.317 | 0.427 | 0.374 | 0.472 | 0.509 | 0.522 | 0.533 |
| 1.440 | 0.137 | 0.211 | 0.322 | 0.391 | 0.138 | 0.167 | 0.284 | 0.395 | 0.261 | 0.317 | 0.422 | 0.377 | 0.472 | 0.509 | 0.522 | 0.533 |
| 1.450 | 0.136 | 0.206 | 0.317 | 0.396 | 0.133 | 0.166 | 0.276 | 0.399 | 0.261 | 0.316 | 0.418 | 0.380 | 0.472 | 0.509 | 0.523 | 0.534 |
| 1.460 | 0.132 | 0.203 | 0.313 | 0.389 | 0.130 | 0.171 | 0.271 | 0.396 | 0.258 | 0.316 | 0.412 | 0.380 | 0.472 | 0.509 | 0.523 | 0.534 |
| 1.470 | 0.130 | 0.201 | 0.310 | 0.384 | 0.127 | 0.168 | 0.272 | 0.387 | 0.258 | 0.317 | 0.405 | 0.376 | 0.472 | 0.509 | 0.524 | 0.534 |
| 1.480 | 0.126 | 0.201 | 0.307 | 0.378 | 0.119 | 0.167 | 0.269 | 0.382 | 0.257 | 0.316 | 0.405 | 0.375 | 0.471 | 0.509 | 0.523 | 0.534 |
| 1.490 | 0.120 | 0.197 | 0.306 | 0.369 | 0.113 | 0.167 | 0.269 | 0.379 | 0.257 | 0.315 | 0.401 | 0.372 | 0.471 | 0.509 | 0.522 | 0.534 |
| 1.500 | 0.118 | 0.198 | 0.299 | 0.366 | 0.111 | 0.169 | 0.268 | 0.372 | 0.256 | 0.316 | 0.398 | 0.373 | 0.471 | 0.509 | 0.522 | 0.534 |
| 1.500 | 0.116 | 0.197 | 0.293 | 0.363 | 0.110 | 0.171 | 0.267 | 0.370 | 0.254 | 0.315 | 0.395 | 0.374 | 0.471 | 0.509 | 0.521 | 0.534 |
| 1.510 | 0.105 | 0.196 | 0.289 | 0.359 | 0.099 | 0.169 | 0.266 | 0.371 | 0.252 | 0.313 | 0.388 | 0.376 | 0.471 | 0.509 | 0.522 | 0.534 |
| 1.520 | 0.097 | 0.197 | 0.290 | 0.353 | 0.093 | 0.167 | 0.265 | 0.369 | 0.250 | 0.311 | 0.382 | 0.375 | 0.470 | 0.508 | 0.523 | 0.534 |
| 1.530 | 0.091 | 0.198 | 0.288 | 0.351 | 0.086 | 0.171 | 0.261 | 0.366 | 0.249 | 0.312 | 0.382 | 0.373 | 0.470 | 0.508 | 0.521 | 0.534 |
| 1.540 | 0.089 | 0.199 | 0.281 | 0.353 | 0.083 | 0.169 | 0.259 | 0.365 | 0.248 | 0.310 | 0.379 | 0.374 | 0.470 | 0.508 | 0.522 | 0.534 |
| 1.550 | 0.085 | 0.202 | 0.279 | 0.351 | 0.077 | 0.171 | 0.259 | 0.364 | 0.247 | 0.310 | 0.380 | 0.372 | 0.469 | 0.508 | 0.522 | 0.534 |
| 1.560 | 0.081 | 0.204 | 0.282 | 0.350 | 0.073 | 0.173 | 0.265 | 0.365 | 0.245 | 0.309 | 0.379 | 0.370 | 0.469 | 0.508 | 0.522 | 0.534 |
| 1.560 | 0.078 | 0.206 | 0.279 | 0.349 | 0.072 | 0.173 | 0.264 | 0.366 | 0.245 | 0.308 | 0.377 | 0.369 | 0.469 | 0.507 | 0.522 | 0.534 |
| 1.570 | 0.068 | 0.204 | 0.281 | 0.338 | 0.063 | 0.173 | 0.264 | 0.360 | 0.243 | 0.307 | 0.377 | 0.369 | 0.468 | 0.507 | 0.521 | 0.534 |
| 1.580 | 0.064 | 0.207 | 0.282 | 0.334 | 0.059 | 0.177 | 0.265 | 0.359 | 0.243 | 0.306 | 0.376 | 0.371 | 0.468 | 0.507 | 0.520 | 0.534 |
| 1.590 | 0.059 | 0.205 | 0.281 | 0.339 | 0.055 | 0.177 | 0.260 | 0.358 | 0.242 | 0.306 | 0.375 | 0.369 | 0.467 | 0.507 | 0.520 | 0.534 |
| 1.600 | 0.055 | 0.206 | 0.278 | 0.335 | 0.050 | 0.178 | 0.257 | 0.356 | 0.241 | 0.304 | 0.375 | 0.366 | 0.467 | 0.506 | 0.521 | 0.534 |
| 1.610 | 0.052 | 0.206 | 0.277 | 0.332 | 0.048 | 0.178 | 0.259 | 0.353 | 0.239 | 0.304 | 0.372 | 0.367 | 0.467 | 0.506 | 0.521 | 0.534 |
| 1.620 | 0.049 | 0.210 | 0.277 | 0.332 | 0.045 | 0.178 | 0.263 | 0.347 | 0.238 | 0.305 | 0.369 | 0.366 | 0.466 | 0.506 | 0.521 | 0.534 |
| 1.620 | 0.047 | 0.209 | 0.277 | 0.332 | 0.042 | 0.180 | 0.265 | 0.349 | 0.237 | 0.304 | 0.367 | 0.365 | 0.466 | 0.506 | 0.521 | 0.534 |
| 1.630 | 0.045 | 0.211 | 0.276 | 0.324 | 0.041 | 0.181 | 0.263 | 0.346 | 0.235 | 0.302 | 0.368 | 0.361 | 0.466 | 0.505 | 0.519 | 0.534 |
| 1.640 | 0.044 | 0.211 | 0.274 | 0.323 | 0.040 | 0.181 | 0.256 | 0.345 | 0.235 | 0.302 | 0.368 | 0.359 | 0.465 | 0.505 | 0.519 | 0.533 |
| 1.650 | 0.043 | 0.211 | 0.275 | 0.319 | 0.039 | 0.183 | 0.259 | 0.343 | 0.235 | 0.301 | 0.366 | 0.357 | 0.465 | 0.505 | 0.519 | 0.534 |
| 1.660 | 0.043 | 0.210 | 0.266 | 0.316 | 0.039 | 0.183 | 0.264 | 0.345 | 0.234 | 0.302 | 0.364 | 0.356 | 0.465 | 0.505 | 0.519 | 0.533 |
| 1.670 | 0.044 | 0.210 | 0.270 | 0.315 | 0.039 | 0.182 | 0.262 | 0.342 | 0.232 | 0.301 | 0.363 | 0.354 | 0.465 | 0.504 | 0.519 | 0.533 |
| 1.680 | 0.045 | 0.211 | 0.269 | 0.314 | 0.041 | 0.183 | 0.259 | 0.337 | 0.230 | 0.299 | 0.363 | 0.353 | 0.464 | 0.504 | 0.519 | 0.533 |
| 1.690 | 0.046 | 0.210 | 0.264 | 0.311 | 0.043 | 0.186 | 0.255 | 0.338 | 0.230 | 0.297 | 0.362 | 0.349 | 0.464 | 0.504 | 0.519 | 0.533 |
| 1.690 | 0.048 | 0.210 | 0.260 | 0.308 | 0.045 | 0.186 | 0.253 | 0.337 | 0.230 | 0.298 | 0.362 | 0.346 | 0.464 | 0.504 | 0.518 | 0.533 |
| 1.700 | 0.051 | 0.211 | 0.258 | 0.307 | 0.048 | 0.187 | 0.252 | 0.335 | 0.228 | 0.296 | 0.355 | 0.342 | 0.464 | 0.504 | 0.518 | 0.533 |
| 1.710 | 0.055 | 0.211 | 0.256 | 0.303 | 0.052 | 0.188 | 0.250 | 0.332 | 0.225 | 0.296 | 0.354 | 0.342 | 0.464 | 0.504 | 0.519 | 0.533 |
| 1.720 | 0.058 | 0.209 | 0.254 | 0.299 | 0.056 | 0.193 | 0.246 | 0.330 | 0.223 | 0.295 | 0.354 | 0.339 | 0.464 | 0.504 | 0.518 | 0.533 |

**Table. 7** Under two different submerged conditions, the streamwise velocity *u* of three vegetation patch coverage conditions changes along the *y* direction.

| **Hydraulic parameters** | **Streamwise velocity *u* (m/s)** | | | | | | | | | | | |
| --- | --- | --- | --- | --- | --- | --- | --- | --- | --- | --- | --- | --- |
| ***y* (m)** | **Non-submerged state (*h*=0.05 m)** | | | | | | **Submerged state (*h*=0.09 m)** | | | | | |
|  | **C_2-2_** | | | **C_3-3_** | | | **C_2-2_** | | | **C_3-3_** | | |
|  | ***Cr*=**  **2.09%** | ***Cr*=**  **4.71%** | ***Cr*=**  **8.37%** | ***Cr*=**  **2.09%** | ***Cr*=**  **4.71%** | ***Cr*=**  **8.37%** | ***Cr*=**  **2.09%** | ***Cr*=**  **4.71%** | ***Cr*=**  **8.37%** | ***Cr*=**  **2.09%** | ***Cr*=**  **4.71%** | ***Cr*=**  **8.37%** |
| 0.000 | 0.000 | 0.000 | 0.000 | 0.000 | 0.000 | 0.000 | 0.000 | 0.000 | 0.000 | 0.000 | 0.000 | 0.000 |
| 0.004 | 0.310 | 0.316 | 0.189 | 0.304 | 0.314 | 0.190 | 0.159 | 0.233 | 0.240 | 0.195 | 0.174 | 0.217 |
| 0.008 | 0.362 | 0.424 | 0.378 | 0.355 | 0.418 | 0.379 | 0.308 | 0.366 | 0.392 | 0.319 | 0.356 | 0.398 |
| 0.012 | 0.392 | 0.457 | 0.475 | 0.383 | 0.446 | 0.476 | 0.335 | 0.387 | 0.414 | 0.332 | 0.384 | 0.414 |
| 0.016 | 0.405 | 0.474 | 0.489 | 0.393 | 0.460 | 0.487 | 0.351 | 0.404 | 0.429 | 0.343 | 0.396 | 0.429 |
| 0.020 | 0.410 | 0.480 | 0.503 | 0.396 | 0.464 | 0.499 | 0.359 | 0.411 | 0.438 | 0.351 | 0.402 | 0.433 |
| 0.024 | 0.411 | 0.481 | 0.508 | 0.396 | 0.465 | 0.503 | 0.364 | 0.417 | 0.446 | 0.356 | 0.406 | 0.438 |
| 0.028 | 0.411 | 0.481 | 0.514 | 0.396 | 0.465 | 0.507 | 0.368 | 0.419 | 0.448 | 0.358 | 0.408 | 0.440 |
| 0.032 | 0.411 | 0.481 | 0.516 | 0.396 | 0.465 | 0.509 | 0.371 | 0.421 | 0.450 | 0.360 | 0.409 | 0.441 |
| 0.036 | 0.411 | 0.481 | 0.517 | 0.396 | 0.465 | 0.509 | 0.372 | 0.422 | 0.450 | 0.361 | 0.410 | 0.441 |
| 0.040 | 0.411 | 0.481 | 0.517 | 0.396 | 0.465 | 0.510 | 0.372 | 0.422 | 0.451 | 0.362 | 0.410 | 0.442 |
| 0.044 | 0.411 | 0.482 | 0.518 | 0.396 | 0.465 | 0.510 | 0.372 | 0.422 | 0.451 | 0.362 | 0.411 | 0.442 |
| 0.049 | 0.411 | 0.482 | 0.518 | 0.396 | 0.465 | 0.510 | 0.372 | 0.422 | 0.451 | 0.362 | 0.411 | 0.442 |
| 0.053 | 0.411 | 0.482 | 0.518 | 0.396 | 0.465 | 0.510 | 0.372 | 0.422 | 0.451 | 0.362 | 0.411 | 0.442 |
| 0.057 | 0.411 | 0.482 | 0.518 | 0.396 | 0.465 | 0.511 | 0.372 | 0.423 | 0.451 | 0.362 | 0.411 | 0.442 |
| 0.061 | 0.411 | 0.482 | 0.519 | 0.396 | 0.466 | 0.511 | 0.372 | 0.423 | 0.451 | 0.362 | 0.411 | 0.442 |
| 0.065 | 0.411 | 0.482 | 0.519 | 0.396 | 0.466 | 0.511 | 0.372 | 0.423 | 0.451 | 0.362 | 0.411 | 0.442 |
| 0.069 | 0.411 | 0.483 | 0.519 | 0.396 | 0.466 | 0.512 | 0.372 | 0.423 | 0.451 | 0.362 | 0.411 | 0.443 |
| 0.073 | 0.412 | 0.483 | 0.519 | 0.396 | 0.466 | 0.512 | 0.372 | 0.423 | 0.452 | 0.362 | 0.411 | 0.443 |
| 0.077 | 0.412 | 0.483 | 0.520 | 0.396 | 0.466 | 0.512 | 0.372 | 0.423 | 0.452 | 0.362 | 0.411 | 0.443 |
| 0.081 | 0.412 | 0.483 | 0.520 | 0.396 | 0.467 | 0.513 | 0.373 | 0.423 | 0.452 | 0.362 | 0.411 | 0.443 |
| 0.085 | 0.412 | 0.484 | 0.521 | 0.396 | 0.467 | 0.513 | 0.373 | 0.423 | 0.452 | 0.362 | 0.412 | 0.443 |
| 0.089 | 0.412 | 0.484 | 0.521 | 0.396 | 0.467 | 0.514 | 0.373 | 0.423 | 0.452 | 0.362 | 0.412 | 0.444 |
| 0.093 | 0.412 | 0.484 | 0.521 | 0.396 | 0.467 | 0.514 | 0.373 | 0.424 | 0.453 | 0.362 | 0.412 | 0.444 |
| 0.097 | 0.412 | 0.484 | 0.522 | 0.396 | 0.468 | 0.514 | 0.373 | 0.424 | 0.453 | 0.362 | 0.412 | 0.444 |
| 0.101 | 0.412 | 0.485 | 0.522 | 0.396 | 0.468 | 0.515 | 0.373 | 0.424 | 0.453 | 0.362 | 0.412 | 0.444 |
| 0.105 | 0.413 | 0.485 | 0.523 | 0.396 | 0.468 | 0.515 | 0.373 | 0.424 | 0.453 | 0.362 | 0.412 | 0.445 |
| 0.109 | 0.413 | 0.485 | 0.523 | 0.397 | 0.468 | 0.516 | 0.373 | 0.424 | 0.454 | 0.362 | 0.413 | 0.445 |
| 0.113 | 0.413 | 0.486 | 0.523 | 0.397 | 0.469 | 0.516 | 0.373 | 0.425 | 0.454 | 0.362 | 0.413 | 0.445 |
| 0.117 | 0.413 | 0.486 | 0.524 | 0.397 | 0.469 | 0.517 | 0.373 | 0.425 | 0.454 | 0.362 | 0.413 | 0.446 |
| 0.121 | 0.414 | 0.486 | 0.524 | 0.397 | 0.469 | 0.517 | 0.374 | 0.425 | 0.454 | 0.362 | 0.413 | 0.446 |
| 0.125 | 0.414 | 0.487 | 0.525 | 0.397 | 0.470 | 0.518 | 0.374 | 0.425 | 0.455 | 0.362 | 0.413 | 0.446 |
| 0.129 | 0.414 | 0.487 | 0.525 | 0.397 | 0.470 | 0.518 | 0.374 | 0.426 | 0.455 | 0.362 | 0.414 | 0.447 |
| 0.133 | 0.414 | 0.488 | 0.526 | 0.397 | 0.470 | 0.519 | 0.374 | 0.426 | 0.455 | 0.362 | 0.414 | 0.447 |
| 0.137 | 0.415 | 0.488 | 0.527 | 0.397 | 0.470 | 0.520 | 0.374 | 0.426 | 0.456 | 0.362 | 0.414 | 0.447 |
| 0.141 | 0.415 | 0.489 | 0.527 | 0.397 | 0.471 | 0.521 | 0.374 | 0.426 | 0.456 | 0.362 | 0.414 | 0.448 |
| 0.145 | 0.415 | 0.489 | 0.527 | 0.397 | 0.471 | 0.521 | 0.375 | 0.427 | 0.456 | 0.362 | 0.414 | 0.448 |
| 0.149 | 0.416 | 0.490 | 0.527 | 0.397 | 0.471 | 0.522 | 0.375 | 0.427 | 0.456 | 0.362 | 0.415 | 0.449 |
| 0.154 | 0.416 | 0.489 | 0.519 | 0.397 | 0.472 | 0.519 | 0.375 | 0.427 | 0.456 | 0.361 | 0.415 | 0.449 |
| 0.158 | 0.416 | 0.485 | 0.510 | 0.397 | 0.472 | 0.516 | 0.375 | 0.428 | 0.454 | 0.361 | 0.415 | 0.449 |
| 0.162 | 0.417 | 0.474 | 0.496 | 0.397 | 0.472 | 0.507 | 0.376 | 0.428 | 0.450 | 0.361 | 0.415 | 0.449 |
| 0.166 | 0.417 | 0.457 | 0.473 | 0.397 | 0.469 | 0.489 | 0.376 | 0.428 | 0.444 | 0.361 | 0.415 | 0.449 |
| 0.170 | 0.418 | 0.437 | 0.451 | 0.397 | 0.458 | 0.471 | 0.376 | 0.428 | 0.432 | 0.361 | 0.415 | 0.447 |
| 0.174 | 0.418 | 0.416 | 0.424 | 0.397 | 0.437 | 0.441 | 0.376 | 0.428 | 0.420 | 0.361 | 0.415 | 0.445 |
| 0.178 | 0.414 | 0.394 | 0.396 | 0.397 | 0.412 | 0.409 | 0.377 | 0.426 | 0.397 | 0.361 | 0.415 | 0.437 |
| 0.182 | 0.404 | 0.372 | 0.368 | 0.396 | 0.385 | 0.376 | 0.377 | 0.425 | 0.374 | 0.361 | 0.414 | 0.421 |
| 0.186 | 0.391 | 0.350 | 0.340 | 0.395 | 0.358 | 0.344 | 0.377 | 0.421 | 0.355 | 0.361 | 0.413 | 0.401 |
| 0.190 | 0.376 | 0.326 | 0.312 | 0.385 | 0.333 | 0.312 | 0.377 | 0.416 | 0.336 | 0.360 | 0.411 | 0.377 |
| 0.194 | 0.361 | 0.303 | 0.284 | 0.368 | 0.309 | 0.284 | 0.377 | 0.408 | 0.319 | 0.360 | 0.408 | 0.353 |
| 0.198 | 0.344 | 0.280 | 0.256 | 0.350 | 0.287 | 0.256 | 0.377 | 0.397 | 0.309 | 0.360 | 0.403 | 0.334 |
| 0.202 | 0.327 | 0.257 | 0.231 | 0.333 | 0.266 | 0.229 | 0.376 | 0.383 | 0.299 | 0.359 | 0.398 | 0.319 |
| 0.206 | 0.310 | 0.237 | 0.207 | 0.317 | 0.244 | 0.204 | 0.375 | 0.367 | 0.290 | 0.358 | 0.390 | 0.303 |
| 0.210 | 0.293 | 0.218 | 0.184 | 0.302 | 0.222 | 0.179 | 0.373 | 0.355 | 0.282 | 0.357 | 0.379 | 0.292 |
| 0.214 | 0.279 | 0.202 | 0.162 | 0.285 | 0.197 | 0.155 | 0.370 | 0.341 | 0.276 | 0.356 | 0.366 | 0.285 |
| 0.218 | 0.267 | 0.186 | 0.143 | 0.264 | 0.170 | 0.130 | 0.367 | 0.327 | 0.269 | 0.353 | 0.352 | 0.285 |
| 0.222 | 0.257 | 0.170 | 0.127 | 0.238 | 0.143 | 0.108 | 0.362 | 0.314 | 0.263 | 0.350 | 0.338 | 0.283 |
| 0.226 | 0.243 | 0.150 | 0.113 | 0.210 | 0.118 | 0.086 | 0.358 | 0.306 | 0.256 | 0.346 | 0.324 | 0.283 |
| 0.230 | 0.223 | 0.125 | 0.099 | 0.183 | 0.096 | 0.064 | 0.355 | 0.299 | 0.249 | 0.343 | 0.313 | 0.288 |
| 0.234 | 0.181 | 0.097 | 0.079 | 0.162 | 0.079 | 0.048 | 0.350 | 0.294 | 0.242 | 0.336 | 0.302 | 0.287 |
| 0.238 | 0.122 | 0.054 | 0.044 | 0.151 | 0.067 | 0.033 | 0.346 | 0.291 | 0.235 | 0.332 | 0.294 | 0.291 |
| 0.242 | 0.071 | 0.009 | 0.016 | 0.152 | 0.059 | 0.022 | 0.343 | 0.289 | 0.226 | 0.331 | 0.289 | 0.288 |
| 0.246 | 0.062 | 0.012 | 0.008 | 0.163 | 0.055 | 0.015 | 0.342 | 0.290 | 0.217 | 0.330 | 0.293 | 0.283 |
| 0.251 | 0.127 | 0.073 | 0.019 | 0.182 | 0.053 | 0.009 | 0.343 | 0.292 | 0.208 | 0.331 | 0.297 | 0.275 |
| 0.255 | 0.198 | 0.106 | 0.022 | 0.201 | 0.054 | 0.007 | 0.344 | 0.293 | 0.201 | 0.331 | 0.299 | 0.265 |
| 0.259 | 0.229 | 0.087 | 0.008 | 0.209 | 0.055 | 0.005 | 0.344 | 0.293 | 0.195 | 0.332 | 0.302 | 0.253 |
| 0.263 | 0.236 | 0.038 | 0.006 | 0.206 | 0.056 | 0.005 | 0.344 | 0.292 | 0.188 | 0.332 | 0.306 | 0.242 |
| 0.267 | 0.221 | 0.007 | 0.029 | 0.193 | 0.058 | 0.005 | 0.347 | 0.295 | 0.182 | 0.331 | 0.308 | 0.229 |
| 0.271 | 0.183 | 0.053 | 0.022 | 0.178 | 0.059 | 0.005 | 0.344 | 0.295 | 0.178 | 0.328 | 0.311 | 0.216 |
| 0.275 | 0.122 | 0.112 | 0.006 | 0.170 | 0.060 | 0.006 | 0.345 | 0.291 | 0.176 | 0.325 | 0.311 | 0.206 |
| 0.279 | 0.061 | 0.108 | 0.016 | 0.169 | 0.063 | 0.007 | 0.346 | 0.288 | 0.173 | 0.327 | 0.307 | 0.201 |
| 0.283 | 0.066 | 0.050 | 0.032 | 0.175 | 0.067 | 0.008 | 0.348 | 0.284 | 0.171 | 0.330 | 0.301 | 0.195 |
| 0.287 | 0.149 | -0.002 | 0.017 | 0.191 | 0.071 | 0.009 | 0.344 | 0.279 | 0.171 | 0.333 | 0.296 | 0.197 |
| 0.291 | 0.217 | 0.020 | 0.006 | 0.210 | 0.077 | 0.010 | 0.344 | 0.277 | 0.174 | 0.335 | 0.284 | 0.197 |
| 0.295 | 0.246 | 0.123 | 0.024 | 0.224 | 0.082 | 0.011 | 0.344 | 0.274 | 0.176 | 0.336 | 0.281 | 0.196 |
| 0.299 | 0.252 | 0.124 | 0.033 | 0.225 | 0.089 | 0.012 | 0.345 | 0.270 | 0.178 | 0.332 | 0.281 | 0.195 |
| 0.303 | 0.242 | 0.080 | 0.009 | 0.216 | 0.095 | 0.012 | 0.343 | 0.269 | 0.184 | 0.327 | 0.280 | 0.196 |
| 0.307 | 0.207 | 0.025 | 0.009 | 0.200 | 0.102 | 0.013 | 0.344 | 0.270 | 0.191 | 0.324 | 0.278 | 0.203 |
| 0.311 | 0.139 | 0.024 | 0.034 | 0.186 | 0.108 | 0.014 | 0.343 | 0.271 | 0.198 | 0.323 | 0.283 | 0.213 |
| 0.315 | 0.068 | 0.105 | 0.034 | 0.179 | 0.114 | 0.015 | 0.347 | 0.273 | 0.206 | 0.325 | 0.287 | 0.224 |
| 0.319 | 0.081 | 0.137 | 0.006 | 0.180 | 0.117 | 0.016 | 0.349 | 0.279 | 0.218 | 0.328 | 0.292 | 0.237 |
| 0.323 | 0.165 | 0.109 | 0.006 | 0.190 | 0.119 | 0.018 | 0.349 | 0.288 | 0.235 | 0.330 | 0.303 | 0.253 |
| 0.327 | 0.225 | 0.039 | 0.047 | 0.207 | 0.119 | 0.021 | 0.349 | 0.299 | 0.251 | 0.331 | 0.317 | 0.269 |
| 0.331 | 0.262 | 0.015 | 0.036 | 0.226 | 0.118 | 0.027 | 0.351 | 0.314 | 0.266 | 0.333 | 0.332 | 0.285 |
| 0.335 | 0.269 | 0.092 | 0.001 | 0.238 | 0.119 | 0.040 | 0.352 | 0.336 | 0.283 | 0.332 | 0.348 | 0.303 |
| 0.339 | 0.253 | 0.157 | 0.025 | 0.238 | 0.124 | 0.052 | 0.354 | 0.360 | 0.304 | 0.334 | 0.365 | 0.320 |
| 0.343 | 0.212 | 0.152 | 0.089 | 0.230 | 0.136 | 0.082 | 0.356 | 0.376 | 0.323 | 0.335 | 0.375 | 0.337 |
| 0.347 | 0.140 | 0.099 | 0.046 | 0.218 | 0.155 | 0.114 | 0.357 | 0.388 | 0.340 | 0.338 | 0.384 | 0.350 |
| 0.352 | 0.069 | 0.007 | -0.017 | 0.212 | 0.180 | 0.153 | 0.361 | 0.397 | 0.362 | 0.341 | 0.385 | 0.368 |
| 0.356 | 0.092 | 0.071 | 0.064 | 0.214 | 0.211 | 0.202 | 0.365 | 0.401 | 0.386 | 0.344 | 0.383 | 0.390 |
| 0.360 | 0.192 | 0.233 | 0.251 | 0.226 | 0.248 | 0.252 | 0.370 | 0.403 | 0.414 | 0.348 | 0.381 | 0.412 |
| 0.364 | 0.269 | 0.301 | 0.313 | 0.250 | 0.290 | 0.299 | 0.375 | 0.403 | 0.435 | 0.350 | 0.383 | 0.429 |
| 0.368 | 0.311 | 0.344 | 0.350 | 0.282 | 0.332 | 0.346 | 0.377 | 0.404 | 0.450 | 0.352 | 0.385 | 0.441 |
| 0.372 | 0.339 | 0.382 | 0.382 | 0.315 | 0.372 | 0.385 | 0.377 | 0.407 | 0.456 | 0.354 | 0.391 | 0.445 |
| 0.376 | 0.361 | 0.413 | 0.410 | 0.345 | 0.407 | 0.414 | 0.374 | 0.410 | 0.457 | 0.350 | 0.393 | 0.446 |
| 0.380 | 0.381 | 0.438 | 0.431 | 0.368 | 0.434 | 0.442 | 0.370 | 0.410 | 0.456 | 0.345 | 0.392 | 0.443 |
| 0.384 | 0.394 | 0.454 | 0.446 | 0.380 | 0.449 | 0.452 | 0.364 | 0.409 | 0.451 | 0.337 | 0.393 | 0.436 |
| 0.388 | 0.397 | 0.454 | 0.455 | 0.377 | 0.444 | 0.460 | 0.351 | 0.402 | 0.442 | 0.320 | 0.382 | 0.429 |
| 0.392 | 0.376 | 0.433 | 0.462 | 0.347 | 0.420 | 0.376 | 0.301 | 0.379 | 0.423 | 0.298 | 0.366 | 0.317 |
| 0.396 | 0.328 | 0.328 | 0.246 | 0.294 | 0.316 | 0.188 | 0.177 | 0.242 | 0.248 | 0.160 | 0.223 | 0.157 |
| 0.400 | 0.000 | 0.000 | 0.000 | 0.000 | 0.000 | 0.000 | 0.000 | 0.000 | 0.000 | 0.000 | 0.000 | 0.000 |

**Table. 8** Under two different submerged conditions, the streamwise velocity *u* of four different fragmentation conditions changes along the *y* direction.

| **Hydraulic parameters** | **Streamwise velocity *u* (m/s)** | | | | | | | | | | | | | | | |
| --- | --- | --- | --- | --- | --- | --- | --- | --- | --- | --- | --- | --- | --- | --- | --- | --- |
| ***y* (m)** | **Non-submerged state (*h*=0.05 m)** | | | | | | | | **Submerged state (*h*=0.09 m)** | | | | | | | |
|  | **C_2-2_** | | | | **C_3-3_** | | | | **C_2-2_** | | | | **C_3-3_** | | | |
|  | **Fragmentation I** | **Fragmentation II** | **Fragmentation III** | **Fragmentation IV** | **Fragmentation I** | **Fragmentation II** | **Fragmentation III** | **Fragmentation IV** | **Fragmentation I** | **Fragmentation II** | **Fragmentation III** | **Fragmentation IV** | **Fragmentation I** | **Fragmentation II** | **Fragmentation III** | **Fragmentation IV** |
| 0.000 | 0.000 | 0.000 | 0.000 | 0.000 | 0.000 | 0.000 | 0.000 | 0.000 | 0.000 | 0.000 | 0.000 | 0.000 | 0.000 | 0.000 | 0.000 | 0.000 |
| 0.004 | 0.419 | 0.394 | 0.356 | 0.189 | 0.413 | 0.388 | 0.356 | 0.190 | 0.282 | 0.128 | 0.272 | 0.240 | 0.264 | 0.100 | 0.244 | 0.217 |
| 0.008 | 0.558 | 0.525 | 0.477 | 0.378 | 0.546 | 0.513 | 0.474 | 0.379 | 0.440 | 0.219 | 0.416 | 0.392 | 0.433 | 0.207 | 0.412 | 0.398 |
| 0.012 | 0.595 | 0.559 | 0.510 | 0.475 | 0.576 | 0.543 | 0.505 | 0.476 | 0.460 | 0.296 | 0.436 | 0.414 | 0.450 | 0.324 | 0.426 | 0.414 |
| 0.016 | 0.612 | 0.575 | 0.527 | 0.489 | 0.590 | 0.556 | 0.520 | 0.487 | 0.479 | 0.369 | 0.446 | 0.429 | 0.462 | 0.442 | 0.438 | 0.429 |
| 0.020 | 0.616 | 0.579 | 0.531 | 0.503 | 0.592 | 0.559 | 0.523 | 0.499 | 0.484 | 0.442 | 0.454 | 0.438 | 0.468 | 0.461 | 0.446 | 0.433 |
| 0.024 | 0.616 | 0.579 | 0.532 | 0.508 | 0.592 | 0.559 | 0.524 | 0.503 | 0.488 | 0.479 | 0.460 | 0.446 | 0.473 | 0.466 | 0.451 | 0.438 |
| 0.028 | 0.616 | 0.579 | 0.532 | 0.514 | 0.592 | 0.559 | 0.524 | 0.507 | 0.491 | 0.483 | 0.462 | 0.448 | 0.475 | 0.470 | 0.453 | 0.440 |
| 0.032 | 0.617 | 0.580 | 0.532 | 0.516 | 0.593 | 0.559 | 0.524 | 0.509 | 0.493 | 0.486 | 0.463 | 0.450 | 0.476 | 0.472 | 0.454 | 0.441 |
| 0.036 | 0.617 | 0.580 | 0.533 | 0.517 | 0.593 | 0.560 | 0.524 | 0.509 | 0.493 | 0.488 | 0.463 | 0.450 | 0.476 | 0.473 | 0.455 | 0.441 |
| 0.040 | 0.617 | 0.580 | 0.533 | 0.517 | 0.593 | 0.560 | 0.524 | 0.510 | 0.494 | 0.489 | 0.464 | 0.451 | 0.476 | 0.474 | 0.455 | 0.442 |
| 0.044 | 0.618 | 0.580 | 0.533 | 0.518 | 0.594 | 0.560 | 0.525 | 0.510 | 0.494 | 0.490 | 0.464 | 0.451 | 0.476 | 0.475 | 0.455 | 0.442 |
| 0.049 | 0.618 | 0.581 | 0.533 | 0.518 | 0.594 | 0.561 | 0.525 | 0.510 | 0.494 | 0.490 | 0.464 | 0.451 | 0.476 | 0.475 | 0.455 | 0.442 |
| 0.053 | 0.619 | 0.581 | 0.534 | 0.518 | 0.595 | 0.561 | 0.525 | 0.510 | 0.494 | 0.490 | 0.464 | 0.451 | 0.476 | 0.475 | 0.455 | 0.442 |
| 0.057 | 0.619 | 0.582 | 0.534 | 0.518 | 0.595 | 0.561 | 0.525 | 0.511 | 0.494 | 0.491 | 0.464 | 0.451 | 0.477 | 0.476 | 0.455 | 0.442 |
| 0.061 | 0.620 | 0.582 | 0.534 | 0.519 | 0.596 | 0.562 | 0.526 | 0.511 | 0.495 | 0.491 | 0.465 | 0.451 | 0.477 | 0.476 | 0.456 | 0.442 |
| 0.065 | 0.620 | 0.582 | 0.534 | 0.519 | 0.596 | 0.562 | 0.526 | 0.511 | 0.495 | 0.492 | 0.465 | 0.451 | 0.477 | 0.476 | 0.456 | 0.442 |
| 0.069 | 0.621 | 0.583 | 0.535 | 0.519 | 0.597 | 0.563 | 0.526 | 0.512 | 0.495 | 0.492 | 0.465 | 0.451 | 0.477 | 0.477 | 0.456 | 0.443 |
| 0.073 | 0.621 | 0.583 | 0.535 | 0.519 | 0.598 | 0.563 | 0.527 | 0.512 | 0.496 | 0.492 | 0.465 | 0.452 | 0.478 | 0.477 | 0.456 | 0.443 |
| 0.077 | 0.622 | 0.584 | 0.536 | 0.520 | 0.598 | 0.564 | 0.527 | 0.512 | 0.496 | 0.492 | 0.465 | 0.452 | 0.478 | 0.477 | 0.456 | 0.443 |
| 0.081 | 0.623 | 0.584 | 0.536 | 0.520 | 0.599 | 0.564 | 0.528 | 0.513 | 0.496 | 0.493 | 0.466 | 0.452 | 0.479 | 0.477 | 0.457 | 0.443 |
| 0.085 | 0.623 | 0.585 | 0.537 | 0.521 | 0.600 | 0.565 | 0.528 | 0.513 | 0.496 | 0.493 | 0.466 | 0.452 | 0.479 | 0.478 | 0.457 | 0.443 |
| 0.089 | 0.624 | 0.586 | 0.537 | 0.521 | 0.600 | 0.565 | 0.529 | 0.514 | 0.497 | 0.493 | 0.466 | 0.452 | 0.479 | 0.478 | 0.457 | 0.444 |
| 0.093 | 0.625 | 0.586 | 0.537 | 0.521 | 0.601 | 0.566 | 0.529 | 0.514 | 0.497 | 0.494 | 0.466 | 0.453 | 0.479 | 0.478 | 0.457 | 0.444 |
| 0.097 | 0.626 | 0.587 | 0.538 | 0.522 | 0.602 | 0.567 | 0.530 | 0.514 | 0.498 | 0.494 | 0.467 | 0.453 | 0.480 | 0.479 | 0.458 | 0.444 |
| 0.101 | 0.628 | 0.587 | 0.538 | 0.522 | 0.603 | 0.567 | 0.530 | 0.515 | 0.498 | 0.495 | 0.467 | 0.453 | 0.480 | 0.479 | 0.458 | 0.444 |
| 0.105 | 0.628 | 0.589 | 0.539 | 0.523 | 0.605 | 0.568 | 0.531 | 0.515 | 0.498 | 0.495 | 0.467 | 0.453 | 0.481 | 0.480 | 0.458 | 0.445 |
| 0.109 | 0.627 | 0.589 | 0.539 | 0.523 | 0.606 | 0.569 | 0.531 | 0.516 | 0.499 | 0.495 | 0.468 | 0.454 | 0.481 | 0.480 | 0.459 | 0.445 |
| 0.113 | 0.620 | 0.589 | 0.540 | 0.523 | 0.605 | 0.570 | 0.531 | 0.516 | 0.499 | 0.495 | 0.468 | 0.454 | 0.482 | 0.481 | 0.459 | 0.445 |
| 0.117 | 0.606 | 0.586 | 0.540 | 0.524 | 0.599 | 0.570 | 0.532 | 0.517 | 0.500 | 0.494 | 0.469 | 0.454 | 0.482 | 0.480 | 0.459 | 0.446 |
| 0.121 | 0.587 | 0.577 | 0.541 | 0.524 | 0.588 | 0.569 | 0.532 | 0.517 | 0.500 | 0.493 | 0.469 | 0.454 | 0.483 | 0.480 | 0.460 | 0.446 |
| 0.125 | 0.565 | 0.562 | 0.542 | 0.525 | 0.569 | 0.562 | 0.533 | 0.518 | 0.500 | 0.492 | 0.469 | 0.455 | 0.483 | 0.479 | 0.460 | 0.446 |
| 0.129 | 0.542 | 0.541 | 0.543 | 0.525 | 0.545 | 0.547 | 0.534 | 0.518 | 0.499 | 0.488 | 0.470 | 0.455 | 0.483 | 0.477 | 0.460 | 0.447 |
| 0.133 | 0.520 | 0.519 | 0.542 | 0.526 | 0.521 | 0.526 | 0.534 | 0.519 | 0.498 | 0.484 | 0.470 | 0.455 | 0.483 | 0.474 | 0.461 | 0.447 |
| 0.137 | 0.498 | 0.496 | 0.535 | 0.527 | 0.497 | 0.502 | 0.533 | 0.520 | 0.496 | 0.480 | 0.470 | 0.456 | 0.482 | 0.470 | 0.461 | 0.447 |
| 0.141 | 0.476 | 0.472 | 0.522 | 0.527 | 0.473 | 0.477 | 0.526 | 0.521 | 0.490 | 0.477 | 0.470 | 0.456 | 0.481 | 0.469 | 0.461 | 0.448 |
| 0.145 | 0.454 | 0.448 | 0.501 | 0.527 | 0.450 | 0.451 | 0.511 | 0.521 | 0.484 | 0.470 | 0.470 | 0.456 | 0.477 | 0.465 | 0.461 | 0.448 |
| 0.149 | 0.432 | 0.424 | 0.476 | 0.527 | 0.428 | 0.426 | 0.487 | 0.522 | 0.476 | 0.463 | 0.469 | 0.456 | 0.472 | 0.459 | 0.461 | 0.449 |
| 0.154 | 0.410 | 0.400 | 0.450 | 0.519 | 0.406 | 0.402 | 0.460 | 0.519 | 0.465 | 0.457 | 0.465 | 0.456 | 0.469 | 0.453 | 0.460 | 0.449 |
| 0.158 | 0.388 | 0.377 | 0.423 | 0.510 | 0.384 | 0.379 | 0.431 | 0.516 | 0.455 | 0.452 | 0.460 | 0.454 | 0.462 | 0.448 | 0.458 | 0.449 |
| 0.162 | 0.365 | 0.353 | 0.395 | 0.496 | 0.362 | 0.356 | 0.400 | 0.507 | 0.443 | 0.449 | 0.455 | 0.450 | 0.451 | 0.442 | 0.454 | 0.449 |
| 0.166 | 0.341 | 0.328 | 0.366 | 0.473 | 0.340 | 0.332 | 0.369 | 0.489 | 0.435 | 0.441 | 0.446 | 0.444 | 0.441 | 0.438 | 0.446 | 0.449 |
| 0.170 | 0.318 | 0.304 | 0.337 | 0.451 | 0.317 | 0.309 | 0.337 | 0.471 | 0.427 | 0.436 | 0.429 | 0.432 | 0.432 | 0.434 | 0.435 | 0.447 |
| 0.174 | 0.295 | 0.280 | 0.307 | 0.424 | 0.295 | 0.285 | 0.306 | 0.441 | 0.421 | 0.427 | 0.412 | 0.420 | 0.422 | 0.429 | 0.420 | 0.445 |
| 0.178 | 0.271 | 0.257 | 0.278 | 0.396 | 0.272 | 0.259 | 0.276 | 0.409 | 0.420 | 0.422 | 0.395 | 0.397 | 0.413 | 0.425 | 0.406 | 0.437 |
| 0.182 | 0.248 | 0.235 | 0.251 | 0.368 | 0.248 | 0.233 | 0.247 | 0.376 | 0.420 | 0.418 | 0.379 | 0.374 | 0.414 | 0.416 | 0.391 | 0.421 |
| 0.186 | 0.226 | 0.214 | 0.226 | 0.340 | 0.224 | 0.212 | 0.221 | 0.344 | 0.422 | 0.414 | 0.364 | 0.355 | 0.413 | 0.411 | 0.376 | 0.401 |
| 0.190 | 0.203 | 0.193 | 0.203 | 0.312 | 0.200 | 0.195 | 0.197 | 0.312 | 0.421 | 0.416 | 0.350 | 0.336 | 0.412 | 0.407 | 0.361 | 0.377 |
| 0.194 | 0.179 | 0.172 | 0.184 | 0.284 | 0.177 | 0.175 | 0.178 | 0.284 | 0.415 | 0.417 | 0.338 | 0.319 | 0.418 | 0.404 | 0.351 | 0.353 |
| 0.198 | 0.154 | 0.154 | 0.168 | 0.256 | 0.152 | 0.151 | 0.162 | 0.256 | 0.408 | 0.422 | 0.325 | 0.309 | 0.417 | 0.407 | 0.338 | 0.334 |
| 0.202 | 0.134 | 0.144 | 0.153 | 0.231 | 0.115 | 0.119 | 0.147 | 0.229 | 0.391 | 0.433 | 0.318 | 0.299 | 0.415 | 0.418 | 0.323 | 0.319 |
| 0.206 | 0.089 | 0.122 | 0.138 | 0.207 | 0.069 | 0.079 | 0.131 | 0.204 | 0.372 | 0.443 | 0.312 | 0.290 | 0.415 | 0.430 | 0.311 | 0.303 |
| 0.210 | -0.002 | 0.042 | 0.122 | 0.184 | 0.051 | 0.042 | 0.112 | 0.179 | 0.358 | 0.453 | 0.306 | 0.282 | 0.405 | 0.439 | 0.301 | 0.292 |
| 0.214 | -0.002 | 0.035 | 0.101 | 0.162 | 0.083 | 0.022 | 0.089 | 0.155 | 0.350 | 0.448 | 0.301 | 0.276 | 0.387 | 0.444 | 0.293 | 0.285 |
| 0.218 | 0.122 | 0.092 | 0.067 | 0.143 | 0.123 | 0.034 | 0.066 | 0.130 | 0.347 | 0.442 | 0.299 | 0.269 | 0.375 | 0.433 | 0.289 | 0.285 |
| 0.222 | 0.158 | 0.139 | 0.026 | 0.127 | 0.135 | 0.057 | 0.046 | 0.108 | 0.352 | 0.431 | 0.303 | 0.263 | 0.367 | 0.422 | 0.290 | 0.283 |
| 0.226 | 0.144 | 0.079 | 0.018 | 0.113 | 0.122 | 0.088 | 0.032 | 0.086 | 0.358 | 0.417 | 0.311 | 0.256 | 0.359 | 0.416 | 0.292 | 0.283 |
| 0.230 | 0.103 | 0.025 | 0.039 | 0.099 | 0.074 | 0.121 | 0.024 | 0.064 | 0.366 | 0.403 | 0.322 | 0.249 | 0.354 | 0.406 | 0.301 | 0.288 |
| 0.234 | -0.002 | 0.041 | 0.040 | 0.079 | 0.047 | 0.157 | 0.021 | 0.048 | 0.375 | 0.385 | 0.340 | 0.242 | 0.355 | 0.390 | 0.315 | 0.287 |
| 0.238 | -0.001 | 0.177 | 0.018 | 0.044 | 0.072 | 0.188 | 0.020 | 0.033 | 0.379 | 0.368 | 0.359 | 0.235 | 0.357 | 0.373 | 0.335 | 0.291 |
| 0.242 | 0.130 | 0.210 | 0.021 | 0.016 | 0.123 | 0.196 | 0.020 | 0.022 | 0.380 | 0.357 | 0.385 | 0.226 | 0.363 | 0.358 | 0.357 | 0.288 |
| 0.246 | 0.165 | 0.203 | 0.047 | 0.008 | 0.146 | 0.180 | 0.021 | 0.015 | 0.375 | 0.353 | 0.408 | 0.217 | 0.372 | 0.345 | 0.384 | 0.283 |
| 0.251 | 0.154 | 0.180 | 0.037 | 0.019 | 0.136 | 0.152 | 0.022 | 0.009 | 0.376 | 0.349 | 0.420 | 0.208 | 0.377 | 0.341 | 0.397 | 0.275 |
| 0.255 | 0.129 | 0.156 | 0.007 | 0.022 | 0.103 | 0.118 | 0.024 | 0.007 | 0.382 | 0.342 | 0.425 | 0.201 | 0.380 | 0.342 | 0.403 | 0.265 |
| 0.259 | -0.002 | 0.115 | 0.035 | 0.008 | 0.063 | 0.082 | 0.030 | 0.005 | 0.387 | 0.337 | 0.426 | 0.195 | 0.377 | 0.343 | 0.400 | 0.253 |
| 0.263 | 0.000 | 0.034 | 0.094 | 0.006 | 0.069 | 0.058 | 0.047 | 0.005 | 0.389 | 0.340 | 0.425 | 0.188 | 0.371 | 0.357 | 0.402 | 0.242 |
| 0.267 | 0.068 | 0.054 | 0.074 | 0.029 | 0.117 | 0.051 | 0.078 | 0.005 | 0.390 | 0.353 | 0.433 | 0.182 | 0.369 | 0.374 | 0.408 | 0.229 |
| 0.271 | 0.166 | 0.112 | 0.005 | 0.022 | 0.147 | 0.062 | 0.128 | 0.005 | 0.391 | 0.378 | 0.446 | 0.178 | 0.376 | 0.396 | 0.416 | 0.216 |
| 0.275 | 0.163 | 0.150 | 0.045 | 0.006 | 0.143 | 0.078 | 0.197 | 0.006 | 0.385 | 0.417 | 0.463 | 0.176 | 0.385 | 0.413 | 0.421 | 0.206 |
| 0.279 | 0.146 | 0.021 | 0.234 | 0.016 | 0.112 | 0.106 | 0.266 | 0.007 | 0.375 | 0.449 | 0.468 | 0.173 | 0.391 | 0.437 | 0.430 | 0.201 |
| 0.283 | 0.054 | 0.020 | 0.305 | 0.032 | 0.070 | 0.144 | 0.316 | 0.008 | 0.363 | 0.475 | 0.467 | 0.171 | 0.399 | 0.454 | 0.443 | 0.195 |
| 0.287 | -0.001 | 0.100 | 0.333 | 0.017 | 0.060 | 0.180 | 0.342 | 0.009 | 0.350 | 0.484 | 0.462 | 0.171 | 0.398 | 0.459 | 0.447 | 0.197 |
| 0.291 | 0.049 | 0.210 | 0.338 | 0.006 | 0.106 | 0.205 | 0.349 | 0.010 | 0.343 | 0.482 | 0.463 | 0.174 | 0.390 | 0.453 | 0.445 | 0.197 |
| 0.295 | 0.159 | 0.215 | 0.334 | 0.024 | 0.153 | 0.210 | 0.344 | 0.011 | 0.344 | 0.475 | 0.457 | 0.176 | 0.379 | 0.444 | 0.442 | 0.196 |
| 0.299 | 0.169 | 0.196 | 0.320 | 0.033 | 0.159 | 0.196 | 0.328 | 0.012 | 0.350 | 0.463 | 0.449 | 0.178 | 0.364 | 0.438 | 0.436 | 0.195 |
| 0.303 | 0.155 | 0.168 | 0.300 | 0.009 | 0.140 | 0.166 | 0.305 | 0.012 | 0.361 | 0.447 | 0.439 | 0.184 | 0.351 | 0.430 | 0.424 | 0.196 |
| 0.307 | 0.090 | 0.144 | 0.273 | 0.009 | 0.098 | 0.123 | 0.276 | 0.013 | 0.380 | 0.430 | 0.426 | 0.191 | 0.348 | 0.422 | 0.413 | 0.203 |
| 0.311 | -0.002 | 0.091 | 0.242 | 0.034 | 0.067 | 0.075 | 0.242 | 0.014 | 0.400 | 0.403 | 0.411 | 0.198 | 0.357 | 0.403 | 0.395 | 0.213 |
| 0.315 | 0.006 | 0.056 | 0.213 | 0.034 | 0.099 | 0.038 | 0.203 | 0.015 | 0.422 | 0.371 | 0.395 | 0.206 | 0.370 | 0.378 | 0.378 | 0.224 |
| 0.319 | 0.152 | 0.075 | 0.162 | 0.006 | 0.145 | 0.034 | 0.160 | 0.016 | 0.443 | 0.336 | 0.375 | 0.218 | 0.394 | 0.358 | 0.362 | 0.237 |
| 0.323 | 0.169 | 0.116 | 0.056 | 0.006 | 0.165 | 0.048 | 0.116 | 0.018 | 0.460 | 0.309 | 0.350 | 0.235 | 0.418 | 0.342 | 0.341 | 0.253 |
| 0.327 | 0.159 | 0.064 | 0.006 | 0.047 | 0.149 | 0.071 | 0.079 | 0.021 | 0.458 | 0.293 | 0.327 | 0.251 | 0.445 | 0.333 | 0.310 | 0.269 |
| 0.331 | 0.107 | 0.016 | 0.061 | 0.036 | 0.111 | 0.105 | 0.049 | 0.027 | 0.436 | 0.292 | 0.302 | 0.266 | 0.463 | 0.326 | 0.279 | 0.285 |
| 0.335 | -0.002 | 0.066 | 0.073 | 0.001 | 0.079 | 0.163 | 0.030 | 0.040 | 0.403 | 0.303 | 0.279 | 0.283 | 0.456 | 0.321 | 0.247 | 0.303 |
| 0.339 | 0.010 | 0.224 | 0.007 | 0.025 | 0.091 | 0.238 | 0.022 | 0.052 | 0.373 | 0.308 | 0.258 | 0.304 | 0.429 | 0.324 | 0.231 | 0.320 |
| 0.343 | 0.145 | 0.281 | 0.013 | 0.089 | 0.140 | 0.283 | 0.020 | 0.082 | 0.348 | 0.315 | 0.241 | 0.323 | 0.393 | 0.335 | 0.219 | 0.337 |
| 0.347 | 0.179 | 0.308 | 0.062 | 0.046 | 0.167 | 0.304 | 0.020 | 0.114 | 0.334 | 0.325 | 0.235 | 0.340 | 0.357 | 0.338 | 0.204 | 0.350 |
| 0.352 | 0.172 | 0.313 | 0.062 | -0.017 | 0.158 | 0.305 | 0.021 | 0.153 | 0.325 | 0.336 | 0.238 | 0.362 | 0.331 | 0.345 | 0.215 | 0.368 |
| 0.356 | 0.135 | 0.300 | 0.004 | 0.064 | 0.117 | 0.290 | 0.022 | 0.202 | 0.326 | 0.350 | 0.250 | 0.386 | 0.315 | 0.357 | 0.225 | 0.390 |
| 0.360 | 0.011 | 0.270 | 0.041 | 0.251 | 0.073 | 0.249 | 0.026 | 0.252 | 0.336 | 0.368 | 0.267 | 0.414 | 0.315 | 0.370 | 0.266 | 0.412 |
| 0.364 | 0.000 | 0.131 | 0.126 | 0.313 | 0.077 | 0.192 | 0.041 | 0.299 | 0.351 | 0.398 | 0.297 | 0.435 | 0.337 | 0.388 | 0.297 | 0.429 |
| 0.368 | 0.119 | 0.039 | 0.087 | 0.350 | 0.132 | 0.131 | 0.071 | 0.346 | 0.358 | 0.428 | 0.327 | 0.450 | 0.354 | 0.415 | 0.333 | 0.441 |
| 0.372 | 0.184 | 0.054 | -0.007 | 0.382 | 0.172 | 0.081 | 0.117 | 0.385 | 0.363 | 0.452 | 0.361 | 0.456 | 0.371 | 0.439 | 0.365 | 0.445 |
| 0.376 | 0.181 | 0.129 | 0.071 | 0.410 | 0.173 | 0.057 | 0.175 | 0.414 | 0.361 | 0.453 | 0.401 | 0.457 | 0.379 | 0.430 | 0.394 | 0.446 |
| 0.380 | 0.156 | 0.099 | 0.234 | 0.431 | 0.144 | 0.061 | 0.241 | 0.442 | 0.350 | 0.424 | 0.433 | 0.456 | 0.370 | 0.403 | 0.414 | 0.443 |
| 0.384 | 0.033 | 0.039 | 0.303 | 0.446 | 0.098 | 0.100 | 0.298 | 0.452 | 0.330 | 0.384 | 0.450 | 0.451 | 0.350 | 0.359 | 0.422 | 0.436 |
| 0.388 | -0.001 | 0.106 | 0.338 | 0.455 | 0.072 | 0.156 | 0.339 | 0.460 | 0.309 | 0.335 | 0.453 | 0.442 | 0.323 | 0.307 | 0.415 | 0.429 |
| 0.392 | 0.052 | 0.204 | 0.364 | 0.462 | 0.107 | 0.213 | 0.366 | 0.376 | 0.287 | 0.282 | 0.440 | 0.423 | 0.295 | 0.252 | 0.401 | 0.317 |
| 0.396 | 0.179 | 0.250 | 0.345 | 0.246 | 0.151 | 0.205 | 0.307 | 0.188 | 0.249 | 0.210 | 0.370 | 0.248 | 0.241 | 0.148 | 0.204 | 0.157 |
| 0.400 | 0.000 | 0.000 | 0.000 | 0.000 | 0.000 | 0.000 | 0.000 | 0.000 | 0.000 | 0.000 | 0.000 | 0.000 | 0.000 | 0.000 | 0.000 | 0.000 |

**Table. 9** In the non-submerged state , the streamwise velocity *u* of three vegetation patch coverage conditions changes with the water depth *h*.

| **Hydraulic parameters** | **Streamwise velocity *u* (m/s)** | | | | | | | | |
| --- | --- | --- | --- | --- | --- | --- | --- | --- | --- |
| **Water depth *h* (m)** | **P_1_** | | | **P_2_** | | | **P_3_** | | |
|  | ***Cr*=2.09%** | ***Cr*=4.71%** | ***Cr*=8.37%** | ***Cr*=2.09%** | ***Cr*=4.71%** | ***Cr*=8.37%** | ***Cr*=2.09%** | ***Cr*=4.71%** | ***Cr*=8.37%** |
| 0.000 | 0.000 | 0.000 | 0.000 | 0.000 | 0.000 | 0.000 | 0.000 | 0.000 | 0.000 |
| 0.001 | 0.079 | 0.037 | 0.012 | 0.054 | 0.012 | -0.016 | 0.147 | 0.176 | 0.197 |
| 0.002 | 0.152 | 0.071 | 0.022 | 0.102 | 0.022 | -0.029 | 0.279 | 0.335 | 0.354 |
| 0.004 | 0.171 | 0.082 | 0.024 | 0.106 | 0.025 | -0.027 | 0.303 | 0.363 | 0.387 |
| 0.005 | 0.188 | 0.092 | 0.025 | 0.112 | 0.027 | -0.026 | 0.325 | 0.390 | 0.415 |
| 0.006 | 0.202 | 0.099 | 0.025 | 0.116 | 0.029 | -0.025 | 0.341 | 0.409 | 0.438 |
| 0.007 | 0.213 | 0.105 | 0.025 | 0.123 | 0.031 | -0.025 | 0.355 | 0.426 | 0.456 |
| 0.009 | 0.222 | 0.108 | 0.025 | 0.130 | 0.033 | -0.024 | 0.366 | 0.439 | 0.472 |
| 0.010 | 0.229 | 0.111 | 0.025 | 0.138 | 0.034 | -0.023 | 0.376 | 0.450 | 0.483 |
| 0.011 | 0.234 | 0.113 | 0.025 | 0.146 | 0.036 | -0.023 | 0.384 | 0.459 | 0.494 |
| 0.012 | 0.238 | 0.114 | 0.026 | 0.155 | 0.038 | -0.023 | 0.390 | 0.466 | 0.502 |
| 0.014 | 0.241 | 0.116 | 0.026 | 0.163 | 0.040 | -0.022 | 0.396 | 0.472 | 0.509 |
| 0.015 | 0.244 | 0.116 | 0.026 | 0.170 | 0.042 | -0.022 | 0.401 | 0.476 | 0.514 |
| 0.016 | 0.246 | 0.117 | 0.026 | 0.176 | 0.044 | -0.022 | 0.405 | 0.480 | 0.517 |
| 0.017 | 0.247 | 0.117 | 0.026 | 0.180 | 0.045 | -0.021 | 0.407 | 0.482 | 0.520 |
| 0.018 | 0.249 | 0.116 | 0.026 | 0.184 | 0.047 | -0.021 | 0.409 | 0.483 | 0.521 |
| 0.020 | 0.250 | 0.116 | 0.026 | 0.186 | 0.048 | -0.021 | 0.411 | 0.484 | 0.522 |
| 0.021 | 0.251 | 0.116 | 0.026 | 0.187 | 0.049 | -0.021 | 0.412 | 0.484 | 0.522 |
| 0.022 | 0.251 | 0.115 | 0.026 | 0.187 | 0.050 | -0.020 | 0.412 | 0.485 | 0.522 |
| 0.023 | 0.252 | 0.115 | 0.026 | 0.187 | 0.052 | -0.020 | 0.412 | 0.485 | 0.522 |
| 0.025 | 0.252 | 0.114 | 0.027 | 0.186 | 0.052 | -0.020 | 0.413 | 0.485 | 0.522 |
| 0.026 | 0.252 | 0.114 | 0.027 | 0.186 | 0.053 | -0.020 | 0.413 | 0.485 | 0.522 |
| 0.027 | 0.253 | 0.114 | 0.027 | 0.185 | 0.054 | -0.019 | 0.413 | 0.485 | 0.522 |
| 0.028 | 0.253 | 0.113 | 0.027 | 0.184 | 0.054 | -0.019 | 0.413 | 0.485 | 0.522 |
| 0.029 | 0.253 | 0.114 | 0.027 | 0.183 | 0.055 | -0.019 | 0.413 | 0.485 | 0.522 |
| 0.031 | 0.253 | 0.114 | 0.027 | 0.182 | 0.055 | -0.019 | 0.413 | 0.485 | 0.522 |
| 0.032 | 0.253 | 0.114 | 0.027 | 0.182 | 0.055 | -0.018 | 0.413 | 0.485 | 0.522 |
| 0.033 | 0.252 | 0.114 | 0.027 | 0.182 | 0.056 | -0.018 | 0.413 | 0.485 | 0.522 |
| 0.034 | 0.252 | 0.115 | 0.027 | 0.181 | 0.056 | -0.018 | 0.413 | 0.485 | 0.522 |
| 0.036 | 0.252 | 0.115 | 0.028 | 0.181 | 0.056 | -0.017 | 0.413 | 0.485 | 0.522 |
| 0.037 | 0.252 | 0.115 | 0.028 | 0.181 | 0.056 | -0.017 | 0.413 | 0.485 | 0.522 |
| 0.038 | 0.252 | 0.115 | 0.028 | 0.181 | 0.056 | -0.017 | 0.413 | 0.485 | 0.522 |
| 0.039 | 0.252 | 0.115 | 0.028 | 0.181 | 0.057 | -0.016 | 0.413 | 0.485 | 0.522 |
| 0.040 | 0.252 | 0.114 | 0.028 | 0.182 | 0.057 | -0.016 | 0.413 | 0.485 | 0.522 |
| 0.042 | 0.252 | 0.114 | 0.029 | 0.182 | 0.057 | -0.016 | 0.413 | 0.485 | 0.522 |
| 0.043 | 0.252 | 0.114 | 0.028 | 0.182 | 0.057 | -0.015 | 0.412 | 0.485 | 0.522 |
| 0.044 | 0.251 | 0.114 | 0.028 | 0.183 | 0.057 | -0.015 | 0.412 | 0.485 | 0.522 |
| 0.045 | 0.251 | 0.114 | 0.028 | 0.183 | 0.057 | -0.014 | 0.412 | 0.485 | 0.522 |
| 0.047 | 0.251 | 0.114 | 0.028 | 0.183 | 0.057 | -0.014 | 0.412 | 0.485 | 0.522 |
| 0.048 | 0.251 | 0.115 | 0.027 | 0.184 | 0.058 | -0.014 | 0.412 | 0.485 | 0.522 |
| 0.049 | 0.251 | 0.115 | 0.027 | 0.184 | 0.058 | -0.013 | 0.412 | 0.485 | 0.522 |
| 0.050 | 0.251 | 0.115 | 0.027 | 0.185 | 0.058 | -0.013 | 0.412 | 0.485 | 0.522 |
| 0.051 | 0.251 | 0.116 | 0.027 | 0.185 | 0.058 | -0.013 | 0.412 | 0.485 | 0.522 |
| 0.053 | 0.251 | 0.116 | 0.027 | 0.185 | 0.058 | -0.012 | 0.412 | 0.485 | 0.522 |
| 0.054 | 0.251 | 0.116 | 0.027 | 0.186 | 0.058 | -0.012 | 0.412 | 0.485 | 0.522 |
| 0.055 | 0.251 | 0.116 | 0.028 | 0.186 | 0.058 | -0.012 | 0.412 | 0.485 | 0.522 |
| 0.056 | 0.251 | 0.116 | 0.028 | 0.186 | 0.058 | -0.012 | 0.412 | 0.485 | 0.522 |
| 0.058 | 0.251 | 0.115 | 0.028 | 0.186 | 0.058 | -0.011 | 0.412 | 0.485 | 0.522 |
| 0.059 | 0.251 | 0.115 | 0.028 | 0.186 | 0.058 | -0.011 | 0.412 | 0.485 | 0.522 |
| 0.060 | 0.251 | 0.115 | 0.028 | 0.186 | 0.058 | -0.011 | 0.412 | 0.485 | 0.522 |

**Table. 10** In the submerged state, the streamwise velocity *u* of three vegetation patch coverage conditions changes with the water depth *h*.

| **Hydraulic parameters** | **Streamwise velocity *u* (m/s)** | | | | | | | | |
| --- | --- | --- | --- | --- | --- | --- | --- | --- | --- |
| **Water depth *h* (m)** | **P_1_** | | | **P_2_** | | | **P_3_** | | |
|  | ***Cr*=2.09%** | ***Cr*=4.71%** | ***Cr*=8.37%** | ***Cr*=2.09%** | ***Cr*=4.71%** | ***Cr*=8.37%** | ***Cr*=2.09%** | ***Cr*=4.71%** | ***Cr*=8.37%** |
| 0.000 | 0.000 | 0.000 | 0.000 | 0.000 | 0.000 | 0.000 | 0.000 | 0.000 | 0.000 |
| 0.002 | 0.098 | 0.086 | 0.056 | 0.033 | 0.005 | 0.017 | 0.081 | 0.093 | 0.072 |
| 0.004 | 0.182 | 0.108 | 0.074 | 0.065 | 0.010 | 0.028 | 0.152 | 0.174 | 0.145 |
| 0.006 | 0.198 | 0.122 | 0.069 | 0.097 | 0.015 | 0.040 | 0.223 | 0.266 | 0.216 |
| 0.008 | 0.208 | 0.126 | 0.054 | 0.129 | 0.019 | 0.049 | 0.294 | 0.358 | 0.285 |
| 0.010 | 0.215 | 0.126 | 0.058 | 0.138 | 0.021 | 0.047 | 0.333 | 0.378 | 0.354 |
| 0.012 | 0.219 | 0.121 | 0.063 | 0.147 | 0.024 | 0.045 | 0.341 | 0.384 | 0.418 |
| 0.014 | 0.224 | 0.113 | 0.062 | 0.155 | 0.026 | 0.044 | 0.348 | 0.390 | 0.423 |
| 0.016 | 0.227 | 0.109 | 0.063 | 0.162 | 0.028 | 0.042 | 0.352 | 0.396 | 0.428 |
| 0.018 | 0.229 | 0.111 | 0.063 | 0.165 | 0.029 | 0.040 | 0.355 | 0.402 | 0.433 |
| 0.020 | 0.230 | 0.111 | 0.063 | 0.167 | 0.032 | 0.037 | 0.358 | 0.409 | 0.439 |
| 0.022 | 0.231 | 0.113 | 0.063 | 0.169 | 0.035 | 0.034 | 0.361 | 0.412 | 0.443 |
| 0.025 | 0.232 | 0.113 | 0.061 | 0.171 | 0.037 | 0.031 | 0.363 | 0.414 | 0.445 |
| 0.027 | 0.233 | 0.111 | 0.061 | 0.173 | 0.038 | 0.028 | 0.364 | 0.416 | 0.447 |
| 0.029 | 0.234 | 0.108 | 0.063 | 0.173 | 0.042 | 0.026 | 0.366 | 0.417 | 0.448 |
| 0.031 | 0.233 | 0.106 | 0.061 | 0.173 | 0.045 | 0.023 | 0.367 | 0.419 | 0.450 |
| 0.033 | 0.232 | 0.104 | 0.063 | 0.172 | 0.046 | 0.021 | 0.369 | 0.421 | 0.451 |
| 0.035 | 0.234 | 0.107 | 0.063 | 0.171 | 0.047 | 0.019 | 0.370 | 0.422 | 0.451 |
| 0.037 | 0.236 | 0.107 | 0.059 | 0.170 | 0.047 | 0.016 | 0.371 | 0.423 | 0.452 |
| 0.039 | 0.236 | 0.101 | 0.059 | 0.169 | 0.048 | 0.014 | 0.371 | 0.423 | 0.452 |
| 0.041 | 0.237 | 0.094 | 0.061 | 0.168 | 0.050 | 0.011 | 0.372 | 0.423 | 0.453 |
| 0.043 | 0.238 | 0.095 | 0.063 | 0.167 | 0.051 | 0.009 | 0.372 | 0.423 | 0.453 |
| 0.045 | 0.237 | 0.102 | 0.065 | 0.166 | 0.051 | 0.007 | 0.372 | 0.424 | 0.453 |
| 0.047 | 0.238 | 0.110 | 0.062 | 0.167 | 0.051 | 0.007 | 0.373 | 0.424 | 0.453 |
| 0.049 | 0.236 | 0.113 | 0.057 | 0.169 | 0.051 | 0.008 | 0.373 | 0.424 | 0.453 |
| 0.051 | 0.235 | 0.111 | 0.062 | 0.170 | 0.052 | 0.009 | 0.373 | 0.424 | 0.453 |
| 0.053 | 0.236 | 0.109 | 0.064 | 0.172 | 0.053 | 0.011 | 0.373 | 0.424 | 0.453 |
| 0.055 | 0.240 | 0.113 | 0.061 | 0.173 | 0.055 | 0.014 | 0.373 | 0.424 | 0.453 |
| 0.057 | 0.242 | 0.116 | 0.058 | 0.174 | 0.056 | 0.016 | 0.373 | 0.424 | 0.453 |
| 0.059 | 0.245 | 0.108 | 0.059 | 0.175 | 0.058 | 0.019 | 0.373 | 0.424 | 0.453 |
| 0.061 | 0.247 | 0.093 | 0.060 | 0.178 | 0.064 | 0.023 | 0.373 | 0.424 | 0.453 |
| 0.063 | 0.248 | 0.084 | 0.056 | 0.186 | 0.072 | 0.028 | 0.373 | 0.424 | 0.453 |
| 0.065 | 0.250 | 0.093 | 0.057 | 0.194 | 0.080 | 0.033 | 0.373 | 0.424 | 0.453 |
| 0.067 | 0.252 | 0.108 | 0.056 | 0.202 | 0.087 | 0.038 | 0.373 | 0.424 | 0.453 |
| 0.069 | 0.254 | 0.122 | 0.056 | 0.210 | 0.098 | 0.043 | 0.373 | 0.424 | 0.453 |
| 0.071 | 0.258 | 0.137 | 0.058 | 0.220 | 0.109 | 0.050 | 0.373 | 0.424 | 0.453 |
| 0.074 | 0.263 | 0.150 | 0.059 | 0.230 | 0.121 | 0.061 | 0.373 | 0.424 | 0.453 |
| 0.076 | 0.269 | 0.162 | 0.060 | 0.242 | 0.133 | 0.071 | 0.373 | 0.424 | 0.453 |
| 0.078 | 0.276 | 0.176 | 0.068 | 0.255 | 0.148 | 0.082 | 0.373 | 0.424 | 0.453 |
| 0.080 | 0.286 | 0.194 | 0.084 | 0.268 | 0.163 | 0.092 | 0.373 | 0.424 | 0.453 |
| 0.082 | 0.296 | 0.211 | 0.107 | 0.281 | 0.178 | 0.105 | 0.373 | 0.424 | 0.453 |
| 0.084 | 0.308 | 0.227 | 0.142 | 0.293 | 0.193 | 0.117 | 0.373 | 0.424 | 0.453 |
| 0.086 | 0.321 | 0.241 | 0.160 | 0.304 | 0.209 | 0.129 | 0.373 | 0.424 | 0.453 |
| 0.088 | 0.333 | 0.255 | 0.173 | 0.315 | 0.224 | 0.142 | 0.373 | 0.424 | 0.453 |
| 0.090 | 0.344 | 0.269 | 0.179 | 0.325 | 0.235 | 0.153 | 0.373 | 0.424 | 0.453 |
| 0.092 | 0.353 | 0.282 | 0.183 | 0.332 | 0.245 | 0.160 | 0.373 | 0.424 | 0.453 |
| 0.094 | 0.361 | 0.292 | 0.186 | 0.338 | 0.254 | 0.164 | 0.373 | 0.424 | 0.453 |
| 0.096 | 0.367 | 0.302 | 0.188 | 0.344 | 0.261 | 0.170 | 0.373 | 0.424 | 0.453 |
| 0.098 | 0.370 | 0.306 | 0.189 | 0.350 | 0.265 | 0.177 | 0.373 | 0.424 | 0.453 |
| 0.100 | 0.373 | 0.310 | 0.190 | 0.356 | 0.270 | 0.183 | 0.373 | 0.424 | 0.453 |

**Table. 11** In the non-submerged state, the streamwise velocity *u* of four different fragmentation conditions changes with the water depth *h*.

| **Hydraulic parameters** | **Streamwise velocity *u* (m/s)** | | | | | | | | | | | |
| --- | --- | --- | --- | --- | --- | --- | --- | --- | --- | --- | --- | --- |
| **Water depth *h* (m)** | **P_1_** | | | | **P_2_** | | | | **P_3_** | | | |
|  | **Fragmentation I** | **Fragmentation II** | **Fragmentation III** | **Fragmentation IV** | **Fragmentation I** | **Fragmentation II** | **Fragmentation III** | **Fragmentation IV** | **Fragmentation I** | **Fragmentation II** | **Fragmentation III** | **Fragmentation IV** |
| 0.000 | 0.000 | 0.000 | 0.000 | 0.000 | 0.000 | 0.000 | 0.000 | 0.000 | 0.000 | 0.000 | 0.000 | 0.000 |
| 0.001 | 0.012 | 0.037 | 0.077 | 0.074 | -0.016 | 0.170 | 0.088 | 0.042 | 0.197 | 0.222 | 0.246 | 0.263 |
| 0.002 | 0.022 | 0.064 | 0.127 | 0.123 | -0.029 | 0.281 | 0.148 | 0.074 | 0.354 | 0.376 | 0.416 | 0.445 |
| 0.004 | 0.024 | 0.074 | 0.134 | 0.130 | -0.027 | 0.290 | 0.162 | 0.090 | 0.387 | 0.413 | 0.455 | 0.488 |
| 0.005 | 0.025 | 0.078 | 0.137 | 0.136 | -0.026 | 0.297 | 0.175 | 0.103 | 0.415 | 0.441 | 0.484 | 0.520 |
| 0.006 | 0.025 | 0.079 | 0.139 | 0.140 | -0.025 | 0.302 | 0.187 | 0.114 | 0.438 | 0.465 | 0.509 | 0.547 |
| 0.007 | 0.025 | 0.075 | 0.139 | 0.142 | -0.025 | 0.305 | 0.195 | 0.121 | 0.456 | 0.482 | 0.527 | 0.567 |
| 0.009 | 0.025 | 0.070 | 0.137 | 0.144 | -0.024 | 0.307 | 0.202 | 0.126 | 0.472 | 0.497 | 0.542 | 0.584 |
| 0.010 | 0.025 | 0.066 | 0.136 | 0.144 | -0.023 | 0.309 | 0.206 | 0.130 | 0.483 | 0.510 | 0.555 | 0.598 |
| 0.011 | 0.025 | 0.063 | 0.133 | 0.144 | -0.023 | 0.310 | 0.208 | 0.132 | 0.494 | 0.519 | 0.565 | 0.608 |
| 0.012 | 0.026 | 0.061 | 0.131 | 0.143 | -0.023 | 0.310 | 0.209 | 0.133 | 0.502 | 0.528 | 0.574 | 0.617 |
| 0.014 | 0.026 | 0.060 | 0.128 | 0.142 | -0.022 | 0.310 | 0.208 | 0.132 | 0.509 | 0.534 | 0.580 | 0.622 |
| 0.015 | 0.026 | 0.060 | 0.125 | 0.140 | -0.022 | 0.310 | 0.207 | 0.132 | 0.514 | 0.539 | 0.585 | 0.626 |
| 0.016 | 0.026 | 0.061 | 0.123 | 0.139 | -0.022 | 0.309 | 0.204 | 0.130 | 0.517 | 0.542 | 0.589 | 0.628 |
| 0.017 | 0.026 | 0.061 | 0.122 | 0.138 | -0.021 | 0.308 | 0.201 | 0.129 | 0.520 | 0.544 | 0.591 | 0.628 |
| 0.018 | 0.026 | 0.061 | 0.121 | 0.137 | -0.021 | 0.306 | 0.198 | 0.127 | 0.521 | 0.545 | 0.592 | 0.629 |
| 0.020 | 0.026 | 0.061 | 0.120 | 0.136 | -0.021 | 0.304 | 0.195 | 0.126 | 0.522 | 0.546 | 0.593 | 0.629 |
| 0.021 | 0.026 | 0.061 | 0.120 | 0.135 | -0.021 | 0.302 | 0.191 | 0.124 | 0.522 | 0.546 | 0.593 | 0.629 |
| 0.022 | 0.026 | 0.060 | 0.121 | 0.134 | -0.020 | 0.299 | 0.187 | 0.123 | 0.522 | 0.546 | 0.593 | 0.630 |
| 0.023 | 0.026 | 0.060 | 0.121 | 0.134 | -0.020 | 0.297 | 0.184 | 0.122 | 0.522 | 0.546 | 0.593 | 0.630 |
| 0.025 | 0.027 | 0.059 | 0.121 | 0.134 | -0.020 | 0.294 | 0.180 | 0.121 | 0.522 | 0.546 | 0.593 | 0.630 |
| 0.026 | 0.027 | 0.059 | 0.122 | 0.133 | -0.020 | 0.292 | 0.177 | 0.121 | 0.522 | 0.546 | 0.593 | 0.630 |
| 0.027 | 0.027 | 0.059 | 0.122 | 0.133 | -0.019 | 0.289 | 0.173 | 0.120 | 0.522 | 0.546 | 0.593 | 0.630 |
| 0.028 | 0.027 | 0.059 | 0.122 | 0.133 | -0.019 | 0.286 | 0.170 | 0.120 | 0.522 | 0.546 | 0.593 | 0.630 |
| 0.029 | 0.027 | 0.059 | 0.122 | 0.133 | -0.019 | 0.283 | 0.167 | 0.120 | 0.522 | 0.546 | 0.593 | 0.630 |
| 0.031 | 0.027 | 0.058 | 0.122 | 0.133 | -0.019 | 0.280 | 0.163 | 0.119 | 0.522 | 0.546 | 0.593 | 0.630 |
| 0.032 | 0.027 | 0.058 | 0.121 | 0.133 | -0.018 | 0.278 | 0.160 | 0.119 | 0.522 | 0.546 | 0.593 | 0.630 |
| 0.033 | 0.027 | 0.058 | 0.121 | 0.134 | -0.018 | 0.275 | 0.157 | 0.120 | 0.522 | 0.546 | 0.593 | 0.630 |
| 0.034 | 0.027 | 0.058 | 0.120 | 0.134 | -0.018 | 0.272 | 0.154 | 0.120 | 0.522 | 0.546 | 0.593 | 0.630 |
| 0.036 | 0.028 | 0.058 | 0.120 | 0.134 | -0.017 | 0.269 | 0.150 | 0.120 | 0.522 | 0.546 | 0.593 | 0.630 |
| 0.037 | 0.028 | 0.058 | 0.120 | 0.134 | -0.017 | 0.266 | 0.147 | 0.120 | 0.522 | 0.546 | 0.593 | 0.630 |
| 0.038 | 0.028 | 0.057 | 0.119 | 0.134 | -0.017 | 0.264 | 0.145 | 0.120 | 0.522 | 0.546 | 0.593 | 0.630 |
| 0.039 | 0.028 | 0.058 | 0.119 | 0.134 | -0.016 | 0.261 | 0.142 | 0.120 | 0.522 | 0.546 | 0.593 | 0.630 |
| 0.040 | 0.028 | 0.058 | 0.119 | 0.134 | -0.016 | 0.259 | 0.139 | 0.120 | 0.522 | 0.546 | 0.593 | 0.630 |
| 0.042 | 0.029 | 0.058 | 0.119 | 0.134 | -0.016 | 0.256 | 0.137 | 0.120 | 0.522 | 0.546 | 0.593 | 0.630 |
| 0.043 | 0.028 | 0.058 | 0.119 | 0.134 | -0.015 | 0.254 | 0.134 | 0.120 | 0.522 | 0.546 | 0.593 | 0.630 |
| 0.044 | 0.028 | 0.058 | 0.119 | 0.134 | -0.015 | 0.252 | 0.132 | 0.121 | 0.522 | 0.546 | 0.593 | 0.629 |
| 0.045 | 0.028 | 0.059 | 0.119 | 0.134 | -0.014 | 0.249 | 0.130 | 0.121 | 0.522 | 0.546 | 0.593 | 0.629 |
| 0.047 | 0.028 | 0.059 | 0.119 | 0.134 | -0.014 | 0.247 | 0.129 | 0.121 | 0.522 | 0.546 | 0.593 | 0.629 |
| 0.048 | 0.027 | 0.059 | 0.118 | 0.134 | -0.014 | 0.245 | 0.127 | 0.121 | 0.522 | 0.546 | 0.593 | 0.629 |
| 0.049 | 0.027 | 0.060 | 0.118 | 0.135 | -0.013 | 0.244 | 0.126 | 0.121 | 0.522 | 0.546 | 0.593 | 0.629 |
| 0.050 | 0.027 | 0.061 | 0.118 | 0.135 | -0.013 | 0.242 | 0.125 | 0.121 | 0.522 | 0.546 | 0.593 | 0.629 |
| 0.051 | 0.027 | 0.062 | 0.118 | 0.135 | -0.013 | 0.240 | 0.124 | 0.121 | 0.522 | 0.546 | 0.593 | 0.629 |
| 0.053 | 0.027 | 0.062 | 0.118 | 0.135 | -0.012 | 0.239 | 0.124 | 0.121 | 0.522 | 0.546 | 0.593 | 0.629 |
| 0.054 | 0.027 | 0.062 | 0.118 | 0.135 | -0.012 | 0.238 | 0.123 | 0.121 | 0.522 | 0.546 | 0.593 | 0.629 |
| 0.055 | 0.028 | 0.062 | 0.118 | 0.135 | -0.012 | 0.237 | 0.123 | 0.121 | 0.522 | 0.546 | 0.593 | 0.629 |
| 0.056 | 0.028 | 0.061 | 0.118 | 0.135 | -0.012 | 0.236 | 0.122 | 0.121 | 0.522 | 0.546 | 0.593 | 0.629 |
| 0.058 | 0.028 | 0.059 | 0.119 | 0.135 | -0.011 | 0.236 | 0.122 | 0.121 | 0.522 | 0.546 | 0.593 | 0.629 |
| 0.059 | 0.028 | 0.058 | 0.119 | 0.135 | -0.011 | 0.236 | 0.122 | 0.121 | 0.522 | 0.546 | 0.593 | 0.629 |
| 0.060 | 0.028 | 0.057 | 0.120 | 0.135 | -0.011 | 0.236 | 0.122 | 0.121 | 0.522 | 0.546 | 0.593 | 0.629 |

**Table. 12** In the submerged state, the streamwise velocity *u* of four different fragmentation conditions changes with the water depth *h*.

| **Hydraulic parameters** | **Streamwise velocity *u* (m/s)** | | | | | | | | | | | |
| --- | --- | --- | --- | --- | --- | --- | --- | --- | --- | --- | --- | --- |
| **Water depth *h* (m)** | **P_1_** | | | | **P_2_** | | | | **P_3_** | | | |
|  | **Fragmentation I** | **Fragmentation II** | **Fragmentation III** | **Fragmentation IV** | **Fragmentation I** | **Fragmentation II** | **Fragmentation III** | **Fragmentation IV** | **Fragmentation I** | **Fragmentation II** | **Fragmentation III** | **Fragmentation IV** |
| 0.000 | 0.000 | 0.000 | 0.000 | 0.000 | 0.000 | 0.000 | 0.000 | 0.000 | 0.000 | 0.000 | 0.000 | 0.000 |
| 0.002 | 0.056 | 0.069 | 0.078 | 0.043 | 0.017 | 0.033 | 0.035 | 0.043 | 0.072 | 0.129 | 0.071 | 0.146 |
| 0.004 | 0.074 | 0.086 | 0.096 | 0.069 | 0.028 | 0.059 | 0.070 | 0.069 | 0.145 | 0.256 | 0.142 | 0.292 |
| 0.006 | 0.069 | 0.084 | 0.102 | 0.085 | 0.040 | 0.084 | 0.091 | 0.085 | 0.216 | 0.362 | 0.213 | 0.421 |
| 0.008 | 0.054 | 0.081 | 0.100 | 0.103 | 0.049 | 0.107 | 0.102 | 0.103 | 0.285 | 0.416 | 0.284 | 0.428 |
| 0.010 | 0.058 | 0.086 | 0.093 | 0.117 | 0.047 | 0.117 | 0.107 | 0.117 | 0.354 | 0.425 | 0.353 | 0.446 |
| 0.012 | 0.063 | 0.096 | 0.094 | 0.125 | 0.045 | 0.122 | 0.110 | 0.125 | 0.418 | 0.436 | 0.422 | 0.457 |
| 0.014 | 0.062 | 0.105 | 0.104 | 0.125 | 0.044 | 0.127 | 0.112 | 0.125 | 0.423 | 0.443 | 0.467 | 0.468 |
| 0.016 | 0.063 | 0.098 | 0.117 | 0.124 | 0.042 | 0.131 | 0.114 | 0.124 | 0.428 | 0.450 | 0.471 | 0.475 |
| 0.018 | 0.063 | 0.083 | 0.129 | 0.121 | 0.040 | 0.130 | 0.115 | 0.121 | 0.433 | 0.456 | 0.474 | 0.479 |
| 0.020 | 0.063 | 0.075 | 0.130 | 0.121 | 0.037 | 0.128 | 0.117 | 0.121 | 0.439 | 0.461 | 0.476 | 0.483 |
| 0.022 | 0.063 | 0.077 | 0.121 | 0.121 | 0.034 | 0.127 | 0.115 | 0.121 | 0.443 | 0.466 | 0.479 | 0.487 |
| 0.025 | 0.061 | 0.093 | 0.116 | 0.120 | 0.031 | 0.125 | 0.113 | 0.120 | 0.445 | 0.468 | 0.481 | 0.493 |
| 0.027 | 0.061 | 0.101 | 0.125 | 0.114 | 0.028 | 0.125 | 0.112 | 0.114 | 0.447 | 0.469 | 0.483 | 0.494 |
| 0.029 | 0.063 | 0.095 | 0.131 | 0.111 | 0.026 | 0.124 | 0.109 | 0.111 | 0.448 | 0.470 | 0.485 | 0.495 |
| 0.031 | 0.061 | 0.085 | 0.131 | 0.108 | 0.023 | 0.123 | 0.107 | 0.108 | 0.450 | 0.472 | 0.487 | 0.497 |
| 0.033 | 0.063 | 0.084 | 0.133 | 0.106 | 0.021 | 0.123 | 0.107 | 0.106 | 0.451 | 0.473 | 0.489 | 0.498 |
| 0.035 | 0.063 | 0.092 | 0.126 | 0.104 | 0.019 | 0.127 | 0.105 | 0.104 | 0.451 | 0.474 | 0.491 | 0.499 |
| 0.037 | 0.059 | 0.090 | 0.125 | 0.107 | 0.016 | 0.132 | 0.103 | 0.107 | 0.452 | 0.474 | 0.493 | 0.499 |
| 0.039 | 0.059 | 0.091 | 0.123 | 0.111 | 0.014 | 0.137 | 0.103 | 0.111 | 0.452 | 0.474 | 0.495 | 0.499 |
| 0.041 | 0.061 | 0.096 | 0.128 | 0.120 | 0.011 | 0.142 | 0.104 | 0.120 | 0.453 | 0.475 | 0.496 | 0.499 |
| 0.043 | 0.063 | 0.102 | 0.126 | 0.128 | 0.009 | 0.149 | 0.107 | 0.128 | 0.453 | 0.475 | 0.496 | 0.499 |
| 0.045 | 0.065 | 0.109 | 0.125 | 0.129 | 0.007 | 0.160 | 0.110 | 0.129 | 0.453 | 0.475 | 0.496 | 0.499 |
| 0.047 | 0.062 | 0.115 | 0.123 | 0.131 | 0.007 | 0.168 | 0.109 | 0.131 | 0.453 | 0.475 | 0.497 | 0.499 |
| 0.049 | 0.057 | 0.118 | 0.128 | 0.130 | 0.008 | 0.175 | 0.113 | 0.130 | 0.453 | 0.475 | 0.497 | 0.500 |
| 0.051 | 0.062 | 0.116 | 0.141 | 0.130 | 0.009 | 0.183 | 0.127 | 0.130 | 0.453 | 0.475 | 0.497 | 0.500 |
| 0.053 | 0.064 | 0.112 | 0.143 | 0.131 | 0.011 | 0.190 | 0.141 | 0.131 | 0.453 | 0.475 | 0.497 | 0.500 |
| 0.055 | 0.061 | 0.107 | 0.143 | 0.132 | 0.014 | 0.197 | 0.157 | 0.132 | 0.453 | 0.475 | 0.498 | 0.500 |
| 0.057 | 0.058 | 0.106 | 0.148 | 0.129 | 0.016 | 0.204 | 0.181 | 0.129 | 0.453 | 0.475 | 0.498 | 0.500 |
| 0.059 | 0.059 | 0.108 | 0.157 | 0.128 | 0.019 | 0.215 | 0.205 | 0.128 | 0.453 | 0.475 | 0.498 | 0.500 |
| 0.061 | 0.060 | 0.098 | 0.165 | 0.126 | 0.023 | 0.228 | 0.225 | 0.126 | 0.453 | 0.475 | 0.498 | 0.500 |
| 0.063 | 0.056 | 0.091 | 0.167 | 0.126 | 0.028 | 0.241 | 0.246 | 0.126 | 0.453 | 0.475 | 0.498 | 0.500 |
| 0.065 | 0.057 | 0.098 | 0.178 | 0.128 | 0.033 | 0.253 | 0.267 | 0.128 | 0.453 | 0.475 | 0.498 | 0.500 |
| 0.067 | 0.056 | 0.118 | 0.181 | 0.135 | 0.038 | 0.266 | 0.286 | 0.135 | 0.453 | 0.475 | 0.497 | 0.500 |
| 0.069 | 0.056 | 0.130 | 0.192 | 0.145 | 0.043 | 0.278 | 0.302 | 0.145 | 0.453 | 0.475 | 0.498 | 0.500 |
| 0.071 | 0.058 | 0.137 | 0.194 | 0.162 | 0.050 | 0.292 | 0.319 | 0.162 | 0.453 | 0.475 | 0.498 | 0.500 |
| 0.074 | 0.059 | 0.144 | 0.191 | 0.183 | 0.061 | 0.307 | 0.335 | 0.183 | 0.453 | 0.475 | 0.498 | 0.500 |
| 0.076 | 0.060 | 0.159 | 0.204 | 0.213 | 0.071 | 0.322 | 0.352 | 0.213 | 0.453 | 0.475 | 0.498 | 0.500 |
| 0.078 | 0.068 | 0.180 | 0.239 | 0.243 | 0.082 | 0.335 | 0.369 | 0.243 | 0.453 | 0.475 | 0.498 | 0.500 |
| 0.080 | 0.084 | 0.200 | 0.259 | 0.267 | 0.092 | 0.344 | 0.384 | 0.267 | 0.453 | 0.475 | 0.498 | 0.500 |
| 0.082 | 0.107 | 0.223 | 0.278 | 0.290 | 0.105 | 0.354 | 0.394 | 0.290 | 0.453 | 0.475 | 0.498 | 0.500 |
| 0.084 | 0.142 | 0.246 | 0.300 | 0.312 | 0.117 | 0.362 | 0.404 | 0.312 | 0.453 | 0.475 | 0.499 | 0.500 |
| 0.086 | 0.160 | 0.270 | 0.321 | 0.331 | 0.129 | 0.371 | 0.413 | 0.331 | 0.453 | 0.475 | 0.499 | 0.500 |
| 0.088 | 0.173 | 0.294 | 0.339 | 0.350 | 0.142 | 0.380 | 0.418 | 0.350 | 0.453 | 0.475 | 0.499 | 0.500 |
| 0.090 | 0.179 | 0.312 | 0.357 | 0.367 | 0.153 | 0.389 | 0.421 | 0.367 | 0.453 | 0.475 | 0.499 | 0.500 |
| 0.092 | 0.183 | 0.325 | 0.374 | 0.379 | 0.160 | 0.395 | 0.424 | 0.379 | 0.453 | 0.475 | 0.499 | 0.500 |
| 0.094 | 0.186 | 0.335 | 0.388 | 0.393 | 0.164 | 0.398 | 0.426 | 0.393 | 0.453 | 0.475 | 0.499 | 0.500 |
| 0.096 | 0.188 | 0.343 | 0.399 | 0.401 | 0.170 | 0.402 | 0.429 | 0.401 | 0.453 | 0.475 | 0.499 | 0.500 |
| 0.098 | 0.189 | 0.348 | 0.406 | 0.408 | 0.177 | 0.406 | 0.431 | 0.408 | 0.453 | 0.475 | 0.499 | 0.500 |
| 0.100 | 0.190 | 0.352 | 0.412 | 0.413 | 0.183 | 0.409 | 0.434 | 0.413 | 0.453 | 0.475 | 0.499 | 0.500 |

**Table. 13** In the non-submerged state , the Reynolds stress *u'w'* of three vegetation patch coverage conditions changes with the water depth *h*.

| **Hydraulic parameters** | **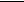Reynolds stress *u'w'* (N/m^2^)** | | | | | | | | |
| --- | --- | --- | --- | --- | --- | --- | --- | --- | --- |
| **Water depth *h* (m)** | **P1** | | | **P2** | | | **P3** | | |
|  | ***Cr*=2.09%** | ***Cr*=4.71%** | ***Cr*=8.37%** | ***Cr*=2.09%** | ***Cr*=4.71%** | ***Cr*=8.37%** | ***Cr*=2.09%** | ***Cr*=4.71%** | ***Cr*=8.37%** |
| 0.000 | 0.15137 | 0.07927 | 0.02482 | 0.09477 | 0.03761 | 0.00321 | 0.30401 | 0.41261 | 0.45982 |
| 0.001 | 0.16110 | 0.08693 | 0.01794 | 0.10135 | 0.04549 | 0.00536 | 0.28760 | 0.39037 | 0.44800 |
| 0.002 | 0.17070 | 0.09413 | 0.01033 | 0.11116 | 0.05436 | 0.00743 | 0.27014 | 0.36616 | 0.43176 |
| 0.004 | 0.19020 | 0.10485 | 0.00222 | 0.14189 | 0.07076 | 0.01616 | 0.24452 | 0.32694 | 0.40228 |
| 0.005 | 0.20610 | 0.11042 | -0.00337 | 0.17907 | 0.08709 | 0.02920 | 0.21980 | 0.28803 | 0.36551 |
| 0.006 | 0.21732 | 0.10271 | -0.00539 | 0.22994 | 0.10317 | 0.02904 | 0.19803 | 0.25016 | 0.32146 |
| 0.007 | 0.22586 | 0.09146 | -0.00544 | 0.28216 | 0.11826 | 0.02828 | 0.17520 | 0.21158 | 0.27087 |
| 0.009 | 0.22300 | 0.07064 | -0.00487 | 0.32554 | 0.13147 | 0.02726 | 0.15036 | 0.17170 | 0.21811 |
| 0.010 | 0.21685 | 0.05218 | -0.00373 | 0.36024 | 0.14199 | 0.02586 | 0.12624 | 0.13460 | 0.16918 |
| 0.011 | 0.20165 | 0.03463 | -0.00280 | 0.37402 | 0.14938 | 0.02441 | 0.10298 | 0.10075 | 0.12304 |
| 0.012 | 0.18512 | 0.02149 | -0.00262 | 0.36744 | 0.15169 | 0.02288 | 0.08167 | 0.07267 | 0.08529 |
| 0.014 | 0.16494 | 0.01013 | -0.00235 | 0.34196 | 0.15026 | 0.02128 | 0.06181 | 0.04881 | 0.05533 |
| 0.015 | 0.14519 | 0.00277 | -0.00206 | 0.29644 | 0.14244 | 0.01977 | 0.04558 | 0.03252 | 0.03315 |
| 0.016 | 0.12479 | -0.00389 | -0.00169 | 0.24353 | 0.13177 | 0.01824 | 0.03096 | 0.01959 | 0.02038 |
| 0.017 | 0.10544 | -0.00711 | -0.00130 | 0.18551 | 0.11573 | 0.01680 | 0.02131 | 0.01266 | 0.01075 |
| 0.018 | 0.08630 | -0.00996 | -0.00077 | 0.12806 | 0.09841 | 0.01553 | 0.01282 | 0.00717 | 0.00707 |
| 0.020 | 0.06955 | -0.00996 | -0.00033 | 0.08223 | 0.07914 | 0.01423 | 0.00855 | 0.00471 | 0.00393 |
| 0.021 | 0.05314 | -0.00969 | -0.00001 | 0.03837 | 0.05980 | 0.01324 | 0.00471 | 0.00256 | 0.00241 |
| 0.022 | 0.04028 | -0.00722 | 0.00004 | 0.01231 | 0.04291 | 0.01215 | 0.00308 | 0.00163 | 0.00132 |
| 0.023 | 0.02785 | -0.00443 | -0.00001 | -0.01179 | 0.02693 | 0.01143 | 0.00159 | 0.00078 | 0.00064 |
| 0.025 | 0.01858 | -0.00005 | -0.00023 | -0.02225 | 0.01592 | 0.01070 | 0.00092 | 0.00038 | 0.00025 |
| 0.026 | 0.00995 | 0.00422 | -0.00048 | -0.03057 | 0.00681 | 0.01016 | 0.00036 | 0.00006 | -0.00004 |
| 0.027 | 0.00382 | 0.00892 | -0.00070 | -0.03069 | 0.00286 | 0.00986 | 0.00010 | -0.00009 | -0.00016 |
| 0.028 | -0.00153 | 0.01245 | -0.00088 | -0.02944 | 0.00118 | 0.00948 | -0.00010 | -0.00020 | -0.00025 |
| 0.029 | -0.00505 | 0.01510 | -0.00093 | -0.02421 | 0.00313 | 0.00947 | -0.00019 | -0.00024 | -0.00028 |
| 0.031 | -0.00774 | 0.01616 | -0.00095 | -0.01859 | 0.00781 | 0.00928 | -0.00025 | -0.00027 | -0.00029 |
| 0.032 | -0.00927 | 0.01605 | -0.00090 | -0.01135 | 0.00680 | 0.00946 | -0.00027 | -0.00027 | -0.00029 |
| 0.033 | -0.01006 | 0.01478 | -0.00079 | -0.00472 | 0.00617 | 0.00955 | -0.00027 | -0.00026 | -0.00028 |
| 0.034 | -0.01021 | 0.01272 | -0.00065 | 0.00251 | 0.00578 | 0.00979 | -0.00026 | -0.00025 | -0.00027 |
| 0.036 | -0.00978 | 0.01046 | -0.00037 | 0.00853 | 0.00563 | 0.01012 | -0.00025 | -0.00023 | -0.00025 |
| 0.037 | -0.00906 | 0.00799 | -0.00014 | 0.01464 | 0.00557 | 0.01036 | -0.00024 | -0.00022 | -0.00024 |
| 0.038 | -0.00793 | 0.00650 | 0.00001 | 0.01933 | 0.00557 | 0.01075 | -0.00022 | -0.00020 | -0.00022 |
| 0.039 | -0.00672 | 0.00516 | 0.00006 | 0.02394 | 0.00558 | 0.01098 | -0.00021 | -0.00019 | -0.00020 |
| 0.040 | -0.00529 | 0.00563 | 0.00004 | 0.02707 | 0.00549 | 0.01132 | -0.00019 | -0.00018 | -0.00019 |
| 0.042 | -0.00385 | 0.00627 | -0.00014 | 0.03003 | 0.00539 | 0.01152 | -0.00018 | -0.00016 | -0.00017 |
| 0.043 | -0.00244 | 0.00840 | -0.00037 | 0.03171 | 0.00511 | 0.01174 | -0.00016 | -0.00015 | -0.00016 |
| 0.044 | -0.00106 | 0.01041 | -0.00088 | 0.03307 | 0.00480 | 0.01179 | -0.00015 | -0.00014 | -0.00015 |
| 0.045 | 0.00015 | 0.01286 | -0.00136 | 0.03342 | 0.00434 | 0.01178 | -0.00014 | -0.00012 | -0.00013 |
| 0.047 | 0.00127 | 0.01465 | -0.00193 | 0.03338 | 0.00386 | 0.01158 | -0.00012 | -0.00011 | -0.00012 |
| 0.048 | 0.00215 | 0.01583 | -0.00217 | 0.03251 | 0.00332 | 0.01130 | -0.00011 | -0.00010 | -0.00011 |
| 0.049 | 0.00289 | 0.01594 | -0.00228 | 0.03127 | 0.00278 | 0.01080 | -0.00010 | -0.00009 | -0.00010 |
| 0.050 | 0.00337 | 0.01480 | -0.00164 | 0.02929 | 0.00225 | 0.01016 | -0.00009 | -0.00008 | -0.00009 |
| 0.051 | 0.00365 | 0.01267 | -0.00079 | 0.02696 | 0.00177 | 0.00937 | -0.00008 | -0.00007 | -0.00007 |
| 0.053 | 0.00371 | 0.00952 | 0.00038 | 0.02399 | 0.00133 | 0.00830 | -0.00007 | -0.00006 | -0.00006 |
| 0.054 | 0.00352 | 0.00637 | 0.00128 | 0.02074 | 0.00098 | 0.00726 | -0.00005 | -0.00005 | -0.00005 |
| 0.055 | 0.00315 | 0.00309 | 0.00149 | 0.01698 | 0.00066 | 0.00587 | -0.00004 | -0.00004 | -0.00004 |
| 0.056 | 0.00255 | 0.00119 | 0.00127 | 0.01302 | 0.00044 | 0.00463 | -0.00003 | -0.00003 | -0.00003 |
| 0.058 | 0.00185 | -0.00029 | 0.00061 | 0.00879 | 0.00025 | 0.00306 | -0.00002 | -0.00002 | -0.00002 |
| 0.059 | 0.00095 | -0.00026 | 0.00027 | 0.00444 | 0.00012 | 0.00160 | -0.00001 | -0.00001 | -0.00001 |
| 0.060 | 0.00000 | 0.00000 | 0.00000 | 0.00000 | 0.00000 | 0.00000 | 0.00000 | 0.00000 | 0.00000 |

**Table. 14** In the submerged state , the Reynolds stress *u'w'* of three vegetation patch coverage conditions changes with the water depth *h*.

| **Hydraulic parameters** | **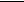Reynolds stress *u'w'* (N/m^2^)** | | | | | | | | |
| --- | --- | --- | --- | --- | --- | --- | --- | --- | --- |
| **Water depth *h* (m)** | **P1** | | | **P2** | | | **P3** | | |
|  | ***Cr*=2.09%** | ***Cr*=4.71%** | ***Cr*=8.37%** | ***Cr*=2.09%** | ***Cr*=4.71%** | ***Cr*=8.37%** | ***Cr*=2.09%** | ***Cr*=4.71%** | ***Cr*=8.37%** |
| 0.000 | 0.15366 | 0.06244 | 0.12077 | 0.12022 | 0.02010 | 0.01622 | 0.22296 | 0.28214 | 0.33860 |
| 0.002 | 0.12478 | 0.04532 | 0.10855 | 0.11195 | 0.02087 | 0.01740 | 0.19894 | 0.25686 | 0.30388 |
| 0.004 | 0.09658 | 0.00791 | 0.08822 | 0.10368 | 0.02167 | 0.01907 | 0.17592 | 0.22907 | 0.26974 |
| 0.006 | 0.07819 | -0.02754 | 0.04885 | 0.09569 | 0.02374 | 0.02323 | 0.15456 | 0.19799 | 0.23559 |
| 0.008 | 0.06753 | -0.00933 | 0.00361 | 0.08752 | 0.02458 | 0.02766 | 0.13261 | 0.16691 | 0.19884 |
| 0.010 | 0.06176 | 0.01712 | -0.02462 | 0.07923 | 0.02417 | 0.02735 | 0.11269 | 0.13583 | 0.16661 |
| 0.012 | 0.05380 | 0.00792 | -0.06454 | 0.06779 | 0.02529 | 0.02721 | 0.09286 | 0.10792 | 0.13438 |
| 0.014 | 0.04498 | 0.00277 | -0.06528 | 0.05635 | 0.02759 | 0.02846 | 0.07455 | 0.08189 | 0.10214 |
| 0.016 | 0.03460 | 0.00620 | -0.03429 | 0.04440 | 0.02958 | 0.02971 | 0.05813 | 0.06569 | 0.07340 |
| 0.018 | 0.02681 | 0.00424 | 0.00940 | 0.03227 | 0.02652 | 0.03096 | 0.04631 | 0.04872 | 0.05950 |
| 0.020 | 0.02062 | 0.00460 | 0.01926 | 0.01998 | 0.02511 | 0.03219 | 0.03896 | 0.03806 | 0.04992 |
| 0.022 | 0.01478 | 0.00177 | 0.01463 | 0.00996 | 0.02648 | 0.03008 | 0.03162 | 0.02843 | 0.04034 |
| 0.025 | 0.01103 | 0.00548 | 0.00566 | 0.01033 | 0.02617 | 0.02809 | 0.02428 | 0.02278 | 0.03076 |
| 0.027 | 0.00937 | 0.00609 | -0.00970 | 0.00868 | 0.02516 | 0.02536 | 0.01811 | 0.01974 | 0.02117 |
| 0.029 | 0.00865 | 0.00468 | -0.01047 | 0.00779 | 0.02639 | 0.01989 | 0.01377 | 0.01671 | 0.01449 |
| 0.031 | 0.00773 | 0.01605 | 0.00138 | 0.00691 | 0.02738 | 0.01441 | 0.01188 | 0.01367 | 0.01200 |
| 0.033 | 0.01365 | 0.00851 | 0.02421 | 0.00602 | 0.02838 | 0.01260 | 0.01002 | 0.01063 | 0.01007 |
| 0.035 | 0.01629 | 0.00633 | 0.02663 | 0.00820 | 0.02596 | 0.01117 | 0.00819 | 0.00721 | 0.00814 |
| 0.037 | 0.01711 | 0.00789 | 0.00865 | 0.01158 | 0.02124 | 0.00973 | 0.00637 | 0.00452 | 0.00621 |
| 0.039 | 0.01743 | 0.01967 | -0.01418 | 0.01367 | 0.01652 | 0.00747 | 0.00455 | 0.00369 | 0.00389 |
| 0.041 | 0.01578 | 0.01813 | -0.01297 | 0.01575 | 0.01180 | 0.00373 | 0.00280 | 0.00307 | 0.00177 |
| 0.043 | 0.01060 | 0.01216 | 0.02220 | 0.01784 | 0.00708 | 0.00061 | 0.00231 | 0.00244 | 0.00128 |
| 0.045 | 0.00258 | 0.01289 | 0.04843 | 0.01992 | 0.00236 | -0.00198 | 0.00195 | 0.00182 | 0.00106 |
| 0.047 | 0.00047 | -0.01195 | 0.03323 | 0.02004 | -0.00236 | -0.00016 | 0.00160 | 0.00119 | 0.00083 |
| 0.049 | 0.00521 | 0.00373 | 0.00792 | 0.01860 | -0.00322 | 0.00464 | 0.00133 | 0.00073 | 0.00061 |
| 0.051 | 0.00799 | 0.01929 | -0.00171 | 0.01476 | 0.01042 | 0.01279 | 0.00106 | 0.00060 | 0.00038 |
| 0.053 | 0.01539 | 0.00888 | 0.02145 | 0.01091 | 0.02477 | 0.02664 | 0.00079 | 0.00046 | 0.00016 |
| 0.055 | 0.02748 | -0.00220 | 0.02270 | 0.00706 | 0.03911 | 0.04095 | 0.00052 | 0.00033 | 0.00006 |
| 0.057 | 0.02590 | 0.00247 | -0.01012 | 0.00321 | 0.05345 | 0.05527 | 0.00026 | 0.00020 | 0.00004 |
| 0.059 | 0.02132 | 0.00634 | -0.06427 | 0.00888 | 0.06779 | 0.06958 | 0.00014 | 0.00010 | 0.00002 |
| 0.061 | 0.02050 | 0.00030 | -0.08352 | 0.02294 | 0.08213 | 0.12011 | 0.00011 | 0.00002 | -0.00001 |
| 0.063 | 0.02476 | 0.00149 | -0.03052 | 0.04104 | 0.09648 | 0.18295 | 0.00007 | -0.00004 | -0.00003 |
| 0.065 | 0.03161 | 0.00099 | 0.06581 | 0.05914 | 0.11135 | 0.24579 | 0.00003 | -0.00004 | -0.00006 |
| 0.067 | 0.04614 | 0.00286 | 0.12245 | 0.08601 | 0.14283 | 0.31057 | 0.00001 | -0.00004 | -0.00006 |
| 0.069 | 0.06510 | 0.00898 | 0.14043 | 0.11591 | 0.17668 | 0.38304 | 0.00000 | -0.00004 | -0.00005 |
| 0.071 | 0.08941 | 0.00476 | 0.15272 | 0.14582 | 0.22402 | 0.45765 | -0.00001 | -0.00004 | -0.00005 |
| 0.074 | 0.11816 | -0.00258 | 0.20230 | 0.16949 | 0.27042 | 0.53226 | -0.00002 | -0.00004 | -0.00005 |
| 0.076 | 0.14136 | 0.00853 | 0.32252 | 0.16964 | 0.31649 | 0.59166 | -0.00002 | -0.00005 | -0.00005 |
| 0.078 | 0.16285 | 0.04102 | 0.46671 | 0.16493 | 0.36256 | 0.60815 | -0.00002 | -0.00006 | -0.00005 |
| 0.080 | 0.17202 | 0.11585 | 0.60172 | 0.15938 | 0.40831 | 0.62464 | -0.00003 | -0.00006 | -0.00005 |
| 0.082 | 0.17805 | 0.26228 | 0.68659 | 0.14745 | 0.40801 | 0.64112 | -0.00003 | -0.00007 | -0.00005 |
| 0.084 | 0.17188 | 0.42275 | 0.75221 | 0.11814 | 0.39842 | 0.64906 | -0.00003 | -0.00007 | -0.00005 |
| 0.086 | 0.16080 | 0.43205 | 0.78552 | 0.09418 | 0.38551 | 0.65073 | -0.00003 | -0.00007 | -0.00006 |
| 0.088 | 0.13581 | 0.41261 | 0.80591 | 0.07763 | 0.37709 | 0.62627 | -0.00003 | -0.00008 | -0.00006 |
| 0.090 | 0.10889 | 0.37000 | 0.76124 | 0.06113 | 0.35153 | 0.54358 | -0.00002 | -0.00008 | -0.00007 |
| 0.092 | 0.08177 | 0.31719 | 0.70548 | 0.04821 | 0.29163 | 0.45236 | -0.00002 | -0.00008 | -0.00008 |
| 0.094 | 0.05625 | 0.25171 | 0.57551 | 0.03530 | 0.22717 | 0.35484 | -0.00002 | -0.00007 | -0.00005 |
| 0.096 | 0.03264 | 0.18033 | 0.43661 | 0.02263 | 0.15144 | 0.25088 | -0.00001 | -0.00005 | -0.00003 |
| 0.098 | 0.01630 | 0.09440 | 0.21855 | 0.01169 | 0.07572 | 0.12679 | -0.00001 | -0.00003 | -0.00002 |
| 0.100 | 0.00000 | 0.00000 | 0.00000 | 0.00000 | 0.00000 | 0.00000 | 0.00000 | 0.00000 | 0.00000 |

**Table. 15** In the non-submerged state, the Reynolds stress *u'w'* of four different fragmentation conditions changes with the water depth *h*.

| **Hydraulic parameters** | **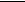Reynolds stress *u'w'* (N/m^2^)** | | | | | | | | | | | |
| --- | --- | --- | --- | --- | --- | --- | --- | --- | --- | --- | --- | --- |
| **Water depth *h* (m)** | **P_1_** | | | | **P_2_** | | | | **P_3_** | | | |
|  | **Fragmentation I** | **Fragmentation II** | **Fragmentation III** | **Fragmentation IV** | **Fragmentation I** | **Fragmentation II** | **Fragmentation III** | **Fragmentation IV** | **Fragmentation I** | **Fragmentation II** | **Fragmentation III** | **Fragmentation IV** |
| 0.000 | 0.02482 | 0.08451 | 0.19466 | 0.10227 | 0.00321 | -0.02357 | 0.04403 | 0.10227 | 0.45982 | 0.50802 | 0.59863 | 0.67324 |
| 0.001 | 0.01794 | 0.06286 | 0.17641 | 0.17837 | 0.00336 | -0.00987 | 0.06696 | 0.17837 | 0.44800 | 0.48597 | 0.55681 | 0.63624 |
| 0.002 | 0.01033 | 0.03741 | 0.15819 | 0.25053 | 0.00360 | 0.00399 | 0.09638 | 0.25053 | 0.43176 | 0.45680 | 0.50596 | 0.58875 |
| 0.004 | 0.00222 | 0.02848 | 0.12885 | 0.31568 | 0.00431 | 0.01806 | 0.12959 | 0.31568 | 0.40228 | 0.41536 | 0.43951 | 0.52311 |
| 0.005 | -0.00337 | 0.02108 | 0.09836 | 0.30579 | 0.00507 | 0.02349 | 0.14672 | 0.30579 | 0.36551 | 0.36999 | 0.38280 | 0.45678 |
| 0.006 | -0.00539 | -0.00095 | 0.06774 | 0.26795 | 0.00607 | 0.02598 | 0.15459 | 0.26795 | 0.32146 | 0.32244 | 0.32944 | 0.38932 |
| 0.007 | -0.00544 | -0.02436 | 0.04328 | 0.22111 | 0.00690 | 0.03031 | 0.14376 | 0.22111 | 0.27087 | 0.26806 | 0.27443 | 0.31384 |
| 0.009 | -0.00487 | -0.02809 | 0.03558 | 0.17870 | 0.00774 | 0.03516 | 0.12380 | 0.17870 | 0.21811 | 0.21541 | 0.22193 | 0.24052 |
| 0.010 | -0.00373 | -0.01220 | 0.03371 | 0.14171 | 0.00846 | 0.04057 | 0.09863 | 0.14171 | 0.16918 | 0.16474 | 0.17231 | 0.16965 |
| 0.011 | -0.00280 | 0.00385 | 0.03988 | 0.10736 | 0.00913 | 0.04584 | 0.06913 | 0.10736 | 0.12304 | 0.12127 | 0.12919 | 0.11272 |
| 0.012 | -0.00262 | 0.01692 | 0.04326 | 0.07386 | 0.00973 | 0.05077 | 0.04052 | 0.07386 | 0.08529 | 0.08189 | 0.08967 | 0.06375 |
| 0.014 | -0.00235 | 0.01845 | 0.04650 | 0.04227 | 0.01026 | 0.05441 | 0.01653 | 0.04227 | 0.05533 | 0.05308 | 0.05934 | 0.03551 |
| 0.015 | -0.00206 | 0.01546 | 0.05298 | 0.01446 | 0.01075 | 0.05658 | -0.00067 | 0.01446 | 0.03315 | 0.03219 | 0.03669 | 0.01784 |
| 0.016 | -0.00169 | 0.00839 | 0.06340 | -0.01076 | 0.01116 | 0.05752 | -0.01444 | -0.01076 | 0.02038 | 0.01736 | 0.01991 | 0.00824 |
| 0.017 | -0.00130 | 0.00074 | 0.07363 | -0.02657 | 0.01156 | 0.05543 | -0.01874 | -0.02657 | 0.01075 | 0.01075 | 0.01221 | 0.00485 |
| 0.018 | -0.00077 | -0.00650 | 0.07965 | -0.03878 | 0.01188 | 0.05224 | -0.01949 | -0.03878 | 0.00707 | 0.00582 | 0.00645 | 0.00247 |
| 0.020 | -0.00033 | -0.01293 | 0.08438 | -0.04439 | 0.01220 | 0.04694 | -0.01710 | -0.04439 | 0.00393 | 0.00343 | 0.00373 | 0.00140 |
| 0.021 | -0.00001 | -0.01821 | 0.08109 | -0.04565 | 0.01248 | 0.04060 | -0.01157 | -0.04565 | 0.00241 | 0.00195 | 0.00206 | 0.00074 |
| 0.022 | 0.00004 | -0.02290 | 0.07783 | -0.04466 | 0.01272 | 0.03371 | -0.00563 | -0.04466 | 0.00132 | 0.00093 | 0.00094 | 0.00029 |
| 0.023 | -0.00001 | -0.02577 | 0.06943 | -0.03992 | 0.01296 | 0.02690 | 0.00141 | -0.03992 | 0.00064 | 0.00046 | 0.00046 | 0.00008 |
| 0.025 | -0.00023 | -0.02797 | 0.05620 | -0.03481 | 0.01313 | 0.02051 | 0.00718 | -0.03481 | 0.00025 | 0.00010 | 0.00010 | -0.00008 |
| 0.026 | -0.00048 | -0.02922 | 0.04138 | -0.02889 | 0.01331 | 0.01486 | 0.01196 | -0.02889 | -0.00004 | -0.00010 | -0.00010 | -0.00017 |
| 0.027 | -0.00070 | -0.02961 | 0.02727 | -0.02379 | 0.01342 | 0.01025 | 0.01383 | -0.02379 | -0.00016 | -0.00020 | -0.00019 | -0.00021 |
| 0.028 | -0.00088 | -0.02967 | 0.01633 | -0.01916 | 0.01350 | 0.00614 | 0.01467 | -0.01916 | -0.00025 | -0.00027 | -0.00024 | -0.00024 |
| 0.029 | -0.00093 | -0.02924 | 0.01273 | -0.01617 | 0.01356 | 0.00310 | 0.01368 | -0.01617 | -0.00028 | -0.00028 | -0.00025 | -0.00025 |
| 0.031 | -0.00095 | -0.02869 | 0.01239 | -0.01399 | 0.01354 | 0.00044 | 0.01180 | -0.01399 | -0.00029 | -0.00029 | -0.00025 | -0.00024 |
| 0.032 | -0.00090 | -0.02790 | 0.01304 | -0.01260 | 0.01354 | -0.00179 | 0.00888 | -0.01260 | -0.00029 | -0.00028 | -0.00024 | -0.00023 |
| 0.033 | -0.00079 | -0.02670 | 0.01521 | -0.01171 | 0.01342 | -0.00356 | 0.00550 | -0.01171 | -0.00028 | -0.00027 | -0.00023 | -0.00022 |
| 0.034 | -0.00065 | -0.02534 | 0.01778 | -0.01082 | 0.01330 | -0.00515 | 0.00233 | -0.01082 | -0.00027 | -0.00025 | -0.00021 | -0.00020 |
| 0.036 | -0.00037 | -0.02362 | 0.01942 | -0.00960 | 0.01310 | -0.00642 | -0.00019 | -0.00960 | -0.00025 | -0.00023 | -0.00020 | -0.00018 |
| 0.037 | -0.00014 | -0.02191 | 0.02116 | -0.00801 | 0.01283 | -0.00750 | -0.00123 | -0.00801 | -0.00024 | -0.00022 | -0.00018 | -0.00016 |
| 0.038 | 0.00001 | -0.01999 | 0.02107 | -0.00612 | 0.01255 | -0.00845 | -0.00160 | -0.00612 | -0.00022 | -0.00020 | -0.00017 | -0.00015 |
| 0.039 | 0.00006 | -0.01822 | 0.01770 | -0.00404 | 0.01216 | -0.00916 | -0.00007 | -0.00404 | -0.00020 | -0.00019 | -0.00016 | -0.00013 |
| 0.040 | 0.00004 | -0.01679 | 0.01561 | -0.00214 | 0.01177 | -0.00980 | 0.00171 | -0.00214 | -0.00019 | -0.00017 | -0.00015 | -0.00012 |
| 0.042 | -0.00014 | -0.01501 | 0.01398 | -0.00058 | 0.01125 | -0.01030 | 0.00382 | -0.00058 | -0.00017 | -0.00016 | -0.00014 | -0.00011 |
| 0.043 | -0.00037 | -0.01339 | 0.01625 | 0.00031 | 0.01073 | -0.01064 | 0.00536 | 0.00031 | -0.00016 | -0.00015 | -0.00013 | -0.00011 |
| 0.044 | -0.00088 | -0.01144 | 0.01883 | 0.00089 | 0.01010 | -0.01088 | 0.00664 | 0.00089 | -0.00015 | -0.00013 | -0.00012 | -0.00010 |
| 0.045 | -0.00136 | -0.00974 | 0.01847 | 0.00081 | 0.00946 | -0.01088 | 0.00684 | 0.00081 | -0.00013 | -0.00012 | -0.00011 | -0.00009 |
| 0.047 | -0.00193 | -0.00871 | 0.01600 | 0.00067 | 0.00874 | -0.01073 | 0.00641 | 0.00067 | -0.00012 | -0.00011 | -0.00010 | -0.00008 |
| 0.048 | -0.00217 | -0.00780 | 0.01126 | 0.00038 | 0.00800 | -0.01040 | 0.00584 | 0.00038 | -0.00011 | -0.00010 | -0.00009 | -0.00007 |
| 0.049 | -0.00228 | -0.00725 | 0.00526 | 0.00027 | 0.00720 | -0.00974 | 0.00499 | 0.00027 | -0.00010 | -0.00009 | -0.00008 | -0.00006 |
| 0.050 | -0.00164 | -0.00668 | 0.00003 | 0.00027 | 0.00641 | -0.00895 | 0.00422 | 0.00027 | -0.00009 | -0.00008 | -0.00007 | -0.00006 |
| 0.051 | -0.00079 | -0.00595 | -0.00058 | 0.00059 | 0.00558 | -0.00790 | 0.00378 | 0.00059 | -0.00007 | -0.00007 | -0.00006 | -0.00005 |
| 0.053 | 0.00038 | -0.00556 | 0.00258 | 0.00101 | 0.00475 | -0.00678 | 0.00356 | 0.00101 | -0.00006 | -0.00006 | -0.00006 | -0.00004 |
| 0.054 | 0.00128 | -0.00545 | 0.00966 | 0.00153 | 0.00397 | -0.00559 | 0.00349 | 0.00153 | -0.00005 | -0.00005 | -0.00005 | -0.00004 |
| 0.055 | 0.00149 | -0.00576 | 0.02269 | 0.00184 | 0.00311 | -0.00446 | 0.00351 | 0.00184 | -0.00004 | -0.00004 | -0.00004 | -0.00003 |
| 0.056 | 0.00127 | -0.00591 | 0.03319 | 0.00195 | 0.00236 | -0.00334 | 0.00343 | 0.00195 | -0.00003 | -0.00003 | -0.00003 | -0.00002 |
| 0.058 | 0.00061 | -0.00515 | 0.03421 | 0.00161 | 0.00151 | -0.00227 | 0.00275 | 0.00161 | -0.00002 | -0.00002 | -0.00002 | -0.00001 |
| 0.059 | 0.00027 | -0.00314 | 0.02073 | 0.00091 | 0.00078 | -0.00115 | 0.00157 | 0.00091 | -0.00001 | -0.00001 | -0.00001 | -0.00001 |
| 0.060 | 0.00000 | 0.00000 | 0.00000 | 0.00000 | 0.00000 | 0.00000 | 0.00000 | 0.00000 | 0.00000 | 0.00000 | 0.00000 | 0.00000 |

**Table. 16** In the submerged state, the Reynolds stress *u'w'* of four different fragmentation conditions changes with the water depth *h*.

| **Hydraulic parameters** | **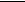Reynolds stress *u'w'* (N/m^2^)** | | | | | | | | | | | |
| --- | --- | --- | --- | --- | --- | --- | --- | --- | --- | --- | --- | --- |
| **Water depth *h* (m)** | **P_1_** | | | | **P_2_** | | | | **P_3_** | | | |
|  | **Fragmentation I** | **Fragmentation II** | **Fragmentation III** | **Fragmentation IV** | **Fragmentation I** | **Fragmentation II** | **Fragmentation III** | **Fragmentation IV** | **Fragmentation I** | **Fragmentation II** | **Fragmentation III** | **Fragmentation IV** |
| 0.000 | 0.12077 | 0.10801 | 0.11150 | 0.11255 | 0.01622 | 0.10628 | 0.05453 | 0.06498 | 0.34026 | 0.36570 | 0.37293 | 0.41447 |
| 0.002 | 0.10855 | 0.11946 | 0.07871 | 0.09326 | 0.01740 | 0.09012 | 0.06065 | 0.05842 | 0.30356 | 0.32890 | 0.34653 | 0.37744 |
| 0.004 | 0.08822 | 0.04913 | 0.05067 | 0.05573 | 0.01907 | 0.07879 | 0.06690 | 0.06665 | 0.26819 | 0.29234 | 0.32013 | 0.34057 |
| 0.006 | 0.04885 | -0.01755 | 0.04199 | 0.02045 | 0.02323 | 0.06873 | 0.05822 | 0.08661 | 0.23320 | 0.25825 | 0.29373 | 0.29568 |
| 0.008 | 0.00361 | -0.00210 | 0.02429 | 0.00108 | 0.02766 | 0.05851 | 0.04160 | 0.08092 | 0.19873 | 0.22444 | 0.26733 | 0.25203 |
| 0.010 | -0.02462 | 0.04023 | 0.02166 | -0.00607 | 0.02735 | 0.05309 | 0.02531 | 0.05753 | 0.16427 | 0.19050 | 0.24186 | 0.20725 |
| 0.012 | -0.06454 | 0.08188 | 0.02758 | -0.00225 | 0.02721 | 0.04533 | 0.01162 | 0.02864 | 0.13109 | 0.15395 | 0.21875 | 0.16246 |
| 0.014 | -0.06528 | 0.02685 | 0.03770 | -0.00214 | 0.02846 | 0.03630 | 0.01441 | 0.00452 | 0.11445 | 0.12498 | 0.19675 | 0.11768 |
| 0.016 | -0.03429 | -0.07451 | 0.02646 | -0.00337 | 0.02971 | 0.02946 | 0.01637 | -0.00335 | 0.09781 | 0.09886 | 0.17732 | 0.09568 |
| 0.018 | 0.00940 | -0.07729 | 0.00956 | -0.00477 | 0.03096 | 0.02292 | 0.01710 | -0.01352 | 0.07821 | 0.07560 | 0.15971 | 0.07771 |
| 0.020 | 0.01926 | 0.03466 | -0.02890 | -0.00567 | 0.03219 | 0.01568 | 0.01058 | -0.01887 | 0.05784 | 0.05467 | 0.14437 | 0.06264 |
| 0.022 | 0.01463 | 0.12086 | -0.05559 | -0.00178 | 0.03008 | 0.00844 | 0.00370 | -0.02550 | 0.04283 | 0.03616 | 0.13079 | 0.04766 |
| 0.025 | 0.00566 | 0.10449 | -0.01791 | 0.00534 | 0.02809 | 0.00044 | -0.00614 | -0.04084 | 0.03451 | 0.02955 | 0.11721 | 0.03268 |
| 0.027 | -0.00970 | 0.02754 | -0.01050 | 0.00794 | 0.02536 | 0.02460 | -0.01420 | -0.04556 | 0.02660 | 0.02529 | 0.10363 | 0.02501 |
| 0.029 | -0.01047 | -0.01353 | -0.02508 | 0.00205 | 0.01989 | 0.05449 | -0.01924 | -0.05127 | 0.02070 | 0.02007 | 0.09005 | 0.02048 |
| 0.031 | 0.00138 | 0.00462 | -0.04436 | -0.00406 | 0.01441 | 0.08438 | -0.02081 | -0.05581 | 0.01516 | 0.01422 | 0.07648 | 0.01595 |
| 0.033 | 0.02421 | 0.05956 | -0.04561 | -0.01404 | 0.01260 | 0.11150 | -0.02045 | -0.02814 | 0.00992 | 0.00888 | 0.06290 | 0.01141 |
| 0.035 | 0.02663 | 0.05299 | -0.03056 | -0.01866 | 0.01117 | 0.16736 | -0.01741 | 0.00229 | 0.00823 | 0.00725 | 0.04932 | 0.00659 |
| 0.037 | 0.00865 | 0.02496 | -0.00158 | -0.02016 | 0.00973 | 0.24114 | -0.00974 | 0.02911 | 0.00650 | 0.00596 | 0.03574 | 0.00475 |
| 0.039 | -0.01418 | 0.05100 | 0.00810 | -0.01806 | 0.00747 | 0.31491 | -0.00121 | 0.06088 | 0.00468 | 0.00491 | 0.02397 | 0.00412 |
| 0.041 | -0.01297 | 0.07035 | 0.01740 | -0.01755 | 0.00373 | 0.38869 | 0.00590 | 0.06595 | 0.00285 | 0.00387 | 0.01739 | 0.00348 |
| 0.043 | 0.02220 | 0.06113 | -0.00835 | -0.01686 | 0.00061 | 0.43657 | 0.01790 | 0.05922 | 0.00225 | 0.00283 | 0.01309 | 0.00285 |
| 0.045 | 0.04843 | 0.07118 | -0.02529 | -0.01842 | -0.00198 | 0.48872 | 0.03728 | 0.01889 | 0.00190 | 0.00204 | 0.01111 | 0.00222 |
| 0.047 | 0.03323 | 0.06893 | 0.01030 | -0.01786 | -0.00016 | 0.52853 | 0.07428 | -0.00548 | 0.00155 | 0.00160 | 0.01006 | 0.00159 |
| 0.049 | 0.00792 | 0.05399 | 0.07934 | -0.01668 | 0.00464 | 0.56314 | 0.15835 | -0.00971 | 0.00121 | 0.00122 | 0.00877 | 0.00105 |
| 0.051 | -0.00171 | 0.04075 | 0.10472 | -0.00947 | 0.01279 | 0.63963 | 0.34993 | -0.01244 | 0.00087 | 0.00105 | 0.00747 | 0.00076 |
| 0.053 | 0.02145 | 0.03381 | 0.10026 | -0.00014 | 0.02664 | 0.77008 | 0.54131 | 0.00617 | 0.00050 | 0.00089 | 0.00618 | 0.00055 |
| 0.055 | 0.02270 | 0.06647 | 0.13433 | 0.01742 | 0.04095 | 0.90054 | 0.74708 | 0.02441 | 0.00034 | 0.00072 | 0.00489 | 0.00035 |
| 0.057 | -0.01012 | 0.08238 | 0.20931 | 0.03770 | 0.05527 | 1.03099 | 1.05714 | 0.04038 | 0.00024 | 0.00056 | 0.00359 | 0.00014 |
| 0.059 | -0.06427 | 0.03164 | 0.24503 | 0.05296 | 0.06958 | 1.18294 | 1.35448 | 0.06445 | 0.00015 | 0.00039 | 0.00230 | 0.00008 |
| 0.061 | -0.08352 | -0.01408 | 0.22391 | 0.04731 | 0.12011 | 1.33470 | 1.62307 | 0.08417 | 0.00007 | 0.00024 | 0.00200 | 0.00004 |
| 0.063 | -0.03052 | 0.04591 | 0.24133 | 0.03757 | 0.18295 | 1.48646 | 1.87040 | 0.12096 | 0.00001 | 0.00016 | 0.00223 | 0.00002 |
| 0.065 | 0.06581 | 0.13918 | 0.26975 | 0.02183 | 0.24579 | 1.63822 | 2.11677 | 0.16940 | -0.00001 | 0.00011 | 0.00245 | 0.00000 |
| 0.067 | 0.12245 | 0.15473 | 0.24480 | 0.02227 | 0.31057 | 1.71683 | 2.26523 | 0.24727 | -0.00002 | 0.00009 | 0.00266 | -0.00002 |
| 0.069 | 0.14043 | 0.11266 | 0.23962 | 0.04343 | 0.38304 | 1.68717 | 2.32059 | 0.34380 | -0.00002 | 0.00008 | 0.00251 | -0.00004 |
| 0.071 | 0.15272 | 0.06434 | 0.15626 | 0.10829 | 0.45765 | 1.64713 | 2.19164 | 0.47412 | -0.00003 | 0.00006 | 0.00237 | -0.00005 |
| 0.074 | 0.20230 | 0.16739 | 0.19943 | 0.17709 | 0.53226 | 1.60691 | 1.96053 | 0.57093 | -0.00003 | 0.00005 | 0.00222 | -0.00005 |
| 0.076 | 0.32252 | 0.32744 | 0.37853 | 0.24788 | 0.59166 | 1.56703 | 1.67407 | 0.69107 | -0.00003 | 0.00003 | 0.00208 | -0.00004 |
| 0.078 | 0.46671 | 0.46246 | 0.41825 | 0.41899 | 0.60815 | 1.49828 | 1.38760 | 0.78129 | -0.00004 | 0.00002 | 0.00193 | -0.00004 |
| 0.080 | 0.60172 | 0.56728 | 0.46220 | 0.62312 | 0.62464 | 1.37600 | 1.08959 | 0.85698 | -0.00004 | 0.00002 | 0.00158 | -0.00003 |
| 0.082 | 0.68659 | 0.72089 | 0.64025 | 0.81294 | 0.64112 | 1.25372 | 0.87595 | 0.88049 | -0.00005 | 0.00002 | 0.00121 | -0.00004 |
| 0.084 | 0.75221 | 0.80339 | 0.80725 | 0.94347 | 0.64906 | 1.11326 | 0.64582 | 0.84806 | -0.00005 | 0.00001 | 0.00105 | -0.00004 |
| 0.086 | 0.78552 | 0.84011 | 0.92973 | 1.01007 | 0.65073 | 0.96288 | 0.41570 | 0.78354 | -0.00005 | 0.00001 | 0.00085 | -0.00004 |
| 0.088 | 0.80591 | 0.80236 | 0.95875 | 1.01834 | 0.62627 | 0.80362 | 0.30774 | 0.70896 | -0.00006 | 0.00000 | 0.00064 | -0.00004 |
| 0.090 | 0.76124 | 0.69209 | 0.89590 | 1.00118 | 0.54358 | 0.65059 | 0.23925 | 0.62934 | -0.00006 | 0.00000 | 0.00043 | -0.00004 |
| 0.092 | 0.70548 | 0.53077 | 0.78452 | 0.91308 | 0.45236 | 0.50029 | 0.17355 | 0.52531 | -0.00006 | -0.00001 | 0.00028 | -0.00004 |
| 0.094 | 0.57551 | 0.36722 | 0.62689 | 0.77275 | 0.35484 | 0.37012 | 0.13180 | 0.42315 | -0.00005 | -0.00001 | 0.00020 | -0.00003 |
| 0.096 | 0.43661 | 0.21248 | 0.44808 | 0.59299 | 0.25088 | 0.24323 | 0.08935 | 0.28918 | -0.00003 | -0.00001 | 0.00012 | -0.00003 |
| 0.098 | 0.21855 | 0.10488 | 0.24744 | 0.31390 | 0.12679 | 0.12161 | 0.05078 | 0.13473 | -0.00002 | 0.00000 | 0.00004 | -0.00001 |
| 0.100 | 0.00000 | 0.00000 | 0.00000 | 0.00000 | 0.00000 | 0.00000 | 0.00000 | 0.00000 | 0.00000 | 0.00000 | 0.00000 | 0.00000 |

**Table. 17** In the non-submerged state (*h*=0.05 m), the Turbulent kinetic energy *TKE* of three vegetation patch coverage conditions changes along the *x* direction.

| **Hydraulic parameters** | **Turbulent kinetic energy *TKE* (m^2^/s^2^)** | | | | | | | | |
| --- | --- | --- | --- | --- | --- | --- | --- | --- | --- |
| ***x* (m)** | **L_1-1_** | | | **L_3-3_** | | | **L_4-4_** | | |
|  | ***Cr*=2.09%** | ***Cr*=4.71%** | ***Cr*=8.37%** | ***Cr*=2.09%** | ***Cr*=4.71%** | ***Cr*=8.37%** | ***Cr*=2.09%** | ***Cr*=4.71%** | ***Cr*=8.37%** |
| 0.000 | 0.00034 | 0.00034 | 0.00034 | 0.00034 | 0.00034 | 0.00034 | 0.00034 | 0.00034 | 0.00034 |
| 0.009 | 0.00031 | 0.00031 | 0.00031 | 0.00031 | 0.00031 | 0.00031 | 0.00031 | 0.00031 | 0.00031 |
| 0.017 | 0.00029 | 0.00029 | 0.00029 | 0.00029 | 0.00029 | 0.00029 | 0.00029 | 0.00029 | 0.00029 |
| 0.026 | 0.00027 | 0.00027 | 0.00027 | 0.00027 | 0.00027 | 0.00027 | 0.00027 | 0.00027 | 0.00027 |
| 0.035 | 0.00025 | 0.00025 | 0.00025 | 0.00025 | 0.00025 | 0.00025 | 0.00025 | 0.00025 | 0.00025 |
| 0.043 | 0.00023 | 0.00023 | 0.00024 | 0.00023 | 0.00023 | 0.00024 | 0.00023 | 0.00023 | 0.00024 |
| 0.052 | 0.00022 | 0.00022 | 0.00022 | 0.00022 | 0.00022 | 0.00022 | 0.00022 | 0.00022 | 0.00022 |
| 0.061 | 0.00021 | 0.00021 | 0.00021 | 0.00021 | 0.00021 | 0.00021 | 0.00021 | 0.00021 | 0.00021 |
| 0.069 | 0.00020 | 0.00020 | 0.00020 | 0.00020 | 0.00020 | 0.00020 | 0.00020 | 0.00020 | 0.00020 |
| 0.078 | 0.00019 | 0.00019 | 0.00019 | 0.00019 | 0.00019 | 0.00019 | 0.00019 | 0.00019 | 0.00019 |
| 0.086 | 0.00018 | 0.00018 | 0.00018 | 0.00018 | 0.00018 | 0.00018 | 0.00018 | 0.00018 | 0.00018 |
| 0.095 | 0.00017 | 0.00017 | 0.00017 | 0.00017 | 0.00017 | 0.00017 | 0.00017 | 0.00017 | 0.00017 |
| 0.104 | 0.00016 | 0.00016 | 0.00016 | 0.00016 | 0.00016 | 0.00016 | 0.00016 | 0.00016 | 0.00016 |
| 0.112 | 0.00016 | 0.00016 | 0.00016 | 0.00016 | 0.00016 | 0.00016 | 0.00016 | 0.00016 | 0.00016 |
| 0.121 | 0.00015 | 0.00015 | 0.00015 | 0.00015 | 0.00015 | 0.00015 | 0.00015 | 0.00015 | 0.00015 |
| 0.130 | 0.00014 | 0.00014 | 0.00014 | 0.00014 | 0.00014 | 0.00014 | 0.00014 | 0.00014 | 0.00014 |
| 0.138 | 0.00014 | 0.00014 | 0.00014 | 0.00014 | 0.00014 | 0.00014 | 0.00014 | 0.00014 | 0.00014 |
| 0.147 | 0.00013 | 0.00013 | 0.00013 | 0.00013 | 0.00013 | 0.00013 | 0.00013 | 0.00013 | 0.00013 |
| 0.156 | 0.00013 | 0.00013 | 0.00013 | 0.00013 | 0.00013 | 0.00013 | 0.00013 | 0.00013 | 0.00013 |
| 0.164 | 0.00012 | 0.00012 | 0.00012 | 0.00012 | 0.00012 | 0.00012 | 0.00012 | 0.00012 | 0.00013 |
| 0.173 | 0.00012 | 0.00012 | 0.00012 | 0.00012 | 0.00012 | 0.00012 | 0.00012 | 0.00012 | 0.00012 |
| 0.182 | 0.00012 | 0.00012 | 0.00012 | 0.00012 | 0.00012 | 0.00012 | 0.00012 | 0.00012 | 0.00012 |
| 0.190 | 0.00011 | 0.00011 | 0.00011 | 0.00011 | 0.00011 | 0.00011 | 0.00011 | 0.00011 | 0.00011 |
| 0.199 | 0.00011 | 0.00011 | 0.00011 | 0.00011 | 0.00011 | 0.00011 | 0.00011 | 0.00011 | 0.00011 |
| 0.207 | 0.00011 | 0.00011 | 0.00011 | 0.00011 | 0.00011 | 0.00011 | 0.00011 | 0.00011 | 0.00011 |
| 0.216 | 0.00010 | 0.00010 | 0.00010 | 0.00010 | 0.00010 | 0.00010 | 0.00010 | 0.00010 | 0.00010 |
| 0.225 | 0.00010 | 0.00010 | 0.00010 | 0.00010 | 0.00010 | 0.00010 | 0.00010 | 0.00010 | 0.00010 |
| 0.233 | 0.00010 | 0.00010 | 0.00010 | 0.00010 | 0.00010 | 0.00010 | 0.00010 | 0.00010 | 0.00010 |
| 0.242 | 0.00009 | 0.00009 | 0.00009 | 0.00009 | 0.00009 | 0.00010 | 0.00010 | 0.00010 | 0.00010 |
| 0.251 | 0.00009 | 0.00009 | 0.00009 | 0.00009 | 0.00009 | 0.00009 | 0.00009 | 0.00009 | 0.00009 |
| 0.259 | 0.00009 | 0.00009 | 0.00009 | 0.00009 | 0.00009 | 0.00009 | 0.00009 | 0.00009 | 0.00009 |
| 0.268 | 0.00009 | 0.00009 | 0.00009 | 0.00009 | 0.00009 | 0.00009 | 0.00009 | 0.00009 | 0.00009 |
| 0.277 | 0.00008 | 0.00008 | 0.00008 | 0.00009 | 0.00009 | 0.00009 | 0.00009 | 0.00009 | 0.00009 |
| 0.285 | 0.00008 | 0.00008 | 0.00008 | 0.00008 | 0.00008 | 0.00008 | 0.00008 | 0.00009 | 0.00009 |
| 0.294 | 0.00008 | 0.00008 | 0.00008 | 0.00008 | 0.00008 | 0.00008 | 0.00008 | 0.00008 | 0.00008 |
| 0.303 | 0.00008 | 0.00008 | 0.00008 | 0.00008 | 0.00008 | 0.00008 | 0.00008 | 0.00008 | 0.00008 |
| 0.311 | 0.00008 | 0.00008 | 0.00008 | 0.00008 | 0.00008 | 0.00008 | 0.00008 | 0.00008 | 0.00008 |
| 0.320 | 0.00008 | 0.00008 | 0.00008 | 0.00008 | 0.00008 | 0.00008 | 0.00008 | 0.00008 | 0.00008 |
| 0.328 | 0.00007 | 0.00007 | 0.00007 | 0.00008 | 0.00008 | 0.00008 | 0.00008 | 0.00008 | 0.00008 |
| 0.337 | 0.00007 | 0.00007 | 0.00007 | 0.00007 | 0.00007 | 0.00008 | 0.00007 | 0.00008 | 0.00008 |
| 0.346 | 0.00007 | 0.00007 | 0.00007 | 0.00007 | 0.00007 | 0.00008 | 0.00007 | 0.00007 | 0.00008 |
| 0.354 | 0.00007 | 0.00007 | 0.00007 | 0.00007 | 0.00007 | 0.00008 | 0.00007 | 0.00007 | 0.00008 |
| 0.363 | 0.00020 | 0.00028 | 0.00024 | 0.00007 | 0.00007 | 0.00007 | 0.00007 | 0.00007 | 0.00007 |
| 0.372 | 0.00125 | 0.00092 | 0.00109 | 0.00007 | 0.00007 | 0.00007 | 0.00007 | 0.00007 | 0.00007 |
| 0.380 | 0.00512 | 0.00268 | 0.00255 | 0.00007 | 0.00007 | 0.00007 | 0.00007 | 0.00007 | 0.00007 |
| 0.389 | 0.00555 | 0.00238 | 0.00269 | 0.00007 | 0.00007 | 0.00007 | 0.00007 | 0.00007 | 0.00007 |
| 0.398 | 0.00498 | 0.00333 | 0.00230 | 0.00007 | 0.00007 | 0.00008 | 0.00007 | 0.00007 | 0.00007 |
| 0.406 | 0.00343 | 0.00381 | 0.00404 | 0.00006 | 0.00007 | 0.00008 | 0.00006 | 0.00007 | 0.00007 |
| 0.415 | 0.00362 | 0.00304 | 0.00310 | 0.00006 | 0.00007 | 0.00010 | 0.00006 | 0.00007 | 0.00007 |
| 0.424 | 0.00395 | 0.00402 | 0.00333 | 0.00006 | 0.00007 | 0.00014 | 0.00006 | 0.00006 | 0.00007 |
| 0.432 | 0.00550 | 0.00282 | 0.00267 | 0.00006 | 0.00006 | 0.00021 | 0.00006 | 0.00006 | 0.00007 |
| 0.441 | 0.00337 | 0.00388 | 0.00223 | 0.00006 | 0.00006 | 0.00031 | 0.00006 | 0.00006 | 0.00007 |
| 0.449 | 0.00446 | 0.00355 | 0.00254 | 0.00006 | 0.00006 | 0.00040 | 0.00006 | 0.00006 | 0.00007 |
| 0.458 | 0.00492 | 0.00322 | 0.00182 | 0.00006 | 0.00006 | 0.00053 | 0.00006 | 0.00006 | 0.00007 |
| 0.467 | 0.00619 | 0.00568 | 0.00222 | 0.00006 | 0.00006 | 0.00067 | 0.00006 | 0.00006 | 0.00006 |
| 0.475 | 0.00376 | 0.00313 | 0.00137 | 0.00006 | 0.00007 | 0.00078 | 0.00006 | 0.00006 | 0.00006 |
| 0.484 | 0.00481 | 0.00395 | 0.00182 | 0.00006 | 0.00007 | 0.00089 | 0.00006 | 0.00006 | 0.00006 |
| 0.493 | 0.00475 | 0.00324 | 0.00149 | 0.00006 | 0.00008 | 0.00101 | 0.00006 | 0.00006 | 0.00006 |
| 0.501 | 0.00445 | 0.00287 | 0.00106 | 0.00006 | 0.00010 | 0.00111 | 0.00005 | 0.00006 | 0.00006 |
| 0.510 | 0.00412 | 0.00258 | 0.00080 | 0.00005 | 0.00012 | 0.00121 | 0.00005 | 0.00006 | 0.00006 |
| 0.519 | 0.00381 | 0.00228 | 0.00065 | 0.00005 | 0.00016 | 0.00130 | 0.00005 | 0.00006 | 0.00006 |
| 0.527 | 0.00352 | 0.00202 | 0.00055 | 0.00005 | 0.00020 | 0.00138 | 0.00005 | 0.00006 | 0.00006 |
| 0.536 | 0.00322 | 0.00181 | 0.00046 | 0.00005 | 0.00027 | 0.00148 | 0.00005 | 0.00006 | 0.00006 |
| 0.545 | 0.00294 | 0.00163 | 0.00041 | 0.00005 | 0.00035 | 0.00159 | 0.00005 | 0.00005 | 0.00006 |
| 0.553 | 0.00272 | 0.00148 | 0.00038 | 0.00005 | 0.00046 | 0.00170 | 0.00005 | 0.00005 | 0.00006 |
| 0.562 | 0.00254 | 0.00135 | 0.00035 | 0.00005 | 0.00058 | 0.00181 | 0.00005 | 0.00005 | 0.00006 |
| 0.570 | 0.00238 | 0.00123 | 0.00032 | 0.00005 | 0.00073 | 0.00192 | 0.00005 | 0.00005 | 0.00006 |
| 0.579 | 0.00224 | 0.00113 | 0.00030 | 0.00005 | 0.00090 | 0.00204 | 0.00005 | 0.00005 | 0.00006 |
| 0.588 | 0.00211 | 0.00104 | 0.00028 | 0.00005 | 0.00109 | 0.00217 | 0.00005 | 0.00005 | 0.00006 |
| 0.596 | 0.00200 | 0.00097 | 0.00026 | 0.00005 | 0.00130 | 0.00233 | 0.00005 | 0.00005 | 0.00006 |
| 0.605 | 0.00189 | 0.00090 | 0.00025 | 0.00005 | 0.00154 | 0.00251 | 0.00005 | 0.00005 | 0.00006 |
| 0.614 | 0.00180 | 0.00084 | 0.00024 | 0.00005 | 0.00180 | 0.00272 | 0.00005 | 0.00005 | 0.00006 |
| 0.622 | 0.00172 | 0.00078 | 0.00024 | 0.00005 | 0.00207 | 0.00295 | 0.00005 | 0.00005 | 0.00005 |
| 0.631 | 0.00165 | 0.00074 | 0.00024 | 0.00005 | 0.00235 | 0.00319 | 0.00005 | 0.00005 | 0.00005 |
| 0.640 | 0.00159 | 0.00070 | 0.00026 | 0.00005 | 0.00263 | 0.00340 | 0.00005 | 0.00005 | 0.00005 |
| 0.648 | 0.00153 | 0.00066 | 0.00028 | 0.00005 | 0.00288 | 0.00355 | 0.00004 | 0.00005 | 0.00005 |
| 0.657 | 0.00150 | 0.00065 | 0.00031 | 0.00005 | 0.00310 | 0.00366 | 0.00004 | 0.00005 | 0.00005 |
| 0.666 | 0.00246 | 0.00116 | 0.00038 | 0.00005 | 0.00326 | 0.00372 | 0.00004 | 0.00005 | 0.00005 |
| 0.674 | 0.00206 | 0.00161 | 0.00076 | 0.00007 | 0.00337 | 0.00376 | 0.00004 | 0.00005 | 0.00005 |
| 0.683 | 0.00353 | 0.00304 | 0.00063 | 0.00011 | 0.00343 | 0.00377 | 0.00004 | 0.00005 | 0.00005 |
| 0.691 | 0.00472 | 0.00206 | 0.00082 | 0.00017 | 0.00345 | 0.00374 | 0.00004 | 0.00005 | 0.00005 |
| 0.700 | 0.00440 | 0.00282 | 0.00055 | 0.00027 | 0.00344 | 0.00366 | 0.00004 | 0.00005 | 0.00005 |
| 0.709 | 0.00371 | 0.00200 | 0.00070 | 0.00039 | 0.00338 | 0.00361 | 0.00004 | 0.00005 | 0.00005 |
| 0.717 | 0.00471 | 0.00234 | 0.00054 | 0.00053 | 0.00330 | 0.00359 | 0.00004 | 0.00005 | 0.00005 |
| 0.726 | 0.00498 | 0.00303 | 0.00043 | 0.00067 | 0.00320 | 0.00349 | 0.00004 | 0.00005 | 0.00005 |
| 0.735 | 0.00396 | 0.00194 | 0.00054 | 0.00080 | 0.00309 | 0.00342 | 0.00004 | 0.00004 | 0.00005 |
| 0.743 | 0.00330 | 0.00291 | 0.00040 | 0.00092 | 0.00297 | 0.00337 | 0.00004 | 0.00004 | 0.00005 |
| 0.752 | 0.00426 | 0.00203 | 0.00055 | 0.00101 | 0.00286 | 0.00330 | 0.00004 | 0.00004 | 0.00005 |
| 0.761 | 0.00458 | 0.00214 | 0.00031 | 0.00108 | 0.00276 | 0.00327 | 0.00004 | 0.00004 | 0.00005 |
| 0.769 | 0.00443 | 0.00245 | 0.00029 | 0.00113 | 0.00267 | 0.00326 | 0.00004 | 0.00004 | 0.00005 |
| 0.778 | 0.00350 | 0.00168 | 0.00025 | 0.00116 | 0.00259 | 0.00324 | 0.00004 | 0.00004 | 0.00005 |
| 0.787 | 0.00451 | 0.00193 | 0.00026 | 0.00118 | 0.00253 | 0.00327 | 0.00004 | 0.00004 | 0.00005 |
| 0.795 | 0.00449 | 0.00192 | 0.00018 | 0.00119 | 0.00248 | 0.00328 | 0.00004 | 0.00004 | 0.00005 |
| 0.804 | 0.00412 | 0.00171 | 0.00011 | 0.00119 | 0.00244 | 0.00330 | 0.00004 | 0.00004 | 0.00005 |
| 0.812 | 0.00371 | 0.00148 | 0.00007 | 0.00119 | 0.00242 | 0.00335 | 0.00004 | 0.00004 | 0.00005 |
| 0.821 | 0.00335 | 0.00126 | 0.00005 | 0.00119 | 0.00240 | 0.00340 | 0.00004 | 0.00004 | 0.00005 |
| 0.830 | 0.00304 | 0.00107 | 0.00004 | 0.00119 | 0.00239 | 0.00346 | 0.00004 | 0.00004 | 0.00005 |
| 0.838 | 0.00278 | 0.00092 | 0.00003 | 0.00119 | 0.00239 | 0.00353 | 0.00004 | 0.00004 | 0.00004 |
| 0.847 | 0.00254 | 0.00080 | 0.00003 | 0.00119 | 0.00239 | 0.00360 | 0.00004 | 0.00004 | 0.00004 |
| 0.856 | 0.00234 | 0.00070 | 0.00003 | 0.00119 | 0.00240 | 0.00368 | 0.00004 | 0.00004 | 0.00004 |
| 0.864 | 0.00217 | 0.00063 | 0.00003 | 0.00119 | 0.00242 | 0.00377 | 0.00004 | 0.00004 | 0.00004 |
| 0.873 | 0.00201 | 0.00057 | 0.00005 | 0.00119 | 0.00244 | 0.00387 | 0.00004 | 0.00004 | 0.00004 |
| 0.882 | 0.00187 | 0.00052 | 0.00007 | 0.00120 | 0.00247 | 0.00398 | 0.00004 | 0.00004 | 0.00004 |
| 0.890 | 0.00175 | 0.00048 | 0.00010 | 0.00120 | 0.00251 | 0.00410 | 0.00003 | 0.00004 | 0.00004 |
| 0.899 | 0.00164 | 0.00044 | 0.00016 | 0.00121 | 0.00255 | 0.00423 | 0.00003 | 0.00004 | 0.00004 |
| 0.908 | 0.00154 | 0.00041 | 0.00023 | 0.00123 | 0.00260 | 0.00438 | 0.00003 | 0.00004 | 0.00004 |
| 0.916 | 0.00145 | 0.00038 | 0.00033 | 0.00125 | 0.00266 | 0.00454 | 0.00003 | 0.00004 | 0.00004 |
| 0.925 | 0.00137 | 0.00036 | 0.00050 | 0.00127 | 0.00273 | 0.00473 | 0.00003 | 0.00004 | 0.00004 |
| 0.933 | 0.00129 | 0.00033 | 0.00079 | 0.00130 | 0.00281 | 0.00493 | 0.00003 | 0.00004 | 0.00004 |
| 0.942 | 0.00123 | 0.00031 | 0.00123 | 0.00133 | 0.00290 | 0.00515 | 0.00003 | 0.00004 | 0.00004 |
| 0.951 | 0.00117 | 0.00029 | 0.00159 | 0.00138 | 0.00298 | 0.00537 | 0.00003 | 0.00004 | 0.00004 |
| 0.959 | 0.00137 | 0.00031 | 0.00137 | 0.00142 | 0.00307 | 0.00559 | 0.00003 | 0.00004 | 0.00004 |
| 0.968 | 0.00194 | 0.00071 | 0.00013 | 0.00147 | 0.00315 | 0.00580 | 0.00003 | 0.00004 | 0.00004 |
| 0.977 | 0.00270 | 0.00120 | 0.00054 | 0.00152 | 0.00323 | 0.00599 | 0.00003 | 0.00004 | 0.00004 |
| 0.985 | 0.00287 | 0.00183 | 0.00010 | 0.00156 | 0.00329 | 0.00614 | 0.00003 | 0.00004 | 0.00004 |
| 0.994 | 0.00311 | 0.00103 | 0.00043 | 0.00161 | 0.00334 | 0.00627 | 0.00003 | 0.00004 | 0.00004 |
| 1.000 | 0.00239 | 0.00208 | 0.00030 | 0.00165 | 0.00338 | 0.00639 | 0.00003 | 0.00004 | 0.00004 |
| 1.010 | 0.00294 | 0.00105 | 0.00036 | 0.00169 | 0.00340 | 0.00650 | 0.00003 | 0.00004 | 0.00004 |
| 1.020 | 0.00329 | 0.00152 | 0.00034 | 0.00172 | 0.00341 | 0.00658 | 0.00003 | 0.00004 | 0.00004 |
| 1.030 | 0.00343 | 0.00131 | 0.00029 | 0.00175 | 0.00341 | 0.00664 | 0.00003 | 0.00004 | 0.00004 |
| 1.040 | 0.00258 | 0.00095 | 0.00030 | 0.00176 | 0.00340 | 0.00667 | 0.00003 | 0.00004 | 0.00004 |
| 1.050 | 0.00241 | 0.00157 | 0.00019 | 0.00177 | 0.00339 | 0.00671 | 0.00003 | 0.00004 | 0.00004 |
| 1.050 | 0.00286 | 0.00077 | 0.00031 | 0.00178 | 0.00339 | 0.00674 | 0.00003 | 0.00003 | 0.00004 |
| 1.060 | 0.00297 | 0.00111 | 0.00023 | 0.00178 | 0.00338 | 0.00674 | 0.00003 | 0.00003 | 0.00004 |
| 1.070 | 0.00229 | 0.00083 | 0.00022 | 0.00178 | 0.00338 | 0.00678 | 0.00003 | 0.00003 | 0.00004 |
| 1.080 | 0.00237 | 0.00076 | 0.00015 | 0.00177 | 0.00339 | 0.00682 | 0.00003 | 0.00003 | 0.00004 |
| 1.090 | 0.00301 | 0.00076 | 0.00013 | 0.00176 | 0.00341 | 0.00687 | 0.00003 | 0.00003 | 0.00004 |
| 1.100 | 0.00295 | 0.00067 | 0.00010 | 0.00176 | 0.00343 | 0.00693 | 0.00003 | 0.00003 | 0.00004 |
| 1.110 | 0.00268 | 0.00056 | 0.00015 | 0.00175 | 0.00347 | 0.00700 | 0.00003 | 0.00003 | 0.00004 |
| 1.110 | 0.00240 | 0.00047 | 0.00030 | 0.00175 | 0.00351 | 0.00708 | 0.00003 | 0.00003 | 0.00004 |
| 1.120 | 0.00217 | 0.00040 | 0.00047 | 0.00175 | 0.00356 | 0.00716 | 0.00003 | 0.00003 | 0.00004 |
| 1.130 | 0.00198 | 0.00034 | 0.00061 | 0.00175 | 0.00361 | 0.00724 | 0.00003 | 0.00003 | 0.00004 |
| 1.140 | 0.00181 | 0.00030 | 0.00074 | 0.00175 | 0.00367 | 0.00733 | 0.00003 | 0.00003 | 0.00004 |
| 1.150 | 0.00167 | 0.00026 | 0.00087 | 0.00175 | 0.00373 | 0.00743 | 0.00003 | 0.00003 | 0.00004 |
| 1.160 | 0.00154 | 0.00023 | 0.00102 | 0.00175 | 0.00380 | 0.00752 | 0.00003 | 0.00003 | 0.00004 |
| 1.170 | 0.00144 | 0.00021 | 0.00117 | 0.00175 | 0.00388 | 0.00761 | 0.00003 | 0.00003 | 0.00004 |
| 1.180 | 0.00134 | 0.00019 | 0.00132 | 0.00176 | 0.00397 | 0.00770 | 0.00003 | 0.00003 | 0.00003 |
| 1.180 | 0.00126 | 0.00017 | 0.00146 | 0.00177 | 0.00406 | 0.00779 | 0.00003 | 0.00003 | 0.00003 |
| 1.190 | 0.00118 | 0.00016 | 0.00159 | 0.00178 | 0.00417 | 0.00788 | 0.00003 | 0.00003 | 0.00003 |
| 1.200 | 0.00112 | 0.00015 | 0.00170 | 0.00179 | 0.00428 | 0.00797 | 0.00003 | 0.00003 | 0.00003 |
| 1.210 | 0.00106 | 0.00014 | 0.00179 | 0.00181 | 0.00439 | 0.00807 | 0.00003 | 0.00003 | 0.00003 |
| 1.220 | 0.00100 | 0.00013 | 0.00184 | 0.00183 | 0.00451 | 0.00816 | 0.00003 | 0.00003 | 0.00003 |
| 1.230 | 0.00095 | 0.00012 | 0.00187 | 0.00185 | 0.00463 | 0.00826 | 0.00003 | 0.00003 | 0.00003 |
| 1.240 | 0.00090 | 0.00012 | 0.00198 | 0.00187 | 0.00475 | 0.00834 | 0.00003 | 0.00003 | 0.00003 |
| 1.240 | 0.00086 | 0.00011 | 0.00215 | 0.00190 | 0.00486 | 0.00842 | 0.00003 | 0.00003 | 0.00003 |
| 1.250 | 0.00083 | 0.00011 | 0.00226 | 0.00193 | 0.00495 | 0.00847 | 0.00003 | 0.00003 | 0.00003 |
| 1.260 | 0.00102 | 0.00019 | 0.00141 | 0.00197 | 0.00503 | 0.00852 | 0.00003 | 0.00003 | 0.00003 |
| 1.270 | 0.00105 | 0.00024 | 0.00018 | 0.00200 | 0.00509 | 0.00854 | 0.00003 | 0.00003 | 0.00003 |
| 1.280 | 0.00217 | 0.00086 | 0.00047 | 0.00203 | 0.00514 | 0.00854 | 0.00003 | 0.00003 | 0.00003 |
| 1.290 | 0.00217 | 0.00085 | 0.00034 | 0.00206 | 0.00516 | 0.00852 | 0.00003 | 0.00003 | 0.00003 |
| 1.300 | 0.00252 | 0.00077 | 0.00058 | 0.00208 | 0.00519 | 0.00849 | 0.00003 | 0.00003 | 0.00003 |
| 1.310 | 0.00142 | 0.00117 | 0.00044 | 0.00211 | 0.00520 | 0.00843 | 0.00003 | 0.00003 | 0.00003 |
| 1.310 | 0.00257 | 0.00060 | 0.00036 | 0.00213 | 0.00522 | 0.00836 | 0.00003 | 0.00003 | 0.00003 |
| 1.320 | 0.00268 | 0.00086 | 0.00048 | 0.00215 | 0.00523 | 0.00829 | 0.00003 | 0.00003 | 0.00003 |
| 1.330 | 0.00276 | 0.00052 | 0.00028 | 0.00216 | 0.00524 | 0.00820 | 0.00003 | 0.00003 | 0.00003 |
| 1.340 | 0.00152 | 0.00074 | 0.00038 | 0.00217 | 0.00524 | 0.00809 | 0.00003 | 0.00003 | 0.00003 |
| 1.350 | 0.00221 | 0.00074 | 0.00033 | 0.00217 | 0.00524 | 0.00797 | 0.00003 | 0.00003 | 0.00003 |
| 1.360 | 0.00243 | 0.00056 | 0.00030 | 0.00218 | 0.00524 | 0.00786 | 0.00003 | 0.00003 | 0.00003 |
| 1.370 | 0.00274 | 0.00089 | 0.00028 | 0.00218 | 0.00523 | 0.00777 | 0.00002 | 0.00003 | 0.00003 |
| 1.370 | 0.00164 | 0.00056 | 0.00033 | 0.00217 | 0.00522 | 0.00768 | 0.00002 | 0.00003 | 0.00003 |
| 1.380 | 0.00210 | 0.00061 | 0.00021 | 0.00217 | 0.00521 | 0.00757 | 0.00002 | 0.00003 | 0.00003 |
| 1.390 | 0.00219 | 0.00059 | 0.00015 | 0.00217 | 0.00520 | 0.00748 | 0.00002 | 0.00003 | 0.00003 |
| 1.400 | 0.00204 | 0.00050 | 0.00009 | 0.00217 | 0.00521 | 0.00741 | 0.00002 | 0.00003 | 0.00003 |
| 1.410 | 0.00184 | 0.00042 | 0.00008 | 0.00217 | 0.00522 | 0.00731 | 0.00002 | 0.00003 | 0.00003 |
| 1.420 | 0.00167 | 0.00035 | 0.00017 | 0.00218 | 0.00524 | 0.00725 | 0.00002 | 0.00003 | 0.00003 |
| 1.430 | 0.00152 | 0.00030 | 0.00031 | 0.00218 | 0.00526 | 0.00721 | 0.00002 | 0.00003 | 0.00003 |
| 1.430 | 0.00139 | 0.00026 | 0.00046 | 0.00218 | 0.00529 | 0.00718 | 0.00002 | 0.00003 | 0.00003 |
| 1.440 | 0.00128 | 0.00022 | 0.00060 | 0.00219 | 0.00533 | 0.00715 | 0.00002 | 0.00003 | 0.00003 |
| 1.450 | 0.00119 | 0.00020 | 0.00075 | 0.00219 | 0.00536 | 0.00711 | 0.00002 | 0.00003 | 0.00003 |
| 1.460 | 0.00111 | 0.00017 | 0.00093 | 0.00220 | 0.00538 | 0.00708 | 0.00002 | 0.00003 | 0.00003 |
| 1.470 | 0.00104 | 0.00016 | 0.00114 | 0.00221 | 0.00540 | 0.00705 | 0.00002 | 0.00003 | 0.00003 |
| 1.480 | 0.00097 | 0.00014 | 0.00135 | 0.00222 | 0.00541 | 0.00701 | 0.00002 | 0.00003 | 0.00003 |
| 1.490 | 0.00091 | 0.00013 | 0.00157 | 0.00223 | 0.00541 | 0.00699 | 0.00002 | 0.00003 | 0.00003 |
| 1.500 | 0.00086 | 0.00012 | 0.00177 | 0.00224 | 0.00541 | 0.00696 | 0.00002 | 0.00003 | 0.00003 |
| 1.500 | 0.00081 | 0.00011 | 0.00197 | 0.00225 | 0.00542 | 0.00692 | 0.00002 | 0.00003 | 0.00003 |
| 1.510 | 0.00077 | 0.00010 | 0.00215 | 0.00227 | 0.00542 | 0.00692 | 0.00002 | 0.00003 | 0.00003 |
| 1.520 | 0.00073 | 0.00010 | 0.00233 | 0.00228 | 0.00543 | 0.00691 | 0.00002 | 0.00003 | 0.00003 |
| 1.530 | 0.00069 | 0.00010 | 0.00248 | 0.00230 | 0.00544 | 0.00689 | 0.00002 | 0.00003 | 0.00003 |
| 1.540 | 0.00066 | 0.00009 | 0.00262 | 0.00232 | 0.00545 | 0.00687 | 0.00002 | 0.00003 | 0.00003 |
| 1.550 | 0.00063 | 0.00009 | 0.00275 | 0.00233 | 0.00545 | 0.00684 | 0.00002 | 0.00003 | 0.00003 |
| 1.560 | 0.00060 | 0.00011 | 0.00286 | 0.00235 | 0.00546 | 0.00682 | 0.00002 | 0.00003 | 0.00003 |
| 1.560 | 0.00058 | 0.00019 | 0.00297 | 0.00237 | 0.00547 | 0.00678 | 0.00002 | 0.00003 | 0.00003 |
| 1.570 | 0.00055 | 0.00041 | 0.00307 | 0.00239 | 0.00549 | 0.00675 | 0.00002 | 0.00003 | 0.00003 |
| 1.580 | 0.00053 | 0.00066 | 0.00317 | 0.00241 | 0.00551 | 0.00674 | 0.00002 | 0.00003 | 0.00003 |
| 1.590 | 0.00051 | 0.00086 | 0.00326 | 0.00243 | 0.00553 | 0.00675 | 0.00002 | 0.00003 | 0.00003 |
| 1.600 | 0.00049 | 0.00102 | 0.00334 | 0.00244 | 0.00554 | 0.00675 | 0.00002 | 0.00003 | 0.00003 |
| 1.610 | 0.00047 | 0.00116 | 0.00341 | 0.00246 | 0.00555 | 0.00674 | 0.00002 | 0.00003 | 0.00003 |
| 1.620 | 0.00045 | 0.00129 | 0.00346 | 0.00248 | 0.00555 | 0.00673 | 0.00002 | 0.00002 | 0.00003 |
| 1.620 | 0.00044 | 0.00141 | 0.00350 | 0.00249 | 0.00554 | 0.00672 | 0.00002 | 0.00002 | 0.00003 |
| 1.630 | 0.00042 | 0.00153 | 0.00354 | 0.00250 | 0.00552 | 0.00672 | 0.00002 | 0.00002 | 0.00003 |
| 1.640 | 0.00041 | 0.00163 | 0.00357 | 0.00252 | 0.00550 | 0.00671 | 0.00002 | 0.00002 | 0.00003 |
| 1.650 | 0.00040 | 0.00174 | 0.00360 | 0.00253 | 0.00548 | 0.00670 | 0.00002 | 0.00002 | 0.00003 |
| 1.660 | 0.00039 | 0.00183 | 0.00361 | 0.00254 | 0.00545 | 0.00670 | 0.00002 | 0.00002 | 0.00003 |
| 1.670 | 0.00038 | 0.00192 | 0.00363 | 0.00255 | 0.00542 | 0.00669 | 0.00002 | 0.00002 | 0.00003 |
| 1.680 | 0.00037 | 0.00200 | 0.00363 | 0.00256 | 0.00540 | 0.00669 | 0.00002 | 0.00002 | 0.00003 |
| 1.690 | 0.00036 | 0.00206 | 0.00364 | 0.00257 | 0.00539 | 0.00669 | 0.00002 | 0.00002 | 0.00003 |
| 1.690 | 0.00035 | 0.00212 | 0.00364 | 0.00258 | 0.00537 | 0.00669 | 0.00002 | 0.00002 | 0.00003 |
| 1.700 | 0.00034 | 0.00217 | 0.00365 | 0.00259 | 0.00537 | 0.00670 | 0.00002 | 0.00002 | 0.00003 |
| 1.710 | 0.00033 | 0.00220 | 0.00366 | 0.00261 | 0.00538 | 0.00671 | 0.00002 | 0.00002 | 0.00003 |
| 1.720 | 0.00033 | 0.00223 | 0.00369 | 0.00262 | 0.00540 | 0.00671 | 0.00002 | 0.00002 | 0.00003 |

**Table. 18** In the submerged state (*h*=0.09 m), the Turbulent kinetic energy *TKE* of three vegetation patch coverage conditions changes along the *x* direction.

| **Hydraulic parameters** | **Turbulent kinetic energy *TKE* (m^2^/s^2^)** | | | | | | | | |
| --- | --- | --- | --- | --- | --- | --- | --- | --- | --- |
| ***x* (m)** | **L_1-1_** | | | **L_3-3_** | | | **L_4-4_** | | |
|  | ***Cr*=2.09%** | ***Cr*=4.71%** | ***Cr*=8.37%** | ***Cr*=2.09%** | ***Cr*=4.71%** | ***Cr*=8.37%** | ***Cr*=2.09%** | ***Cr*=4.71%** | ***Cr*=8.37%** |
| 0.000 | 0.00034 | 0.00034 | 0.00034 | 0.00034 | 0.00034 | 0.00034 | 0.00034 | 0.00034 | 0.00034 |
| 0.009 | 0.00031 | 0.00031 | 0.00031 | 0.00031 | 0.00031 | 0.00031 | 0.00031 | 0.00031 | 0.00031 |
| 0.017 | 0.00029 | 0.00029 | 0.00029 | 0.00029 | 0.00029 | 0.00029 | 0.00029 | 0.00029 | 0.00029 |
| 0.026 | 0.00027 | 0.00027 | 0.00027 | 0.00027 | 0.00027 | 0.00027 | 0.00027 | 0.00027 | 0.00027 |
| 0.035 | 0.00025 | 0.00025 | 0.00025 | 0.00025 | 0.00025 | 0.00025 | 0.00025 | 0.00025 | 0.00025 |
| 0.043 | 0.00024 | 0.00023 | 0.00023 | 0.00023 | 0.00024 | 0.00023 | 0.00023 | 0.00024 | 0.00024 |
| 0.052 | 0.00022 | 0.00022 | 0.00022 | 0.00022 | 0.00022 | 0.00022 | 0.00022 | 0.00022 | 0.00022 |
| 0.061 | 0.00021 | 0.00021 | 0.00021 | 0.00021 | 0.00021 | 0.00021 | 0.00021 | 0.00021 | 0.00021 |
| 0.069 | 0.00020 | 0.00020 | 0.00020 | 0.00020 | 0.00020 | 0.00020 | 0.00020 | 0.00020 | 0.00020 |
| 0.078 | 0.00019 | 0.00019 | 0.00019 | 0.00019 | 0.00019 | 0.00019 | 0.00019 | 0.00019 | 0.00019 |
| 0.086 | 0.00018 | 0.00018 | 0.00018 | 0.00018 | 0.00018 | 0.00018 | 0.00018 | 0.00018 | 0.00018 |
| 0.095 | 0.00017 | 0.00017 | 0.00017 | 0.00017 | 0.00017 | 0.00017 | 0.00017 | 0.00017 | 0.00017 |
| 0.104 | 0.00016 | 0.00016 | 0.00016 | 0.00016 | 0.00016 | 0.00016 | 0.00016 | 0.00016 | 0.00016 |
| 0.112 | 0.00016 | 0.00016 | 0.00016 | 0.00016 | 0.00016 | 0.00016 | 0.00016 | 0.00016 | 0.00016 |
| 0.121 | 0.00015 | 0.00015 | 0.00015 | 0.00015 | 0.00015 | 0.00015 | 0.00015 | 0.00015 | 0.00015 |
| 0.130 | 0.00014 | 0.00014 | 0.00014 | 0.00014 | 0.00014 | 0.00014 | 0.00014 | 0.00014 | 0.00014 |
| 0.138 | 0.00014 | 0.00014 | 0.00014 | 0.00014 | 0.00014 | 0.00014 | 0.00014 | 0.00014 | 0.00014 |
| 0.147 | 0.00013 | 0.00013 | 0.00013 | 0.00013 | 0.00013 | 0.00013 | 0.00013 | 0.00013 | 0.00013 |
| 0.156 | 0.00013 | 0.00013 | 0.00013 | 0.00013 | 0.00013 | 0.00013 | 0.00013 | 0.00013 | 0.00013 |
| 0.164 | 0.00012 | 0.00012 | 0.00012 | 0.00012 | 0.00012 | 0.00012 | 0.00013 | 0.00013 | 0.00012 |
| 0.173 | 0.00012 | 0.00012 | 0.00012 | 0.00012 | 0.00012 | 0.00012 | 0.00012 | 0.00012 | 0.00012 |
| 0.182 | 0.00012 | 0.00012 | 0.00012 | 0.00012 | 0.00012 | 0.00012 | 0.00012 | 0.00012 | 0.00012 |
| 0.190 | 0.00011 | 0.00011 | 0.00011 | 0.00011 | 0.00011 | 0.00011 | 0.00011 | 0.00011 | 0.00011 |
| 0.199 | 0.00011 | 0.00011 | 0.00011 | 0.00011 | 0.00011 | 0.00011 | 0.00011 | 0.00011 | 0.00011 |
| 0.207 | 0.00011 | 0.00011 | 0.00011 | 0.00011 | 0.00011 | 0.00011 | 0.00011 | 0.00011 | 0.00011 |
| 0.216 | 0.00010 | 0.00010 | 0.00010 | 0.00010 | 0.00010 | 0.00010 | 0.00010 | 0.00010 | 0.00010 |
| 0.225 | 0.00010 | 0.00010 | 0.00010 | 0.00010 | 0.00010 | 0.00010 | 0.00010 | 0.00010 | 0.00010 |
| 0.233 | 0.00010 | 0.00010 | 0.00010 | 0.00010 | 0.00010 | 0.00010 | 0.00010 | 0.00010 | 0.00010 |
| 0.242 | 0.00009 | 0.00009 | 0.00009 | 0.00009 | 0.00009 | 0.00009 | 0.00010 | 0.00010 | 0.00010 |
| 0.251 | 0.00009 | 0.00009 | 0.00009 | 0.00009 | 0.00009 | 0.00009 | 0.00009 | 0.00009 | 0.00009 |
| 0.259 | 0.00009 | 0.00009 | 0.00009 | 0.00009 | 0.00009 | 0.00009 | 0.00009 | 0.00009 | 0.00009 |
| 0.268 | 0.00009 | 0.00009 | 0.00009 | 0.00009 | 0.00009 | 0.00009 | 0.00009 | 0.00009 | 0.00009 |
| 0.277 | 0.00008 | 0.00008 | 0.00008 | 0.00009 | 0.00009 | 0.00009 | 0.00009 | 0.00009 | 0.00009 |
| 0.285 | 0.00008 | 0.00008 | 0.00008 | 0.00008 | 0.00008 | 0.00008 | 0.00008 | 0.00008 | 0.00009 |
| 0.294 | 0.00008 | 0.00008 | 0.00008 | 0.00008 | 0.00008 | 0.00008 | 0.00008 | 0.00008 | 0.00008 |
| 0.303 | 0.00008 | 0.00008 | 0.00008 | 0.00008 | 0.00008 | 0.00008 | 0.00008 | 0.00008 | 0.00008 |
| 0.311 | 0.00008 | 0.00008 | 0.00008 | 0.00008 | 0.00008 | 0.00008 | 0.00008 | 0.00008 | 0.00008 |
| 0.320 | 0.00008 | 0.00008 | 0.00007 | 0.00008 | 0.00008 | 0.00008 | 0.00008 | 0.00008 | 0.00008 |
| 0.328 | 0.00007 | 0.00007 | 0.00007 | 0.00007 | 0.00008 | 0.00008 | 0.00008 | 0.00008 | 0.00008 |
| 0.337 | 0.00007 | 0.00007 | 0.00007 | 0.00007 | 0.00007 | 0.00007 | 0.00007 | 0.00007 | 0.00008 |
| 0.346 | 0.00007 | 0.00007 | 0.00007 | 0.00007 | 0.00007 | 0.00007 | 0.00007 | 0.00007 | 0.00007 |
| 0.354 | 0.00007 | 0.00007 | 0.00007 | 0.00007 | 0.00007 | 0.00007 | 0.00007 | 0.00007 | 0.00007 |
| 0.363 | 0.00007 | 0.00007 | 0.00007 | 0.00007 | 0.00007 | 0.00007 | 0.00007 | 0.00007 | 0.00007 |
| 0.372 | 0.00007 | 0.00007 | 0.00008 | 0.00007 | 0.00007 | 0.00007 | 0.00007 | 0.00007 | 0.00007 |
| 0.380 | 0.00007 | 0.00007 | 0.00009 | 0.00007 | 0.00007 | 0.00007 | 0.00007 | 0.00007 | 0.00007 |
| 0.389 | 0.00007 | 0.00007 | 0.00025 | 0.00007 | 0.00007 | 0.00007 | 0.00007 | 0.00007 | 0.00007 |
| 0.398 | 0.00006 | 0.00007 | 0.00117 | 0.00006 | 0.00007 | 0.00007 | 0.00006 | 0.00007 | 0.00007 |
| 0.406 | 0.00006 | 0.00007 | 0.00103 | 0.00006 | 0.00006 | 0.00007 | 0.00006 | 0.00006 | 0.00007 |
| 0.415 | 0.00006 | 0.00007 | 0.00109 | 0.00006 | 0.00006 | 0.00007 | 0.00006 | 0.00006 | 0.00007 |
| 0.424 | 0.00006 | 0.00007 | 0.00115 | 0.00006 | 0.00006 | 0.00006 | 0.00006 | 0.00006 | 0.00006 |
| 0.432 | 0.00007 | 0.00007 | 0.00106 | 0.00006 | 0.00006 | 0.00006 | 0.00006 | 0.00006 | 0.00006 |
| 0.441 | 0.00007 | 0.00008 | 0.00119 | 0.00006 | 0.00006 | 0.00006 | 0.00006 | 0.00006 | 0.00006 |
| 0.449 | 0.00007 | 0.00009 | 0.00136 | 0.00006 | 0.00006 | 0.00006 | 0.00006 | 0.00006 | 0.00006 |
| 0.458 | 0.00009 | 0.00009 | 0.00155 | 0.00006 | 0.00006 | 0.00006 | 0.00006 | 0.00006 | 0.00006 |
| 0.467 | 0.00009 | 0.00013 | 0.00185 | 0.00006 | 0.00006 | 0.00006 | 0.00006 | 0.00006 | 0.00006 |
| 0.475 | 0.00009 | 0.00016 | 0.00200 | 0.00006 | 0.00006 | 0.00006 | 0.00006 | 0.00006 | 0.00006 |
| 0.484 | 0.00012 | 0.00024 | 0.00219 | 0.00005 | 0.00006 | 0.00006 | 0.00006 | 0.00006 | 0.00006 |
| 0.493 | 0.00014 | 0.00040 | 0.00202 | 0.00005 | 0.00006 | 0.00006 | 0.00005 | 0.00006 | 0.00006 |
| 0.501 | 0.00020 | 0.00067 | 0.00240 | 0.00005 | 0.00006 | 0.00007 | 0.00005 | 0.00006 | 0.00006 |
| 0.510 | 0.00020 | 0.00066 | 0.00242 | 0.00005 | 0.00005 | 0.00006 | 0.00005 | 0.00005 | 0.00006 |
| 0.519 | 0.00023 | 0.00060 | 0.00230 | 0.00005 | 0.00006 | 0.00007 | 0.00005 | 0.00005 | 0.00006 |
| 0.527 | 0.00022 | 0.00067 | 0.00258 | 0.00005 | 0.00005 | 0.00007 | 0.00005 | 0.00005 | 0.00006 |
| 0.536 | 0.00021 | 0.00059 | 0.00236 | 0.00005 | 0.00005 | 0.00007 | 0.00005 | 0.00005 | 0.00006 |
| 0.545 | 0.00020 | 0.00054 | 0.00263 | 0.00005 | 0.00005 | 0.00007 | 0.00005 | 0.00005 | 0.00005 |
| 0.553 | 0.00019 | 0.00056 | 0.00312 | 0.00005 | 0.00005 | 0.00008 | 0.00005 | 0.00005 | 0.00005 |
| 0.562 | 0.00019 | 0.00056 | 0.00316 | 0.00005 | 0.00005 | 0.00009 | 0.00005 | 0.00005 | 0.00005 |
| 0.570 | 0.00018 | 0.00054 | 0.00293 | 0.00005 | 0.00005 | 0.00009 | 0.00005 | 0.00005 | 0.00005 |
| 0.579 | 0.00016 | 0.00053 | 0.00295 | 0.00005 | 0.00005 | 0.00008 | 0.00005 | 0.00005 | 0.00005 |
| 0.588 | 0.00016 | 0.00056 | 0.00343 | 0.00005 | 0.00005 | 0.00010 | 0.00005 | 0.00005 | 0.00005 |
| 0.596 | 0.00015 | 0.00054 | 0.00363 | 0.00005 | 0.00006 | 0.00011 | 0.00005 | 0.00005 | 0.00005 |
| 0.605 | 0.00014 | 0.00069 | 0.00360 | 0.00005 | 0.00006 | 0.00012 | 0.00005 | 0.00005 | 0.00005 |
| 0.614 | 0.00014 | 0.00071 | 0.00362 | 0.00005 | 0.00006 | 0.00014 | 0.00005 | 0.00005 | 0.00005 |
| 0.622 | 0.00014 | 0.00068 | 0.00376 | 0.00004 | 0.00007 | 0.00019 | 0.00004 | 0.00005 | 0.00005 |
| 0.631 | 0.00015 | 0.00077 | 0.00399 | 0.00004 | 0.00006 | 0.00019 | 0.00004 | 0.00005 | 0.00005 |
| 0.640 | 0.00014 | 0.00083 | 0.00419 | 0.00004 | 0.00006 | 0.00023 | 0.00004 | 0.00005 | 0.00005 |
| 0.648 | 0.00014 | 0.00089 | 0.00462 | 0.00004 | 0.00007 | 0.00030 | 0.00004 | 0.00005 | 0.00005 |
| 0.657 | 0.00014 | 0.00105 | 0.00444 | 0.00004 | 0.00007 | 0.00031 | 0.00004 | 0.00005 | 0.00005 |
| 0.666 | 0.00014 | 0.00113 | 0.00440 | 0.00004 | 0.00007 | 0.00032 | 0.00004 | 0.00004 | 0.00005 |
| 0.674 | 0.00013 | 0.00124 | 0.00439 | 0.00004 | 0.00007 | 0.00040 | 0.00004 | 0.00004 | 0.00005 |
| 0.683 | 0.00013 | 0.00132 | 0.00418 | 0.00004 | 0.00008 | 0.00044 | 0.00004 | 0.00004 | 0.00005 |
| 0.691 | 0.00014 | 0.00139 | 0.00393 | 0.00004 | 0.00007 | 0.00049 | 0.00004 | 0.00004 | 0.00005 |
| 0.700 | 0.00013 | 0.00146 | 0.00365 | 0.00004 | 0.00008 | 0.00054 | 0.00004 | 0.00004 | 0.00005 |
| 0.709 | 0.00013 | 0.00153 | 0.00342 | 0.00004 | 0.00008 | 0.00056 | 0.00004 | 0.00004 | 0.00005 |
| 0.717 | 0.00014 | 0.00159 | 0.00322 | 0.00004 | 0.00007 | 0.00064 | 0.00004 | 0.00004 | 0.00005 |
| 0.726 | 0.00014 | 0.00164 | 0.00304 | 0.00004 | 0.00008 | 0.00070 | 0.00004 | 0.00004 | 0.00004 |
| 0.735 | 0.00016 | 0.00178 | 0.00292 | 0.00004 | 0.00007 | 0.00077 | 0.00004 | 0.00004 | 0.00004 |
| 0.743 | 0.00016 | 0.00191 | 0.00282 | 0.00004 | 0.00009 | 0.00086 | 0.00004 | 0.00004 | 0.00004 |
| 0.752 | 0.00017 | 0.00201 | 0.00269 | 0.00004 | 0.00008 | 0.00092 | 0.00004 | 0.00004 | 0.00004 |
| 0.761 | 0.00022 | 0.00221 | 0.00261 | 0.00004 | 0.00011 | 0.00097 | 0.00004 | 0.00004 | 0.00004 |
| 0.769 | 0.00022 | 0.00244 | 0.00260 | 0.00004 | 0.00012 | 0.00101 | 0.00004 | 0.00004 | 0.00004 |
| 0.778 | 0.00027 | 0.00254 | 0.00268 | 0.00004 | 0.00010 | 0.00108 | 0.00004 | 0.00004 | 0.00004 |
| 0.787 | 0.00030 | 0.00242 | 0.00266 | 0.00004 | 0.00010 | 0.00113 | 0.00004 | 0.00004 | 0.00004 |
| 0.795 | 0.00033 | 0.00239 | 0.00265 | 0.00004 | 0.00010 | 0.00116 | 0.00004 | 0.00004 | 0.00004 |
| 0.804 | 0.00032 | 0.00220 | 0.00258 | 0.00004 | 0.00012 | 0.00120 | 0.00004 | 0.00004 | 0.00004 |
| 0.812 | 0.00031 | 0.00188 | 0.00254 | 0.00004 | 0.00015 | 0.00118 | 0.00004 | 0.00004 | 0.00004 |
| 0.821 | 0.00028 | 0.00187 | 0.00252 | 0.00004 | 0.00013 | 0.00124 | 0.00004 | 0.00004 | 0.00004 |
| 0.830 | 0.00024 | 0.00170 | 0.00238 | 0.00004 | 0.00014 | 0.00127 | 0.00004 | 0.00004 | 0.00004 |
| 0.838 | 0.00021 | 0.00182 | 0.00244 | 0.00004 | 0.00016 | 0.00129 | 0.00003 | 0.00004 | 0.00004 |
| 0.847 | 0.00022 | 0.00181 | 0.00244 | 0.00004 | 0.00019 | 0.00134 | 0.00003 | 0.00004 | 0.00004 |
| 0.856 | 0.00021 | 0.00191 | 0.00244 | 0.00004 | 0.00016 | 0.00133 | 0.00003 | 0.00004 | 0.00004 |
| 0.864 | 0.00018 | 0.00190 | 0.00237 | 0.00003 | 0.00016 | 0.00138 | 0.00003 | 0.00004 | 0.00004 |
| 0.873 | 0.00020 | 0.00189 | 0.00252 | 0.00003 | 0.00018 | 0.00139 | 0.00003 | 0.00004 | 0.00004 |
| 0.882 | 0.00020 | 0.00195 | 0.00248 | 0.00003 | 0.00022 | 0.00142 | 0.00003 | 0.00004 | 0.00004 |
| 0.890 | 0.00020 | 0.00201 | 0.00242 | 0.00004 | 0.00019 | 0.00141 | 0.00003 | 0.00004 | 0.00004 |
| 0.899 | 0.00019 | 0.00209 | 0.00249 | 0.00003 | 0.00021 | 0.00141 | 0.00003 | 0.00004 | 0.00004 |
| 0.908 | 0.00019 | 0.00213 | 0.00244 | 0.00003 | 0.00024 | 0.00140 | 0.00003 | 0.00004 | 0.00004 |
| 0.916 | 0.00022 | 0.00210 | 0.00244 | 0.00004 | 0.00025 | 0.00140 | 0.00003 | 0.00004 | 0.00004 |
| 0.925 | 0.00022 | 0.00219 | 0.00251 | 0.00004 | 0.00029 | 0.00141 | 0.00003 | 0.00003 | 0.00004 |
| 0.933 | 0.00020 | 0.00218 | 0.00261 | 0.00004 | 0.00031 | 0.00140 | 0.00003 | 0.00003 | 0.00004 |
| 0.942 | 0.00020 | 0.00234 | 0.00269 | 0.00004 | 0.00029 | 0.00142 | 0.00003 | 0.00003 | 0.00004 |
| 0.951 | 0.00021 | 0.00244 | 0.00287 | 0.00003 | 0.00030 | 0.00141 | 0.00003 | 0.00003 | 0.00004 |
| 0.959 | 0.00022 | 0.00251 | 0.00288 | 0.00004 | 0.00032 | 0.00143 | 0.00003 | 0.00003 | 0.00004 |
| 0.968 | 0.00022 | 0.00255 | 0.00279 | 0.00003 | 0.00035 | 0.00141 | 0.00003 | 0.00003 | 0.00004 |
| 0.977 | 0.00022 | 0.00248 | 0.00273 | 0.00004 | 0.00040 | 0.00142 | 0.00003 | 0.00003 | 0.00004 |
| 0.985 | 0.00024 | 0.00243 | 0.00259 | 0.00004 | 0.00043 | 0.00141 | 0.00003 | 0.00003 | 0.00004 |
| 0.994 | 0.00023 | 0.00222 | 0.00249 | 0.00004 | 0.00043 | 0.00145 | 0.00003 | 0.00003 | 0.00004 |
| 1.000 | 0.00024 | 0.00203 | 0.00245 | 0.00004 | 0.00043 | 0.00145 | 0.00003 | 0.00003 | 0.00004 |
| 1.010 | 0.00025 | 0.00187 | 0.00238 | 0.00004 | 0.00046 | 0.00144 | 0.00003 | 0.00003 | 0.00004 |
| 1.020 | 0.00027 | 0.00172 | 0.00229 | 0.00004 | 0.00049 | 0.00144 | 0.00003 | 0.00003 | 0.00003 |
| 1.030 | 0.00028 | 0.00161 | 0.00223 | 0.00004 | 0.00048 | 0.00148 | 0.00003 | 0.00003 | 0.00003 |
| 1.040 | 0.00029 | 0.00151 | 0.00219 | 0.00004 | 0.00052 | 0.00147 | 0.00003 | 0.00003 | 0.00003 |
| 1.050 | 0.00032 | 0.00143 | 0.00213 | 0.00004 | 0.00055 | 0.00148 | 0.00003 | 0.00003 | 0.00003 |
| 1.050 | 0.00038 | 0.00137 | 0.00207 | 0.00004 | 0.00056 | 0.00156 | 0.00003 | 0.00003 | 0.00003 |
| 1.060 | 0.00039 | 0.00132 | 0.00203 | 0.00004 | 0.00059 | 0.00154 | 0.00003 | 0.00003 | 0.00003 |
| 1.070 | 0.00045 | 0.00127 | 0.00196 | 0.00004 | 0.00061 | 0.00154 | 0.00003 | 0.00003 | 0.00003 |
| 1.080 | 0.00043 | 0.00123 | 0.00190 | 0.00004 | 0.00060 | 0.00154 | 0.00003 | 0.00003 | 0.00003 |
| 1.090 | 0.00048 | 0.00122 | 0.00188 | 0.00004 | 0.00060 | 0.00157 | 0.00003 | 0.00003 | 0.00003 |
| 1.100 | 0.00043 | 0.00121 | 0.00185 | 0.00004 | 0.00064 | 0.00159 | 0.00003 | 0.00003 | 0.00003 |
| 1.110 | 0.00046 | 0.00121 | 0.00183 | 0.00004 | 0.00063 | 0.00160 | 0.00003 | 0.00003 | 0.00003 |
| 1.110 | 0.00041 | 0.00120 | 0.00185 | 0.00004 | 0.00064 | 0.00159 | 0.00003 | 0.00003 | 0.00003 |
| 1.120 | 0.00039 | 0.00122 | 0.00179 | 0.00004 | 0.00067 | 0.00162 | 0.00003 | 0.00003 | 0.00003 |
| 1.130 | 0.00038 | 0.00120 | 0.00177 | 0.00004 | 0.00068 | 0.00162 | 0.00003 | 0.00003 | 0.00003 |
| 1.140 | 0.00037 | 0.00118 | 0.00184 | 0.00004 | 0.00069 | 0.00161 | 0.00003 | 0.00003 | 0.00003 |
| 1.150 | 0.00035 | 0.00120 | 0.00184 | 0.00004 | 0.00071 | 0.00164 | 0.00003 | 0.00003 | 0.00003 |
| 1.160 | 0.00034 | 0.00122 | 0.00182 | 0.00004 | 0.00074 | 0.00164 | 0.00003 | 0.00003 | 0.00003 |
| 1.170 | 0.00033 | 0.00122 | 0.00181 | 0.00004 | 0.00072 | 0.00164 | 0.00003 | 0.00003 | 0.00003 |
| 1.180 | 0.00032 | 0.00119 | 0.00180 | 0.00004 | 0.00076 | 0.00166 | 0.00003 | 0.00003 | 0.00003 |
| 1.180 | 0.00030 | 0.00120 | 0.00182 | 0.00004 | 0.00080 | 0.00167 | 0.00003 | 0.00003 | 0.00003 |
| 1.190 | 0.00028 | 0.00121 | 0.00183 | 0.00005 | 0.00079 | 0.00169 | 0.00003 | 0.00003 | 0.00003 |
| 1.200 | 0.00029 | 0.00121 | 0.00186 | 0.00004 | 0.00081 | 0.00172 | 0.00003 | 0.00003 | 0.00003 |
| 1.210 | 0.00029 | 0.00123 | 0.00188 | 0.00005 | 0.00081 | 0.00176 | 0.00003 | 0.00003 | 0.00003 |
| 1.220 | 0.00032 | 0.00126 | 0.00198 | 0.00005 | 0.00082 | 0.00178 | 0.00003 | 0.00003 | 0.00003 |
| 1.230 | 0.00033 | 0.00134 | 0.00197 | 0.00005 | 0.00086 | 0.00179 | 0.00003 | 0.00003 | 0.00003 |
| 1.240 | 0.00032 | 0.00142 | 0.00196 | 0.00005 | 0.00091 | 0.00182 | 0.00002 | 0.00003 | 0.00003 |
| 1.240 | 0.00031 | 0.00151 | 0.00199 | 0.00006 | 0.00089 | 0.00184 | 0.00002 | 0.00003 | 0.00003 |
| 1.250 | 0.00032 | 0.00166 | 0.00207 | 0.00006 | 0.00094 | 0.00188 | 0.00002 | 0.00003 | 0.00003 |
| 1.260 | 0.00034 | 0.00171 | 0.00213 | 0.00006 | 0.00095 | 0.00190 | 0.00002 | 0.00003 | 0.00003 |
| 1.270 | 0.00034 | 0.00167 | 0.00212 | 0.00006 | 0.00100 | 0.00188 | 0.00002 | 0.00003 | 0.00003 |
| 1.280 | 0.00034 | 0.00158 | 0.00214 | 0.00007 | 0.00102 | 0.00194 | 0.00002 | 0.00003 | 0.00003 |
| 1.290 | 0.00034 | 0.00144 | 0.00209 | 0.00007 | 0.00099 | 0.00195 | 0.00002 | 0.00003 | 0.00003 |
| 1.300 | 0.00035 | 0.00130 | 0.00202 | 0.00006 | 0.00103 | 0.00197 | 0.00002 | 0.00003 | 0.00003 |
| 1.310 | 0.00036 | 0.00118 | 0.00196 | 0.00007 | 0.00103 | 0.00201 | 0.00002 | 0.00003 | 0.00003 |
| 1.310 | 0.00036 | 0.00110 | 0.00189 | 0.00007 | 0.00104 | 0.00206 | 0.00002 | 0.00003 | 0.00003 |
| 1.320 | 0.00037 | 0.00103 | 0.00183 | 0.00008 | 0.00104 | 0.00206 | 0.00002 | 0.00003 | 0.00003 |
| 1.330 | 0.00038 | 0.00097 | 0.00176 | 0.00008 | 0.00108 | 0.00209 | 0.00002 | 0.00003 | 0.00003 |
| 1.340 | 0.00037 | 0.00092 | 0.00170 | 0.00008 | 0.00108 | 0.00214 | 0.00002 | 0.00003 | 0.00003 |
| 1.350 | 0.00038 | 0.00088 | 0.00164 | 0.00007 | 0.00113 | 0.00216 | 0.00002 | 0.00003 | 0.00003 |
| 1.360 | 0.00039 | 0.00087 | 0.00159 | 0.00007 | 0.00116 | 0.00216 | 0.00002 | 0.00003 | 0.00003 |
| 1.370 | 0.00039 | 0.00085 | 0.00155 | 0.00007 | 0.00114 | 0.00217 | 0.00002 | 0.00003 | 0.00003 |
| 1.370 | 0.00039 | 0.00085 | 0.00151 | 0.00008 | 0.00116 | 0.00218 | 0.00002 | 0.00003 | 0.00003 |
| 1.380 | 0.00040 | 0.00085 | 0.00151 | 0.00009 | 0.00114 | 0.00219 | 0.00002 | 0.00003 | 0.00003 |
| 1.390 | 0.00041 | 0.00084 | 0.00152 | 0.00008 | 0.00113 | 0.00222 | 0.00002 | 0.00003 | 0.00003 |
| 1.400 | 0.00047 | 0.00079 | 0.00153 | 0.00007 | 0.00112 | 0.00223 | 0.00002 | 0.00003 | 0.00003 |
| 1.410 | 0.00047 | 0.00080 | 0.00148 | 0.00008 | 0.00117 | 0.00223 | 0.00002 | 0.00002 | 0.00003 |
| 1.420 | 0.00050 | 0.00080 | 0.00150 | 0.00008 | 0.00120 | 0.00225 | 0.00002 | 0.00002 | 0.00003 |
| 1.430 | 0.00045 | 0.00085 | 0.00145 | 0.00008 | 0.00118 | 0.00227 | 0.00002 | 0.00002 | 0.00003 |
| 1.430 | 0.00044 | 0.00085 | 0.00146 | 0.00008 | 0.00119 | 0.00227 | 0.00002 | 0.00002 | 0.00003 |
| 1.440 | 0.00045 | 0.00086 | 0.00142 | 0.00008 | 0.00122 | 0.00230 | 0.00002 | 0.00002 | 0.00003 |
| 1.450 | 0.00045 | 0.00086 | 0.00140 | 0.00008 | 0.00122 | 0.00233 | 0.00002 | 0.00002 | 0.00003 |
| 1.460 | 0.00049 | 0.00092 | 0.00140 | 0.00008 | 0.00123 | 0.00231 | 0.00002 | 0.00002 | 0.00003 |
| 1.470 | 0.00049 | 0.00090 | 0.00136 | 0.00009 | 0.00123 | 0.00239 | 0.00002 | 0.00002 | 0.00003 |
| 1.480 | 0.00048 | 0.00097 | 0.00134 | 0.00009 | 0.00128 | 0.00241 | 0.00002 | 0.00002 | 0.00003 |
| 1.490 | 0.00048 | 0.00108 | 0.00133 | 0.00009 | 0.00127 | 0.00245 | 0.00002 | 0.00002 | 0.00003 |
| 1.500 | 0.00049 | 0.00105 | 0.00133 | 0.00009 | 0.00128 | 0.00246 | 0.00002 | 0.00002 | 0.00003 |
| 1.500 | 0.00049 | 0.00103 | 0.00132 | 0.00009 | 0.00127 | 0.00249 | 0.00002 | 0.00002 | 0.00003 |
| 1.510 | 0.00050 | 0.00106 | 0.00132 | 0.00010 | 0.00128 | 0.00255 | 0.00002 | 0.00002 | 0.00003 |
| 1.520 | 0.00050 | 0.00105 | 0.00132 | 0.00010 | 0.00128 | 0.00260 | 0.00002 | 0.00002 | 0.00003 |
| 1.530 | 0.00052 | 0.00105 | 0.00132 | 0.00010 | 0.00129 | 0.00266 | 0.00002 | 0.00002 | 0.00003 |
| 1.540 | 0.00052 | 0.00105 | 0.00132 | 0.00010 | 0.00128 | 0.00272 | 0.00002 | 0.00002 | 0.00003 |
| 1.550 | 0.00052 | 0.00104 | 0.00132 | 0.00009 | 0.00134 | 0.00276 | 0.00002 | 0.00002 | 0.00003 |
| 1.560 | 0.00050 | 0.00102 | 0.00129 | 0.00009 | 0.00134 | 0.00285 | 0.00002 | 0.00002 | 0.00003 |
| 1.560 | 0.00052 | 0.00099 | 0.00132 | 0.00009 | 0.00134 | 0.00292 | 0.00002 | 0.00002 | 0.00002 |
| 1.570 | 0.00053 | 0.00096 | 0.00133 | 0.00009 | 0.00135 | 0.00301 | 0.00002 | 0.00002 | 0.00002 |
| 1.580 | 0.00056 | 0.00093 | 0.00134 | 0.00011 | 0.00137 | 0.00307 | 0.00002 | 0.00002 | 0.00002 |
| 1.590 | 0.00056 | 0.00090 | 0.00135 | 0.00010 | 0.00137 | 0.00318 | 0.00002 | 0.00002 | 0.00002 |
| 1.600 | 0.00057 | 0.00088 | 0.00134 | 0.00010 | 0.00137 | 0.00325 | 0.00002 | 0.00002 | 0.00002 |
| 1.610 | 0.00058 | 0.00086 | 0.00132 | 0.00011 | 0.00137 | 0.00337 | 0.00002 | 0.00002 | 0.00002 |
| 1.620 | 0.00057 | 0.00087 | 0.00133 | 0.00011 | 0.00137 | 0.00348 | 0.00002 | 0.00002 | 0.00002 |
| 1.620 | 0.00056 | 0.00089 | 0.00132 | 0.00011 | 0.00138 | 0.00358 | 0.00002 | 0.00002 | 0.00002 |
| 1.630 | 0.00057 | 0.00092 | 0.00129 | 0.00011 | 0.00141 | 0.00371 | 0.00002 | 0.00002 | 0.00002 |
| 1.640 | 0.00059 | 0.00097 | 0.00130 | 0.00010 | 0.00142 | 0.00382 | 0.00002 | 0.00002 | 0.00002 |
| 1.650 | 0.00057 | 0.00105 | 0.00130 | 0.00010 | 0.00144 | 0.00395 | 0.00002 | 0.00002 | 0.00002 |
| 1.660 | 0.00056 | 0.00112 | 0.00131 | 0.00010 | 0.00146 | 0.00407 | 0.00002 | 0.00002 | 0.00002 |
| 1.670 | 0.00057 | 0.00119 | 0.00133 | 0.00010 | 0.00146 | 0.00420 | 0.00002 | 0.00002 | 0.00002 |
| 1.680 | 0.00057 | 0.00129 | 0.00132 | 0.00011 | 0.00145 | 0.00434 | 0.00002 | 0.00002 | 0.00002 |
| 1.690 | 0.00061 | 0.00135 | 0.00131 | 0.00010 | 0.00147 | 0.00447 | 0.00002 | 0.00002 | 0.00002 |
| 1.690 | 0.00059 | 0.00141 | 0.00132 | 0.00011 | 0.00149 | 0.00461 | 0.00002 | 0.00002 | 0.00002 |
| 1.700 | 0.00061 | 0.00150 | 0.00132 | 0.00011 | 0.00149 | 0.00475 | 0.00002 | 0.00002 | 0.00002 |
| 1.710 | 0.00064 | 0.00155 | 0.00134 | 0.00013 | 0.00153 | 0.00490 | 0.00002 | 0.00002 | 0.00002 |
| 1.720 | 0.00064 | 0.00156 | 0.00134 | 0.00012 | 0.00151 | 0.00499 | 0.00002 | 0.00002 | 0.00002 |

**Table. 19** In the non-submerged state (*h*=0.05 m), the Turbulent kinetic energy *TKE* of four different fragmentation conditions changes along the *x* direction.

| **Hydraulic parameters** | **Turbulent kinetic energy *TKE* (m^2^/s^2^)** | | | | | | | | | | | |
| --- | --- | --- | --- | --- | --- | --- | --- | --- | --- | --- | --- | --- |
| ***x* (m)** | **L_1-1_** | | | | **L_3-3_** | | | | **L_4-4_** | | | |
|  | **Fragmentation I** | **Fragmentation II** | **Fragmentation III** | **Fragmentation IV** | **Fragmentation I** | **Fragmentation II** | **Fragmentation III** | **Fragmentation IV** | **Fragmentation I** | **Fragmentation II** | **Fragmentation III** | **Fragmentation IV** |
| 0.000 | 0.00034 | 0.00034 | 0.00034 | 0.00034 | 0.00034 | 0.00034 | 0.00034 | 0.00034 | 0.00034 | 0.00034 | 0.00034 | 0.00034 |
| 0.009 | 0.00031 | 0.00031 | 0.00031 | 0.00031 | 0.00031 | 0.00031 | 0.00031 | 0.00031 | 0.00031 | 0.00031 | 0.00031 | 0.00031 |
| 0.017 | 0.00029 | 0.00029 | 0.00029 | 0.00029 | 0.00029 | 0.00029 | 0.00029 | 0.00029 | 0.00029 | 0.00029 | 0.00029 | 0.00029 |
| 0.026 | 0.00027 | 0.00027 | 0.00027 | 0.00027 | 0.00027 | 0.00027 | 0.00027 | 0.00027 | 0.00027 | 0.00027 | 0.00027 | 0.00027 |
| 0.035 | 0.00025 | 0.00025 | 0.00025 | 0.00025 | 0.00025 | 0.00025 | 0.00025 | 0.00025 | 0.00025 | 0.00025 | 0.00025 | 0.00025 |
| 0.043 | 0.00023 | 0.00023 | 0.00023 | 0.00024 | 0.00023 | 0.00023 | 0.00023 | 0.00024 | 0.00023 | 0.00023 | 0.00023 | 0.00024 |
| 0.052 | 0.00022 | 0.00022 | 0.00022 | 0.00022 | 0.00022 | 0.00022 | 0.00022 | 0.00022 | 0.00022 | 0.00022 | 0.00022 | 0.00022 |
| 0.061 | 0.00021 | 0.00021 | 0.00021 | 0.00021 | 0.00021 | 0.00021 | 0.00021 | 0.00021 | 0.00021 | 0.00021 | 0.00021 | 0.00021 |
| 0.069 | 0.00020 | 0.00020 | 0.00020 | 0.00020 | 0.00020 | 0.00020 | 0.00020 | 0.00020 | 0.00020 | 0.00020 | 0.00020 | 0.00020 |
| 0.078 | 0.00019 | 0.00019 | 0.00019 | 0.00019 | 0.00019 | 0.00019 | 0.00019 | 0.00019 | 0.00019 | 0.00019 | 0.00019 | 0.00019 |
| 0.086 | 0.00018 | 0.00018 | 0.00018 | 0.00018 | 0.00018 | 0.00018 | 0.00018 | 0.00018 | 0.00018 | 0.00018 | 0.00018 | 0.00018 |
| 0.095 | 0.00017 | 0.00017 | 0.00017 | 0.00017 | 0.00017 | 0.00017 | 0.00017 | 0.00017 | 0.00017 | 0.00017 | 0.00017 | 0.00017 |
| 0.104 | 0.00016 | 0.00016 | 0.00016 | 0.00016 | 0.00016 | 0.00016 | 0.00016 | 0.00016 | 0.00016 | 0.00016 | 0.00016 | 0.00016 |
| 0.112 | 0.00016 | 0.00016 | 0.00016 | 0.00016 | 0.00016 | 0.00016 | 0.00016 | 0.00016 | 0.00016 | 0.00016 | 0.00016 | 0.00016 |
| 0.121 | 0.00015 | 0.00015 | 0.00015 | 0.00015 | 0.00015 | 0.00015 | 0.00015 | 0.00015 | 0.00015 | 0.00015 | 0.00015 | 0.00015 |
| 0.130 | 0.00014 | 0.00014 | 0.00014 | 0.00014 | 0.00014 | 0.00014 | 0.00014 | 0.00014 | 0.00014 | 0.00014 | 0.00014 | 0.00014 |
| 0.138 | 0.00014 | 0.00014 | 0.00014 | 0.00014 | 0.00014 | 0.00014 | 0.00014 | 0.00014 | 0.00014 | 0.00014 | 0.00014 | 0.00014 |
| 0.147 | 0.00013 | 0.00013 | 0.00013 | 0.00013 | 0.00013 | 0.00013 | 0.00013 | 0.00013 | 0.00013 | 0.00013 | 0.00013 | 0.00013 |
| 0.156 | 0.00013 | 0.00013 | 0.00013 | 0.00013 | 0.00013 | 0.00013 | 0.00013 | 0.00013 | 0.00013 | 0.00013 | 0.00013 | 0.00013 |
| 0.164 | 0.00012 | 0.00012 | 0.00012 | 0.00012 | 0.00012 | 0.00012 | 0.00012 | 0.00012 | 0.00013 | 0.00013 | 0.00013 | 0.00013 |
| 0.173 | 0.00012 | 0.00012 | 0.00012 | 0.00012 | 0.00012 | 0.00012 | 0.00012 | 0.00012 | 0.00012 | 0.00012 | 0.00012 | 0.00012 |
| 0.182 | 0.00012 | 0.00012 | 0.00012 | 0.00012 | 0.00012 | 0.00012 | 0.00012 | 0.00012 | 0.00012 | 0.00012 | 0.00012 | 0.00012 |
| 0.190 | 0.00011 | 0.00011 | 0.00011 | 0.00011 | 0.00011 | 0.00011 | 0.00011 | 0.00011 | 0.00011 | 0.00011 | 0.00011 | 0.00011 |
| 0.199 | 0.00011 | 0.00011 | 0.00011 | 0.00011 | 0.00011 | 0.00011 | 0.00011 | 0.00011 | 0.00011 | 0.00011 | 0.00011 | 0.00011 |
| 0.207 | 0.00010 | 0.00010 | 0.00011 | 0.00011 | 0.00011 | 0.00011 | 0.00011 | 0.00011 | 0.00011 | 0.00011 | 0.00011 | 0.00011 |
| 0.216 | 0.00010 | 0.00010 | 0.00010 | 0.00010 | 0.00010 | 0.00010 | 0.00010 | 0.00010 | 0.00011 | 0.00011 | 0.00011 | 0.00010 |
| 0.225 | 0.00010 | 0.00010 | 0.00010 | 0.00010 | 0.00010 | 0.00010 | 0.00010 | 0.00010 | 0.00010 | 0.00010 | 0.00010 | 0.00010 |
| 0.233 | 0.00010 | 0.00010 | 0.00010 | 0.00010 | 0.00010 | 0.00010 | 0.00010 | 0.00010 | 0.00010 | 0.00010 | 0.00010 | 0.00010 |
| 0.242 | 0.00009 | 0.00009 | 0.00009 | 0.00009 | 0.00010 | 0.00010 | 0.00010 | 0.00010 | 0.00010 | 0.00010 | 0.00010 | 0.00010 |
| 0.251 | 0.00009 | 0.00009 | 0.00009 | 0.00009 | 0.00009 | 0.00009 | 0.00009 | 0.00009 | 0.00010 | 0.00010 | 0.00009 | 0.00009 |
| 0.259 | 0.00009 | 0.00009 | 0.00009 | 0.00009 | 0.00009 | 0.00009 | 0.00009 | 0.00009 | 0.00009 | 0.00009 | 0.00009 | 0.00009 |
| 0.268 | 0.00009 | 0.00009 | 0.00009 | 0.00009 | 0.00009 | 0.00009 | 0.00009 | 0.00009 | 0.00009 | 0.00009 | 0.00009 | 0.00009 |
| 0.277 | 0.00008 | 0.00008 | 0.00008 | 0.00008 | 0.00009 | 0.00009 | 0.00009 | 0.00009 | 0.00009 | 0.00009 | 0.00009 | 0.00009 |
| 0.285 | 0.00038 | 0.00008 | 0.00008 | 0.00008 | 0.00010 | 0.00009 | 0.00009 | 0.00008 | 0.00009 | 0.00009 | 0.00009 | 0.00009 |
| 0.294 | 0.00114 | 0.00024 | 0.00008 | 0.00008 | 0.00090 | 0.00009 | 0.00008 | 0.00008 | 0.00009 | 0.00009 | 0.00009 | 0.00008 |
| 0.303 | 0.00512 | 0.00100 | 0.00008 | 0.00008 | 0.00200 | 0.00010 | 0.00008 | 0.00008 | 0.00009 | 0.00008 | 0.00008 | 0.00008 |
| 0.311 | 0.00572 | 0.00200 | 0.00008 | 0.00008 | 0.00285 | 0.00022 | 0.00008 | 0.00008 | 0.00008 | 0.00008 | 0.00008 | 0.00008 |
| 0.320 | 0.00616 | 0.00125 | 0.00047 | 0.00008 | 0.00246 | 0.00090 | 0.00008 | 0.00008 | 0.00008 | 0.00008 | 0.00008 | 0.00008 |
| 0.328 | 0.00422 | 0.00396 | 0.00112 | 0.00007 | 0.00167 | 0.00186 | 0.00008 | 0.00008 | 0.00008 | 0.00008 | 0.00008 | 0.00008 |
| 0.337 | 0.00630 | 0.00328 | 0.00155 | 0.00007 | 0.00275 | 0.00250 | 0.00009 | 0.00008 | 0.00008 | 0.00008 | 0.00008 | 0.00008 |
| 0.346 | 0.00630 | 0.00241 | 0.00235 | 0.00007 | 0.00299 | 0.00280 | 0.00011 | 0.00008 | 0.00008 | 0.00008 | 0.00008 | 0.00008 |
| 0.354 | 0.00486 | 0.00177 | 0.00223 | 0.00007 | 0.00301 | 0.00320 | 0.00021 | 0.00008 | 0.00008 | 0.00008 | 0.00008 | 0.00008 |
| 0.363 | 0.00439 | 0.00146 | 0.00281 | 0.00024 | 0.00305 | 0.00300 | 0.00049 | 0.00007 | 0.00008 | 0.00008 | 0.00008 | 0.00007 |
| 0.372 | 0.00490 | 0.00159 | 0.00241 | 0.00109 | 0.00319 | 0.00229 | 0.00089 | 0.00007 | 0.00008 | 0.00007 | 0.00007 | 0.00007 |
| 0.380 | 0.00565 | 0.00144 | 0.00252 | 0.00255 | 0.00350 | 0.00192 | 0.00129 | 0.00007 | 0.00008 | 0.00007 | 0.00007 | 0.00007 |
| 0.389 | 0.00470 | 0.00190 | 0.00208 | 0.00269 | 0.00350 | 0.00182 | 0.00163 | 0.00007 | 0.00007 | 0.00007 | 0.00007 | 0.00007 |
| 0.398 | 0.00502 | 0.00264 | 0.00155 | 0.00230 | 0.00331 | 0.00167 | 0.00196 | 0.00008 | 0.00007 | 0.00007 | 0.00007 | 0.00007 |
| 0.406 | 0.00312 | 0.00329 | 0.00118 | 0.00404 | 0.00295 | 0.00161 | 0.00229 | 0.00008 | 0.00007 | 0.00007 | 0.00007 | 0.00007 |
| 0.415 | 0.00585 | 0.00289 | 0.00095 | 0.00310 | 0.00271 | 0.00187 | 0.00259 | 0.00010 | 0.00007 | 0.00007 | 0.00007 | 0.00007 |
| 0.424 | 0.00585 | 0.00260 | 0.00079 | 0.00333 | 0.00265 | 0.00220 | 0.00276 | 0.00014 | 0.00007 | 0.00007 | 0.00007 | 0.00007 |
| 0.432 | 0.00527 | 0.00220 | 0.00067 | 0.00267 | 0.00252 | 0.00227 | 0.00281 | 0.00021 | 0.00007 | 0.00007 | 0.00007 | 0.00007 |
| 0.441 | 0.00323 | 0.00206 | 0.00058 | 0.00223 | 0.00240 | 0.00228 | 0.00273 | 0.00031 | 0.00007 | 0.00007 | 0.00007 | 0.00007 |
| 0.449 | 0.00549 | 0.00209 | 0.00050 | 0.00254 | 0.00227 | 0.00242 | 0.00248 | 0.00040 | 0.00007 | 0.00007 | 0.00007 | 0.00007 |
| 0.458 | 0.00546 | 0.00290 | 0.00045 | 0.00182 | 0.00230 | 0.00243 | 0.00208 | 0.00053 | 0.00007 | 0.00007 | 0.00007 | 0.00007 |
| 0.467 | 0.00448 | 0.00149 | 0.00059 | 0.00222 | 0.00235 | 0.00225 | 0.00164 | 0.00067 | 0.00007 | 0.00007 | 0.00007 | 0.00006 |
| 0.475 | 0.00385 | 0.00325 | 0.00113 | 0.00137 | 0.00232 | 0.00204 | 0.00128 | 0.00078 | 0.00007 | 0.00007 | 0.00006 | 0.00006 |
| 0.484 | 0.00545 | 0.00358 | 0.00212 | 0.00182 | 0.00215 | 0.00199 | 0.00108 | 0.00089 | 0.00007 | 0.00006 | 0.00006 | 0.00006 |
| 0.493 | 0.00623 | 0.00314 | 0.00180 | 0.00149 | 0.00232 | 0.00213 | 0.00096 | 0.00101 | 0.00007 | 0.00006 | 0.00006 | 0.00006 |
| 0.501 | 0.00530 | 0.00300 | 0.00143 | 0.00106 | 0.00246 | 0.00242 | 0.00088 | 0.00111 | 0.00007 | 0.00006 | 0.00006 | 0.00006 |
| 0.510 | 0.00513 | 0.00301 | 0.00143 | 0.00080 | 0.00253 | 0.00218 | 0.00083 | 0.00121 | 0.00007 | 0.00006 | 0.00006 | 0.00006 |
| 0.519 | 0.00369 | 0.00346 | 0.00100 | 0.00065 | 0.00227 | 0.00197 | 0.00080 | 0.00130 | 0.00007 | 0.00006 | 0.00006 | 0.00006 |
| 0.527 | 0.00509 | 0.00206 | 0.00099 | 0.00055 | 0.00201 | 0.00186 | 0.00079 | 0.00138 | 0.00006 | 0.00006 | 0.00006 | 0.00006 |
| 0.536 | 0.00489 | 0.00265 | 0.00103 | 0.00046 | 0.00201 | 0.00179 | 0.00079 | 0.00148 | 0.00006 | 0.00006 | 0.00006 | 0.00006 |
| 0.545 | 0.00449 | 0.00155 | 0.00083 | 0.00041 | 0.00196 | 0.00165 | 0.00079 | 0.00159 | 0.00006 | 0.00006 | 0.00006 | 0.00006 |
| 0.553 | 0.00384 | 0.00457 | 0.00064 | 0.00038 | 0.00187 | 0.00153 | 0.00080 | 0.00170 | 0.00006 | 0.00006 | 0.00006 | 0.00006 |
| 0.562 | 0.00463 | 0.00438 | 0.00053 | 0.00035 | 0.00173 | 0.00148 | 0.00082 | 0.00181 | 0.00006 | 0.00006 | 0.00006 | 0.00006 |
| 0.570 | 0.00428 | 0.00404 | 0.00047 | 0.00032 | 0.00171 | 0.00149 | 0.00085 | 0.00192 | 0.00006 | 0.00006 | 0.00006 | 0.00006 |
| 0.579 | 0.00390 | 0.00381 | 0.00044 | 0.00030 | 0.00170 | 0.00152 | 0.00089 | 0.00204 | 0.00006 | 0.00006 | 0.00006 | 0.00006 |
| 0.588 | 0.00335 | 0.00349 | 0.00049 | 0.00028 | 0.00174 | 0.00141 | 0.00094 | 0.00217 | 0.00006 | 0.00006 | 0.00006 | 0.00006 |
| 0.596 | 0.00295 | 0.00204 | 0.00065 | 0.00026 | 0.00150 | 0.00126 | 0.00099 | 0.00233 | 0.00006 | 0.00006 | 0.00006 | 0.00006 |
| 0.605 | 0.00364 | 0.00144 | 0.00082 | 0.00025 | 0.00148 | 0.00115 | 0.00105 | 0.00251 | 0.00006 | 0.00006 | 0.00006 | 0.00006 |
| 0.614 | 0.00311 | 0.00256 | 0.00142 | 0.00024 | 0.00152 | 0.00106 | 0.00112 | 0.00272 | 0.00006 | 0.00006 | 0.00006 | 0.00006 |
| 0.622 | 0.00329 | 0.00206 | 0.00115 | 0.00024 | 0.00154 | 0.00105 | 0.00123 | 0.00295 | 0.00006 | 0.00006 | 0.00006 | 0.00005 |
| 0.631 | 0.00260 | 0.00378 | 0.00137 | 0.00024 | 0.00148 | 0.00108 | 0.00131 | 0.00319 | 0.00006 | 0.00006 | 0.00006 | 0.00005 |
| 0.640 | 0.00369 | 0.00344 | 0.00099 | 0.00026 | 0.00138 | 0.00120 | 0.00131 | 0.00340 | 0.00006 | 0.00006 | 0.00005 | 0.00005 |
| 0.648 | 0.00326 | 0.00343 | 0.00117 | 0.00028 | 0.00153 | 0.00139 | 0.00126 | 0.00355 | 0.00006 | 0.00006 | 0.00005 | 0.00005 |
| 0.657 | 0.00286 | 0.00329 | 0.00074 | 0.00031 | 0.00173 | 0.00149 | 0.00120 | 0.00366 | 0.00006 | 0.00006 | 0.00005 | 0.00005 |
| 0.666 | 0.00203 | 0.00346 | 0.00056 | 0.00038 | 0.00171 | 0.00140 | 0.00114 | 0.00372 | 0.00006 | 0.00005 | 0.00005 | 0.00005 |
| 0.674 | 0.00311 | 0.00272 | 0.00060 | 0.00076 | 0.00162 | 0.00121 | 0.00109 | 0.00376 | 0.00006 | 0.00005 | 0.00005 | 0.00005 |
| 0.683 | 0.00314 | 0.00361 | 0.00062 | 0.00063 | 0.00175 | 0.00111 | 0.00105 | 0.00377 | 0.00006 | 0.00005 | 0.00005 | 0.00005 |
| 0.691 | 0.00279 | 0.00185 | 0.00045 | 0.00082 | 0.00177 | 0.00114 | 0.00102 | 0.00374 | 0.00006 | 0.00005 | 0.00005 | 0.00005 |
| 0.700 | 0.00218 | 0.00314 | 0.00033 | 0.00055 | 0.00164 | 0.00118 | 0.00101 | 0.00366 | 0.00006 | 0.00005 | 0.00005 | 0.00005 |
| 0.709 | 0.00263 | 0.00312 | 0.00031 | 0.00070 | 0.00143 | 0.00122 | 0.00102 | 0.00361 | 0.00006 | 0.00005 | 0.00005 | 0.00005 |
| 0.717 | 0.00328 | 0.00307 | 0.00046 | 0.00054 | 0.00144 | 0.00139 | 0.00103 | 0.00359 | 0.00006 | 0.00005 | 0.00005 | 0.00005 |
| 0.726 | 0.00277 | 0.00299 | 0.00084 | 0.00043 | 0.00156 | 0.00157 | 0.00106 | 0.00349 | 0.00006 | 0.00005 | 0.00005 | 0.00005 |
| 0.735 | 0.00300 | 0.00277 | 0.00135 | 0.00054 | 0.00162 | 0.00164 | 0.00110 | 0.00342 | 0.00006 | 0.00005 | 0.00005 | 0.00005 |
| 0.743 | 0.00206 | 0.00324 | 0.00183 | 0.00040 | 0.00148 | 0.00161 | 0.00116 | 0.00337 | 0.00005 | 0.00005 | 0.00005 | 0.00005 |
| 0.752 | 0.00299 | 0.00165 | 0.00223 | 0.00055 | 0.00133 | 0.00142 | 0.00123 | 0.00330 | 0.00005 | 0.00005 | 0.00005 | 0.00005 |
| 0.761 | 0.00290 | 0.00271 | 0.00261 | 0.00031 | 0.00137 | 0.00120 | 0.00132 | 0.00327 | 0.00005 | 0.00005 | 0.00005 | 0.00005 |
| 0.769 | 0.00290 | 0.00136 | 0.00227 | 0.00029 | 0.00141 | 0.00108 | 0.00138 | 0.00326 | 0.00005 | 0.00005 | 0.00005 | 0.00005 |
| 0.778 | 0.00182 | 0.00312 | 0.00102 | 0.00025 | 0.00141 | 0.00102 | 0.00141 | 0.00324 | 0.00005 | 0.00005 | 0.00005 | 0.00005 |
| 0.787 | 0.00279 | 0.00287 | 0.00086 | 0.00026 | 0.00137 | 0.00101 | 0.00143 | 0.00327 | 0.00005 | 0.00005 | 0.00005 | 0.00005 |
| 0.795 | 0.00294 | 0.00267 | 0.00101 | 0.00018 | 0.00147 | 0.00149 | 0.00146 | 0.00328 | 0.00005 | 0.00005 | 0.00005 | 0.00005 |
| 0.804 | 0.00262 | 0.00252 | 0.00093 | 0.00011 | 0.00161 | 0.00161 | 0.00150 | 0.00330 | 0.00005 | 0.00005 | 0.00005 | 0.00005 |
| 0.812 | 0.00232 | 0.00231 | 0.00077 | 0.00007 | 0.00160 | 0.00170 | 0.00153 | 0.00335 | 0.00005 | 0.00005 | 0.00005 | 0.00005 |
| 0.821 | 0.00238 | 0.00167 | 0.00063 | 0.00005 | 0.00145 | 0.00162 | 0.00156 | 0.00340 | 0.00005 | 0.00005 | 0.00005 | 0.00005 |
| 0.830 | 0.00243 | 0.00133 | 0.00054 | 0.00004 | 0.00138 | 0.00149 | 0.00158 | 0.00346 | 0.00005 | 0.00005 | 0.00005 | 0.00005 |
| 0.838 | 0.00233 | 0.00180 | 0.00046 | 0.00003 | 0.00121 | 0.00140 | 0.00161 | 0.00353 | 0.00005 | 0.00005 | 0.00005 | 0.00004 |
| 0.847 | 0.00242 | 0.00170 | 0.00037 | 0.00003 | 0.00121 | 0.00133 | 0.00164 | 0.00360 | 0.00005 | 0.00005 | 0.00005 | 0.00004 |
| 0.856 | 0.00145 | 0.00257 | 0.00030 | 0.00003 | 0.00109 | 0.00127 | 0.00168 | 0.00368 | 0.00005 | 0.00005 | 0.00005 | 0.00004 |
| 0.864 | 0.00225 | 0.00251 | 0.00025 | 0.00003 | 0.00109 | 0.00149 | 0.00171 | 0.00377 | 0.00005 | 0.00005 | 0.00005 | 0.00004 |
| 0.873 | 0.00217 | 0.00223 | 0.00040 | 0.00005 | 0.00120 | 0.00158 | 0.00175 | 0.00387 | 0.00005 | 0.00005 | 0.00004 | 0.00004 |
| 0.882 | 0.00226 | 0.00195 | 0.00093 | 0.00007 | 0.00129 | 0.00165 | 0.00180 | 0.00398 | 0.00005 | 0.00005 | 0.00004 | 0.00004 |
| 0.890 | 0.00168 | 0.00165 | 0.00155 | 0.00010 | 0.00119 | 0.00168 | 0.00185 | 0.00410 | 0.00005 | 0.00005 | 0.00004 | 0.00004 |
| 0.899 | 0.00228 | 0.00121 | 0.00202 | 0.00016 | 0.00111 | 0.00166 | 0.00189 | 0.00423 | 0.00005 | 0.00005 | 0.00004 | 0.00004 |
| 0.908 | 0.00230 | 0.00211 | 0.00239 | 0.00023 | 0.00118 | 0.00158 | 0.00191 | 0.00438 | 0.00005 | 0.00005 | 0.00004 | 0.00004 |
| 0.916 | 0.00188 | 0.00119 | 0.00321 | 0.00033 | 0.00123 | 0.00150 | 0.00195 | 0.00454 | 0.00005 | 0.00005 | 0.00004 | 0.00004 |
| 0.925 | 0.00164 | 0.00184 | 0.00141 | 0.00050 | 0.00116 | 0.00143 | 0.00208 | 0.00473 | 0.00005 | 0.00005 | 0.00004 | 0.00004 |
| 0.933 | 0.00149 | 0.00185 | 0.00124 | 0.00079 | 0.00103 | 0.00139 | 0.00227 | 0.00493 | 0.00005 | 0.00005 | 0.00004 | 0.00004 |
| 0.942 | 0.00187 | 0.00164 | 0.00095 | 0.00123 | 0.00110 | 0.00140 | 0.00240 | 0.00515 | 0.00005 | 0.00004 | 0.00004 | 0.00004 |
| 0.951 | 0.00174 | 0.00149 | 0.00112 | 0.00159 | 0.00123 | 0.00145 | 0.00241 | 0.00537 | 0.00005 | 0.00004 | 0.00004 | 0.00004 |
| 0.959 | 0.00174 | 0.00142 | 0.00100 | 0.00137 | 0.00121 | 0.00150 | 0.00233 | 0.00559 | 0.00005 | 0.00004 | 0.00004 | 0.00004 |
| 0.968 | 0.00108 | 0.00144 | 0.00073 | 0.00013 | 0.00103 | 0.00150 | 0.00219 | 0.00580 | 0.00005 | 0.00004 | 0.00004 | 0.00004 |
| 0.977 | 0.00184 | 0.00073 | 0.00067 | 0.00054 | 0.00099 | 0.00145 | 0.00205 | 0.00599 | 0.00005 | 0.00004 | 0.00004 | 0.00004 |
| 0.985 | 0.00177 | 0.00156 | 0.00064 | 0.00010 | 0.00107 | 0.00138 | 0.00199 | 0.00614 | 0.00005 | 0.00004 | 0.00004 | 0.00004 |
| 0.994 | 0.00173 | 0.00041 | 0.00044 | 0.00043 | 0.00116 | 0.00136 | 0.00204 | 0.00627 | 0.00005 | 0.00004 | 0.00004 | 0.00004 |
| 1.000 | 0.00110 | 0.00150 | 0.00034 | 0.00030 | 0.00114 | 0.00135 | 0.00218 | 0.00639 | 0.00005 | 0.00004 | 0.00004 | 0.00004 |
| 1.010 | 0.00218 | 0.00147 | 0.00033 | 0.00036 | 0.00112 | 0.00137 | 0.00237 | 0.00650 | 0.00005 | 0.00004 | 0.00004 | 0.00004 |
| 1.020 | 0.00212 | 0.00131 | 0.00047 | 0.00034 | 0.00119 | 0.00145 | 0.00256 | 0.00658 | 0.00005 | 0.00004 | 0.00004 | 0.00004 |
| 1.030 | 0.00193 | 0.00120 | 0.00083 | 0.00029 | 0.00125 | 0.00154 | 0.00272 | 0.00664 | 0.00005 | 0.00004 | 0.00004 | 0.00004 |
| 1.040 | 0.00160 | 0.00107 | 0.00125 | 0.00030 | 0.00117 | 0.00158 | 0.00283 | 0.00667 | 0.00005 | 0.00004 | 0.00004 | 0.00004 |
| 1.050 | 0.00140 | 0.00078 | 0.00164 | 0.00019 | 0.00109 | 0.00158 | 0.00289 | 0.00671 | 0.00005 | 0.00004 | 0.00004 | 0.00004 |
| 1.050 | 0.00180 | 0.00049 | 0.00198 | 0.00031 | 0.00115 | 0.00156 | 0.00292 | 0.00674 | 0.00005 | 0.00004 | 0.00004 | 0.00004 |
| 1.060 | 0.00158 | 0.00111 | 0.00242 | 0.00023 | 0.00122 | 0.00151 | 0.00309 | 0.00674 | 0.00005 | 0.00004 | 0.00004 | 0.00004 |
| 1.070 | 0.00167 | 0.00069 | 0.00120 | 0.00022 | 0.00121 | 0.00147 | 0.00337 | 0.00678 | 0.00005 | 0.00004 | 0.00004 | 0.00004 |
| 1.080 | 0.00121 | 0.00133 | 0.00120 | 0.00015 | 0.00109 | 0.00147 | 0.00358 | 0.00682 | 0.00005 | 0.00004 | 0.00004 | 0.00004 |
| 1.090 | 0.00198 | 0.00122 | 0.00124 | 0.00013 | 0.00110 | 0.00150 | 0.00360 | 0.00687 | 0.00005 | 0.00004 | 0.00004 | 0.00004 |
| 1.100 | 0.00181 | 0.00107 | 0.00138 | 0.00010 | 0.00125 | 0.00155 | 0.00349 | 0.00693 | 0.00005 | 0.00004 | 0.00004 | 0.00004 |
| 1.110 | 0.00189 | 0.00093 | 0.00067 | 0.00015 | 0.00135 | 0.00162 | 0.00331 | 0.00700 | 0.00004 | 0.00004 | 0.00004 | 0.00004 |
| 1.110 | 0.00115 | 0.00092 | 0.00087 | 0.00030 | 0.00125 | 0.00165 | 0.00315 | 0.00708 | 0.00004 | 0.00004 | 0.00004 | 0.00004 |
| 1.120 | 0.00137 | 0.00074 | 0.00060 | 0.00047 | 0.00116 | 0.00162 | 0.00312 | 0.00716 | 0.00004 | 0.00004 | 0.00004 | 0.00004 |
| 1.130 | 0.00146 | 0.00131 | 0.00057 | 0.00061 | 0.00124 | 0.00154 | 0.00321 | 0.00724 | 0.00004 | 0.00004 | 0.00004 | 0.00004 |
| 1.140 | 0.00135 | 0.00046 | 0.00053 | 0.00074 | 0.00131 | 0.00149 | 0.00336 | 0.00733 | 0.00004 | 0.00004 | 0.00004 | 0.00004 |
| 1.150 | 0.00133 | 0.00119 | 0.00041 | 0.00087 | 0.00127 | 0.00149 | 0.00351 | 0.00743 | 0.00004 | 0.00004 | 0.00004 | 0.00004 |
| 1.160 | 0.00111 | 0.00136 | 0.00039 | 0.00102 | 0.00114 | 0.00154 | 0.00364 | 0.00752 | 0.00004 | 0.00004 | 0.00004 | 0.00004 |
| 1.170 | 0.00141 | 0.00112 | 0.00063 | 0.00117 | 0.00122 | 0.00163 | 0.00375 | 0.00761 | 0.00004 | 0.00004 | 0.00004 | 0.00004 |
| 1.180 | 0.00135 | 0.00093 | 0.00117 | 0.00132 | 0.00130 | 0.00173 | 0.00383 | 0.00770 | 0.00004 | 0.00004 | 0.00004 | 0.00003 |
| 1.180 | 0.00143 | 0.00080 | 0.00179 | 0.00146 | 0.00123 | 0.00180 | 0.00400 | 0.00779 | 0.00005 | 0.00004 | 0.00004 | 0.00003 |
| 1.190 | 0.00118 | 0.00085 | 0.00235 | 0.00159 | 0.00113 | 0.00179 | 0.00431 | 0.00788 | 0.00005 | 0.00004 | 0.00004 | 0.00003 |
| 1.200 | 0.00154 | 0.00055 | 0.00274 | 0.00170 | 0.00117 | 0.00169 | 0.00464 | 0.00797 | 0.00005 | 0.00004 | 0.00004 | 0.00003 |
| 1.210 | 0.00142 | 0.00116 | 0.00330 | 0.00179 | 0.00132 | 0.00158 | 0.00477 | 0.00807 | 0.00005 | 0.00004 | 0.00004 | 0.00003 |
| 1.220 | 0.00130 | 0.00031 | 0.00373 | 0.00184 | 0.00136 | 0.00155 | 0.00469 | 0.00816 | 0.00005 | 0.00004 | 0.00004 | 0.00003 |
| 1.230 | 0.00086 | 0.00108 | 0.00243 | 0.00187 | 0.00120 | 0.00155 | 0.00462 | 0.00826 | 0.00005 | 0.00004 | 0.00004 | 0.00003 |
| 1.240 | 0.00110 | 0.00109 | 0.00174 | 0.00198 | 0.00119 | 0.00162 | 0.00463 | 0.00834 | 0.00005 | 0.00004 | 0.00004 | 0.00003 |
| 1.240 | 0.00124 | 0.00091 | 0.00183 | 0.00215 | 0.00133 | 0.00175 | 0.00463 | 0.00842 | 0.00005 | 0.00004 | 0.00004 | 0.00003 |
| 1.250 | 0.00112 | 0.00073 | 0.00103 | 0.00226 | 0.00139 | 0.00190 | 0.00454 | 0.00847 | 0.00005 | 0.00004 | 0.00003 | 0.00003 |
| 1.260 | 0.00129 | 0.00058 | 0.00161 | 0.00141 | 0.00126 | 0.00202 | 0.00439 | 0.00852 | 0.00005 | 0.00004 | 0.00003 | 0.00003 |
| 1.270 | 0.00129 | 0.00062 | 0.00079 | 0.00018 | 0.00116 | 0.00204 | 0.00422 | 0.00854 | 0.00005 | 0.00004 | 0.00003 | 0.00003 |
| 1.280 | 0.00145 | 0.00041 | 0.00071 | 0.00047 | 0.00128 | 0.00189 | 0.00417 | 0.00854 | 0.00005 | 0.00004 | 0.00003 | 0.00003 |
| 1.290 | 0.00130 | 0.00079 | 0.00064 | 0.00034 | 0.00151 | 0.00174 | 0.00425 | 0.00852 | 0.00005 | 0.00004 | 0.00003 | 0.00003 |
| 1.300 | 0.00111 | 0.00051 | 0.00050 | 0.00058 | 0.00154 | 0.00169 | 0.00441 | 0.00849 | 0.00006 | 0.00004 | 0.00003 | 0.00003 |
| 1.310 | 0.00078 | 0.00106 | 0.00040 | 0.00044 | 0.00141 | 0.00171 | 0.00460 | 0.00843 | 0.00006 | 0.00004 | 0.00003 | 0.00003 |
| 1.310 | 0.00112 | 0.00107 | 0.00034 | 0.00036 | 0.00152 | 0.00186 | 0.00478 | 0.00836 | 0.00006 | 0.00004 | 0.00003 | 0.00003 |
| 1.320 | 0.00113 | 0.00088 | 0.00033 | 0.00048 | 0.00171 | 0.00212 | 0.00494 | 0.00829 | 0.00006 | 0.00004 | 0.00003 | 0.00003 |
| 1.330 | 0.00105 | 0.00069 | 0.00042 | 0.00028 | 0.00165 | 0.00240 | 0.00510 | 0.00820 | 0.00006 | 0.00005 | 0.00003 | 0.00003 |
| 1.340 | 0.00074 | 0.00069 | 0.00070 | 0.00038 | 0.00143 | 0.00253 | 0.00530 | 0.00809 | 0.00007 | 0.00005 | 0.00003 | 0.00003 |
| 1.350 | 0.00107 | 0.00069 | 0.00105 | 0.00033 | 0.00133 | 0.00242 | 0.00555 | 0.00797 | 0.00007 | 0.00005 | 0.00003 | 0.00003 |
| 1.360 | 0.00124 | 0.00086 | 0.00144 | 0.00030 | 0.00157 | 0.00221 | 0.00575 | 0.00786 | 0.00007 | 0.00005 | 0.00003 | 0.00003 |
| 1.370 | 0.00119 | 0.00063 | 0.00195 | 0.00028 | 0.00175 | 0.00219 | 0.00582 | 0.00777 | 0.00008 | 0.00005 | 0.00003 | 0.00003 |
| 1.370 | 0.00108 | 0.00086 | 0.00091 | 0.00033 | 0.00154 | 0.00221 | 0.00581 | 0.00768 | 0.00008 | 0.00005 | 0.00003 | 0.00003 |
| 1.380 | 0.00102 | 0.00102 | 0.00213 | 0.00021 | 0.00147 | 0.00241 | 0.00584 | 0.00757 | 0.00008 | 0.00006 | 0.00003 | 0.00003 |
| 1.390 | 0.00125 | 0.00080 | 0.00103 | 0.00015 | 0.00167 | 0.00275 | 0.00587 | 0.00748 | 0.00009 | 0.00006 | 0.00003 | 0.00003 |
| 1.400 | 0.00119 | 0.00065 | 0.00109 | 0.00009 | 0.00186 | 0.00304 | 0.00585 | 0.00741 | 0.00009 | 0.00006 | 0.00003 | 0.00003 |
| 1.410 | 0.00111 | 0.00054 | 0.00090 | 0.00008 | 0.00162 | 0.00312 | 0.00576 | 0.00731 | 0.00010 | 0.00006 | 0.00003 | 0.00003 |
| 1.420 | 0.00075 | 0.00060 | 0.00057 | 0.00017 | 0.00137 | 0.00296 | 0.00563 | 0.00725 | 0.00010 | 0.00007 | 0.00003 | 0.00003 |
| 1.430 | 0.00103 | 0.00032 | 0.00051 | 0.00031 | 0.00160 | 0.00263 | 0.00555 | 0.00721 | 0.00011 | 0.00007 | 0.00003 | 0.00003 |
| 1.430 | 0.00098 | 0.00081 | 0.00053 | 0.00046 | 0.00197 | 0.00244 | 0.00554 | 0.00718 | 0.00011 | 0.00008 | 0.00003 | 0.00003 |
| 1.440 | 0.00103 | 0.00021 | 0.00041 | 0.00060 | 0.00198 | 0.00247 | 0.00565 | 0.00715 | 0.00012 | 0.00008 | 0.00003 | 0.00003 |
| 1.450 | 0.00082 | 0.00080 | 0.00032 | 0.00075 | 0.00158 | 0.00268 | 0.00581 | 0.00711 | 0.00013 | 0.00008 | 0.00003 | 0.00003 |
| 1.460 | 0.00085 | 0.00086 | 0.00027 | 0.00093 | 0.00171 | 0.00309 | 0.00601 | 0.00708 | 0.00013 | 0.00009 | 0.00003 | 0.00003 |
| 1.470 | 0.00090 | 0.00088 | 0.00033 | 0.00114 | 0.00227 | 0.00351 | 0.00622 | 0.00705 | 0.00014 | 0.00010 | 0.00003 | 0.00003 |
| 1.480 | 0.00079 | 0.00075 | 0.00055 | 0.00135 | 0.00262 | 0.00382 | 0.00644 | 0.00701 | 0.00015 | 0.00010 | 0.00003 | 0.00003 |
| 1.490 | 0.00068 | 0.00068 | 0.00079 | 0.00157 | 0.00269 | 0.00404 | 0.00663 | 0.00699 | 0.00015 | 0.00011 | 0.00003 | 0.00003 |
| 1.500 | 0.00057 | 0.00066 | 0.00101 | 0.00177 | 0.00265 | 0.00421 | 0.00680 | 0.00696 | 0.00016 | 0.00011 | 0.00003 | 0.00003 |
| 1.500 | 0.00048 | 0.00065 | 0.00120 | 0.00197 | 0.00262 | 0.00435 | 0.00695 | 0.00692 | 0.00017 | 0.00012 | 0.00003 | 0.00003 |
| 1.510 | 0.00042 | 0.00065 | 0.00137 | 0.00215 | 0.00263 | 0.00447 | 0.00713 | 0.00692 | 0.00018 | 0.00013 | 0.00003 | 0.00003 |
| 1.520 | 0.00036 | 0.00066 | 0.00152 | 0.00233 | 0.00269 | 0.00456 | 0.00733 | 0.00691 | 0.00018 | 0.00014 | 0.00003 | 0.00003 |
| 1.530 | 0.00032 | 0.00066 | 0.00163 | 0.00248 | 0.00278 | 0.00463 | 0.00753 | 0.00689 | 0.00019 | 0.00015 | 0.00003 | 0.00003 |
| 1.540 | 0.00029 | 0.00066 | 0.00172 | 0.00262 | 0.00290 | 0.00469 | 0.00767 | 0.00687 | 0.00020 | 0.00016 | 0.00003 | 0.00003 |
| 1.550 | 0.00026 | 0.00066 | 0.00178 | 0.00275 | 0.00303 | 0.00475 | 0.00777 | 0.00684 | 0.00021 | 0.00017 | 0.00003 | 0.00003 |
| 1.560 | 0.00024 | 0.00066 | 0.00182 | 0.00286 | 0.00317 | 0.00481 | 0.00781 | 0.00682 | 0.00022 | 0.00018 | 0.00003 | 0.00003 |
| 1.560 | 0.00022 | 0.00065 | 0.00184 | 0.00297 | 0.00331 | 0.00487 | 0.00783 | 0.00678 | 0.00023 | 0.00019 | 0.00004 | 0.00003 |
| 1.570 | 0.00020 | 0.00064 | 0.00185 | 0.00307 | 0.00345 | 0.00494 | 0.00785 | 0.00675 | 0.00024 | 0.00020 | 0.00004 | 0.00003 |
| 1.580 | 0.00019 | 0.00063 | 0.00183 | 0.00317 | 0.00360 | 0.00503 | 0.00787 | 0.00674 | 0.00025 | 0.00021 | 0.00004 | 0.00003 |
| 1.590 | 0.00018 | 0.00061 | 0.00181 | 0.00326 | 0.00375 | 0.00512 | 0.00790 | 0.00675 | 0.00026 | 0.00022 | 0.00004 | 0.00003 |
| 1.600 | 0.00017 | 0.00060 | 0.00178 | 0.00334 | 0.00390 | 0.00522 | 0.00792 | 0.00675 | 0.00027 | 0.00024 | 0.00004 | 0.00003 |
| 1.610 | 0.00016 | 0.00058 | 0.00174 | 0.00341 | 0.00405 | 0.00533 | 0.00794 | 0.00674 | 0.00028 | 0.00025 | 0.00004 | 0.00003 |
| 1.620 | 0.00015 | 0.00057 | 0.00170 | 0.00346 | 0.00418 | 0.00545 | 0.00794 | 0.00673 | 0.00029 | 0.00026 | 0.00004 | 0.00003 |
| 1.620 | 0.00014 | 0.00055 | 0.00165 | 0.00350 | 0.00432 | 0.00557 | 0.00793 | 0.00672 | 0.00031 | 0.00028 | 0.00005 | 0.00003 |
| 1.630 | 0.00013 | 0.00053 | 0.00160 | 0.00354 | 0.00445 | 0.00570 | 0.00790 | 0.00672 | 0.00032 | 0.00029 | 0.00005 | 0.00003 |
| 1.640 | 0.00013 | 0.00051 | 0.00155 | 0.00357 | 0.00458 | 0.00583 | 0.00786 | 0.00671 | 0.00033 | 0.00031 | 0.00005 | 0.00003 |
| 1.650 | 0.00012 | 0.00049 | 0.00150 | 0.00360 | 0.00472 | 0.00594 | 0.00782 | 0.00670 | 0.00034 | 0.00032 | 0.00006 | 0.00003 |
| 1.660 | 0.00011 | 0.00048 | 0.00146 | 0.00361 | 0.00485 | 0.00604 | 0.00777 | 0.00670 | 0.00036 | 0.00034 | 0.00006 | 0.00003 |
| 1.670 | 0.00011 | 0.00046 | 0.00141 | 0.00363 | 0.00497 | 0.00612 | 0.00771 | 0.00669 | 0.00037 | 0.00036 | 0.00007 | 0.00003 |
| 1.680 | 0.00011 | 0.00044 | 0.00136 | 0.00363 | 0.00508 | 0.00619 | 0.00766 | 0.00669 | 0.00039 | 0.00038 | 0.00007 | 0.00003 |
| 1.690 | 0.00010 | 0.00043 | 0.00132 | 0.00364 | 0.00518 | 0.00626 | 0.00761 | 0.00669 | 0.00040 | 0.00039 | 0.00008 | 0.00003 |
| 1.690 | 0.00010 | 0.00041 | 0.00128 | 0.00364 | 0.00528 | 0.00632 | 0.00755 | 0.00669 | 0.00042 | 0.00041 | 0.00009 | 0.00003 |
| 1.700 | 0.00009 | 0.00040 | 0.00124 | 0.00365 | 0.00537 | 0.00638 | 0.00751 | 0.00670 | 0.00044 | 0.00044 | 0.00010 | 0.00003 |
| 1.710 | 0.00009 | 0.00038 | 0.00120 | 0.00366 | 0.00547 | 0.00644 | 0.00747 | 0.00671 | 0.00046 | 0.00046 | 0.00011 | 0.00003 |
| 1.720 | 0.00009 | 0.00037 | 0.00119 | 0.00369 | 0.00556 | 0.00650 | 0.00745 | 0.00671 | 0.00048 | 0.00048 | 0.00013 | 0.00003 |

**Table. 20** In the submerged state (*h*=0.09 m), the Turbulent kinetic energy *TKE* of four different fragmentation conditions changes along the *x* direction.

| **Hydraulic parameters** | **Turbulent kinetic energy *TKE* (m^2^/s^2^)** | | | | | | | | | | | |
| --- | --- | --- | --- | --- | --- | --- | --- | --- | --- | --- | --- | --- |
| ***x* (m)** | **L_1-1_** | | | | **L_3-3_** | | | | **L_4-4_** | | | |
|  | **Fragmentation I** | **Fragmentation II** | **Fragmentation III** | **Fragmentation IV** | **Fragmentation I** | **Fragmentation II** | **Fragmentation III** | **Fragmentation IV** | **Fragmentation I** | **Fragmentation II** | **Fragmentation III** | **Fragmentation IV** |
| 0.000 | 0.00034 | 0.00033 | 0.00034 | 0.00034 | 0.00034 | 0.00033 | 0.00034 | 0.00034 | 0.00034 | 0.00033 | 0.00034 | 0.00034 |
| 0.009 | 0.00031 | 0.00031 | 0.00031 | 0.00031 | 0.00031 | 0.00031 | 0.00031 | 0.00031 | 0.00031 | 0.00031 | 0.00031 | 0.00031 |
| 0.017 | 0.00029 | 0.00029 | 0.00029 | 0.00029 | 0.00029 | 0.00029 | 0.00029 | 0.00029 | 0.00029 | 0.00029 | 0.00029 | 0.00029 |
| 0.026 | 0.00027 | 0.00027 | 0.00027 | 0.00027 | 0.00027 | 0.00027 | 0.00027 | 0.00027 | 0.00027 | 0.00027 | 0.00027 | 0.00027 |
| 0.035 | 0.00025 | 0.00025 | 0.00025 | 0.00025 | 0.00025 | 0.00025 | 0.00025 | 0.00025 | 0.00025 | 0.00025 | 0.00025 | 0.00025 |
| 0.043 | 0.00024 | 0.00024 | 0.00024 | 0.00023 | 0.00023 | 0.00024 | 0.00024 | 0.00023 | 0.00024 | 0.00024 | 0.00023 | 0.00024 |
| 0.052 | 0.00022 | 0.00022 | 0.00022 | 0.00022 | 0.00022 | 0.00022 | 0.00022 | 0.00022 | 0.00022 | 0.00022 | 0.00022 | 0.00022 |
| 0.061 | 0.00021 | 0.00021 | 0.00021 | 0.00021 | 0.00021 | 0.00021 | 0.00021 | 0.00021 | 0.00021 | 0.00021 | 0.00021 | 0.00021 |
| 0.069 | 0.00020 | 0.00020 | 0.00020 | 0.00020 | 0.00020 | 0.00020 | 0.00020 | 0.00020 | 0.00020 | 0.00020 | 0.00020 | 0.00020 |
| 0.078 | 0.00019 | 0.00019 | 0.00019 | 0.00019 | 0.00019 | 0.00019 | 0.00019 | 0.00019 | 0.00019 | 0.00019 | 0.00019 | 0.00019 |
| 0.086 | 0.00018 | 0.00018 | 0.00018 | 0.00018 | 0.00018 | 0.00018 | 0.00018 | 0.00018 | 0.00018 | 0.00018 | 0.00018 | 0.00018 |
| 0.095 | 0.00017 | 0.00017 | 0.00017 | 0.00017 | 0.00017 | 0.00017 | 0.00017 | 0.00017 | 0.00017 | 0.00017 | 0.00017 | 0.00017 |
| 0.104 | 0.00016 | 0.00016 | 0.00016 | 0.00016 | 0.00016 | 0.00016 | 0.00016 | 0.00016 | 0.00016 | 0.00016 | 0.00016 | 0.00016 |
| 0.112 | 0.00016 | 0.00016 | 0.00016 | 0.00016 | 0.00016 | 0.00016 | 0.00016 | 0.00016 | 0.00016 | 0.00016 | 0.00016 | 0.00016 |
| 0.121 | 0.00015 | 0.00015 | 0.00015 | 0.00015 | 0.00015 | 0.00015 | 0.00015 | 0.00015 | 0.00015 | 0.00015 | 0.00015 | 0.00015 |
| 0.130 | 0.00014 | 0.00014 | 0.00014 | 0.00014 | 0.00014 | 0.00014 | 0.00014 | 0.00014 | 0.00014 | 0.00014 | 0.00014 | 0.00014 |
| 0.138 | 0.00014 | 0.00014 | 0.00014 | 0.00014 | 0.00014 | 0.00014 | 0.00014 | 0.00014 | 0.00014 | 0.00014 | 0.00014 | 0.00014 |
| 0.147 | 0.00013 | 0.00013 | 0.00013 | 0.00013 | 0.00013 | 0.00013 | 0.00013 | 0.00013 | 0.00013 | 0.00013 | 0.00013 | 0.00013 |
| 0.156 | 0.00013 | 0.00013 | 0.00013 | 0.00013 | 0.00013 | 0.00013 | 0.00013 | 0.00013 | 0.00013 | 0.00013 | 0.00013 | 0.00013 |
| 0.164 | 0.00012 | 0.00012 | 0.00012 | 0.00012 | 0.00012 | 0.00012 | 0.00012 | 0.00012 | 0.00013 | 0.00012 | 0.00012 | 0.00012 |
| 0.173 | 0.00012 | 0.00012 | 0.00012 | 0.00012 | 0.00012 | 0.00012 | 0.00012 | 0.00012 | 0.00012 | 0.00012 | 0.00012 | 0.00012 |
| 0.182 | 0.00012 | 0.00012 | 0.00012 | 0.00012 | 0.00012 | 0.00012 | 0.00012 | 0.00012 | 0.00012 | 0.00012 | 0.00012 | 0.00012 |
| 0.190 | 0.00011 | 0.00011 | 0.00011 | 0.00011 | 0.00011 | 0.00011 | 0.00011 | 0.00011 | 0.00011 | 0.00011 | 0.00011 | 0.00011 |
| 0.199 | 0.00011 | 0.00011 | 0.00011 | 0.00011 | 0.00011 | 0.00011 | 0.00011 | 0.00011 | 0.00011 | 0.00011 | 0.00011 | 0.00011 |
| 0.207 | 0.00011 | 0.00010 | 0.00011 | 0.00011 | 0.00011 | 0.00011 | 0.00011 | 0.00011 | 0.00011 | 0.00011 | 0.00011 | 0.00011 |
| 0.216 | 0.00010 | 0.00010 | 0.00010 | 0.00010 | 0.00010 | 0.00010 | 0.00010 | 0.00010 | 0.00010 | 0.00010 | 0.00010 | 0.00010 |
| 0.225 | 0.00010 | 0.00010 | 0.00010 | 0.00010 | 0.00010 | 0.00010 | 0.00010 | 0.00010 | 0.00010 | 0.00010 | 0.00010 | 0.00010 |
| 0.233 | 0.00010 | 0.00010 | 0.00010 | 0.00010 | 0.00010 | 0.00010 | 0.00010 | 0.00010 | 0.00010 | 0.00010 | 0.00010 | 0.00010 |
| 0.242 | 0.00009 | 0.00009 | 0.00009 | 0.00009 | 0.00009 | 0.00009 | 0.00009 | 0.00009 | 0.00010 | 0.00010 | 0.00010 | 0.00010 |
| 0.251 | 0.00009 | 0.00009 | 0.00009 | 0.00009 | 0.00009 | 0.00009 | 0.00009 | 0.00009 | 0.00009 | 0.00009 | 0.00009 | 0.00009 |
| 0.259 | 0.00009 | 0.00009 | 0.00009 | 0.00009 | 0.00009 | 0.00009 | 0.00009 | 0.00009 | 0.00009 | 0.00009 | 0.00009 | 0.00009 |
| 0.268 | 0.00009 | 0.00009 | 0.00009 | 0.00009 | 0.00009 | 0.00009 | 0.00009 | 0.00009 | 0.00009 | 0.00009 | 0.00009 | 0.00009 |
| 0.277 | 0.00008 | 0.00008 | 0.00008 | 0.00008 | 0.00009 | 0.00009 | 0.00009 | 0.00009 | 0.00009 | 0.00009 | 0.00009 | 0.00009 |
| 0.285 | 0.00008 | 0.00008 | 0.00008 | 0.00008 | 0.00009 | 0.00008 | 0.00008 | 0.00008 | 0.00009 | 0.00009 | 0.00009 | 0.00009 |
| 0.294 | 0.00008 | 0.00008 | 0.00008 | 0.00008 | 0.00008 | 0.00008 | 0.00008 | 0.00008 | 0.00008 | 0.00008 | 0.00008 | 0.00008 |
| 0.303 | 0.00008 | 0.00009 | 0.00008 | 0.00008 | 0.00008 | 0.00008 | 0.00008 | 0.00008 | 0.00008 | 0.00008 | 0.00008 | 0.00008 |
| 0.311 | 0.00008 | 0.00009 | 0.00008 | 0.00008 | 0.00008 | 0.00008 | 0.00008 | 0.00008 | 0.00008 | 0.00008 | 0.00008 | 0.00008 |
| 0.320 | 0.00009 | 0.00009 | 0.00008 | 0.00007 | 0.00008 | 0.00008 | 0.00008 | 0.00008 | 0.00008 | 0.00008 | 0.00008 | 0.00008 |
| 0.328 | 0.00009 | 0.00009 | 0.00009 | 0.00007 | 0.00008 | 0.00008 | 0.00008 | 0.00008 | 0.00008 | 0.00008 | 0.00008 | 0.00008 |
| 0.337 | 0.00013 | 0.00015 | 0.00010 | 0.00007 | 0.00008 | 0.00008 | 0.00008 | 0.00007 | 0.00008 | 0.00008 | 0.00008 | 0.00008 |
| 0.346 | 0.00015 | 0.00020 | 0.00011 | 0.00007 | 0.00008 | 0.00008 | 0.00007 | 0.00007 | 0.00008 | 0.00007 | 0.00007 | 0.00007 |
| 0.354 | 0.00014 | 0.00017 | 0.00015 | 0.00007 | 0.00008 | 0.00008 | 0.00007 | 0.00007 | 0.00007 | 0.00007 | 0.00007 | 0.00007 |
| 0.363 | 0.00017 | 0.00022 | 0.00029 | 0.00007 | 0.00008 | 0.00008 | 0.00007 | 0.00007 | 0.00007 | 0.00007 | 0.00007 | 0.00007 |
| 0.372 | 0.00015 | 0.00025 | 0.00044 | 0.00008 | 0.00008 | 0.00009 | 0.00007 | 0.00007 | 0.00007 | 0.00007 | 0.00007 | 0.00007 |
| 0.380 | 0.00018 | 0.00031 | 0.00050 | 0.00009 | 0.00008 | 0.00009 | 0.00007 | 0.00007 | 0.00007 | 0.00007 | 0.00007 | 0.00007 |
| 0.389 | 0.00018 | 0.00040 | 0.00073 | 0.00025 | 0.00007 | 0.00009 | 0.00007 | 0.00007 | 0.00007 | 0.00007 | 0.00007 | 0.00007 |
| 0.398 | 0.00019 | 0.00038 | 0.00062 | 0.00117 | 0.00007 | 0.00012 | 0.00008 | 0.00007 | 0.00007 | 0.00007 | 0.00007 | 0.00007 |
| 0.406 | 0.00020 | 0.00037 | 0.00066 | 0.00103 | 0.00009 | 0.00016 | 0.00008 | 0.00007 | 0.00007 | 0.00007 | 0.00007 | 0.00007 |
| 0.415 | 0.00019 | 0.00033 | 0.00067 | 0.00109 | 0.00010 | 0.00018 | 0.00009 | 0.00007 | 0.00007 | 0.00007 | 0.00007 | 0.00007 |
| 0.424 | 0.00018 | 0.00039 | 0.00069 | 0.00115 | 0.00011 | 0.00023 | 0.00009 | 0.00006 | 0.00007 | 0.00006 | 0.00007 | 0.00006 |
| 0.432 | 0.00020 | 0.00036 | 0.00074 | 0.00106 | 0.00011 | 0.00024 | 0.00010 | 0.00006 | 0.00006 | 0.00006 | 0.00006 | 0.00006 |
| 0.441 | 0.00016 | 0.00037 | 0.00086 | 0.00119 | 0.00011 | 0.00024 | 0.00013 | 0.00006 | 0.00006 | 0.00006 | 0.00006 | 0.00006 |
| 0.449 | 0.00015 | 0.00038 | 0.00090 | 0.00136 | 0.00014 | 0.00027 | 0.00014 | 0.00006 | 0.00006 | 0.00006 | 0.00006 | 0.00006 |
| 0.458 | 0.00014 | 0.00040 | 0.00106 | 0.00155 | 0.00014 | 0.00027 | 0.00014 | 0.00006 | 0.00006 | 0.00006 | 0.00006 | 0.00006 |
| 0.467 | 0.00011 | 0.00040 | 0.00129 | 0.00185 | 0.00018 | 0.00032 | 0.00021 | 0.00006 | 0.00006 | 0.00006 | 0.00006 | 0.00006 |
| 0.475 | 0.00013 | 0.00037 | 0.00158 | 0.00200 | 0.00019 | 0.00043 | 0.00027 | 0.00006 | 0.00006 | 0.00006 | 0.00006 | 0.00006 |
| 0.484 | 0.00011 | 0.00037 | 0.00176 | 0.00219 | 0.00026 | 0.00039 | 0.00028 | 0.00006 | 0.00006 | 0.00006 | 0.00006 | 0.00006 |
| 0.493 | 0.00014 | 0.00036 | 0.00190 | 0.00202 | 0.00028 | 0.00043 | 0.00035 | 0.00006 | 0.00006 | 0.00006 | 0.00006 | 0.00006 |
| 0.501 | 0.00012 | 0.00039 | 0.00208 | 0.00240 | 0.00025 | 0.00045 | 0.00047 | 0.00007 | 0.00006 | 0.00006 | 0.00006 | 0.00006 |
| 0.510 | 0.00011 | 0.00031 | 0.00206 | 0.00242 | 0.00026 | 0.00048 | 0.00067 | 0.00006 | 0.00006 | 0.00006 | 0.00006 | 0.00006 |
| 0.519 | 0.00011 | 0.00033 | 0.00211 | 0.00230 | 0.00031 | 0.00045 | 0.00073 | 0.00007 | 0.00006 | 0.00006 | 0.00006 | 0.00006 |
| 0.527 | 0.00013 | 0.00036 | 0.00212 | 0.00258 | 0.00033 | 0.00046 | 0.00082 | 0.00007 | 0.00006 | 0.00006 | 0.00006 | 0.00006 |
| 0.536 | 0.00017 | 0.00037 | 0.00207 | 0.00236 | 0.00034 | 0.00046 | 0.00089 | 0.00007 | 0.00006 | 0.00006 | 0.00006 | 0.00006 |
| 0.545 | 0.00016 | 0.00036 | 0.00209 | 0.00263 | 0.00031 | 0.00047 | 0.00097 | 0.00007 | 0.00006 | 0.00005 | 0.00006 | 0.00005 |
| 0.553 | 0.00018 | 0.00042 | 0.00197 | 0.00312 | 0.00035 | 0.00044 | 0.00094 | 0.00008 | 0.00005 | 0.00005 | 0.00005 | 0.00005 |
| 0.562 | 0.00019 | 0.00048 | 0.00204 | 0.00316 | 0.00038 | 0.00045 | 0.00095 | 0.00009 | 0.00005 | 0.00005 | 0.00005 | 0.00005 |
| 0.570 | 0.00025 | 0.00047 | 0.00196 | 0.00293 | 0.00038 | 0.00042 | 0.00093 | 0.00009 | 0.00005 | 0.00005 | 0.00005 | 0.00005 |
| 0.579 | 0.00027 | 0.00049 | 0.00198 | 0.00295 | 0.00039 | 0.00044 | 0.00100 | 0.00008 | 0.00005 | 0.00005 | 0.00005 | 0.00005 |
| 0.588 | 0.00025 | 0.00053 | 0.00236 | 0.00343 | 0.00040 | 0.00042 | 0.00116 | 0.00010 | 0.00005 | 0.00005 | 0.00005 | 0.00005 |
| 0.596 | 0.00028 | 0.00059 | 0.00251 | 0.00363 | 0.00039 | 0.00043 | 0.00116 | 0.00011 | 0.00005 | 0.00005 | 0.00005 | 0.00005 |
| 0.605 | 0.00036 | 0.00066 | 0.00280 | 0.00360 | 0.00043 | 0.00044 | 0.00121 | 0.00012 | 0.00005 | 0.00005 | 0.00005 | 0.00005 |
| 0.614 | 0.00039 | 0.00079 | 0.00312 | 0.00362 | 0.00045 | 0.00043 | 0.00124 | 0.00014 | 0.00005 | 0.00005 | 0.00005 | 0.00005 |
| 0.622 | 0.00045 | 0.00098 | 0.00337 | 0.00376 | 0.00051 | 0.00041 | 0.00127 | 0.00019 | 0.00005 | 0.00005 | 0.00005 | 0.00005 |
| 0.631 | 0.00051 | 0.00100 | 0.00346 | 0.00399 | 0.00058 | 0.00045 | 0.00130 | 0.00019 | 0.00005 | 0.00005 | 0.00005 | 0.00005 |
| 0.640 | 0.00056 | 0.00092 | 0.00357 | 0.00419 | 0.00067 | 0.00057 | 0.00135 | 0.00023 | 0.00005 | 0.00005 | 0.00005 | 0.00005 |
| 0.648 | 0.00062 | 0.00113 | 0.00358 | 0.00462 | 0.00069 | 0.00056 | 0.00144 | 0.00030 | 0.00005 | 0.00005 | 0.00005 | 0.00005 |
| 0.657 | 0.00068 | 0.00112 | 0.00344 | 0.00444 | 0.00069 | 0.00054 | 0.00145 | 0.00031 | 0.00005 | 0.00005 | 0.00005 | 0.00005 |
| 0.666 | 0.00077 | 0.00116 | 0.00344 | 0.00440 | 0.00075 | 0.00057 | 0.00149 | 0.00032 | 0.00005 | 0.00005 | 0.00005 | 0.00005 |
| 0.674 | 0.00083 | 0.00126 | 0.00339 | 0.00439 | 0.00079 | 0.00061 | 0.00160 | 0.00040 | 0.00005 | 0.00005 | 0.00005 | 0.00005 |
| 0.683 | 0.00085 | 0.00146 | 0.00345 | 0.00418 | 0.00078 | 0.00061 | 0.00161 | 0.00044 | 0.00005 | 0.00005 | 0.00005 | 0.00005 |
| 0.691 | 0.00094 | 0.00164 | 0.00347 | 0.00393 | 0.00084 | 0.00066 | 0.00165 | 0.00049 | 0.00005 | 0.00005 | 0.00005 | 0.00005 |
| 0.700 | 0.00101 | 0.00177 | 0.00327 | 0.00365 | 0.00086 | 0.00075 | 0.00162 | 0.00054 | 0.00005 | 0.00005 | 0.00005 | 0.00005 |
| 0.709 | 0.00108 | 0.00188 | 0.00315 | 0.00342 | 0.00093 | 0.00084 | 0.00160 | 0.00056 | 0.00005 | 0.00005 | 0.00005 | 0.00005 |
| 0.717 | 0.00109 | 0.00201 | 0.00320 | 0.00322 | 0.00093 | 0.00088 | 0.00156 | 0.00064 | 0.00005 | 0.00005 | 0.00005 | 0.00005 |
| 0.726 | 0.00120 | 0.00198 | 0.00317 | 0.00304 | 0.00098 | 0.00087 | 0.00161 | 0.00070 | 0.00005 | 0.00005 | 0.00005 | 0.00004 |
| 0.735 | 0.00131 | 0.00220 | 0.00322 | 0.00292 | 0.00100 | 0.00094 | 0.00172 | 0.00077 | 0.00005 | 0.00005 | 0.00004 | 0.00004 |
| 0.743 | 0.00138 | 0.00232 | 0.00338 | 0.00282 | 0.00105 | 0.00103 | 0.00181 | 0.00086 | 0.00005 | 0.00004 | 0.00004 | 0.00004 |
| 0.752 | 0.00144 | 0.00235 | 0.00343 | 0.00269 | 0.00102 | 0.00101 | 0.00189 | 0.00092 | 0.00005 | 0.00004 | 0.00004 | 0.00004 |
| 0.761 | 0.00150 | 0.00232 | 0.00366 | 0.00261 | 0.00097 | 0.00108 | 0.00196 | 0.00097 | 0.00004 | 0.00004 | 0.00004 | 0.00004 |
| 0.769 | 0.00169 | 0.00231 | 0.00355 | 0.00260 | 0.00100 | 0.00113 | 0.00196 | 0.00101 | 0.00004 | 0.00004 | 0.00004 | 0.00004 |
| 0.778 | 0.00176 | 0.00238 | 0.00352 | 0.00268 | 0.00097 | 0.00110 | 0.00203 | 0.00108 | 0.00004 | 0.00004 | 0.00004 | 0.00004 |
| 0.787 | 0.00186 | 0.00240 | 0.00342 | 0.00266 | 0.00098 | 0.00115 | 0.00207 | 0.00113 | 0.00004 | 0.00004 | 0.00004 | 0.00004 |
| 0.795 | 0.00191 | 0.00261 | 0.00334 | 0.00265 | 0.00102 | 0.00114 | 0.00206 | 0.00116 | 0.00004 | 0.00004 | 0.00004 | 0.00004 |
| 0.804 | 0.00207 | 0.00297 | 0.00327 | 0.00258 | 0.00108 | 0.00116 | 0.00213 | 0.00120 | 0.00004 | 0.00004 | 0.00004 | 0.00004 |
| 0.812 | 0.00226 | 0.00317 | 0.00313 | 0.00254 | 0.00106 | 0.00127 | 0.00211 | 0.00118 | 0.00004 | 0.00004 | 0.00004 | 0.00004 |
| 0.821 | 0.00248 | 0.00347 | 0.00304 | 0.00252 | 0.00103 | 0.00131 | 0.00219 | 0.00124 | 0.00004 | 0.00004 | 0.00004 | 0.00004 |
| 0.830 | 0.00261 | 0.00364 | 0.00303 | 0.00238 | 0.00105 | 0.00131 | 0.00227 | 0.00127 | 0.00004 | 0.00004 | 0.00004 | 0.00004 |
| 0.838 | 0.00280 | 0.00347 | 0.00297 | 0.00244 | 0.00111 | 0.00127 | 0.00221 | 0.00129 | 0.00004 | 0.00004 | 0.00004 | 0.00004 |
| 0.847 | 0.00317 | 0.00338 | 0.00297 | 0.00244 | 0.00114 | 0.00128 | 0.00218 | 0.00134 | 0.00004 | 0.00004 | 0.00004 | 0.00004 |
| 0.856 | 0.00332 | 0.00312 | 0.00297 | 0.00244 | 0.00115 | 0.00129 | 0.00212 | 0.00133 | 0.00004 | 0.00004 | 0.00004 | 0.00004 |
| 0.864 | 0.00345 | 0.00292 | 0.00295 | 0.00237 | 0.00117 | 0.00128 | 0.00214 | 0.00138 | 0.00004 | 0.00004 | 0.00004 | 0.00004 |
| 0.873 | 0.00344 | 0.00290 | 0.00300 | 0.00252 | 0.00124 | 0.00128 | 0.00209 | 0.00139 | 0.00004 | 0.00004 | 0.00004 | 0.00004 |
| 0.882 | 0.00344 | 0.00288 | 0.00292 | 0.00248 | 0.00129 | 0.00131 | 0.00212 | 0.00142 | 0.00004 | 0.00004 | 0.00004 | 0.00004 |
| 0.890 | 0.00343 | 0.00298 | 0.00293 | 0.00242 | 0.00127 | 0.00131 | 0.00216 | 0.00141 | 0.00004 | 0.00004 | 0.00004 | 0.00004 |
| 0.899 | 0.00340 | 0.00299 | 0.00310 | 0.00249 | 0.00133 | 0.00138 | 0.00222 | 0.00141 | 0.00004 | 0.00004 | 0.00004 | 0.00004 |
| 0.908 | 0.00339 | 0.00304 | 0.00311 | 0.00244 | 0.00137 | 0.00135 | 0.00223 | 0.00140 | 0.00004 | 0.00004 | 0.00004 | 0.00004 |
| 0.916 | 0.00342 | 0.00315 | 0.00300 | 0.00244 | 0.00143 | 0.00138 | 0.00225 | 0.00140 | 0.00004 | 0.00004 | 0.00004 | 0.00004 |
| 0.925 | 0.00335 | 0.00311 | 0.00282 | 0.00251 | 0.00145 | 0.00136 | 0.00224 | 0.00141 | 0.00004 | 0.00004 | 0.00004 | 0.00004 |
| 0.933 | 0.00331 | 0.00315 | 0.00267 | 0.00261 | 0.00151 | 0.00138 | 0.00225 | 0.00140 | 0.00004 | 0.00004 | 0.00004 | 0.00004 |
| 0.942 | 0.00323 | 0.00318 | 0.00254 | 0.00269 | 0.00154 | 0.00133 | 0.00226 | 0.00142 | 0.00004 | 0.00004 | 0.00004 | 0.00004 |
| 0.951 | 0.00328 | 0.00317 | 0.00246 | 0.00287 | 0.00160 | 0.00143 | 0.00226 | 0.00141 | 0.00004 | 0.00004 | 0.00004 | 0.00004 |
| 0.959 | 0.00325 | 0.00327 | 0.00233 | 0.00288 | 0.00163 | 0.00146 | 0.00229 | 0.00143 | 0.00004 | 0.00004 | 0.00004 | 0.00004 |
| 0.968 | 0.00324 | 0.00339 | 0.00229 | 0.00279 | 0.00171 | 0.00144 | 0.00227 | 0.00141 | 0.00004 | 0.00004 | 0.00004 | 0.00004 |
| 0.977 | 0.00322 | 0.00333 | 0.00227 | 0.00265 | 0.00171 | 0.00143 | 0.00229 | 0.00142 | 0.00004 | 0.00004 | 0.00004 | 0.00004 |
| 0.985 | 0.00315 | 0.00317 | 0.00228 | 0.00243 | 0.00179 | 0.00141 | 0.00228 | 0.00141 | 0.00004 | 0.00004 | 0.00004 | 0.00004 |
| 0.994 | 0.00310 | 0.00303 | 0.00230 | 0.00222 | 0.00184 | 0.00139 | 0.00226 | 0.00145 | 0.00004 | 0.00004 | 0.00004 | 0.00004 |
| 1.000 | 0.00303 | 0.00295 | 0.00217 | 0.00203 | 0.00190 | 0.00141 | 0.00225 | 0.00145 | 0.00004 | 0.00004 | 0.00004 | 0.00004 |
| 1.010 | 0.00296 | 0.00293 | 0.00221 | 0.00187 | 0.00195 | 0.00140 | 0.00232 | 0.00144 | 0.00004 | 0.00004 | 0.00004 | 0.00004 |
| 1.020 | 0.00285 | 0.00292 | 0.00220 | 0.00172 | 0.00205 | 0.00140 | 0.00230 | 0.00144 | 0.00004 | 0.00004 | 0.00004 | 0.00003 |
| 1.030 | 0.00279 | 0.00294 | 0.00220 | 0.00161 | 0.00212 | 0.00135 | 0.00224 | 0.00148 | 0.00004 | 0.00004 | 0.00004 | 0.00003 |
| 1.040 | 0.00273 | 0.00295 | 0.00225 | 0.00151 | 0.00215 | 0.00132 | 0.00230 | 0.00147 | 0.00004 | 0.00004 | 0.00004 | 0.00003 |
| 1.050 | 0.00267 | 0.00294 | 0.00230 | 0.00143 | 0.00219 | 0.00136 | 0.00232 | 0.00148 | 0.00004 | 0.00004 | 0.00003 | 0.00003 |
| 1.050 | 0.00267 | 0.00285 | 0.00231 | 0.00137 | 0.00222 | 0.00139 | 0.00232 | 0.00156 | 0.00004 | 0.00004 | 0.00003 | 0.00003 |
| 1.060 | 0.00266 | 0.00284 | 0.00225 | 0.00132 | 0.00227 | 0.00139 | 0.00234 | 0.00154 | 0.00004 | 0.00004 | 0.00003 | 0.00003 |
| 1.070 | 0.00261 | 0.00287 | 0.00219 | 0.00127 | 0.00234 | 0.00144 | 0.00236 | 0.00154 | 0.00004 | 0.00004 | 0.00003 | 0.00003 |
| 1.080 | 0.00262 | 0.00291 | 0.00213 | 0.00123 | 0.00235 | 0.00141 | 0.00235 | 0.00154 | 0.00004 | 0.00004 | 0.00003 | 0.00003 |
| 1.090 | 0.00257 | 0.00286 | 0.00205 | 0.00122 | 0.00238 | 0.00149 | 0.00233 | 0.00157 | 0.00004 | 0.00004 | 0.00003 | 0.00003 |
| 1.100 | 0.00256 | 0.00290 | 0.00196 | 0.00121 | 0.00240 | 0.00151 | 0.00233 | 0.00159 | 0.00004 | 0.00004 | 0.00003 | 0.00003 |
| 1.110 | 0.00265 | 0.00300 | 0.00186 | 0.00121 | 0.00248 | 0.00150 | 0.00232 | 0.00160 | 0.00004 | 0.00004 | 0.00003 | 0.00003 |
| 1.110 | 0.00258 | 0.00312 | 0.00181 | 0.00120 | 0.00246 | 0.00154 | 0.00234 | 0.00159 | 0.00004 | 0.00004 | 0.00003 | 0.00003 |
| 1.120 | 0.00257 | 0.00312 | 0.00176 | 0.00122 | 0.00240 | 0.00152 | 0.00238 | 0.00162 | 0.00004 | 0.00004 | 0.00003 | 0.00003 |
| 1.130 | 0.00254 | 0.00296 | 0.00173 | 0.00120 | 0.00247 | 0.00149 | 0.00236 | 0.00162 | 0.00003 | 0.00004 | 0.00003 | 0.00003 |
| 1.140 | 0.00256 | 0.00281 | 0.00184 | 0.00118 | 0.00243 | 0.00152 | 0.00235 | 0.00161 | 0.00003 | 0.00004 | 0.00003 | 0.00003 |
| 1.150 | 0.00252 | 0.00269 | 0.00184 | 0.00120 | 0.00239 | 0.00157 | 0.00233 | 0.00164 | 0.00003 | 0.00005 | 0.00003 | 0.00003 |
| 1.160 | 0.00248 | 0.00265 | 0.00189 | 0.00122 | 0.00238 | 0.00157 | 0.00236 | 0.00164 | 0.00003 | 0.00004 | 0.00003 | 0.00003 |
| 1.170 | 0.00249 | 0.00253 | 0.00205 | 0.00122 | 0.00229 | 0.00161 | 0.00234 | 0.00164 | 0.00003 | 0.00004 | 0.00003 | 0.00003 |
| 1.180 | 0.00241 | 0.00248 | 0.00205 | 0.00119 | 0.00231 | 0.00162 | 0.00237 | 0.00166 | 0.00003 | 0.00005 | 0.00003 | 0.00003 |
| 1.180 | 0.00242 | 0.00243 | 0.00222 | 0.00120 | 0.00230 | 0.00165 | 0.00236 | 0.00167 | 0.00003 | 0.00005 | 0.00003 | 0.00003 |
| 1.190 | 0.00241 | 0.00235 | 0.00230 | 0.00121 | 0.00225 | 0.00169 | 0.00237 | 0.00169 | 0.00003 | 0.00005 | 0.00003 | 0.00003 |
| 1.200 | 0.00239 | 0.00235 | 0.00250 | 0.00121 | 0.00222 | 0.00174 | 0.00238 | 0.00172 | 0.00003 | 0.00005 | 0.00003 | 0.00003 |
| 1.210 | 0.00241 | 0.00242 | 0.00254 | 0.00123 | 0.00216 | 0.00186 | 0.00240 | 0.00176 | 0.00003 | 0.00005 | 0.00003 | 0.00003 |
| 1.220 | 0.00238 | 0.00243 | 0.00251 | 0.00126 | 0.00213 | 0.00185 | 0.00240 | 0.00178 | 0.00003 | 0.00005 | 0.00003 | 0.00003 |
| 1.230 | 0.00241 | 0.00240 | 0.00240 | 0.00134 | 0.00209 | 0.00186 | 0.00242 | 0.00179 | 0.00003 | 0.00005 | 0.00003 | 0.00003 |
| 1.240 | 0.00244 | 0.00234 | 0.00225 | 0.00142 | 0.00209 | 0.00191 | 0.00240 | 0.00182 | 0.00003 | 0.00005 | 0.00003 | 0.00003 |
| 1.240 | 0.00241 | 0.00231 | 0.00214 | 0.00151 | 0.00207 | 0.00197 | 0.00246 | 0.00184 | 0.00003 | 0.00005 | 0.00003 | 0.00003 |
| 1.250 | 0.00246 | 0.00227 | 0.00194 | 0.00166 | 0.00205 | 0.00202 | 0.00246 | 0.00188 | 0.00003 | 0.00005 | 0.00003 | 0.00003 |
| 1.260 | 0.00245 | 0.00221 | 0.00177 | 0.00171 | 0.00202 | 0.00203 | 0.00246 | 0.00190 | 0.00003 | 0.00005 | 0.00003 | 0.00003 |
| 1.270 | 0.00244 | 0.00210 | 0.00166 | 0.00167 | 0.00199 | 0.00205 | 0.00248 | 0.00188 | 0.00003 | 0.00005 | 0.00003 | 0.00003 |
| 1.280 | 0.00242 | 0.00203 | 0.00164 | 0.00158 | 0.00197 | 0.00203 | 0.00247 | 0.00194 | 0.00003 | 0.00006 | 0.00003 | 0.00003 |
| 1.290 | 0.00240 | 0.00199 | 0.00164 | 0.00144 | 0.00194 | 0.00201 | 0.00249 | 0.00195 | 0.00003 | 0.00006 | 0.00003 | 0.00003 |
| 1.300 | 0.00238 | 0.00197 | 0.00163 | 0.00130 | 0.00190 | 0.00203 | 0.00251 | 0.00197 | 0.00003 | 0.00005 | 0.00003 | 0.00003 |
| 1.310 | 0.00234 | 0.00198 | 0.00170 | 0.00118 | 0.00186 | 0.00212 | 0.00248 | 0.00201 | 0.00003 | 0.00005 | 0.00003 | 0.00003 |
| 1.310 | 0.00236 | 0.00200 | 0.00173 | 0.00110 | 0.00184 | 0.00209 | 0.00249 | 0.00206 | 0.00003 | 0.00007 | 0.00003 | 0.00003 |
| 1.320 | 0.00241 | 0.00201 | 0.00165 | 0.00103 | 0.00182 | 0.00211 | 0.00248 | 0.00206 | 0.00003 | 0.00008 | 0.00003 | 0.00003 |
| 1.330 | 0.00240 | 0.00201 | 0.00160 | 0.00097 | 0.00177 | 0.00209 | 0.00250 | 0.00209 | 0.00003 | 0.00007 | 0.00003 | 0.00003 |
| 1.340 | 0.00232 | 0.00200 | 0.00161 | 0.00092 | 0.00171 | 0.00214 | 0.00251 | 0.00214 | 0.00003 | 0.00006 | 0.00003 | 0.00003 |
| 1.350 | 0.00234 | 0.00201 | 0.00157 | 0.00088 | 0.00168 | 0.00218 | 0.00251 | 0.00216 | 0.00003 | 0.00006 | 0.00003 | 0.00003 |
| 1.360 | 0.00230 | 0.00198 | 0.00157 | 0.00087 | 0.00166 | 0.00218 | 0.00252 | 0.00216 | 0.00003 | 0.00006 | 0.00003 | 0.00003 |
| 1.370 | 0.00231 | 0.00192 | 0.00168 | 0.00085 | 0.00164 | 0.00215 | 0.00251 | 0.00217 | 0.00003 | 0.00006 | 0.00003 | 0.00003 |
| 1.370 | 0.00226 | 0.00189 | 0.00180 | 0.00085 | 0.00160 | 0.00221 | 0.00252 | 0.00218 | 0.00003 | 0.00006 | 0.00003 | 0.00003 |
| 1.380 | 0.00223 | 0.00189 | 0.00186 | 0.00085 | 0.00155 | 0.00229 | 0.00250 | 0.00219 | 0.00003 | 0.00007 | 0.00003 | 0.00003 |
| 1.390 | 0.00224 | 0.00187 | 0.00178 | 0.00084 | 0.00153 | 0.00234 | 0.00248 | 0.00222 | 0.00003 | 0.00006 | 0.00003 | 0.00003 |
| 1.400 | 0.00225 | 0.00185 | 0.00164 | 0.00079 | 0.00151 | 0.00241 | 0.00251 | 0.00223 | 0.00003 | 0.00007 | 0.00003 | 0.00003 |
| 1.410 | 0.00223 | 0.00184 | 0.00151 | 0.00080 | 0.00147 | 0.00238 | 0.00249 | 0.00223 | 0.00003 | 0.00008 | 0.00003 | 0.00003 |
| 1.420 | 0.00222 | 0.00182 | 0.00134 | 0.00080 | 0.00144 | 0.00248 | 0.00249 | 0.00225 | 0.00003 | 0.00007 | 0.00003 | 0.00003 |
| 1.430 | 0.00225 | 0.00183 | 0.00129 | 0.00085 | 0.00141 | 0.00245 | 0.00251 | 0.00227 | 0.00003 | 0.00007 | 0.00003 | 0.00003 |
| 1.430 | 0.00232 | 0.00191 | 0.00125 | 0.00085 | 0.00138 | 0.00250 | 0.00251 | 0.00227 | 0.00003 | 0.00007 | 0.00003 | 0.00003 |
| 1.440 | 0.00230 | 0.00198 | 0.00120 | 0.00086 | 0.00136 | 0.00250 | 0.00249 | 0.00230 | 0.00003 | 0.00007 | 0.00003 | 0.00003 |
| 1.450 | 0.00229 | 0.00197 | 0.00116 | 0.00086 | 0.00133 | 0.00249 | 0.00257 | 0.00233 | 0.00003 | 0.00006 | 0.00003 | 0.00003 |
| 1.460 | 0.00228 | 0.00194 | 0.00118 | 0.00092 | 0.00129 | 0.00255 | 0.00253 | 0.00231 | 0.00003 | 0.00006 | 0.00003 | 0.00003 |
| 1.470 | 0.00232 | 0.00195 | 0.00115 | 0.00090 | 0.00130 | 0.00266 | 0.00252 | 0.00239 | 0.00003 | 0.00005 | 0.00003 | 0.00003 |
| 1.480 | 0.00236 | 0.00198 | 0.00114 | 0.00097 | 0.00128 | 0.00260 | 0.00251 | 0.00241 | 0.00003 | 0.00007 | 0.00003 | 0.00003 |
| 1.490 | 0.00248 | 0.00205 | 0.00111 | 0.00108 | 0.00130 | 0.00269 | 0.00253 | 0.00245 | 0.00003 | 0.00008 | 0.00003 | 0.00003 |
| 1.500 | 0.00248 | 0.00210 | 0.00108 | 0.00105 | 0.00125 | 0.00289 | 0.00256 | 0.00246 | 0.00003 | 0.00008 | 0.00003 | 0.00003 |
| 1.500 | 0.00244 | 0.00219 | 0.00110 | 0.00103 | 0.00122 | 0.00276 | 0.00254 | 0.00249 | 0.00003 | 0.00009 | 0.00003 | 0.00003 |
| 1.510 | 0.00240 | 0.00242 | 0.00112 | 0.00106 | 0.00121 | 0.00290 | 0.00254 | 0.00255 | 0.00004 | 0.00007 | 0.00003 | 0.00003 |
| 1.520 | 0.00246 | 0.00247 | 0.00110 | 0.00105 | 0.00119 | 0.00296 | 0.00257 | 0.00260 | 0.00004 | 0.00006 | 0.00003 | 0.00003 |
| 1.530 | 0.00244 | 0.00244 | 0.00123 | 0.00105 | 0.00119 | 0.00278 | 0.00255 | 0.00266 | 0.00003 | 0.00009 | 0.00003 | 0.00003 |
| 1.540 | 0.00245 | 0.00246 | 0.00121 | 0.00105 | 0.00116 | 0.00284 | 0.00255 | 0.00272 | 0.00003 | 0.00007 | 0.00003 | 0.00003 |
| 1.550 | 0.00245 | 0.00241 | 0.00125 | 0.00104 | 0.00117 | 0.00280 | 0.00254 | 0.00276 | 0.00003 | 0.00007 | 0.00003 | 0.00003 |
| 1.560 | 0.00247 | 0.00242 | 0.00136 | 0.00102 | 0.00118 | 0.00279 | 0.00254 | 0.00285 | 0.00004 | 0.00008 | 0.00003 | 0.00003 |
| 1.560 | 0.00251 | 0.00237 | 0.00136 | 0.00099 | 0.00118 | 0.00280 | 0.00255 | 0.00292 | 0.00004 | 0.00008 | 0.00003 | 0.00002 |
| 1.570 | 0.00249 | 0.00227 | 0.00138 | 0.00096 | 0.00118 | 0.00285 | 0.00258 | 0.00301 | 0.00003 | 0.00009 | 0.00003 | 0.00002 |
| 1.580 | 0.00252 | 0.00229 | 0.00144 | 0.00093 | 0.00114 | 0.00285 | 0.00258 | 0.00307 | 0.00004 | 0.00009 | 0.00003 | 0.00002 |
| 1.590 | 0.00247 | 0.00228 | 0.00145 | 0.00090 | 0.00117 | 0.00282 | 0.00258 | 0.00318 | 0.00004 | 0.00010 | 0.00003 | 0.00002 |
| 1.600 | 0.00245 | 0.00224 | 0.00153 | 0.00088 | 0.00119 | 0.00281 | 0.00261 | 0.00325 | 0.00004 | 0.00008 | 0.00003 | 0.00002 |
| 1.610 | 0.00248 | 0.00225 | 0.00157 | 0.00086 | 0.00120 | 0.00286 | 0.00263 | 0.00337 | 0.00004 | 0.00008 | 0.00003 | 0.00002 |
| 1.620 | 0.00250 | 0.00227 | 0.00158 | 0.00087 | 0.00121 | 0.00285 | 0.00263 | 0.00348 | 0.00004 | 0.00008 | 0.00003 | 0.00002 |
| 1.620 | 0.00250 | 0.00233 | 0.00160 | 0.00089 | 0.00123 | 0.00283 | 0.00265 | 0.00358 | 0.00004 | 0.00008 | 0.00003 | 0.00002 |
| 1.630 | 0.00252 | 0.00232 | 0.00161 | 0.00092 | 0.00124 | 0.00278 | 0.00266 | 0.00371 | 0.00004 | 0.00010 | 0.00003 | 0.00002 |
| 1.640 | 0.00252 | 0.00224 | 0.00162 | 0.00097 | 0.00126 | 0.00275 | 0.00268 | 0.00382 | 0.00004 | 0.00010 | 0.00003 | 0.00002 |
| 1.650 | 0.00253 | 0.00224 | 0.00166 | 0.00105 | 0.00132 | 0.00274 | 0.00272 | 0.00395 | 0.00004 | 0.00010 | 0.00003 | 0.00002 |
| 1.660 | 0.00253 | 0.00227 | 0.00170 | 0.00112 | 0.00133 | 0.00278 | 0.00273 | 0.00407 | 0.00004 | 0.00012 | 0.00003 | 0.00002 |
| 1.670 | 0.00253 | 0.00224 | 0.00166 | 0.00119 | 0.00134 | 0.00277 | 0.00276 | 0.00420 | 0.00004 | 0.00012 | 0.00003 | 0.00002 |
| 1.680 | 0.00260 | 0.00218 | 0.00171 | 0.00129 | 0.00135 | 0.00271 | 0.00277 | 0.00434 | 0.00004 | 0.00010 | 0.00003 | 0.00002 |
| 1.690 | 0.00249 | 0.00214 | 0.00171 | 0.00135 | 0.00143 | 0.00270 | 0.00278 | 0.00447 | 0.00004 | 0.00011 | 0.00003 | 0.00002 |
| 1.690 | 0.00251 | 0.00216 | 0.00168 | 0.00141 | 0.00143 | 0.00272 | 0.00280 | 0.00461 | 0.00004 | 0.00012 | 0.00003 | 0.00002 |
| 1.700 | 0.00255 | 0.00217 | 0.00168 | 0.00150 | 0.00146 | 0.00271 | 0.00286 | 0.00475 | 0.00004 | 0.00011 | 0.00003 | 0.00002 |
| 1.710 | 0.00257 | 0.00218 | 0.00171 | 0.00155 | 0.00145 | 0.00270 | 0.00288 | 0.00490 | 0.00004 | 0.00010 | 0.00003 | 0.00002 |
| 1.720 | 0.00257 | 0.00221 | 0.00169 | 0.00156 | 0.00149 | 0.00268 | 0.00287 | 0.00499 | 0.00004 | 0.00012 | 0.00003 | 0.00002 |
